# Pd(ii)-catalyzed remote regiodivergent *ortho*- and *meta*-C–H functionalizations of phenylethylamines†
†Electronic supplementary information (ESI) available: Experimental procedures and data for new compounds. CCDC 1056709 and 1406595. For ESI and crystallographic data in CIF or other electronic format see DOI: 10.1039/c5sc01737h


**DOI:** 10.1039/c5sc01737h

**Published:** 2015-06-25

**Authors:** Shangda Li, Huafang Ji, Lei Cai, Gang Li

**Affiliations:** a Key Laboratory of Coal to Ethylene Glycol and Its Related Technology , Fujian Institute of Research on the Structure of Matter , Chinese Academy of Sciences , 155 West Yang-Qiao Road , Fuzhou , Fujian 350002 , P. R. China . Email: gangli@fjirsm.ac.cn; b State Key Laboratory of Structural Chemistry , Chinese Academy of Sciences , Fuzhou , Fujian 350002 , P. R. China

## Abstract

Site selectivity control is of vital importance in the direct functionalization of inert C–H bonds. Reported here is a novel example of remote regiodivergent *ortho*- and *meta*-C–H bond functionalizations of phenylethylamine derivatives by using a novel 2-cyanobenzoyl group as the original directing functionality, where the regioselectivity was adjusted by a methylation. The potential of the method was exemplified by sequential functionalizations of both *ortho*- and *meta*-C–H bonds of a phenylethylamine derivative in a streamlined manner.

## Abstract 2

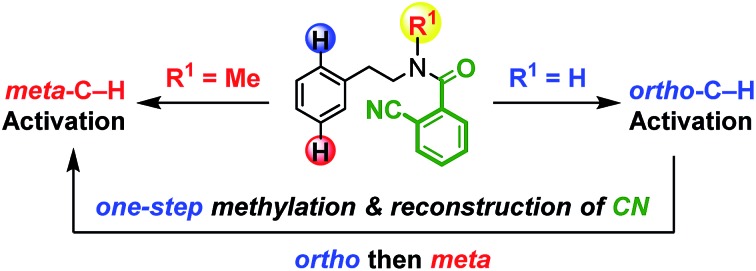
A methylation switches the remote regioselectivity of C–H functionalizations of phenylethylamines.

## Introduction

Controlling site selectivity is an outstanding challenge in the direct functionalization of inert C–H bonds that are ubiquitous in organic molecules.^1^ The increasing applications of these type of transformations in organic synthesis also demand accessibility to diverse site selectivities.^2^ While numerous directing groups have been introduced to assist the cleavage of proximal *ortho*-C–H bonds in most cases with transition metals,^1^,^3^–^10^ directing group assisted *meta*-selective C–H functionalization of arenes has proved especially challenging and is still very rare.^5^,^6^,^8^,^9^ In 2009, a remarkable breakthrough was reported by Gaunt *et al.*, who developed a carbonyl group directed unprecedented *meta*-selective C–H arylation of anilides by using a Cu(ii) catalyst and diaryliodonium salts.5*a* This method was later extended to α-aryl carbonyl compounds by the same group.5*b* Another impressive breakthrough came from the Frost group, who introduced an ingenious method of *meta*-selective C–H sulfonation of 2-phenylpyridines *via* cyclometalated ruthenium intermediates.6a,b A similar strategy was then employed by Ackermann to realize a *meta*-selective C–H alkylation with secondary alkyl halides.6*c* Recently, a small number of ground-breaking examples of Pd(ii) catalyzed directed *meta*-selective C–H functionalizations of arenes that were attached with elegantly devised nitrile-based templates were disclosed, pioneered by Yu and then further studied by Tan and Maiti.^8^ By using the above directing group assisted *meta*-selective C–H functionalization of arenes, elegant regiodivergent functionalizations of *ortho*- and *meta*-C–H bonds have been reported by Gaunt,4b,5b Frost6*b* and Yu,8*b* and examples of reactions reported by Gaunt4b,5b and Frost6*b* could even be performed sequentially.8i,^11^,^12^ However, the use of analogous directing groups to achieve *remote-selective* regiodivergent activation of *ortho*- and *meta*-C–H bonds has not been examined and remains a significant challenge.^13^,^14^ We envision that such methodology is highly desirable for drug discovery and material sciences, since it only requires a single operation to achieve a different remote regioselectivity.2*f* Herein, we report a novel strategy for regiodivergent *ortho*- and *meta*-C–H functionalizations of phenylethylamine derivatives.

**Fig. 1:**
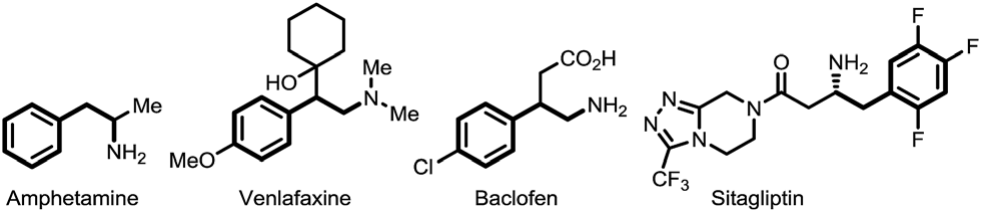
Representative drugs containing a phenylethylamine core.

**Scheme 1:**
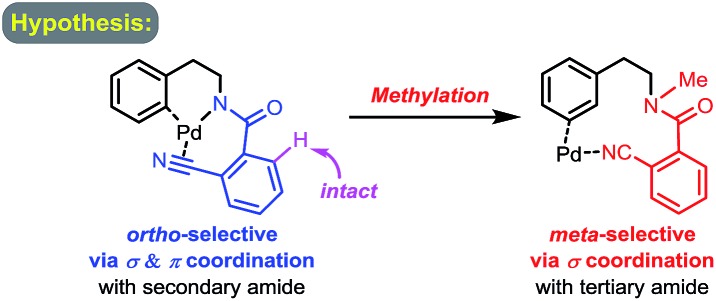
Hypothesis of regioselectivity changed by a methylation.

To test our hypothesis of a regiodivergent C–H functionalization strategy by using analogous directing groups, we selected phenylethylamines as the testing compounds, since they are a class of aromatic compounds that are important core structures of numerous drug molecules (Fig. 1). Moreover, although *ortho*-C–H functionalizations have been reported for phenylethylamine derivatives, their *meta*-selective C–H functionalization remains elusive.^15^ Inspired by recent studies on directed *meta*-selective C–H functionalizations of arenes,^8^ we proposed that a 2-cyanobenzoyl group could act as the key directing functionality for both *ortho*- and *meta*-C–H functionalizations of phenylethylamines with a Pd(ii) catalyst by taking advantage of the σ and π coordination ability of the nitrile group (Scheme 1).^16^ However, during our study we found that our proposed mode of *ortho*-selective C–H bond cleavage was not feasible and a novel remote-selective *ortho*-C–H bond cleavage was observed instead (*vide infra*).^13^,^14^

## Results and discussion

**Scheme 2:**
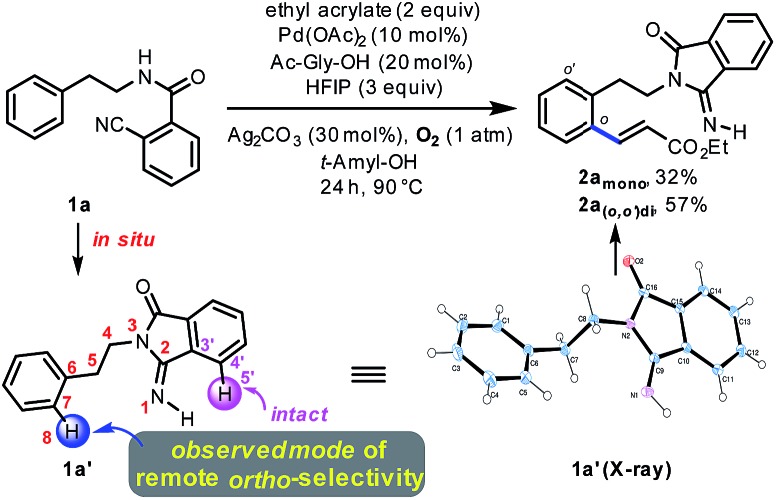
A novel remote-selective *ortho*-C–H olefination.

To examine our original hypothesis (Scheme 2), we chose olefination as the model reaction.1o,^17^ After extensive condition screening with Pd(OAc)_2_ as the catalyst (see ESI†), we were able to produce a high combined yield of *ortho*-olefinated products by treating **1a** with ethyl acrylate under oxygen with hexafluoroisopropanol (HFIP) as an additive and *N*-acetyl-glycine (Ac-Gly-OH) ligand.8a,^18^ Interestingly, the 2-cyanobenzoyl motif cyclized to an imidamide derivative in the products. To ascertain the mechanism of this olefination, **1a** was subjected to the above reaction conditions without adding ethyl acrylate, affording **1a′** that was believed to be the reactive substrate for the olefination. Indeed, after **1a′** was treated with the same olefination conditions, the desired products were generated in similar yields (see ESI†). Although this reaction pathway is not desired from our original hypothesis, the site selectivity of this reaction is surprisingly uncommon since the imino group of **1a′**, the most likely directing group on **1a′**, directed the cleavage of a remote *ortho*-C–H bond rather than a proximal *ortho*-C–H bond on the arene attached to the imidamide, which is in marked contrast to the *ortho*-C–H functionalizations of arylimine derivatives.^19^ The exact origin of the selectivity is unclear at present, and the study of the mechanism is under way.^14^

Several representative substrates were then surveyed briefly (Table 1). It was found that electron-withdrawing groups like chloride and fluoride were tolerated (**2b–c**), giving good yields of desired products. Good to excellent yields of products were also generated with substrates containing electron-donating groups such as methyl at the *meta*-position (**2d**) and methoxy at the *ortho*- (**2e**) and *para*-position (**2f**).

**Table 1:** Representative substrates of remote *ortho*-C–H olefination^*a*^

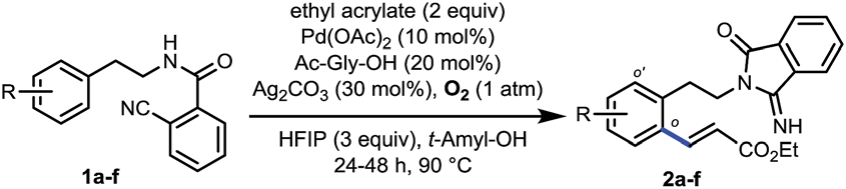
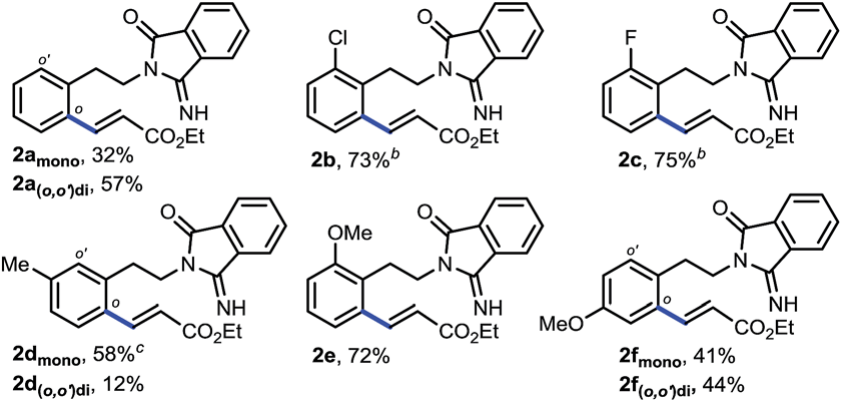

^*a*^Reaction conditions: **1** (0.2 mmol), ethyl acrylate (0.4 mmol), Pd(OAc)_2_ (10 mol%), Ac-Gly-OH (20 mol%), HFIP (0.6 mmol), Ag_2_CO_3_ (0.06 mmol), O_2_ (1 atm), *t*-amyl-OH (2 mL), 24–48 h, 90 °C. Isolated yields are reported, see the ESI for details.

^*b*^80 °C.

^*c*^70 °C.

Having established the remote-selective *ortho*-C–H olefination of the secondary phenylethylamide, we were eager to test whether the selectivity could be switched to a remote-selective *meta*-C–H olefination after the secondary amide is methylated into a tertiary one (see the ESI† for methylation with MeI). Starting with the above *ortho*-olefination reaction conditions, we were very delighted to find that *N*-methyl amide **3a** could lead to a 10% yield of the desired product with a trace of other regioisomers (Table 2, entry 1). Inspired by the previous discovery that HFIP was a compatible solvent with nitrile-based templates,^8^ we switched the solvent to HFIP and found that the combined yield of desired products was increased dramatically to 58% with silver acetate as the sole oxidant (entry 2). Since when using weakly acidic HFIP as the sole solvent some substrate might decompose, DCE was added as the co-solvent, resulting in an increased yield of 73% (entry 3). To optimize the solvent system, we decreased the volume of HFIP to 15% and found that the combined yield was only slightly improved (entry 4). However, a further decreased volume of HFIP led to a much diminished yield (entry 5). Other solvents were also screened, but the combination of DCE and HFIP proved to be the best. The addition of a weak base, such as KHCO_3_, to tune the acidity of the reaction system was not effective either (entry 6). Since a higher catalytic turnover of the Pd catalyst was observed with 50% volume of HFIP, we repeated the reaction with this solvent system at 80 °C and found that the combined yield was improved to 90% in 32 hours under nitrogen (entry 7), representing the highest catalytic turnover of the Pd catalyst. Finally, by adding 5 equivalents of DMF we were able to get more mono-olefinated product in 28 hours while maintaining the overall efficiency (entry 8, see the ESI† for more condition screenings). However, further screening of reaction conditions could not result in better mono- *vs.* di-olefination selectivity at present, and a study on this issue is actively being carried out in our laboratory. The *meta*-selectivity was unambiguously verified by X-ray crystallographic analysis of a derivative obtained by hydrolyzing the ester group of **4a_mono_** (see the ESI†).

**Table 2:** Screening of reaction conditions for *meta*-C–H olefination^*a*^

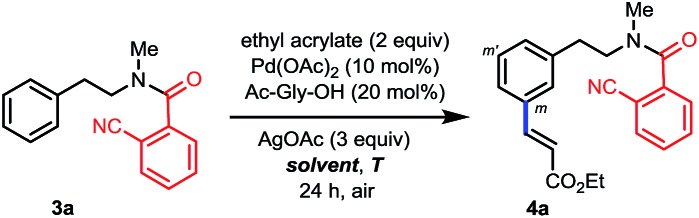				
Entry	Solvents [v/v]	*T* (°C)	Yield (%) [**4a_mono_**, **4a_(m,m′)di_**]	**3a** (%)
1^*b*^	*t*-Amyl-OH	90	10 [10, 0]	90
2	HFIP	90	58 [13, 45]	Trace
3	DCE/HFIP [50/50]	90	71 [32, 39]	Trace
4	DCE/HFIP [85/15]	90	73 [48, 25]	10
5	DCE/HFIP [95/5]	90	39 [32, 7]	44
6^*c*^	DCE/HFIP [85/15]	90	26 [26, 0]	55
**7** ^*d*^ ^,^ ^*e*^	**DCE/HFIP [50/50]**	**80**	**90 [46, 44]**	**Trace**
8^*d*^ ^,^^*f*^	DCE/HFIP [50/50]	80	90 [58, 32]	Trace

^*a*^Reaction conditions: **3a** (0.2 mmol), ethyl acrylate (0.4 mmol), Pd(OAc)_2_ (10 mol%), Ac-Gly-OH (20 mol%), AgOAc (0.6 mmol), solvent (2 mL), 24 h, 80–90 °C. Yield was determined by ^1^H NMR analysis using CH_2_Br_2_ as the internal standard.

^*b*^Using the same conditions as in Scheme 2.

^*c*^KHCO_3_ (2 equiv.) was added.

^*d*^Under N_2_.

^*e*^32 h. Isolated yields were 45% of **4a_mono_** and 37% of **4a_(m,m′)di_**.

^*f*^28 h, DMF (5 equiv.) was added.

With the optimized conditions at hand, we examined the scope of this remote *meta*-selective olefination protocol (Table 3). *Ortho*-substituted substrates with both electron-donating methyl and methoxy and electron-withdrawing bromo and chloro groups proved to be suitable substrates, producing good combined yields of *meta*-olefinated products (**4b–4e**). It is worth noting that arenes bearing bromo or chloro substituents (**4d** and **4e**) were compatible substrates, enabling further elaboration at the halogenated positions. Moreover, although we could not circumvent di-olefination (**4b**, **4d–e**), the fact that both *meta*-positions of 2-substituted substrates could be functionalized provides a great opportunity for synthesis of diversely substituted arenes, which is particularly beneficial for the drug discovery industry. The remaining *meta*-position of *meta*-substituted substrates was also selectively olefinated in high yields (**4f–4i**). *Para*-substituted compounds carrying methoxy or halide groups were also viable substrates for obtaining high yields of the desired products (**4j–4l**). Notably, despite the steric hindrance, the olefin partner could also be installed selectively at the *meta*-position with poly-substituted substrates (**4m–4n**), displaying an uncommon procedure for constructing new penta-substituted phenylethylamines. It is interesting to note that the reaction was not sensitive to the difference of steric hindrance and both *meta*-positions of **3n** could be olefinated. Finally, substituents at the benzylic position were also tolerated (**4o** and **4p**), presenting the potential utility of this protocol with a drug molecule (**4p**). The *meta*-selectivity of various substrates was generally excellent with only minor amounts of other isomers whose amounts were hard to determine due to the presence of rotamers in the ^1^H NMR spectra of the crude olefinated products. The exceptional substrate is **3g**, which also generated around 10% of other isomeric products owing to the electron-donating methoxy substituent. However, it is notable that the intrinsic electronic biases of the molecules were overall successfully overridden (**4d–4f**, **4k–4n**). Moreover, removal of the directing group was smoothly realized by hydrolysis with HCl to afford high yields of new *meta*-substituted phenylethylamines (see the ESI†).

**Table 3:** *meta*-Olefination of phenylethylamine derivatives^*a*^

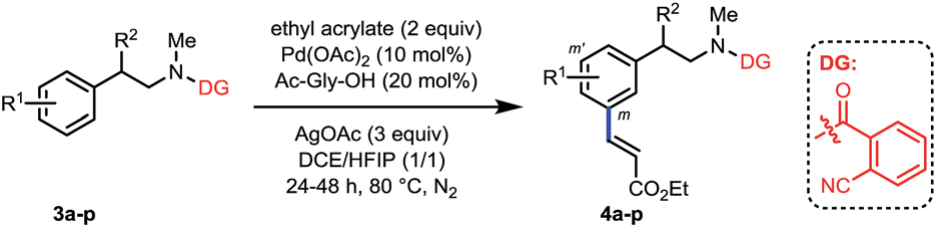
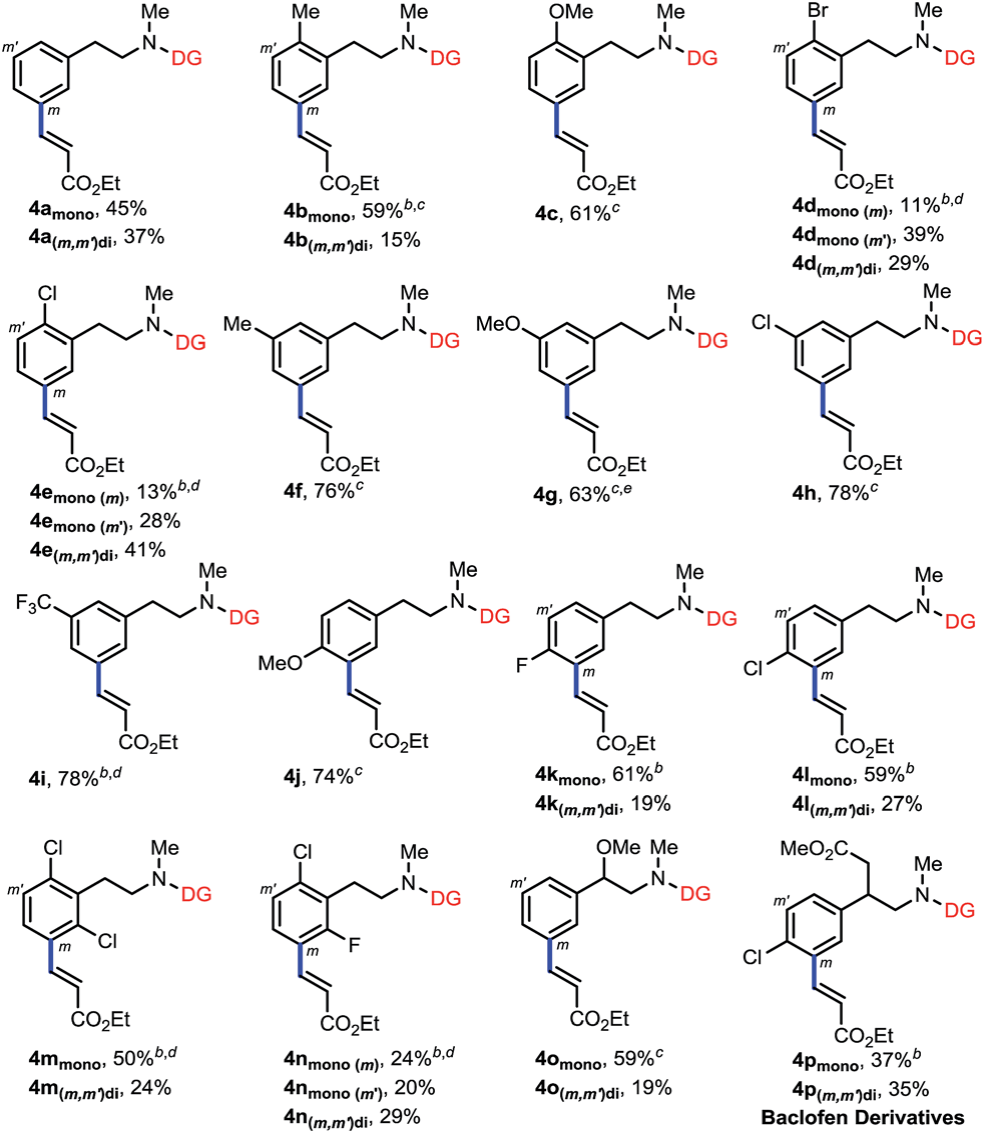

^*a*^Reaction conditions: **3** (0.2 mmol), ethyl acrylate (0.4 mmol), Pd(OAc)_2_ (10 mol%), Ac-Gly-OH (20 mol%), AgOAc (0.6 mmol), DCE (1 mL), HFIP (1 mL), 24–48 h, 80 °C, N_2_. Isolated yields are reported, see the ESI† for details.

^*b*^90 °C.

^*c*^DMF (1 mmol) was added.

^*d*^DCE (0 mL)/HFIP (2 mL).

^*e*^Around 10% of other isomers detected by ^1^H NMR.

To further expand the scope of this reaction, we examined various olefin coupling partners and found olefination of **3f** with α,β-unsaturated ketone, amide and phosphonate afforded desired products in good yields (Table 4, **6a–6c**). We were also pleased to find *trans*-2-butenoate reacted stereoselectively with **3f** to give **6d** in moderate yield. It is noteworthy that this reaction was also compatible with cyclic tri-substituted olefin to give high yield of allylated product (**6e**). Finally, electron deficient styrene such as pentafluorostyrene **5f** was also effective with this method to produce excellent yield of product (**6f**), albeit electron-rich styrenes were not applicable coupling partners.

**Table 4:** Scope of olefin coupling partners^*a*^

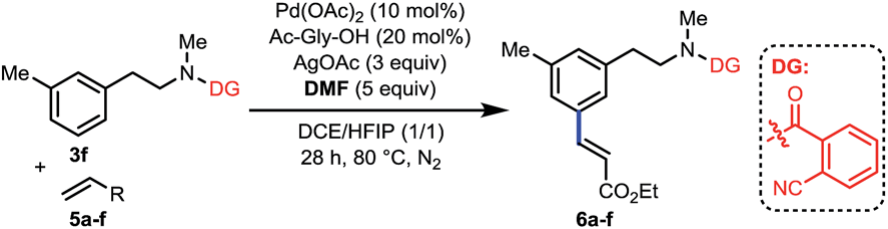
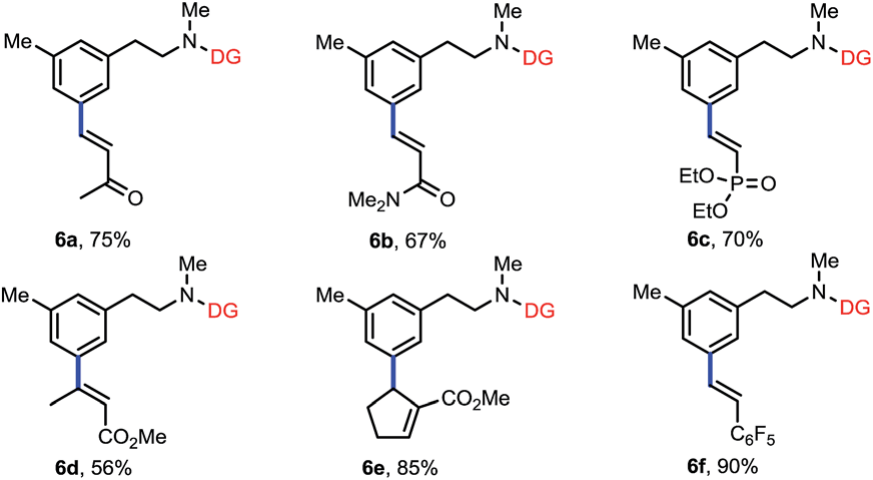

^*a*^Reaction conditions: **3f** (0.1 mmol), **5** (0.2 mmol), Pd(OAc)_2_ (10 mol%), Ac-Gly-OH (20 mol%), AgOAc (0.3 mmol), DMF (0.5 mmol), DCE (0.5 mL), HFIP (0.5 mL), 28 h, 80 °C, N_2_. Isolated yields are reported.

**Scheme 3:**
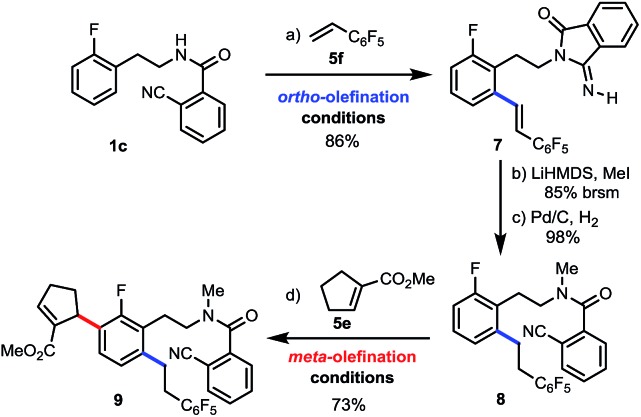
Sequential *ortho*- and *meta*-C–H functionalizations. (a) **5f** (2 equiv.), Pd(OAc)_2_ (10 mol%), Ac-Gly-OH (20 mol%), HFIP (3 equiv.), Ag_2_CO_3_ (30 mol%), O_2_ (1 atm), *t*-amyl-OH, 24 h, 90 °C, 86% yield; (b) LiHMDS (2.5 equiv.), MeI (3 equiv.), THF, –15 °C, 58% yield (85% yield based on recovered starting material [brsm]); (c) Pd/C (12 mol%), H_2_ (1 atm), MeOH, 98%; (d) **5e** (2 equiv.), Pd(OAc)_2_ (10 mol%), Ac-Gly-OH (20 mol%), AgOAc (3 equiv.), DCE (1 mL)/HFIP (1 mL), 48 h, 90 °C, N_2_, 73%.

Finally, to demonstrate the potential of our method for streamlined synthesis of highly substituted arenes, we first subjected **1c** to our standard *ortho*-olefination conditions with **5f** and obtained *ortho*-olefinated **7** in 86% yield (Scheme 3). Then, much to our delight, we were able to convert **7** to the desired amide **8** with the required nitrile group which was reconstructed simultaneously with methylation by using LiHMDS, followed by hydrogenation of the double bond.^20^ Lastly, the *meta*-selective allylation proceeded efficiently with tri-substituted olefin **5e** to afford tetrasubstituted arene **9** in good yield, enabling the building of complexity in a concise manner.

## Conclusions

In summary, a novel example of remote regiodivergent *ortho*- and *meta*-C–H functionalizations has been developed with phenylethylamine derivatives by introducing a novel 2-cyanobenzoyl group as the original directing functionality. A single methylation was sufficient to switch the remote regioselectivity. This method also enabled the novel sequential functionalizations of *ortho*- and *meta*-C–H bonds of a phenylethylamine derivative. Further development of this strategy will improve C–H functionalization to become a more versatile synthetic tool.

## Supplementary Material

Supplementary information

Crystal structure data
