# Supplementary material for: Pd(ii)-catalyzed remote regiodivergent ortho- and meta-C–H functionalizations of phenylethylamines
Source: Chem Sci. 2015 Jun 25;6(10):5595–600. doi: 10.1039/c5sc01737h (PMC5949857; doi:10.1039/c5sc01737h)
Supplement: Supplementary file 1 [file SC-006-C5SC01737H-s001.pdf]

Electronic Supplementary Material (ESI) for Chemical Science.
This journal is © The Royal Society of Chemistry 2015

Electronic Supplementary Information

Pd(II)-Catalyzed Remote Regiodivergent *ortho*- and *meta*-C–H Functionalizations of Phenylethylamines†

Shangda Li,‡[a] Huafang Ji,‡[a], Lei Cai[a] and Gang Li*[ab]

[a] *Key Laboratory of Coal to Ethylene Glycol and Its Related Technology, Fujian Institute of Research on the Structure of Matter, Chinese Academy of Sciences, 155 West Yang-Qiao Road, Fuzhou, Fujian, 350002, P. R. China*
[b] *State Key Laboratory of Structural Chemistry, Chinese Academy of Sciences, Fuzhou, Fujian, 350002, P. R. China*

gangli@fjirsm.ac.cn

Table of Contents

1. General Information……S2
2. Experimental Section……S2
2.1 Preparation and characterization of substrates……S2
2.2 Optimization of olefination reaction conditions……S11
2.3 General remote-selective *ortho*-C–H bond olefination procedure and characterization of olefinated compounds……S14
2.4 General remote-selective *meta*-C–H bond olefination procedure and characterization of olefinated compounds……S18
2.5 Sequential remote-selective *ortho*- and *meta*-C–H bond olefination and characterization of the products……S32
2.6 Removal of the directing group and characterization of selected hydrolyzed products……S34
2.7 Hydrolysis of compound **4a$_{mono}$** to **S17**……S36
2.8 Reference……S37
3. NMR Spectra of New Compounds……S38
4. X-Ray Crystallography data for **1a'** (**CCDC 1056709**) and **S17** (**CCDC 1406595**)……S117

1. General Information

Unless otherwise noted, commercial available reagents were purchased from commercial suppliers (such as Strem, J&K Chemical Co., Energy Chemical and Sinopharm,), and used as received. Solvents were generally distilled over CaH_2 or dried over 3 Å molecular seives. THF was distilled over sodium under nitrogen. Hexafluoroisopropanol (HFIP) was distilled before use. Unless otherwise noted, all reactions were run under air and the indicated reaction temperature was that of the oil bath. The reaction vessels used for C–H functionalization were 38 mL sealed tube or 50 mL Schlenk tube (Synthware). Purification of products was performed by flash chromatography (FC) using silica gel or preparative thin layer chromatography. ^{1}H and ^{13}C NMR spectra were recorded on a Bruker AVANCE III spectrometer (400 MHz and 101 MHz, respectively). Chemical shifts are reported parts per million (ppm) referenced to $CDCl_3$ (δ 7.26 ppm) for ^{1}H NMR, $CDCl_3$ (δ 77.16 ppm) for ^{13}C NMR or tetramethylsilane (TMS, δ 0.00 ppm for ^{1}H NMR). The following abbreviations (or combinations thereof) were used to explain multiplicities: s = singlet, d = doublet, t = triplet, q = quartet, hept = heptaplet, m = multiplet, and br = broad. High-resolution mass spectra (HRMS) were obtained on an AB Sciex TripleTOF 5600 LC/MS equipped with an ESI source at the Shanghai Institute of Organic Chemistry (CAS). X-Ray Crystallography data was obtained at Fujian Institute of Research on the Structure of Matter.

2. Experimental Section

2.1 Preparation and characterization of substrates.

***General procedure for preparing substrates of remote-selective ortho-C–H bond olefination*:**

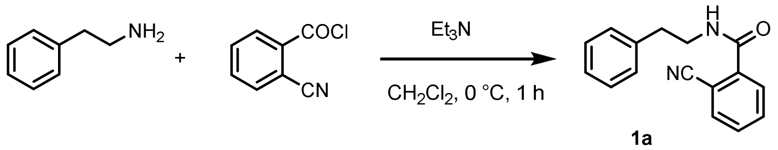

1a

To a solution of 2-cyanobenzoic acid [S1] (441 mg, 3.0 mmol) and DMF (10 μL) in CH_2Cl_2 (20 mL) under N_2 atmosphere was added oxalyl chloride (570 mg, 4.5 mmol) dropwise. The reaction was stirred until the solid suspension disappeared. Then the solvent was removed under reduced pressure. The residue was dissolved in CH_2Cl_2 (10 mL). Then the above solution was added dropwise to a solution of 2-phenylethan-1-amine (363 mg, 3.0 mmol) and Et_3N (0.5 mL, 3.6 mmol) in CH_2Cl_2 (20 mL) at 0 °C. After stirring for 1 h at 0 °C, the reaction mixture was diluted with CH_2Cl_2 (20 mL) and washed with H_2O (20 mL). The organic phase was dried over anhydrous Na_2SO_4 and concentrated under reduced pressure. The residue was purified by flash silica gel chromatography with petroleum ether/EtOAc (2:1) to afford compound 2-cyano-*N*-phenethylbenzamide **1a** (607 mg, 81%).

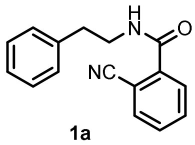

1a **2-Cyano-*N*-phenethylbenzamide**: ^{1}H NMR (400 MHz, $CDCl_3$) δ 7.81-7.67 (m, 2H), 7.63 (t, *J* = 7.6 Hz, 1H), 7.55 (t, *J* = 7.5 Hz, 1H), 7.38-7.30 (m, 2H), 7.30-7.21 (m, 3H), 6.24 (brs, 1H), 3.77 (dd, *J* = 13.1, 6.7 Hz, 2H), 2.99 (t, *J* = 7.0 Hz, 2H); ^{13}C NMR (101 MHz, $CDCl_3$) δ 165.4, 138.8, 138.6, 134.1, 132.9, 131.0, 128.9, 128.8, 128.5, 126.7, 117.6, 110.6, 41.6, 35.4; HRMS (m/z, ESI-TOF): Calcd for $C_{16}H_{15}N_2O^+$ [M+H$^+$] 251.1179, found 251.1180.

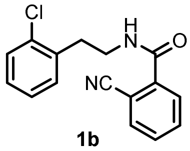

1b ***N*-(2-chlorophenethyl)-2-cyanobenzamide**: ^{1}H NMR (400 MHz, $CDCl_3$) δ 7.74 (t, *J* = 8.2 Hz, 2H), 7.65 (td, *J* = 7.7, 1.1 Hz, 1H), 7.56 (td, *J* = 7.5, 1.2 Hz, 1H), 7.38 (dd, *J* = 7.5, 1.6 Hz, 1H), 7.33 (dd, *J* = 7.2, 1.8 Hz, 1H), 7.25-7.16 (m, 2H), 6.27 (brs, 1H), 3.80 (dd, *J* = 13.0, 6.9 Hz, 2H), 3.14 (t, *J* = 7.0 Hz, 2H); ^{13}C NMR (101 MHz, $CDCl_3$) δ 165.4, 138.7, 136.4, 134.3, 134.2, 133.0, 131.3, 131.1, 129.8, 128.6, 128.4, 127.3, 117.6, 110.7, 40.3, 33.2; HRMS (m/z, ESI-TOF): Calcd for $C_{16}H_{14}ClN_2O^+$ [M+H$^+$] 285.0789, found 285.0790.

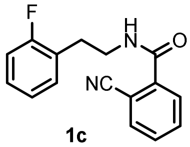

1c **2-Cyano-*N*-(2-fluorophenethyl)benzamide**: ^{1}H NMR (400 MHz, $CDCl_3$) δ 7.75-7.67 (m, 2H), 7.62 (t, *J* = 7.6 Hz, 1H), 7.55 (t, *J* = 7.6 Hz, 1H), 7.30-7.18 (m, 2H), 7.13-6.99 (m, 2H), 6.46 (brs, 1H), 3.74 (q, *J* = 6.6 Hz, 2H), 3.02 (t, *J* = 7.0 Hz, 2H); ^{13}C NMR (101 MHz, $CDCl_3$) δ 165.4, 161.4 (d, J_{C-F} = 245.4 Hz), 138.7, 134.2, 132.9, 131.3 (d, J_{C-F} = 5.1 Hz), 131.0, 128.6 (d, J_{C-F} = 8.1 Hz), 128.5, 125.6 (d, J_{C-F} = 16.2 Hz), 124.4 (d, J_{C-F} = 4.0 Hz), 117.6, 115.5 (d, J_{C-F} = 22.2 Hz), 110.7, 40.6, 29.1; HRMS (m/z, ESI-TOF): Calcd for $C_{16}H_{14}FN_2O^+$ [M+H$^+$] 269.1085, found 269.1085.

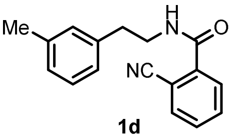

1d **2-Cyano-*N*-(3-methylphenethyl)benzamide**: ^{1}H NMR (400 MHz, $CDCl_3$) δ 7.79-7.66 (m, 2H), 7.62 (t, *J* = 7.5 Hz, 1H), 7.54 (t, *J* = 7.6 Hz, 1H), 7.21 (t, *J* = 7.5 Hz, 1H), 7.15-7.00 (m, 3H), 6.35 (brs, 1H), 3.73 (q, *J* = 6.9 Hz, 2H), 2.93 (t, *J* = 7.1 Hz, 2H), 2.33 (s, 3H); ^{13}C NMR (101 MHz, $CDCl_3$) δ 165.3, 138.8, 138.5, 134.1, 132.9, 131.0, 129.7, 128.7, 128.6, 127.5, 125.9, 117.6, 110.6, 41.7, 35.3, 21.5; HRMS (m/z, ESI-TOF): Calcd for $C_{17}H_{17}N_2O^+$ [M+H$^+$] 265.1335, found 265.1336.

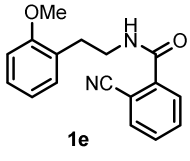

1e **2-Cyano-*N*-(2-methoxyphenethyl)benzamide**: ^{1}H NMR (400 MHz, $CDCl_3$) δ 7.73 (d, *J* = 7.7 Hz, 1H), 7.66 (dd, *J* = 7.7, 1.2 Hz, 1H), 7.61 (td, *J* = 7.6, 1.4 Hz, 1H), 7.54 (td, *J* = 7.5, 1.3 Hz, 1H), 7.26-7.17 (m, 2H), 6.92 (t, *J* = 7.4 Hz, 1H), 6.87 (d, *J* = 8.2 Hz, 1H), 6.54 (brs, 1H), 3.83 (s, 3H), 3.73 (q, *J* = 6.4 Hz, 2H), 2.98 (t, *J* = 6.7 Hz, 2H); ^{13}C NMR (101 MHz, $CDCl_3$)

δ 165.3, 157.6, 139.1, 134.2, 132.8, 130.9, 130.9, 128.5, 128.2, 127.3, 121.0, 117.6, 110.7, 110.7, 55.5, 41.1, 30.1; HRMS (m/z, ESI-TOF): Calcd for $C_{17}H_{17}N_2O_2^+$ [M+H$^+$] 281.1285, found 281.1296.

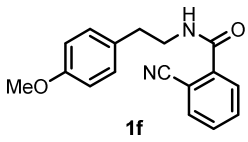

1f **2-Cyano-*N*-(4-methoxyphenethyl)benzamide**: ^{1}H NMR (400 MHz, $CDCl_3$) δ 7.69 (dd, *J* = 7.6, 1.3 Hz, 1H), 7.65 (dd, *J* = 7.8, 1.4 Hz, 1H), 7.59 (td, *J* = 7.6, 1.4 Hz, 1H), 7.52 (td, *J* = 7.6, 1.4 Hz, 1H), 7.15 (d, *J* = 8.5 Hz, 2H), 6.83 (d, *J* = 8.6 Hz, 2H), 6.47 (brs, 1H), 3.76 (s, 3H), 3.67 (q, *J* = 6.9 Hz, 2H), 2.88 (t, *J* = 7.1 Hz, 2H); ^{13}C NMR (101 MHz, $CDCl_3$) δ 165.4, 158.4, 138.8, 134.1, 132.9, 130.9, 130.6, 129.8, 128.4, 117.6, 114.2, 110.6, 55.3, 41.8, 34.5; HRMS (m/z, ESI-TOF): Calcd for $C_{17}H_{17}N_2O_2^+$ [M+H$^+$] 281.1285, found 281.1295.

General procedure for preparing substrates of remote meta-C–H bond olefination:

Method A (for substrates **3a-3n**, except **3e** and **3i**):

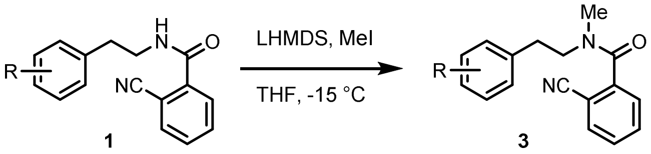

To a cooled solution of amide **1** (2.0 mmol, *directly synthesized as above without purification*) in THF (20 mL) at -15 °C was added LHMDS (1.0 M in THF, 2.4 mL, 2.4 mmol, 1.2 equiv). After stirring at -15 °C for 15 min, MeI (150 μL, 2.4 mmol, 1.2 equiv) was added dropwise to the above solution. The solution was then slowly warmed to room temperature. After 1 h, the reaction was diluted with EtOAc (30 mL) and quenched with water (20 mL). After removing the organic phase, the aqueous phase was extracted again with EtOAc twice (30 mL × 2). The combined organic extract was washed with brine, dried over Na_2SO_4 and concentrated *in vacuo*. The residue was purified by flash silica gel chromatography with petroleum ether/EtOAc to afford corresponding amide **3** in 70%-90% yields.

Method B (for substrates **3e** and **3i**):

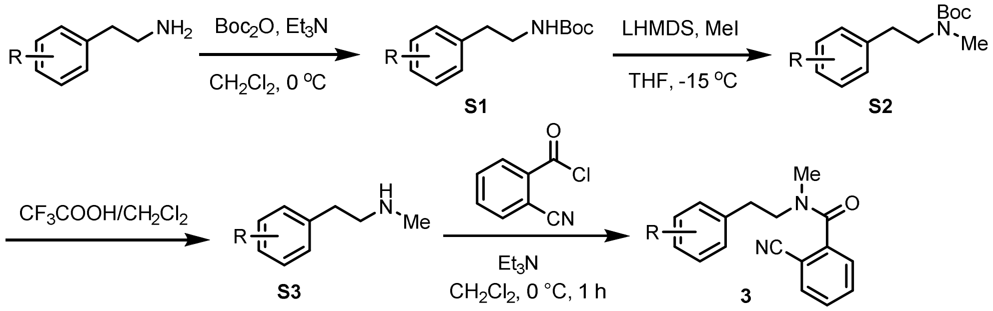

Step 1: To a solution of 2-phenylethan-1-amine (2.0 mmol) and Et_3N (404 mg, 4.0 mmol) in CH_2Cl_2 (7 mL) was added Boc_2O (654 mg, 3.0 mmol, dissolved in 3 mL CH_2Cl_2) at 0 °C. After stirring at 0 °C for 5 min, H_2O (10 mL) was added to quench the reaction. The aqueous phase was extracted with CH_2Cl_2 (10 mL × 2). The combined organic phase was dried over anhydrous Na_2SO_4 and concentrated under reduced pressure. The residue was purified by flash silica gel

chromatography with petroleum ether/EtOAc to afford corresponding carbamate **S1** in quantitative yields.
Step 2: similar procedure as **Method A**.
Step 3: To a solution of the crude product **S2** (without purification) from Step 2 in CH_2Cl_2 (10 mL) was dropwise added CF_3COOH (10 mL) at room temperature. The reaction was then stirred for 10 minutes and the solvent was removed under reduced pressure. To the residue was added NaOH solution (1 N, 10 mL), and the aqueous phase was extracted with CH_2Cl_2 (10 mL × 3). The combined organic phase was dried over anhydrous Na_2SO_4 and concentrated under reduced pressure to yield **S3** in 80-90% yields.
Step 4: similar procedure as the preparation for substrate **1**.

Preparation for substrates **3o**:

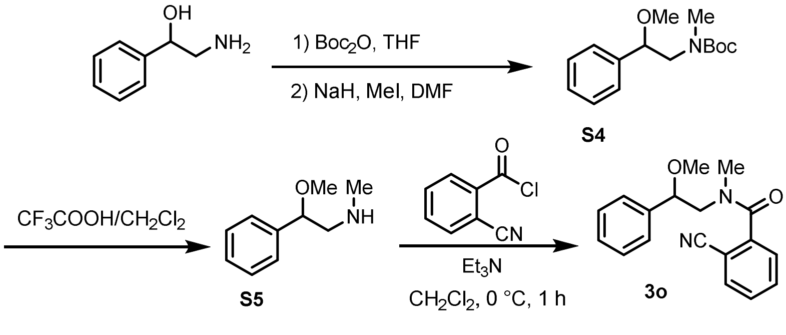

Step 1 and 2: To a solution of 2-amino-1-phenylethan-1-ol (548 mg, 4.0 mmol) in THF (10 mL) at 0 °C was added Boc_2O (959 mg, 4.4 mmol, dissolved in 5.0 mL THF). The mixture was then stirred at 0 °C for 0.5 h. After removal of the solvent, the residue was redissolved in DMF (20 mL) and cooled to 0 °C. To this solution was added NaH (60% in mineral oil, 480 mg, 12 mmol) and the reaction was stirred for 0.5 h at 0 °C. Then MeI (1.7 g, 12 mmol) was added dropwise to the reaction and the solution was stirred for another 2 h while gradually warmed to room temperature. The reaction was then quenched with water (40 mL). The aqueous phase was extracted with EtOAc (20 mL × 3). The combined organic phase was dried over anhydrous Na_2SO_4 and concentrated under reduced pressure. The residue was purified by flash silica gel chromatography with petroleum ether/EtOAc (10:1) to afford product **S4** (890 mg, 84% for two steps) as a colorless oil. **S4**: ^1H NMR (400 MHz, $CDCl_3$) δ 7.41-7.26 (m, 5H), 4.52-4.26 (m, 1H), 3.49-3.37 (m, 1H), 3.37-3.28 (m, 1H), 3.24 (s, 3H), 2.96-2.68 (m, 3H), 1.51-1.34 (m, 9H); ^{13}C NMR (101 MHz, $CDCl_3$) δ 156.0, 155.7, 140.0, 128.6, 128.5, 128.0, 127.9, 126.8, 83.3, 82.6, 79.5, 57.2, 55.9, 36.7, 36.1, 28.5.

Step 3: To a solution of **S4** in CH_2Cl_2 (10 mL) was dropwise added CF_3COOH (10 mL) at room temperature. The reaction was then stirred for 30 minutes and the solvent was removed under reduced pressure. To the residue was added H_2O (20 mL) and NaOH solution (1 N, 10 mL), and the aqueous phase was extracted with CH_2Cl_2 (20 mL × 3). The combined organic phase was dried over anhydrous Na_2SO_4 and concentrated under reduced pressure to yield **S5** (488 mg, 88%). **S5**: ^1H NMR (400 MHz, $CDCl_3$) δ 7.40-7.29 (m, 5H), 4.40 (dd, J = 9.1, 3.5 Hz, 1H), 3.27 (s, 3H), 2.92-2.71 (m, 3H), 2.51 (s, 3H); ^{13}C NMR (101 MHz, $CDCl_3$) δ 139.9, 128.7, 128.2, 126.8, 82.4, 58.3, 57.0, 36.0.

Step 4: **3o** was then prepared with the same procedure as the preparation for substrate **1** in **Section 2.1**.

Preparation for substrate **3p**:

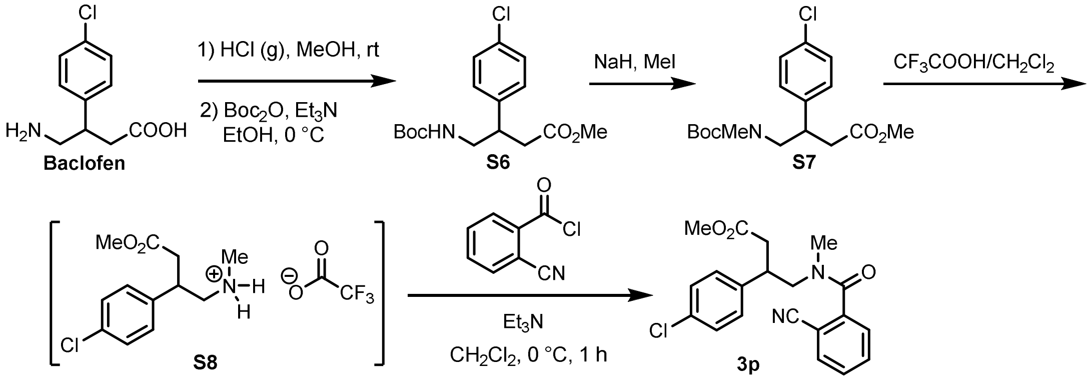

Step 1: To a suspension of baclofen [(*RS*)-4-amino-3-(4-chlorophenyl)butanoic acid] (500 mg, 2.5 mmol) in MeOH (10 mL) was bubbled with HCl gas (generated by adding concentrated H_2SO_4 dropwise to NaCl solid) until a clear solution was observed. Then HCl was bubbled for another 10 min, and the solution was evaporated to give methyl (*RS*)-4-amino-3-(4-chlorophenyl)-butanoate·HCl (590 mg) as colorless crystal. Then the salt (590 mg, 2.2 mmol) was directly dissolved in EtOH (30 mL) without further purification. To this solution was added a solution of Et_3N (1.0 mL, 7.5 mmol) and Boc_2O (1.3 g, 6.2 mmol) in EtOH (8 mL) at 0 °C. The reaction was stirred at 0 °C for 2 h. After evaporation of EtOH, the residue was dissolved in a mixture of H_2O (20 mL) and EtOAc (20 mL). The organic phase was separated and the aqueous phase was extracted with EtOAc (20 mL × 2). The combined organic phase was dried over anhydrous Na_2SO_4 and concentrated under reduced pressure to yield **S6** (688 mg, 84%). **S6** is a known compound,[S2] a sample of **S6** was purified by silica gel chromatography with petroleum ether/EtOAc (10:1). **S6**: ^{1}H NMR (400 MHz, $CDCl_3$) δ 7.29 (d, *J* = 8.4 Hz, 2H), 7.15 (d, *J* = 8.1 Hz, 2H), 4.59 (brs, 1H), 3.59 (s, 3H), 3.49-3.36 (m, 1H), 3.36-3.20 (m, 2H), 2.70 (dd, *J* = 15.7, 6.1 Hz, 1H), 2.58 (dd, *J* = 15.7, 8.1 Hz, 1H), 1.40 (s, 9H); ^{13}C NMR (101 MHz, $CDCl_3$) δ 172.3, 155.9, 139.8, 132.9, 129.0, 128.9, 79.5, 51.8, 45.6, 41.9, 38.1, 28.4.

Step 2: To a solution of **S6** (656 mg, 2.0 mmol, directly synthesized as above without further purification) in DMF (8 mL) at 0 °C was added NaH (60% in mineral oil, 120 mg, 3 mmol, 1.5 equiv). After stirring at 0 °C for 0.5 h, MeI (250 μL, 4 mmol, 2 equiv) was added dropwise. The solution was stirred for another 2 h while gradually warmed to room temperature. Then the reaction was diluted with EtOAc (30 mL) and quenched with water (20 mL). After removing the organic phase, the aqueous phase was extracted again with EtOAc twice (30 mL × 2). The combined organic extract was washed with brine, dried over Na_2SO_4 and concentrated *in vacuo*. The residue was purified by silica gel chromatography with petroleum ether/EtOAc (10:1) to afford **S7** (570 mg, 83%). **S7** (some peaks are broad due to existence of rotamers): ^{1}H NMR (400 MHz, $CDCl_3$) δ 7.27 (d, *J* = 7.1 Hz, 2H), 7.21-7.08 (brm, 2H), 3.67-3.38 (brm, 5H), 3.37-3.20 (m, 1H), 2.83-2.64 (m, 4H), 2.59 (dd, *J* = 15.8, 8.2 Hz, 1H), 1.40 (s, 9H).

Step 3 and 4: **S7** (115 mg, 0.34 mmol) was dissolved in CH_2Cl_2 (5 mL) and cooled to 0 °C. CF_3COOH (5 mL) was added dropwise and the solution was stirred for another 30 min at 0 °C. Then the solvent was removed under reduced pressure to yield **S8**. [Note: attempt to neutralize and purify **S8** without forming a CF_3COOH salt failed since a corresponding lactam would be gradually formed from attacking of the methyl ester by the free methyl amine.] Without further purification, the residue was dissolved in CH_2Cl_2 (5 mL) under N_2 atmosphere and cooled to 0 °C. 2-Cyanobenzoyl chloride (prepared using the method described in **Section 2.1** using 2-cyanobenzoic acid and oxalyl chloride, 0.4 mmol, dissolved in 5 mL CH_2Cl_2) was added,

followed by the addition of Et_3N (140 μL, 1 mmol) dropwise. After stirring for 1 h at 0 °C, the reaction mixture was diluted with CH_2Cl_2 (20 mL) and washed with H_2O (20 mL). The organic phase was dried over anhydrous Na_2SO_4 and concentrated under reduced pressure. The residue was purified by silica gel chromatography with petroleum ether/EtOAc (2:1) to afford **3p** (103 mg, 81%).

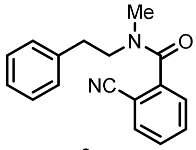

3a **2-Cyano-*N*-methyl-*N*-phenethylbenzamide**: The two rotamers' ratio is about 1:1. ^{1}H NMR (400 MHz, $CDCl_3$) δ 7.71 (d, *J* = 7.8 Hz, 0.50H), 7.63 (t, *J* = 7.1 Hz, 0.98H), 7.54-7.40 (m, 1.55H), 7.40-7.29 (m, 2.51H), 7.28-7.20 (m, 1.95H), 7.01-6.87 (m, 0.98H), 6.75 (dd, *J* = 6.4, 2.4 Hz, 0.49H), 3.82 (t, *J* = 7.6 Hz, 1.00H), 3.44 (t, *J* = 7.0 Hz, 1.00H), 3.21 (s, 1.52H), 3.05 (dd, *J* = 8.6, 6.7 Hz, 1.02H), 2.87-2.76 (m, 2.56H); ^{13}C NMR (101 MHz, $CDCl_3$) δ 168.0, 167.6, 140.8, 140.4, 138.8, 137.7, 133.1, 133.0, 132.9, 132.6, 129.5, 129.2, 129.0, 128.9, 128.9, 128.6, 127.6, 127.4, 126.9, 126.6, 116.9, 116.8, 110.0, 109.8, 52.6, 49.5, 37.4, 34.5, 33.4, 33.0; HRMS (m/z, ESI-TOF): Calcd for $C_{17}H_{17}N_2O^+$ [M+H$^+$] 265.1335, found 265.1336.

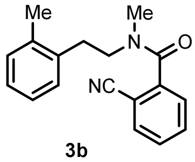

3b **2-Cyano-*N*-methyl-*N*-(2-methylphenethyl)benzamide**: The two rotamers' ratio is about 1:1. ^{1}H NMR (400 MHz, $CDCl_3$) δ 7.75-7.58 (m, 1.44H), 7.55-7.35 (m, 2.00H), 7.30-6.99 (m, 3.53H), 6.91 (d, *J* = 7.1 Hz, 0.51H), 6.85-6.78 (m, 0.50H), 3.80-3.69 (m, 0.93H), 3.38 (t, *J* = 7.2 Hz, 1.07H), 3.23 (s, 1.58H), 3.10-3.01 (m, 0.93H), 2.87 (s, 1.39H), 2.81 (t, *J* = 7.2 Hz, 1.07H), 2.43 (s, 1.38H), 1.93 (s, 1.55H); ^{13}C NMR (101 MHz, $CDCl_3$) δ 167.9, 167.5, 140.7, 140.4, 136.8, 136.5, 136.1, 135.6, 133.1, 132.9, 132.6, 130.6, 130.5, 129.8, 129.6, 129.5, 129.2, 127.6, 127.4, 127.1, 126.8, 126.4, 126.2, 116.9, 116.8, 110.0, 109.8, 51.2, 48.4, 37.2, 33.1, 32.0, 30.8, 19.3, 18.8; HRMS (m/z, ESI-TOF): Calcd for $C_{18}H_{19}N_2O^+$ [M+H$^+$] 279.1492, found 279.1497.

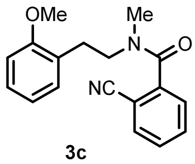

3c **2-Cyano-*N*-(2-methoxyphenethyl)-*N*-methylbenzamide**: The two rotamers' ratio is about 2:1. ^{1}H NMR (400 MHz, $CDCl_3$) δ 7.69 (d, *J* = 7.8 Hz, 0.32H), 7.66-7.58 (m, 0.96H), 7.50-7.39 (m, 1.63H), 7.35 (d, *J* = 7.7 Hz, 0.31H), 7.29-7.17 (m, 1.46H), 6.97-6.77 (m, 2.60H), 6.72 (d, *J* = 8.2 Hz, 0.64H), 3.86 (s, 0.97H), 3.84-3.77 (m, 0.69H), 3.54 (s, 1.97H), 3.43 (t, *J* = 6.9 Hz, 1.31H), 3.21 (s, 1.94H), 3.09-3.01 (m, 0.66H), 2.86-2.76 (m, 2.29H); ^{13}C NMR (101 MHz, $CDCl_3$) δ 167.8, 167.3, 157.6, 157.4, 140.8, 140.4, 132.9, 132.8, 132.6, 132.3, 130.7, 130.6, 129.2, 128.8, 128.2, 127.8, 127.6, 127.2, 127.0, 125.7, 120.5, 120.5, 116.8, 110.2, 110.2, 109.8, 109.7, 55.2, 54.8, 50.6, 47.5, 37.1, 32.8, 29.4, 28.2; HRMS (m/z, ESI-TOF): Calcd for $C_{18}H_{19}N_2O_2^+$ [M+H$^+$] 295.1441, found 295.1442.

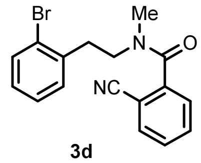

3d **N-(2-bromophenethyl)-2-Cyano-N-methylbenzamide**: The two rotamers' ratio is about 1:1. ^{1}H NMR (400 MHz, $CDCl_3$) δ 7.70 (d, J = 7.7 Hz, 0.48H), 7.68-7.61 (m, 0.97H), 7.58-7.37 (m, 3.47H), 7.28 (t, J = 7.5 Hz, 0.54H), 7.22 (t, J = 7.4 Hz, 0.57H), 7.10 (t, J = 7.6 Hz, 1.02H), 7.05 (d, J = 7.5 Hz, 0.52H), 6.86 (d, J = 7.4 Hz, 0.53H), 3.88-3.76 (m, 0.92H), 3.49 (t, J = 7.1 Hz, 1.06H), 3.23 (s, 1.58H), 3.19 (t, J = 7.6 Hz, 0.94H), 2.93 (t, J = 7.1 Hz, 1.04H), 2.85 (s, 1.36H); ^{13}C NMR (101 MHz, $CDCl_3$) δ 167.8, 167.4, 140.3, 139.9, 137.9, 136.6, 132.9, 132.8, 132.8, 132.7, 132.5, 131.2, 131.0, 129.3, 129.2, 128.6, 128.3, 127.7, 127.6, 127.3, 127.1, 124.3, 124.2, 116.7, 116.7, 109.6, 109.6, 50.2, 47.5, 37.2, 34.6, 33.4, 32.9; HRMS (m/z, ESI-TOF): Calcd for $C_{17}H_{16}BrN_2O^+$ [M+H$^+$] 343.0441, found 343.0448.

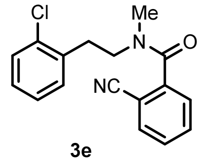

3e **N-(2-chlorophenethyl)-2-cyano-N-methylbenzamide**: The two rotamers' ratio is about 1:1. ^{1}H NMR (400 MHz, $CDCl_3$) δ 7.61 (d, J = 7.7 Hz, 0.47H), 7.59-7.51 (m, 0.96H), 7.45-7.24 (m, 2.89H), 7.18-7.05 (m, 2.51H), 6.97 (dd, J = 6.2, 2.8 Hz, 0.51H), 6.80 (d, J = 7.3 Hz, 0.50H), 3.73 (dd, J = 8.5, 6.5 Hz, 0.92H), 3.39 (t, J = 7.1 Hz, 1.06H), 3.17-3.06 (m, 2.49H), 2.85 (t, J = 7.0 Hz, 1.08H), 2.75 (s, 1.40H); ^{13}C NMR (101 MHz, $CDCl_3$) δ 168.0, 167.6, 140.6, 140.2, 136.4, 135.1, 134.1, 134.0, 133.1, 133.0, 132.9, 132.6, 131.5, 131.2, 129.7, 129.6, 129.5, 129.3, 128.5, 128.2, 127.5, 127.3, 127.3, 127.2, 116.8, 116.8, 109.9, 109.8, 50.4, 47.7, 37.3, 33.1, 32.6, 31.2; HRMS (m/z, ESI-TOF): Calcd for $C_{17}H_{16}ClN_2O^+$ [M+H$^+$] 299.0946, found 299.0957.

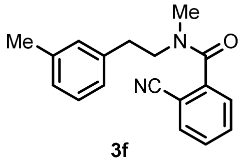

3f **2-Cyano-N-methyl-N-(3-methylphenethyl)benzamide**: The two rotamers' ratio is about 45:55. ^{1}H NMR (400 MHz, $CDCl_3$) δ 7.70 (d, J = 7.8 Hz, 0.45H), 7.67-7.59 (m, 0.93H), 7.54-7.40 (m, 1.46H), 7.37 (d, J = 7.7 Hz, 0.45H), 7.21 (t, J = 7.5 Hz, 0.48H), 7.17-7.07 (m, 1.42H), 7.04 (d, J = 7.3 Hz, 0.99H), 6.82-6.69 (m, 1.46H), 3.79 (dd, J = 8.4, 7.0 Hz, 0.93H), 3.42 (t, J = 6.9 Hz, 1.07H), 3.20 (s, 1.50H), 3.00 (dd, J = 8.8, 6.6 Hz, 0.95H), 2.82 (s, 1.42H), 2.77 (t, J = 7.0 Hz, 1.10H), 2.34 (s, 1.51H), 2.26 (s, 1.46H); ^{13}C NMR (101 MHz, $CDCl_3$) δ 168.0, 167.5, 140.8, 140.4, 138.7, 138.4, 138.2, 137.6, 133.1, 133.0, 132.8, 132.6, 129.8, 129.6, 129.5, 129.2, 128.7, 128.5, 127.7, 127.6, 127.4, 127.3, 126.0, 125.9, 116.9, 116.9, 110.0, 109.8, 52.7, 49.6, 37.4, 34.4, 33.3, 32.9, 21.43, 21.36; HRMS (m/z, ESI-TOF): Calcd for $C_{18}H_{19}N_2O^+$ [M+H$^+$] 279.1492, found 279.1492.

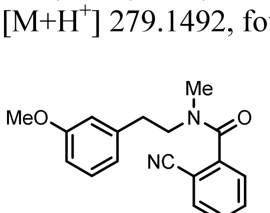

3g **2-Cyano-N-(3-methoxyphenethyl)-N-methylbenzamide**: The two rotamers' ratio is about 1:1. ^{1}H NMR (400 MHz, $CDCl_3$) δ 7.65 (d, J = 7.8 Hz, 0.51H), 7.62-7.53 (m, 0.99H), 7.49-7.36 (m, 1.50H), 7.33 (d, J = 7.7 Hz, 0.50H), 7.20 (t, J = 7.9 Hz, 0.51H), 7.12 (t, J = 7.9 Hz, 0.49H), 6.87 (d, J = 7.6 Hz, 0.50H), 6.83 (s, 0.50H), 6.79 (d, J = 7.4 Hz, 0.49H), 6.74

(dt, J = 8.0, 3.0 Hz, 0.97H), 6.51 (d, J = 7.5 Hz, 0.49H), 6.41 (s, 0.47H), 3.81-3.73 (m, 2.54H), 3.68 (s, 1.48H), 3.39 (t, J = 6.9 Hz, 0.97H), 3.14 (s, 1.48H), 2.98 (t, J = 7.6 Hz, 1.03H), 2.77 (s, 1.49H), 2.74 (t, J = 6.9 Hz, 0.98H); ^{13}C NMR (101 MHz, CDCl$_3$) δ 167.8, 167.4, 159.8, 159.7, 140.5, 140.3, 140.2, 139.0, 133.0, 132.8, 132.7, 132.5, 129.7, 129.5, 129.4, 129.1, 127.5, 127.1, 121.1, 121.0, 116.8, 116.7, 114.3, 112.1, 112.0, 109.7, 109.5, 55.1, 55.0, 52.4, 49.2, 37.2, 34.3, 33.3, 32.7; HRMS (m/z, ESI-TOF): Calcd for $C_{18}H_{19}N_2O_2^+$ [M+H$^+$] 295.1441, found 295.1447.

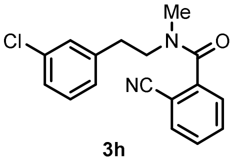

3h **N-(3-chlorophenethyl)-2-cyano-N-methylbenzamide**: The two rotamers' ratio is about 3:2. ^{1}H NMR (400 MHz, CDCl$_3$) δ 7.71 (d, J = 7.7 Hz, 0.57H), 7.65 (t, J = 7.9 Hz, 0.97H), 7.56-7.44 (m, 1.51H), 7.39 (d, J = 7.7 Hz, 0.58H), 7.34-7.16 (m, 3.39H), 6.91-6.79 (m, 1.26H), 3.80 (t, J = 7.5 Hz, 1.14H), 3.45 (t, J = 6.7 Hz, 0.88H), 3.20 (s, 1.33H), 3.02 (t, J = 7.5 Hz, 1.14H), 2.84 (s, 1.67H), 2.80 (t, J = 6.8 Hz, 0.91H); ^{13}C NMR (101 MHz, CDCl$_3$) δ 167.5, 167.2, 140.5, 140.0, 139.7, 139.4, 134.0, 133.7, 132.8, 132.6, 132.6, 132.3, 129.7, 129.6, 129.2, 129.1, 128.6, 128.4, 127.0, 126.9, 126.8, 126.6, 126.3, 116.5, 116.4, 109.3, 109.2, 51.7, 48.6, 36.9, 33.6, 32.5, 32.4; HRMS (m/z, ESI-TOF): Calcd for $C_{17}H_{16}ClN_2O^+$ [M+H$^+$] 299.0946, found 299.0946.

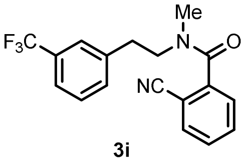

3i **2-Cyano-N-methyl-N-(3-(trifluoromethyl)phenethyl)benzamide**: The two rotamers' ratio is about 3:2. ^{1}H NMR (400 MHz, CDCl$_3$) δ 7.70 (d, J = 7.7 Hz, 0.61H), 7.64 (t, J = 7.5 Hz, 0.98H), 7.58-7.40 (m, 4.25H), 7.37 (t, J = 7.2 Hz, 0.95H), 7.17 (d, J = 7.6 Hz, 0.37H), 7.13 (s, 0.33H), 6.80 (dd, J = 6.6, 2.1 Hz, 0.35H), 3.81 (dd, J = 8.7, 6.5 Hz, 1.14H), 3.47 (t, J = 6.9 Hz, 0.83H), 3.20 (s, 1.12H), 3.10 (t, J = 7.7 Hz, 1.17H), 2.88 (t, J = 7.0 Hz, 0.77H), 2.84 (s, 1.75H); ^{13}C NMR (101 MHz, CDCl$_3$) δ 167.8, 167.6, 140.3, 140.0, 139.7, 138.6, 133.1, 132.8, 132.6, 132.4, 132.3, 130.6 (q, $J_{C\text{-}F}$ = 32.0 Hz), 130.5 (q, $J_{C\text{-}F}$ = 32.1 Hz), 129.5, 129.4, 129.2, 129.0, 127.1, 125.4 (q, $J_{C\text{-}F}$ = 4.0 Hz), 125.2 (q, $J_{C\text{-}F}$ = 3.1 Hz), 124.1 (q, $J_{C\text{-}F}$ = 273.2 Hz), 123.9 (q, $J_{C\text{-}F}$ = 273.6 Hz), 123.59 (q, $J_{C\text{-}F}$ = 3.7 Hz), 123.28 (q, $J_{C\text{-}F}$ = 3.9 Hz), 116.7, 116.7, 109.7, 109.6, 52.1, 49.0, 37.1, 34.1, 32.9, 32.7; HRMS (m/z, ESI-TOF): Calcd for $C_{18}H_{16}F_3N_2O^+$ [M+H$^+$] 333.1209, found 333.1221.

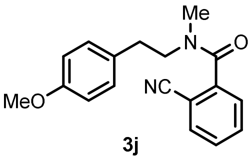

3j **2-Cyano-N-(4-methoxyphenethyl)-N-methylbenzamide**: The two rotamers' ratio is about 1:1. ^{1}H NMR (400 MHz, CDCl$_3$) δ 7.66 (d, J = 7.7 Hz, 0.51H), 7.60 (t, J = 6.9 Hz, 0.96H), 7.50-7.37 (m, 1.48H), 7.34 (d, J = 7.7 Hz, 0.47H), 7.20 (d, J = 8.3 Hz, 0.97H), 6.89-6.71 (m, 2.45H), 6.75 (d, J = 8.5 Hz, 0.96H), 3.82-3.66 (m, 4.00H), 3.36 (t, J = 6.9 Hz, 0.96H), 3.15 (s, 1.46H), 2.95 (t, J = 7.5 Hz, 0.99H), 2.77 (s, 1.45H), 2.72 (t, J = 6.9 Hz, 1.02H); ^{13}C NMR (101 MHz, CDCl$_3$) δ 167.9, 167.4, 158.5, 158.2, 140.7, 140.3, 133.0, 132.9, 132.8, 132.6, 130.7, 129.8, 129.7, 129.6, 129.4, 129.2, 127.5, 127.2, 116.8, 116.8, 114.1, 114.0, 109.8, 109.6, 55.3, 55.2, 52.7, 49.6, 37.3, 33.5, 32.8, 32.4; HRMS (m/z, ESI-TOF): Calcd for $C_{18}H_{19}N_2O_2^+$ [M+H$^+$] 295.1441, found 295.1443.

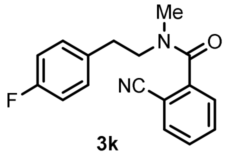

2-Cyano-*N*-(4-fluorophenethyl)-*N*-methylbenzamide: The two rotamers' ratio is about 54:46. ^{1}H NMR (400 MHz, $CDCl_3$) δ 7.70 (d, *J* = 7.7 Hz, 0.54H), 7.64 (t, *J* = 7.9 Hz, 0.98H), 7.56-7.40 (m, 1.46H), 7.37 (d, *J* = 7.7 Hz, 0.54H), 7.28 (t, *J* = 6.8 Hz, 1.21H), 7.01 (t, *J* = 8.5 Hz, 1.14H), 6.95-6.86 (m, 2.02H), 3.78 (t, *J* = 7.6 Hz, 1.12H), 3.42 (t, *J* = 6.9 Hz, 0.85H), 3.19 (s, 1.25H), 3.02 (t, *J* = 7.5 Hz, 1.12H), 2.88-2.74 (m, 2.63H); ^{13}C NMR (101 MHz, $CDCl_3$) δ 168.0, 167.6, 161.9 (d, J_{C-F} = 246 Hz), 161.7 (d, J_{C-F} = 245 Hz), 140.6, 140.3, 134.5 (d, J_{C-F} = 3 Hz), 133.4 (d, J_{C-F} = 3 Hz), 133.1 (d, J_{C-F} = 16 Hz), 132.8 (d, J_{C-F} = 16 Hz), 130.4, 130.4, 130.3, 130.3, 129.5, 129.4, 127.5, 127.3, 116.9, 116.8, 115.7 (d, J_{C-F} = 21 Hz), 115.4 (d, J_{C-F} = 21 Hz), 110.0, 109.8, 52.7, 49.5, 37.4, 33.7, 33.0, 32.6; HRMS (m/z, ESI-TOF): Calcd for $C_{17}H_{16}FN_2O^+$ [M+H$^+$] 283.1241, found 283.1242.

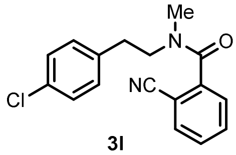

***N*-(4-chlorophenethyl)-2-cyano-*N*-methylbenzamide**: The two rotamers' ratio is about 56:44. ^{1}H NMR (400 MHz, $CDCl_3$) δ 7.70 (d, *J* = 7.8 Hz, 0.56H), 7.64 (t, *J* = 7.2 Hz, 0.97H), 7.55-7.42 (m, 1.41H), 7.37 (d, *J* = 7.7 Hz, 0.57H), 7.33-7.16 (m, 3.19H), 6.88 (t, *J* = 8.4 Hz, 1.19H), 3.86-3.73 (m, 1.16H), 3.42 (t, *J* = 7.0 Hz, 0.83H), 3.18 (s, 1.22H), 3.06-2.97 (m, 1.17H), 2.82 (s, 1.73H), 2.79 (t, *J* = 7.0 Hz, 0.83H); ^{13}C NMR (101 MHz, $CDCl_3$) δ 167.9, 167.6, 140.6, 140.2, 137.3, 136.2, 133.2, 133.0, 133.0, 132.8, 132.7, 132.4, 130.4, 130.2, 129.6, 129.4, 129.0, 128.8, 127.6, 127.3, 116.9, 116.8, 109.9, 109.8, 52.5, 49.4, 37.4, 33.9, 33.0, 32.7; HRMS (m/z, ESI-TOF): Calcd for $C_{17}H_{16}ClN_2O^+$ [M+H$^+$] 299.0946, found 299.0947.

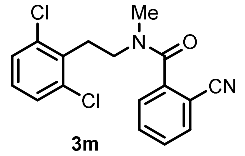

2-Cyano-*N*-(2,6-dichlorophenethyl)-*N*-methylbenzamide: The two rotamers' ratio is about 2:1. ^{1}H NMR (400 MHz, $CDCl_3$) δ 7.74-7.61 (m, 1.28H), 7.57 (t, *J* = 7.6 Hz, 0.63H), 7.54-7.43 (m, 1.32H), 7.32 (d, *J* = 8.0 Hz, 0.65H), 7.28-7.23 (m, 1.23H), 7.20 (d, *J* = 8.0 Hz, 1.20H), 7.15-7.04 (m, 0.99H), 3.83 (t, *J* = 7.3 Hz, 0.68H), 3.48-3.35 (m, 1.96H), 3.29 (s, 1.87H), 3.15 (t, *J* = 7.8 Hz, 1.28H), 2.93 (s, 1.04H); ^{13}C NMR (101 MHz, $CDCl_3$) δ 167.9, 167.6, 140.6, 140.4, 135.9, 135.7, 134.9, 133.3, 133.1, 133.0, 132.9, 132.9, 129.5, 129.4, 128.7, 128.4, 128.4, 127.5, 116.8, 110.2, 110.0, 48.9, 46.1, 37.2, 33.3, 30.2, 28.9; HRMS (m/z, ESI-TOF): Calcd for $C_{17}H_{15}Cl_2N_2O^+$ [M+H$^+$] 333.0556, found 333.0567.

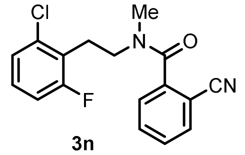

***N*-(2-chloro-6-fluorophenethyl)-2-cyano-*N*-methylbenzamide**: The two rotamers' ratio is about 3:2. ^{1}H NMR (400 MHz, $CDCl_3$) δ 7.74-7.60 (m, 1.36H), 7.56-7.40 (m, 1.94H), 7.23-7.11 (m, 1.53H), 7.10-6.96 (m, 1.61H), 6.92 (t, *J* = 8.7 Hz, 0.61H), 3.83 (t, *J* = 7.1

Hz, 0.75H), 3.44 (t, $J = 7.3$ Hz, 1.19H), 3.30-3.18 (m, 2.56H), 3.01 (t, $J = 7.2$ Hz, 1.19H), 2.89 (s, 1.16H); ^{13}C NMR (101 MHz, $CDCl_3$) δ 167.9, 167.6, 161.8 (d, $J_{C\text{-}F} = 248$ Hz), 161.7 (d, $J_{C\text{-}F} = 249$ Hz), 140.6, 140.2, 135.5 (d, $J_{C\text{-}F} = 6$ Hz), 135.4 (d, $J_{C\text{-}F} = 6$ Hz), 133.1, 132.9, 132.8, 129.5, 129.4, 128.8 (d, $J_{C\text{-}F} = 10$ Hz), 128.5 (d, $J_{C\text{-}F} = 10$ Hz), 127.4 (d, $J_{C\text{-}F} = 4$ Hz), 125.4 (d, $J_{C\text{-}F} = 4$ Hz), 125.3, 124.9 (d, $J_{C\text{-}F} = 19$ Hz), 123.4 (d, $J_{C\text{-}F} = 19$ Hz), 116.8, 114.2 (d, $J_{C\text{-}F} = 23$ Hz), 114.0 (d, $J_{C\text{-}F} = 23$ Hz), 110.0, 109.9, 49.4, 46.6, 37.2, 33.1, 25.3 (d, $J_{C\text{-}F} = 2$ Hz), 24.3 (d, $J_{C\text{-}F} = 2$ Hz); HRMS (m/z, ESI-TOF): Calcd for $C_{17}H_{15}ClFN_2O^+$ [M+H$^+$] 317.0851, found 317.0861.

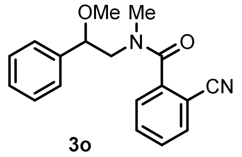

3o **2-Cyano-*N*-(2-methoxy-2-phenylethyl)-*N*-methylbenzamide**: The two rotamers' ratio is about 56:44. ^{1}H NMR (400 MHz, $CDCl_3$) δ 7.72 (d, $J = 7.8$ Hz, 0.56H), 7.70-7.60 (m, 1.00H), 7.59-7.35 (m, 4.39H), 7.36-7.26 (m, 1.91H), 7.18 (d, $J = 7.6$ Hz, 0.44H), 7.09-6.97 (m, 0.88H), 4.72 (dd, $J = 8.7$, 3.9 Hz, 0.56H), 4.29 (dd, $J = 8.3$, 4.2 Hz, 0.44H), 3.95 (dd, $J = 13.7$, 3.9 Hz, 0.56H), 3.55-3.43 (m, 1.03H), 3.38-3.17 (m, 4.86H), 2.93 (s, 1.73H); ^{13}C NMR (101 MHz, $CDCl_3$) δ 168.6, 167.7, 140.6, 140.4, 139.4, 138.4, 132.9, 132.8, 132.6, 132.4, 129.3, 129.1, 128.6, 128.4, 128.2, 127.9, 127.8, 127.0, 126.6, 126.3, 116.8, 116.7, 110.3, 109.7, 82.0, 80.9, 57.2, 56.9, 56.8, 54.6, 38.9, 33.8; HRMS (m/z, ESI-TOF): Calcd for $C_{18}H_{19}N_2O_2^+$ [M+H$^+$] 295.1441, found 295.1447.

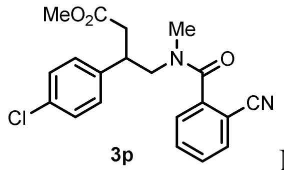

3p **Methyl 3-(4-chlorophenyl)-4-(2-cyano-*N*-methylbenzamido)butanoate**: The two rotamers' ratio is about 2:1. ^{1}H NMR (400 MHz, $CDCl_3$) δ 7.69 (d, $J = 7.7$ Hz, 0.68H), 7.67-7.56 (m, 1.04H), 7.55-7.42 (m, 1.42H), 7.36-7.28 (m, 2.68H), 7.22 (d, $J = 8.3$ Hz, 0.68H), 7.16 (d, $J = 7.6$ Hz, 0.69H), 6.88 (d, $J = 8.3$ Hz, 0.65H), 6.83-6.74 (m, 0.29H), 3.99 (dd, $J = 16.6$, 10.5 Hz, 0.71H), 3.73-3.63 (m, 1.47H), 3.63-3.56 (m, 3.00H), 3.56-3.30 (m, 1.09H), 3.14 (s, 0.94H), 2.92-2.82 (m, 0.75H), 2.78-2.63 (m, 2.80H), 2.59-2.42 (m, 0.69H); ^{13}C NMR (101 MHz, $CDCl_3$) δ 172.1, 171.4, 168.1, 168.0, 140.4, 139.7, 139.6, 138.7, 133.5, 133.1, 133.1, 133.0, 132.6, 129.6, 129.5, 129.3, 129.2, 128.9, 128.2, 127.2, 116.9, 116.7, 109.9, 109.9, 56.5, 52.3, 52.0, 51.8, 39.9, 39.6, 38.3, 37.4, 33.6; HRMS (m/z, ESI-TOF): Calcd for $C_{20}H_{20}ClN_2O_3^+$ [M+H$^+$] 371.1157, found 371.1168.

2.2 Optimization of olefination reaction conditions.

2.2.1 Condition optimization for remote-selective ortho-C–H bond olefination: [a]

General optimization procedure: A 35 mL sealed tube or 50 mL Schlenk sealed tube (with a Teflon cap, Schlenk sealed tube was used when oxygen was the terminal oxidant) equipped with a magnetic stir bar was charged with amide **1a** (0.10 mmol, 1.0 equiv), $Pd(OAc)_2$ (2.3 mg, 0.010 mmol, 10 mol%), Ac-Gly-OH (2.4 mg, 0.020 mmol, 20 mol%), oxidant and solid additive (if additive was used) sequentially. The tube was then purged with oxygen (1 atm, if oxygen was used). *t*-Amyl-OH (0.5 mL) was added to the mixture, followed by ethyl acrylate (2.0 equiv) and HFIP (32 μL, if used). And then another 0.5 mL of *t*-Amyl-OH was added along the inside wall of the tube. The tube was then capped under a flow of O_2 (1 atm, if oxygen was used) and submerged into a preheated 90 °C oil bath. The reaction was stirred for 20–36 h and cooled to

room temperature. The crude reaction mixture was diluted with EtOAc (5 mL) and filtered through a short pad of Celite. The sealed tube and Celite pad were washed with an additional 20 mL of EtOAc. The filtrate was concentrated *in vacuo*, and crude ^{1}H NMR spectrum was taken using CH_2Br_2 as internal standard. (Note: all the chemicals are added as their pure form, for example, not in a solution.)

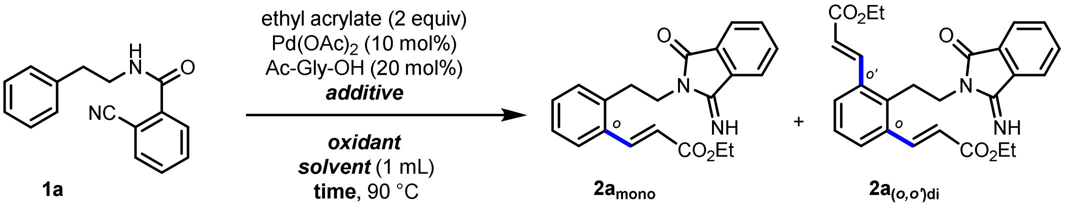

Entry	t (h)	oxidant (equiv)	additive (equiv)	solvent	Yield of $3a_{mono}$ (%)	Yield of $3a_{di(o,o')}$ (%)
1	24	AgOAc (3)	--	*t*-Amyl-OH	32	50
2	24	AgOAc (3)	--	toluene	29	6
**3**	**20**	**Ag_2CO_3 (3)**	**--**	***t*-Amyl-OH**	**40**	**55**
4	20	O_2 (1 atm)	$Cu(OAc)_2$ (0.3)	*t*-Amyl-OH	30	16
5	20	O_2 (1 atm)	HFIP (3)	*t*-Amyl-OH	33	40
6	36	air (1 atm)	HFIP (3)	*t*-Amyl-OH	trace	trace
7	20	O_2 (1 atm)	HFIP (3)	DCE	0	0
8[b]	20	O_2 (1 atm)	HFIP (3)	*t*-Amyl-OH	trace	--
9	36	O_2 (1 atm)	HFIP (3)	*t*-Amyl-OH	33	34
10[c]	20	O_2 (1 atm)	HFIP (3)	*t*-Amyl-OH	trace	--
**11[d]**	**23**	**O_2 (1 atm)**	**HFIP (3)**	***t*-Amyl-OH**	**32**	**57**
12[d]	23	O_2 (1 atm)	--	*t*-Amyl-OH	40	14

[a] Conditions: **1a** (0.1 mmol), ethyl acrylate (0.2 mmol), $Pd(OAc)_2$ (10 mol%), Ac-Gly-OH (20 mol%). Yield was determined by ^{1}H NMR analysis using CH_2Br_2 as internal standard. [b] Ac-Ile-OH (20 mol%) was used instead of Ac-Gly-OH. [c] 10 mol% *p*-benzoquinone was added. [d] 0.2 mmol scale, Ag_2CO_3 (30 mol%) was added and the isolated yields were reported for entry 11.

2.2.2 Condition optimization for remote meta-C–H bond olefination: [a]

General optimization procedure: A 35 mL sealed tube or 50 mL Schlenk sealed tube (with a Teflon cap, Schlenk sealed tube was used when the reaction was run under oxygen/nitrogen) equipped with a magnetic stir bar was charged with amide **3** (0.20 mmol, 1.0 equiv), $Pd(OAc)_2$ (4.6 mg, 0.020 mmol, 10 mol%), Ac-Gly-OH (4.7 mg, 0.040 mmol, 20 mol%) and AgOAc (100 mg, 0.60 mmol, 3 equiv). The tube was then purged with nitrogen (1 atm, if nitrogen was used). DCE (1.0 mL) was added to the mixture, followed by ethyl acrylate (2.0 equiv) [and DMF (77 μL, 1.0 mmol, 5 equiv) when necessary as noted below]. And then 1.0 mL of HFIP was added

along the inside wall of the tube. The tube was then capped under a flow of nitrogen (if nitrogen was used) and submerged into a preheated 90 °C oil bath. The reaction was stirred for 24–48 h and cooled to room temperature. The crude reaction mixture was diluted with EtOAc (5 mL) and filtered through a short pad of Celite. The sealed tube and Celite pad were washed with an additional 20 mL of EtOAc. The filtrate was concentrated *in vacuo*, and crude ^{1}H NMR spectrum was taken using CH_2Br_2 as internal standard. (Note: all the chemicals are added as their pure form, for example, not in a solution.)

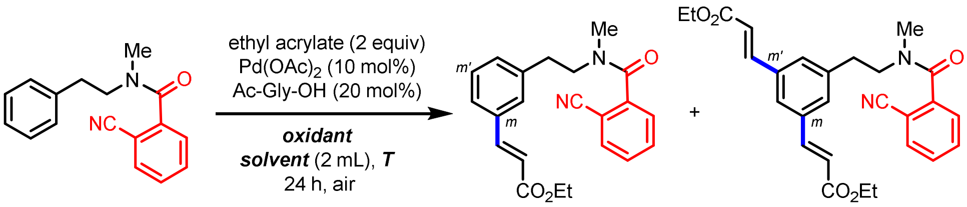

3a **4a**$_{mono}$ **4a**$_{(m,m')di}$

Entry	T (°C)	solvents [v/v]	oxidant (equiv)	additive (equiv)	Yield of **4a**$_{mono}$ (%)	Yield of **4a**$_{(m,m')di}$ (%)	**3a** (%)
1	90	*t*-Amyl-OH	O_2 (1 atm)	Ag_2CO_3 (0.3)	10	--	90
2	90	HFIP	AgOAc (3)	--	13	45	trace
3^b	90	HFIP	AgOAc (3)	--	17	24	trace
4^c	90	HFIP	AgOAc (3)	--	messy mixture		trace
5	90	DCE/HFIP [50/50]	AgOAc (3)	--	32	39	trace
6	90	DCE/HFIP [85/15]	AgOAc (3)	--	48	25	10
7	90	DCE/HFIP [85/15]	AgOAc (3)	$KHCO_3$ (2)	26	0	55
8	90	DCE/HFIP [85/15]	AgOAc (3)	KH_2PO_4 (2)	50	22	15
9	90	DCE/HFIP [85/15]	Ag_2CO_3 (3)	--	40	31	7
10^d	90	DCE/HFIP [85/15]	AgOAc (3)	--	44	27	10
11^d	90	DCE/HFIP [85/15]	AgOAc (3)	DMF (5)	44	42	trace
12	90	DCE/HFIP [95/5]	AgOAc (3)	--	32	7	44
13	90	DCE/HFIP [80/20]	AgOAc (3)	--	42	33	6
14	90	DCE/HFIP [70/30]	AgOAc (3)	--	43	31	trace
15^d	90	DCE/HFIP [50/50]	AgOAc (3)	--	46	37	trace
**16^{d,e}**	**80**	**DCE/HFIP [50/50]**	**AgOAc (3)**	**--**	**46**	**44**	**trace**

17[d,f]	**80**	**DCE/HFIP [50/50]**	**AgOAc (3)**	**DMF (5)**	**58**	**32**	**trace**
18[d]	90	EtOAc/HFIP [50/50]	AgOAc (3)	--	42	18	15
19	90	CF_3CH_2OH	AgOAc (3)	--	29	37	trace
20	90	CF_3CH_2OH/ HFIP [85/15]	AgOAc (3)	--	34	35	trace
21	90	CF_3CH_2OH/ DCE [50/50]	AgOAc (3)	--	44	28	10
22[g]	90	DCE/HFIP [50/50]	Ag_2O (3)	DMF (5)	--	--	95
23[g]	90	DCE/HFIP [50/50]	$Cu(OAc)_2$ (3)	DMF (5)	trace	--	~100
24[g,h]	60	DCE/HFIP [50/50]	AgOAc (2)	--	61	14	25
25[g,h,i]	60	DCE/HFIP [50/50]	AgOAc (2)	--	61	13	25
26[e,g,j]	80	DCE/HFIP [50/50]	AgOAc (3)	--	54	8	32

[a] Conditions: **1a** (0.2 mmol), ethyl acrylate (0.4 mmol), $Pd(OAc)_2$ (10 mol%), Ac-Gly-OH (20 mol%). Yield was determined by ^1H NMR analysis using CH_2Br_2 as internal standard. [b] Ac-Ile-OH (20 mol%) was used instead of Ac-Gly-OH. [c] Ac-Gly-OH was not added. [d] under N_2. [e] 32 h. [f] 28 h. [g] 0.1 mmol scale. [h] ethyl acrylate (0.3 mmol); 48 h. [i] DCE/HFIP (0.25mL/0.25mL). [j] $Pd(OAc)_2$ (3 mol%), Ac-Gly-OH (6 mol%; DCE/HFIP (0.3mL/0.3mL).

2.3 General remote-selective *ortho*-C–H bond olefination procedure and characterization of olefinated compounds.

General remote-selective ortho-C–H bond olefination procedure:

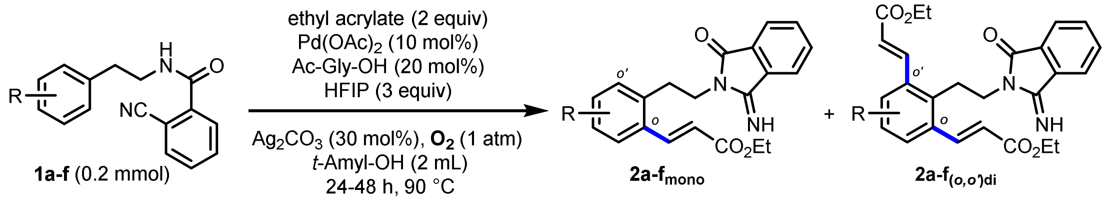

A 50 mL Schlenk sealed tube (with a Teflon cap) equipped with a magnetic stir bar was charged with amide **1** (0.20 mmol, 1.0 equiv), $Pd(OAc)_2$ (4.6 mg, 0.020 mmol, 10 mol%), Ac-Gly-OH (4.7 mg, 0.040 mmol, 20 mol%) and Ag_2CO_3 (16 mg, 0.060 mmol, 0.3 equiv). The tube was then purged with oxygen. *t*-Amyl-OH (1.0 mL) and HFIP (63 μL) was added to the mixture, followed by ethyl acrylate (2.0 equiv). And then another 1.0 mL of *t*-Amyl-OH was added along the inside wall of the tube. The tube was then capped under a flow of O_2 and submerged into a preheated 90 °C oil bath. The reaction was stirred for 20–48 h and cooled to room temperature. The crude reaction mixture was diluted with EtOAc (5 mL) and filtered through a short pad of Celite. The sealed tube and Celite pad were washed with an additional 20 mL of EtOAc. The filtrate was concentrated *in vacuo*, and the resulting residue was purified by preparative thin layer chromatography using petroleum ether/EtOAc as the eluent.

Preparation of Substrate 1a':

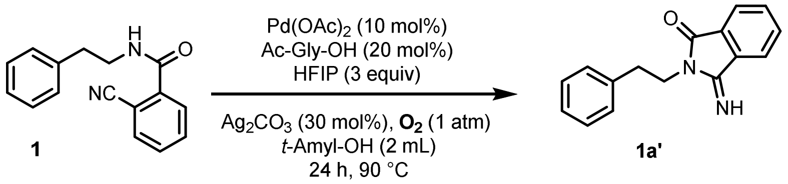

Substrate **1a'** was obtained in 85% yields (by ^{1}H NMR) using the above olefination procedure without adding ethyl acrylate in 24 h, or by heating with NaH (1.2 equiv) at 90 °C in THF for 1 h for large scale preparation in almost quantitative yield. The olefination reaction with **1a'** as the substrate using the above olefination procedure produced 32% of $\mathbf{2a_{mono}}$ and 52% of $\mathbf{2a_{(o,o')di}}$, in 23 h, by ^{1}H NMR analysis using CH_2Br_2 as internal standard.

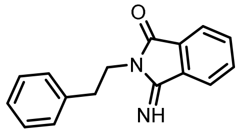

1a'

3-Imino-2-phenethylisoindolin-1-one: ^{1}H NMR (400 MHz, $CDCl_3$) δ 7.87-7.80 (m, 1H), 7.73 (d, *J* = 6.7 Hz, 1H), 7.65 (pd, *J* = 7.4, 1.3 Hz, 2H), 7.28 (d, *J* = 4.3 Hz, 4H), 7.25-7.16 (m, 1H), 4.06 (dd, *J* = 8.7, 6.8 Hz, 2H), 3.13-2.92 (m, 2H); ^{13}C NMR (101 MHz, $CDCl_3$) δ 167.7, 160.2, 138.5, 132.9, 132.2, 131.0, 128.9, 128.5, 126.5, 123.1, 120.9, 39.7, 34.5.

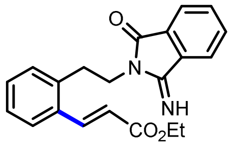

$2a_{mono}$

(*E*)-ethyl-3-(2-(2-(1-imino-3-oxoisoindolin-2-yl)ethyl)phenyl)acrylate: The corresponding reaction was run with standard conditions for 24 h. ^{1}H NMR (400 MHz, $CDCl_3$) δ 8.16 (d, *J* = 15.8 Hz, 1H), 7.80 (d, *J* = 7.1 Hz, 1H), 7.74-7.59 (m, 3H), 7.55 (d, *J* = 7.6 Hz, 1H), 7.32-7.21 (m, 3H), 6.27 (d, *J* = 15.7 Hz, 1H), 4.24 (q, *J* = 7.1 Hz, 2H), 4.03 (t, *J* = 7.3 Hz, 2H), 3.19 (t, *J* = 7.3 Hz, 2H), 1.35 (t, *J* = 7.1 Hz, 3H); ^{13}C NMR (101 MHz, $CDCl_3$) δ 167.7, 166.9, 141.9, 138.4, 133.9, 133.0, 132.3, 131.1, 130.8, 130.3, 127.4, 126.8, 123.4, 120.9, 120.1, 60.6, 39.5, 31.9, 14.5; HRMS (m/z, ESI-TOF): Calcd for $C_{21}H_{21}N_2O_3^+$ [M+H$^+$] 349.1547, found 349.1547.

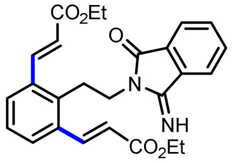

$2a_{(o,o')di}$

(2E,2'*E*)-diethyl-3,3'-(2-(2-(1-imino-3-oxoisoindolin-2-yl)ethyl)-1,3-phenylene)diacrylate: ^{1}H NMR (400 MHz, $CDCl_3$) δ 8.16 (d, *J* = 15.7 Hz, 2H), 7.77-7.72 (m, 1H), 7.68-7.55 (m, 3H), 7.52 (d, *J* = 7.8 Hz, 2H), 7.26 (t, *J* = 7.8 Hz, 1H), 6.21 (d, *J* = 15.7 Hz, 2H), 4.23 (q, *J* = 7.1 Hz, 4H), 3.99 (t, *J* = 7.0 Hz, 2H), 3.31 (t, *J* = 7.0 Hz, 2H), 1.34 (t, *J* = 7.1 Hz, 6H); ^{13}C NMR (101 MHz, $CDCl_3$) δ 167.5, 166.5, 160.1, 141.9, 137.2, 135.3, 132.8, 132.2, 131.0, 128.5, 127.6, 123.3, 121.5, 120.9, 60.6, 38.5, 27.6, 14.4; HRMS (m/z, ESI-TOF): Calcd for $C_{26}H_{27}N_2O_5^+$ [M+H$^+$] 447.1914, found 447.1907.

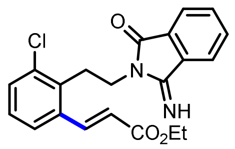

2b **(*E*)-ethyl-3-(3-chloro-2-(2-(1-imino-3-oxoisoindolin-2-yl)ethyl)phenyl) acrylate**: The corresponding reaction was run with standard conditions at 80 °C for 36 h. ^{1}H NMR (400 MHz, $CDCl_3$) δ 8.06 (d, J = 15.7 Hz, 1H), 7.75 (d, J = 7.0 Hz, 1H), 7.72-7.54 (m, 3H), 7.39 (d, J = 7.9 Hz, 1H), 7.36 (d, J = 7.8 Hz, 1H), 7.16 (t, J = 7.9 Hz, 1H), 6.07 (d, J = 15.7 Hz, 1H), 4.18 (q, J = 7.1 Hz, 2H), 4.05 (t, J = 6.5 Hz, 2H), 3.35 (t, J = 6.8 Hz, 2H), 1.31 (t, J = 7.1 Hz, 3H); ^{13}C NMR (101 MHz, $CDCl_3$) δ 167.6, 166.3, 141.7, 136.5, 135.8, 132.9, 132.2, 131.0, 130.9, 128.1, 125.4, 123.2, 121.5, 120.9, 60.6, 37.3, 28.7, 14.4; HRMS (m/z, ESI-TOF): Calcd for $C_{21}H_{20}ClN_2O_3^+$ [M+H$^+$] 383.1157, found 383.1153.

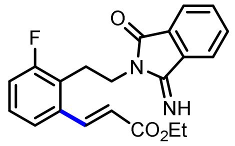

2c **(*E*)-ethyl-3-(3-fluoro-2-(2-(1-imino-3-oxoisoindolin-2-yl)ethyl)phenyl) acrylate**: The corresponding reaction was run with standard conditions at 80 °C for 48 h in 0.1 mmol scale. ^{1}H NMR (400 MHz, $CDCl_3$) δ 7.92 (d, J = 15.7 Hz, 1H), 7.68 (d, J = 7.0 Hz, 1H), 7.61 (d, J = 7.1 Hz, 1H), 7.59-7.48 (m, 2H), 7.19 (d, J = 7.7 Hz, 1H), 7.17-7.07 (m, 1H), 6.98 (t, J = 8.8 Hz, 1H), 6.04 (d, J = 15.7 Hz, 1H), 4.12 (q, J = 7.1 Hz, 2H), 3.97 (t, J = 6.7 Hz, 2H), 3.17 (t, J = 6.5 Hz, 2H), 1.25 (t, J = 7.1 Hz, 3H); ^{13}C NMR (101 MHz, $CDCl_3$) δ 167.6, 166.4, 161.8 (d, $J_{C\text{-}F}$ = 246.4 Hz), 160.2, 140.8 (d, $J_{C\text{-}F}$ = 3.0 Hz), 136.3 (d, $J_{C\text{-}F}$ = 5.1 Hz), 132.9, 132.2, 132.1, 131.0, 128.3 (d, $J_{C\text{-}F}$ = 9.1 Hz), 125.7 (d, $J_{C\text{-}F}$ = 17.2 Hz), 123.2, 122.3 (d, $J_{C\text{-}F}$ = 3.0 Hz), 121.1, 120.9, 116.5 (d, $J_{C\text{-}F}$ = 21.2 Hz), 60.6, 38.1, 24.0 (d, $J_{C\text{-}F}$ = 4.0 Hz), 14.4; HRMS (ESI-TOF): Calcd for $C_{21}H_{20}FN_2O_3^+$ [M+H$^+$] 367.1452, found 367.1450.

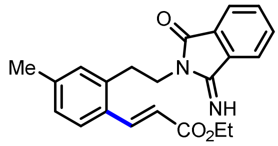

$2d_{mono}$ **(*E*)-ethyl-3-(2-(2-(1-imino-3-oxoisoindolin-2-yl)ethyl)-4-methylphenyl) acrylate**: The corresponding reaction was run with standard conditions at 70 °C for 48 h in 0.1 mmol scale. ^{1}H NMR (400 MHz, $CDCl_3$) δ 8.14 (d, J = 15.7 Hz, 1H), 7.81 (d, J = 6.8 Hz, 1H), 7.71 (d, J = 6.8 Hz, 1H), 7.69-7.59 (m, 2H), 7.46 (d, J = 8.0 Hz, 1H), 7.11 (s, 1H), 7.06 (d, J = 8.0 Hz, 1H), 6.25 (d, J = 15.7 Hz, 1H), 4.23 (q, J = 7.1 Hz, 2H), 4.01 (t, J = 7.6 Hz, 2H), 3.15 (t, J = 7.6 Hz, 2H), 2.31 (s, 3H), 1.34 (t, J = 7.1 Hz, 3H); ^{13}C NMR (101 MHz, $CDCl_3$) δ 167.7, 167.1, 141.8, 140.6, 138.3, 132.9, 132.3, 131.5, 131.2, 131.0, 128.3, 126.7, 123.3, 121.0, 119.0, 60.5, 39.6, 31.9, 21.4, 14.5; HRMS (ESI-TOF): Calcd for $C_{22}H_{23}N_2O_3^+$ [M+H$^+$] 363.1703, found 363.1701.

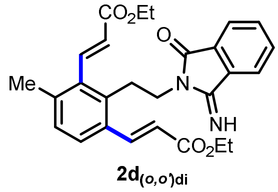

$2d_{(o,o')di}$ **(2*E*,2'*E*)-diethyl-3,3'-(2-(2-(1-imino-3-oxoisoindolin-2-yl)ethyl)-4-methyl-1,3-phenylene)diacrylate**: ^{1}H NMR (400 MHz, $CDCl_3$) δ 8.64 (s, 1H), 8.16 (d, J = 15.6

Hz, 1H), 7.91 (d, *J* = 16.3 Hz, 1H), 7.77 (d, *J* = 6.9 Hz, 1H), 7.72-7.56 (m, 3H), 7.41 (d, *J* = 8.0 Hz, 1H), 7.14 (d, *J* = 8.0 Hz, 1H), 6.17 (d, *J* = 15.6 Hz, 1H), 6.08 (d, *J* = 16.3 Hz, 1H), 4.31 (q, *J* = 7.1 Hz, 2H), 4.23 (q, *J* = 7.1 Hz, 2H), 3.94 (brs, 2H), 3.20 (t, *J* = 7.1 Hz, 2H), 2.33 (d, *J* = 9.2 Hz, 3H), 1.40-1.32 (m, 6H); ^{13}C NMR (101 MHz, $CDCl_3$) δ 167.5, 166.8, 166.3, 143.1, 142.1, 138.4, 136.3, 132.9, 132.4, 132.2, 131.1, 129.6, 126.6, 126.2, 123.3, 120.9, 120.0, 60.8, 60.6, 38.3, 28.6, 21.5, 14.5; HRMS (ESI-TOF): Calcd for $C_{27}H_{29}N_2O_5^+$ [M+H$^+$] 461.2071, found 461.2064.

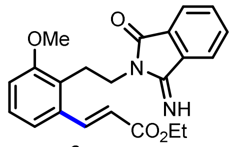

2e **(*E*)-ethyl-3-(2-(2-(1-imino-3-oxoisoindolin-2-yl)ethyl)-3-methoxyphenyl) acrylate**: The corresponding reaction was run with standard conditions for 24 h. ^{1}H NMR (400 MHz, $CDCl_3$) δ 8.03 (d, *J* = 15.7 Hz, 1H), 7.74 (d, *J* = 6.7 Hz, 1H), 7.70 (d, *J* = 7.0 Hz, 1H), 7.64-7.54 (m, 2H), 7.18 (t, *J* = 8.0 Hz, 1H), 7.07 (d, *J* = 7.8 Hz, 1H), 6.86 (d, *J* = 8.1 Hz, 1H), 6.08 (d, *J* = 15.7 Hz, 1H), 4.18 (q, *J* = 7.1 Hz, 2H), 3.98 (t, *J* = 6.8 Hz, 2H), 3.81 (s, 3H), 3.21 (t, *J* = 6.8 Hz, 2H), 1.31 (t, *J* = 7.2 Hz, 3H); ^{13}C NMR (101 MHz, $CDCl_3$) δ 167.6, 166.6, 159.9, 158.1, 142.1, 135.3, 132.8, 132.1, 131.1, 127.9, 127.1, 123.1, 121.0, 120.5, 118.8, 111.5, 60.4, 55.7, 37.8, 24.7, 14.4; HRMS (ESI-TOF): Calcd for $C_{22}H_{23}N_2O_4^+$ [M+H$^+$] 379.1652, found 379.1663.

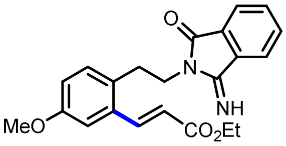

$2f_{mono}$ **(*E*)-ethyl-3-(2-(2-(1-imino-3-oxoisoindolin-2-yl)ethyl)-5-methoxyphenyl)acrylate**: The corresponding reaction was run with standard conditions for 24 h. ^{1}H NMR (400 MHz, $CDCl_3$) δ 8.11 (d, *J* = 15.7 Hz, 1H), 7.81 (d, *J* = 6.6 Hz, 1H), 7.76 (d, *J* = 7.0 Hz, 1H), 7.72-7.59 (m, 2H), 7.20 (d, *J* = 8.4 Hz, 1H), 7.05 (d, *J* = 2.5 Hz, 1H), 6.87 (dd, *J* = 8.4, 2.5 Hz, 1H), 6.25 (d, *J* = 15.7 Hz, 1H), 4.24 (q, *J* = 7.1 Hz, 2H), 4.00 (t, *J* = 7.3 Hz, 2H), 3.80 (s, 3H), 3.13 (t, *J* = 7.3 Hz, 2H), 1.35 (t, *J* = 7.1 Hz, 3H); ^{13}C NMR (101 MHz, $CDCl_3$) δ 167.7, 166.9, 158.7, 141.9, 134.8, 133.1, 132.5, 132.1, 131.9, 131.0, 130.6, 123.4, 121.2, 120.1, 116.6, 111.3, 60.6, 55.4, 39.8, 31.1, 14.5; HRMS (ESI-TOF): Calcd for $C_{22}H_{23}N_2O_4^+$ [M+H$^+$] 379.1652, found 379.1659.

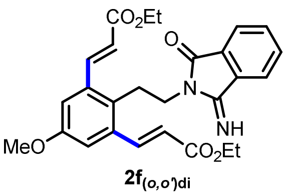

$2f_{(o,o')di}$ **(2*E*,2'*E*)-diethyl-3,3'-(2-(2-(1-imino-3-oxoisoindolin-2-yl)ethyl)-5-methoxy-1,3-phenylene)diacrylate**: ^{1}H NMR (400 MHz, $CDCl_3$) δ 8.13 (d, *J* = 15.6 Hz, 2H), 7.75 (dd, *J* = 6.3, 1.4 Hz, 1H), 7.69-7.52 (m, 3H), 7.06 (s, 2H), 6.21 (d, *J* = 15.6 Hz, 2H), 4.24 (q, *J* = 7.1 Hz, 4H), 3.95 (t, *J* = 7.0 Hz, 2H), 3.81 (s, 3H), 3.25 (t, *J* = 7.0 Hz, 2H), 1.34 (t, *J* = 7.1 Hz, 6H); ^{13}C NMR (101 MHz, $CDCl_3$) δ 167.5, 166.6, 158.4, 142.0, 136.5, 133.9, 132.9, 132.2, 131.1, 129.9, 123.3, 121.9, 121.6, 120.9, 114.0, 60.7, 55.5, 38.8, 27.0, 14.5; HRMS (ESI-TOF): Calcd for $C_{27}H_{29}N_2O_6^+$ [M+H$^+$] 477.2020, found 477.2029.

2.4 General remote-selective *meta*-C–H bond olefination procedure and characterization of olefinated compounds.

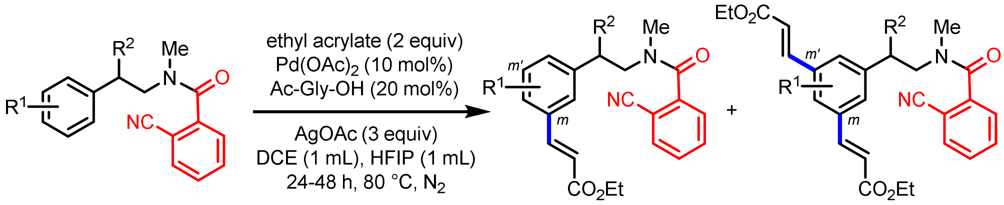

3a-p **4a-p$_{mono}$** **4a-p$_{(m,m')di}$**

A 50 mL Schlenk sealed tube (with a Teflon cap) equipped with a magnetic stir bar was charged with amide **3** (0.20 mmol, 1.0 equiv), $Pd(OAc)_2$ (4.6 mg, 0.020 mmol, 10 mol%), Ac-Gly-OH (4.7 mg, 0.040 mmol, 20 mol%) and AgOAc (100 mg, 0.60 mmol, 3 equiv). The tube was then purged with nitrogen. DCE (1.0 mL) was added to the mixture, followed by ethyl acrylate (2.0 equiv) [and DMF (77 μL, 1.0 mmol, 5 equiv) when necessary as noted below]. And then 1.0 mL of HFIP was added along the inside wall of the tube. The tube was then capped under a flow of nitrogen and submerged into a preheated 80 °C oil bath. The reaction was stirred for 24–48 h and cooled to room temperature. The crude reaction mixture was diluted with EtOAc (5 mL) and filtered through a short pad of Celite. The sealed tube and Celite pad were washed with an additional 20 mL of EtOAc. The filtrate was concentrated *in vacuo*, and the resulting residue was purified by preparative thin layer chromatography using petroleum ether/EtOAc as the eluent. The site selectivity was assigned by ^{1}H NMR analysis of the product or the hydrolyzed product with EtOH/HCl (see **Section 2.6**).

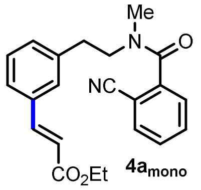

4a$_{mono}$ **(*E*)-ethyl-3-(3-(2-(2-cyano-*N*-methylbenzamido)ethyl)phenyl)acrylate**: The corresponding reaction was run with standard conditions for 32 h. The two rotamers' ratio is about 55:45. ^{1}H NMR (400 MHz, $CDCl_3$) δ 7.73-7.66 (m, 0.85H), 7.68-7.58 (m, 1.37H), 7.56 (d, *J* = 16.1 Hz, 0.50H), 7.53-7.40 (m, 2.16H), 7.42-7.31 (m, 2.75H), 7.27 (t, *J* = 7.6 Hz, 0.74H), 7.02 (s, 0.44H), 6.98 (d, *J* = 7.6 Hz, 0.43H), 6.80 (dd, *J* = 6.0, 2.9 Hz, 0.46H), 6.45 (d, *J* = 16.0 Hz, 0.55H), 6.33 (d, *J* = 16.0 Hz, 0.44H), 4.26 (q, *J* = 7.1 Hz, 2.14H), 3.81 (t, *J* = 7.6 Hz, 1.12H), 3.45 (t, *J* = 6.9 Hz, 0.92H), 3.20 (s, 1.47H), 3.05 (t, *J* = 7.6 Hz, 1.13H), 2.83 (s, 2.67H), 1.37-1.30 (m, 3.28H); ^{13}C NMR (101 MHz, $CDCl_3$) δ 168.0, 167.6, 167.1, 166.9, 144.5, 144.0, 140.6, 140.3, 139.6, 138.5, 135.1, 134.9, 133.2, 133.0, 132.9, 132.7, 131.0, 130.8, 129.6, 129.5, 129.4, 129.3, 128.6, 128.4, 127.6, 127.4, 126.6, 126.4, 118.9, 118.6, 116.9, 116.8, 110.0, 109.8, 60.7, 60.6, 52.4, 49.4, 37.4, 34.4, 33.2, 33.0, 14.4; HRMS (m/z, ESI-TOF): Calcd for $C_{22}H_{23}N_2O_3^+$ [M+H$^+$] 363.1703, found 363.1703.

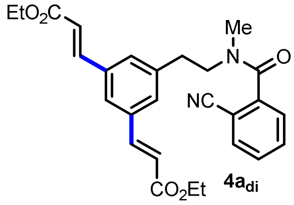

4a$_{di}$ **(2*E*,2'*E*)-diethyl-3,3'-(5-(2-(2-cyano-*N*-methylbenzamido)ethyl)-1,3-phenylene)diacrylate**: The two rotamers' ratio is about 3:2. ^{1}H NMR (400 MHz, $CDCl_3$) δ 7.74-7.59 (m, 3.17H), 7.57 (d, *J* = 16.0 Hz, 0.81H), 7.55-7.44 (m, 3.66H), 7.37 (d, *J* = 7.7 Hz, 0.61H),

7.06 (s, 0.72H), 6.92-6.84 (m, 0.38H), 6.49 (d, *J* = 16.0 Hz, 1.17H), 6.37 (d, *J* = 16.0 Hz, 0.78H), 4.27 (q, *J* = 7.1 Hz, 4.27H), 3.82 (t, *J* = 7.6 Hz, 1.19H), 3.49 (t, *J* = 6.9 Hz, 0.79H), 3.20 (s, 1.30H), 3.08 (t, *J* = 7.6 Hz, 1.24H), 2.91-2.79 (m, 2.69H), 1.39-1.30 (m, 6.41H); ^{13}C NMR (101 MHz, $CDCl_3$) δ 167.9, 167.7, 166.8, 166.6, 143.6, 143.1, 140.5, 140.3, 140.2, 139.2, 135.8, 135.6, 133.2, 133.0, 132.8, 130.1, 129.7, 129.6, 129.5, 127.6, 127.4, 126.1, 119.9, 119.6, 116.8, 116.8, 110.0, 109.9, 60.8, 60.7, 52.2, 49.3, 37.4, 34.3, 33.1, 33.0, 14.4; HRMS (m/z, ESI-TOF): Calcd for $C_{27}H_{29}N_2O_5^+$ [M+H$^+$] 461.2071, found 461.2062.

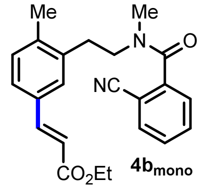

4b$_{mono}$ **(*E*)-ethyl-3-(3-(2-(2-cyano-*N*-methylbenzamido)ethyl)-4-methylphenyl) acrylate**: The corresponding reaction was run with standard conditions and DMF (5 equiv) at 90 °C for 36 h. The two rotamers' ratio is about 54:46. ^{1}H NMR (400 MHz, $CDCl_3$) δ 7.75-7.60 (m, 2.09H), 7.59-7.37 (m, 3.01H), 7.31 (t, *J* = 9.9 Hz, 1.00H), 7.19 (d, *J* = 7.6 Hz, 0.60H), 7.07 (d, *J* = 8.0 Hz, 0.44H), 7.00 (s, 0.44H), 6.96 (d, *J* = 7.3 Hz, 0.41H), 6.42 (d, *J* = 16.0 Hz, 0.54H), 6.31 (d, *J* = 16.0 Hz, 0.46H), 4.26 (q, *J* = 6.9 Hz, 2.00H), 3.74 (t, *J* = 7.9 Hz, 1.09H), 3.40 (t, *J* = 7.2 Hz, 0.92H), 3.25 (s, 1.29H), 3.06 (t, *J* = 8.0 Hz, 1.09H), 2.91 (s, 1.69H), 2.83 (t, *J* = 6.9 Hz, 0.93H), 2.46 (s, 1.68H), 2.00 (s, 1.29H), 1.34 (t, *J* = 7.0 Hz, 3.00H); ^{13}C NMR (101 MHz, $CDCl_3$) δ 167.9, 167.6, 167.2, 167.0, 144.5, 144.0, 140.6, 140.3, 139.4, 138.8, 137.5, 136.4, 133.2, 133.0, 133.0, 132.8, 132.7, 132.5, 131.3, 131.1, 129.6, 129.4, 129.4, 129.3, 127.6, 127.4, 126.6, 126.5, 117.9, 117.5, 116.8, 116.8, 110.0, 109.9, 60.6, 60.5, 51.3, 48.3, 37.2, 33.2, 32.0, 30.8, 19.4, 18.9, 14.4; HRMS (m/z, ESI-TOF): Calcd for $C_{23}H_{25}N_2O_3^+$ [M+H$^+$] 377.1860, found 377.1857.

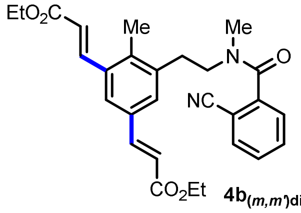

4b$_{(m,m')di}$ **(2*E*,2'*E*)-diethyl-3,3'-(5-(2-(2-cyano-*N*-methylbenzamido)ethyl)-4-methyl-1,3-phenylene)diacrylate**: The two rotamers' ratio is about 3:2. ^{1}H NMR (400 MHz, $CDCl_3$) δ 8.03 (d, *J* = 15.8 Hz, 0.62H), 7.85 (d, *J* = 15.8 Hz, 0.45H), 7.72 (d, *J* = 7.8 Hz, 0.66H), 7.71-7.61 (m, 1.63H), 7.61-7.38 (m, 4.02H), 7.09 (s, 0.38H), 7.00-6.89 (m, 0.38H), 6.47 (d, *J* = 16.0 Hz, 0.60H), 6.42-6.27 (m, 1.40H), 4.36-4.21 (m, 4.04H), 3.73 (dd, *J* = 9.0, 6.9 Hz, 1.17H), 3.42 (t, *J* = 7.2 Hz, 0.84H), 3.26 (s, 1.24H), 3.13 (dd, *J* = 6.5, 3.7 Hz, 1.20H), 2.94 (s, 1.80H), 2.89 (t, *J* = 7.3 Hz, 0.80H), 2.52 (s, 1.73H), 1.95 (s, 1.12H), 1.44-1.30 (m, 6.07H); ^{13}C NMR (101 MHz, $CDCl_3$) δ 167.9, 167.7, 167.0, 166.8, 166.8, 166.7, 143.8, 143.3, 142.6, 142.1, 140.5, 140.2, 138.5, 138.5, 137.7, 137.4, 135.6, 135.5, 133.3, 133.0, 133.0, 132.8, 132.8, 132.6, 130.7, 130.7, 129.7, 129.6, 127.7, 127.5, 125.3, 125.2, 121.5, 121.2, 119.0, 118.6, 116.9, 116.8, 110.1, 110.0, 60.9, 60.8, 60.7, 51.2, 48.6, 37.4, 33.3, 32.8, 31.5, 15.6, 15.0, 14.4; HRMS (m/z, ESI-TOF): Calcd for $C_{28}H_{31}N_2O_5^+$ [M+H$^+$] 475.2227, found 475.2220.

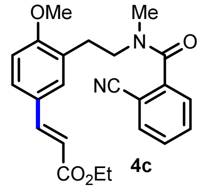

4c **(*E*)-ethyl-3-(3-(2-(2-cyano-*N*-methylbenzamido)ethyl)-4-methoxyphenyl) acrylate**: The corresponding reaction was run with standard conditions and DMF (5 equiv) for 24 h. The two rotamers' ratio is about 2:3. ^{1}H NMR (400 MHz, $CDCl_3$) δ 7.68 (d, *J* = 7.7 Hz, 0.46H), 7.66-7.57 (m, 1.54H), 7.54 (d, *J* = 15.9 Hz, 0.66H), 7.50-7.42 (m, 2.03H), 7.42-7.34 (m, 1.09H), 7.31 (d, *J* = 7.7 Hz, 0.42H), 7.08 (s, 0.56H), 6.91-6.83 (m, 1.00H), 6.73 (d, *J* = 8.5 Hz, 0.58H), 6.33 (d, *J* = 16.0 Hz, 0.40H), 6.22 (d, *J* = 16.0 Hz, 0.58H), 4.24 (q, *J* = 7.1 Hz, 2.16H), 3.89 (s, 1.25H), 3.79 (t, *J* = 7.3 Hz, 0.88H), 3.60 (s, 1.82H), 3.42 (t, *J* = 6.9 Hz, 1.25H), 3.20 (s, 1.93H), 3.03 (t, *J* = 7.3 Hz, 0.84H), 2.84-2.78 (m, 2.52H), 1.32 (t, *J* = 7.1 Hz, 3.16H); ^{13}C NMR (101 MHz, $CDCl_3$) δ 167.9, 167.6, 167.4, 167.2, 159.7, 159.4, 144.3, 143.9, 140.9, 140.6, 133.1, 133.0, 132.8, 132.6, 130.3, 130.2, 129.4, 129.1, 128.8, 127.9, 127.8, 127.4, 127.3, 127.1, 126.6, 116.9, 116.3, 116.0, 110.7, 110.6, 110.0, 60.5, 60.4, 55.7, 55.3, 50.8, 47.5, 37.3, 33.0, 29.5, 28.4, 14.4; HRMS (m/z, ESI-TOF): Calcd for $C_{23}H_{25}N_2O_4^+$ [M+H$^+$] 393.1809, found 393.1805.

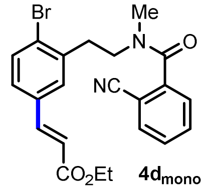

$4d_{mono\ (m)}$ **(*E*)-ethyl-3-(4-bromo-3-(2-(2-cyano-*N*-methylbenzamido)ethyl)phenyl) acrylate**: The corresponding reaction was run with standard conditions and ethyl acrylate (2.5 equiv) at 90 °C for 36 h using HFIP as the sole solvent. The two rotamers' ratio is about 54:46. ^{1}H NMR (400 MHz, $CDCl_3$) δ 7.72 (d, *J* = 7.7 Hz, 0.54H), 7.70-7.63 (m, 1.29H), 7.60 (d, *J* = 7.2 Hz, 0.55H), 7.58-7.48 (m, 2.86H), 7.45 (d, *J* = 8.3 Hz, 0.57H), 7.42 (d, *J* = 7.7 Hz, 0.54H), 7.28 (d, *J* = 2.6 Hz, 0.48H), 7.25 (dd, *J* = 6.0, 2.2 Hz, 0.56H), 7.14 (d, *J* = 1.9 Hz, 0.52H), 7.01 (dd, *J* = 7.4, 1.5 Hz, 0.47H), 6.48 (d, *J* = 16.0 Hz, 0.52H), 6.37 (d, *J* = 16.0 Hz, 0.45H), 4.31-4.23 (m, 2.00H), 3.82 (dd, *J* = 8.4, 6.9 Hz, 1.08H), 3.50 (t, *J* = 7.2 Hz, 0.97H), 3.26 (s, 1.44H), 3.21 (dd, *J* = 8.7, 6.6 Hz, 1.08H), 2.97 (t, *J* = 7.3 Hz, 0.99H), 2.89 (s, 1.66H), 1.38-1.31 (m, 3.07H); ^{13}C NMR (101 MHz, $CDCl_3$) δ 168.0, 167.8, 166.8, 166.6, 143.1, 142.7, 140.6, 140.3, 138.9, 137.7, 134.5, 134.4, 133.8, 133.6, 133.2, 133.1, 133.1, 132.9, 130.9, 130.6, 129.6, 129.5, 127.8, 127.7, 127.7, 127.5, 126.4, 126.4, 119.7, 119.5, 116.9, 116.9, 110.1, 110.1, 60.8, 60.7, 50.6, 47.8, 37.4, 35.1, 33.7, 33.3, 14.4; HRMS (m/z, ESI-TOF): Calcd for $C_{22}H_{22}BrN_2O_3^+$ [M+H$^+$] 441.0808, found 441.0818.

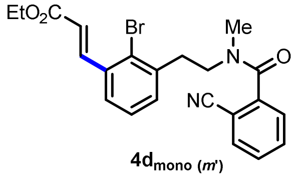

$4d_{mono\ (m')}$ **(*E*)-ethyl-3-(2-bromo-3-(2-(2-cyano-*N*-methylbenzamido)ethyl) phenyl)acrylate**: The two rotamers' ratio is about 1:1. ^{1}H NMR (400 MHz, $CDCl_3$) δ 8.14 (d, *J* = 15.9 Hz, 0.43H), 7.93 (d, *J* = 15.9 Hz, 0.47H), 7.72 (d, *J* = 7.8 Hz, 0.53H), 7.70-7.61 (m, 1.05H), 7.55-7.40 (m, 3.63H), 7.34-7.23 (m, 1.13H), 7.10 (dd, *J* = 7.5, 1.4 Hz, 0.49H), 6.84-6.79 (m, 0.45H), 6.41-6.30 (m, 0.90H), 4.28 (q, *J* = 7.1 Hz, 2.07H), 3.82 (dd, *J* = 8.5, 6.7 Hz, 0.99H), 3.54 (t, *J* = 7.0 Hz, 1.00H), 3.31-3.18 (m, 2.67H), 3.00 (t, *J* = 7.0 Hz, 1.11H), 2.88 (s, 1.47H), 1.35 (t, *J* = 7.0 Hz, 3.01H); ^{13}C NMR (101 MHz, $CDCl_3$) δ 168.0, 167.6, 166.4, 166.3, 143.8, 143.4, 140.5, 140.0, 139.5, 138.2, 135.9, 135.6, 133.1, 133.0, 132.8, 132.7, 132.4, 129.5, 129.4,

127.9, 127.8, 127.5, 127.4, 127.2, 127.1, 126.8, 126.5, 121.7, 121.4, 116.8, 116.8, 109.9, 109.9, 60.8, 60.7, 50.1, 47.7, 37.4, 35.5, 34.3, 33.1, 14.3; HRMS (m/z, ESI-TOF): Calcd for $C_{22}H_{22}BrN_2O_3^+$ [M+H$^+$] 441.0808, found 441.0819.

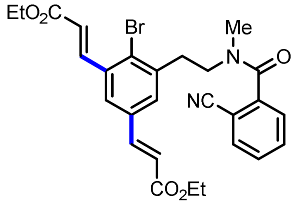

4d$_{(m,m')di}$ **(2*E*,2'*E*)-diethyl-3,3'-(4-bromo-5-(2-(2-cyano-*N*-methylbenzamido)ethyl)-1,3-phenylene)diacrylate**: The two rotamers' ratio is about 53:47. ^{1}H NMR (400 MHz, $CDCl_3$) δ 8.10 (d, *J* = 15.9 Hz, 0.53H), 7.91 (d, *J* = 15.9 Hz, 0.47H), 7.73 (d, *J* = 7.7 Hz, 0.60H), 7.71-7.63 (m, 1.43H), 7.63-7.55 (m, 1.80H), 7.57-7.46 (m, 1.81H), 7.43 (d, *J* = 7.6 Hz, 0.60H), 7.20 (d, *J* = 1.6 Hz, 0.41H), 7.01-6.96 (m, 0.39H), 6.53 (d, *J* = 16.0 Hz, 0.52H), 6.48-6.33 (m, 1.37H), 4.34-4.23 (m, 4.10H), 3.90-3.80 (m, 1.07H), 3.54 (t, *J* = 7.1 Hz, 0.92H), 3.33-3.21 (m, 2.56H), 3.03 (t, *J* = 7.1 Hz, 0.94H), 2.92 (s, 1.52H), 1.40-1.31 (m, 6.18H); ^{13}C NMR (101 MHz, $CDCl_3$) δ 167.9, 167.8, 166.5, 166.3, 166.2, 166.1, 143.3, 142.8, 142.4, 142.0, 140.5, 140.3, 140.1, 139.0, 136.7, 136.4, 134.3, 134.2, 133.2, 133.0, 133.0, 132.9, 131.3, 131.0, 129.6, 129.6, 128.6, 128.4, 127.6, 127.4, 125.9, 125.9, 122.6, 122.3, 120.5, 120.3, 116.9, 116.8, 110.1, 110.0, 61.0, 60.9, 60.8, 50.2, 47.6, 37.4, 35.7, 34.3, 33.3, 14.4; HRMS (m/z, ESI-TOF): Calcd for $C_{27}H_{28}BrN_2O_5^+$ [M+H$^+$] 539.1176, found 539.1185.

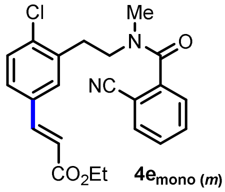

4e$_{mono\ (m)}$ **(*E*)-ethyl-3-(4-chloro-3-(2-(2-cyano-*N*-methylbenzamido)ethyl)phenyl)acrylate**: The corresponding reaction was run with standard conditions and ethyl acrylate (2.5 equiv) at 90 ^{o}C for 48 h using HFIP as the sole solvent. The two rotamers' ratio is about 53:47. ^{1}H NMR (400 MHz, $CDCl_3$) δ 7.72 (d, *J* = 7.7 Hz, 0.53H), 7.72-7.59 (m, 1.54H), 7.59-7.45 (m, 2.53H), 7.44-7.31 (m, 2.13H), 7.30-7.24 (m, 0.95H), 7.15 (d, *J* = 2.1 Hz, 0.50H), 7.01 (d, *J* = 7.6 Hz, 0.50H), 6.46 (d, *J* = 16.0 Hz, 0.53H), 6.35 (d, *J* = 16.0 Hz, 0.47H), 4.27 (q, *J* = 7.1 Hz, 2.00H), 3.83 (dd, *J* = 8.6, 6.5 Hz, 1.06H), 3.49 (t, *J* = 7.2 Hz, 0.94H), 3.25 (s, 1.38H), 3.20 (dd, *J* = 8.5, 6.6 Hz, 1.07H), 2.96 (t, *J* = 7.2 Hz, 0.94H), 2.88 (s, 1.61H), 1.34 (t, *J* = 7.1 Hz, 3.05H); ^{13}C NMR (101 MHz, $CDCl_3$) δ 168.0, 167.7, 166.9, 166.7, 143.1, 142.7, 140.6, 140.3, 137.2, 136.0, 136.0, 133.9, 133.8, 133.2, 133.1, 132.9, 131.0, 130.7, 130.4, 130.3, 129.6, 129.5, 127.7, 127.7, 127.5, 119.6, 119.4, 116.9, 116.9, 110.1, 60.8, 60.7, 50.5, 47.7, 37.4, 33.3, 32.7, 31.3, 14.4; HRMS (m/z, ESI-TOF): Calcd for $C_{22}H_{22}ClN_2O_3^+$ [M+H$^+$] 397.1313, found 397.1324.

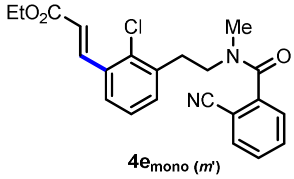

4e$_{mono\ (m')}$ **(*E*)-ethyl-3-(2-chloro-3-(2-(2-cyano-*N*-methylbenzamido)ethyl)phenyl)acrylate**: The two rotamers' ratio is about 1:1. ^{1}H NMR (400 MHz, $CDCl_3$) δ 8.17 (d, *J* = 16.0 Hz, 0.43H), 7.96 (d, *J* = 16.0 Hz, 0.47H), 7.72 (d, *J* = 7.7 Hz, 0.55H), 7.71-7.62 (m, 1.09H), 7.57-7.50 (m, 1.53H), 7.50-7.39 (m, 2.19H), 7.28 (t, *J* = 3.8 Hz, 0.57H), 7.23 (t, *J* = 7.7

Hz, 0.51H), 7.12 (d, *J* = 7.4 Hz, 0.50H), 6.88-6.81 (m, 0.48H), 6.45-6.35 (m, 1.00H), 4.29 (q, *J* = 7.1 Hz, 2.11H), 3.84 (dd, *J* = 8.6, 6.5 Hz, 1.05H), 3.53 (t, *J* = 7.0 Hz, 1.09H), 3.31-3.19 (m, 2.63H), 2.99 (t, *J* = 7.0 Hz, 1.06H), 2.87 (s, 1.50H), 1.36 (t, *J* = 7.1 Hz, 3.18H); ^{13}C NMR (101 MHz, CDCl$_3$) δ 168.1, 167.7, 166.6, 166.4, 141.1, 140.6, 140.1, 137.8, 136.4, 134.8, 134.7, 133.9, 133.7, 133.2, 133.1, 132.9, 132.9, 132.8, 132.6, 129.6, 129.5, 127.6, 127.4, 127.3, 127.2, 126.7, 126.4, 121.6, 121.3, 116.9, 116.8, 110.1, 110.0, 60.9, 60.8, 50.1, 47.7, 37.4, 33.2, 33.2, 31.7, 14.4; HRMS (m/z, ESI-TOF): Calcd for $C_{22}H_{22}ClN_2O_3^+$ [M+H$^+$] 397.1313, found 397.1324.

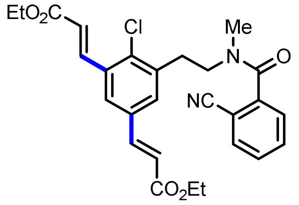

4e$_{(m,m')di}$ **(2*E*,2'*E*)-diethyl-3,3'-(4-chloro-5-(2-(2-cyano-*N*-methylbenzamido)ethyl)-1,3-phenylene)diacrylate**: The two rotamers' ratio is about 55:45. ^{1}H NMR (400 MHz, CDCl$_3$) δ 8.09 (d, *J* = 16.0 Hz, 0.52H), 7.89 (d, *J* = 16.0 Hz, 0.43H), 7.69 (d, *J* = 7.7 Hz, 0.58H), 7.67-7.56 (m, 3.05H), 7.53 (d, *J* = 16.3 Hz, 0.64H), 7.51-7.43 (m, 1.28H), 7.39 (d, *J* = 7.7 Hz, 0.56H), 7.19 (d, *J* = 1.8 Hz, 0.47H), 7.02-6.93 (m, 0.45H), 6.48 (d, *J* = 16.1 Hz, 0.51H), 6.46-6.34 (m, 1.42H), 4.31-4.19 (m, 4.00H), 3.85-3.77 (m, 1.09H), 3.49 (t, *J* = 7.2 Hz, 0.91H), 3.26-3.17 (m, 2.47H), 2.98 (t, *J* = 7.2 Hz, 0.91H), 2.87 (s, 1.55H), 1.36-1.27 (m, 6.01H); ^{13}C NMR (101 MHz, CDCl$_3$) δ 167.9, 167.7, 166.5, 166.3, 166.3, 166.1, 142.4, 142.0, 140.5, 140.4, 140.1, 140.0, 138.4, 137.1, 136.1, 136.0, 134.5, 134.3, 133.7, 133.6, 133.2, 133.0, 132.9, 132.8, 131.5, 131.2, 129.6, 129.6, 127.5, 127.4, 125.9, 125.8, 122.5, 122.2, 120.4, 120.2, 116.8, 116.8, 110.1, 110.0, 61.0, 60.9, 60.8, 60.7, 50.1, 47.5, 37.4, 33.2, 33.2, 31.7, 14.3; HRMS (m/z, ESI-TOF): Calcd for $C_{27}H_{28}ClN_2O_5^+$ [M+H$^+$] 495.1681, found 495.1689.

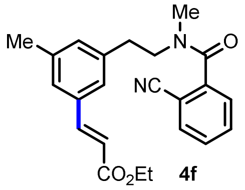

4f **(*E*)-ethyl-3-(3-(2-(2-cyano-*N*-methylbenzamido)ethyl)-5-methylphenyl)acrylate**: The corresponding reaction was run with standard conditions and DMF (5 equiv) for 32 h. The two rotamers' ratio is about 53:47. ^{1}H NMR (400 MHz, CDCl$_3$) δ 7.69 (d, *J* = 7.7 Hz, 0.61H), 7.68-7.58 (m, 1.54H), 7.53 (d, *J* = 16.4 Hz, 0.66H), 7.51-7.41 (m, 1.34H), 7.37 (d, *J* = 7.7 Hz, 0.57H), 7.27 (s, 0.57H), 7.20 (s, 1.01H), 7.16 (s, 0.49H), 6.86-6.69 (m, 1.39H), 6.43 (d, *J* = 16.0 Hz, 0.50H), 6.31 (d, *J* = 16.0 Hz, 0.44H), 4.25 (q, *J* = 7.1 Hz, 2.02H), 3.78 (dd, *J* = 8.8, 6.5 Hz, 1.05H), 3.44 (t, *J* = 6.9 Hz, 0.95H), 3.19 (s, 1.43H), 3.00 (t, *J* = 7.7 Hz, 1.05H), 2.83 (s, 1.58H), 2.78 (t, *J* = 6.8 Hz, 0.95H), 2.35 (s, 1.57H), 2.28 (s, 1.44H), 1.36-1.28 (m, 3.03H); ^{13}C NMR (101 MHz, CDCl$_3$) δ 167.9, 167.6, 167.1, 166.9, 144.6, 144.2, 140.7, 140.3, 139.4, 139.2, 138.9, 138.3, 135.0, 134.8, 133.2, 133.0, 132.8, 132.7, 131.9, 131.7, 129.5, 129.3, 127.7, 127.4, 127.2, 127.1, 125.8, 125.6, 118.6, 118.3, 116.9, 116.8, 110.0, 109.8, 60.6, 60.5, 52.4, 49.5, 37.4, 34.2, 33.1, 32.9, 21.3, 21.2, 14.4; HRMS (m/z, ESI-TOF): Calcd for $C_{23}H_{25}N_2O_3^+$ [M+H$^+$] 377.1860, found 377.1856.

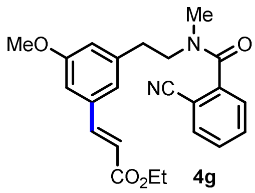

4g **(*E*)-ethyl-3-(3-(2-(2-cyano-*N*-methylbenzamido)ethyl)-5-methoxyphenyl) acrylate**: The corresponding reaction was run with standard conditions and DMF (5 equiv) for 32 h. The two rotamers' ratio is about 54:46. ^{1}H NMR (400 MHz, $CDCl_3$) δ 7.69 (d, *J* = 7.7 Hz, 0.65H), 7.67-7.57 (m, 1.68H), 7.56-7.38 (m, 2.22H), 7.36 (d, *J* = 7.7 Hz, 0.56H), 7.06 (s, 0.49H), 6.94-6.83 (m, 1.96H), 6.62 (s, 0.40H), 6.49 (s, 0.43H), 6.43 (d, *J* = 16.0 Hz, 0.51H), 6.31 (d, *J* = 16.0 Hz, 0.44H), 4.25 (q, *J* = 7.0 Hz, 2.15H), 3.84-3.77 (m, 2.77H), 3.75 (s, 1.48H), 3.45 (t, *J* = 6.9 Hz, 0.91H), 3.18 (s, 1.37H), 3.01 (t, *J* = 7.6 Hz, 1.09H), 2.83 (s, 1.58H), 2.78 (t, *J* = 6.8 Hz, 0.93H), 1.36-1.29 (m, 3.32H); ^{13}C NMR (101 MHz, $CDCl_3$) δ 168.0, 167.6, 167.0, 166.8, 160.3, 160.3, 144.5, 144.0, 141.0, 140.7, 140.3, 139.8, 136.3, 136.1, 133.2, 133.0, 132.9, 132.7, 129.6, 129.4, 127.7, 127.4, 121.4, 121.1, 119.1, 118.9, 116.9, 116.8, 116.7, 111.4, 111.2, 110.0, 109.9, 60.7, 60.6, 55.5, 55.4, 52.3, 49.3, 37.4, 34.5, 33.4, 33.0, 14.4; HRMS (m/z, ESI-TOF): Calcd for $C_{23}H_{25}N_2O_4^+$ [M+H$^+$] 393.1809, found 393.1805.

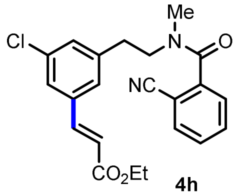

4h **(*E*)-ethyl-3-(3-chloro-5-(2-(2-cyano-*N*-methylbenzamido)ethyl) phenyl)acrylate**: The corresponding reaction was run with standard conditions and DMF (5 equiv) for 32 h. The two rotamers' ratio is about 3:2. ^{1}H NMR (400 MHz, $CDCl_3$) δ 7.73-7.57 (m, 2.11H), 7.56 (s, 0.38H), 7.54-7.44 (m, 1.93H), 7.41-7.32 (m, 2.15H), 7.30 (s, 0.56H), 6.95-6.83 (m, 1.07H), 6.44 (d, *J* = 16.0 Hz, 0.58H), 6.33 (d, *J* = 16.0 Hz, 0.38H), 4.24 (q, *J* = 6.9 Hz, 2.07H), 3.78 (t, *J* = 7.6 Hz, 1.28H), 3.45 (t, *J* = 6.8 Hz, 0.78H), 3.17 (s, 1.32H), 3.02 (t, *J* = 7.6 Hz, 1.26H), 2.84 (s, 1.79H), 2.84-2.76 (m, 0.87H), 1.31 (t, *J* = 7.0 Hz, 3.15H); ^{13}C NMR (101 MHz, $CDCl_3$) δ 167.9, 167.7, 166.6, 166.4, 142.9, 142.4, 141.4, 140.5, 140.3, 140.1, 136.7, 136.6, 135.3, 135.0, 133.2, 133.0, 132.8, 130.5, 130.3, 129.6, 129.6, 127.6, 127.4, 127.0, 126.7, 126.2, 126.1, 120.4, 120.1, 116.8, 116.7, 110.0, 109.8, 60.8, 60.7, 52.0, 49.2, 37.4, 34.1, 32.9, 14.3; HRMS (m/z, ESI-TOF): Calcd for $C_{22}H_{22}ClN_2O_3^+$ [M+H$^+$] 397.1313, found 397.1309.

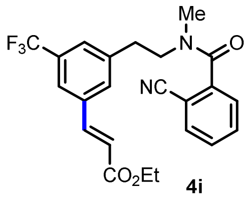

4i **(*E*)-ethyl-3-(3-(2-(2-cyano-*N*-methylbenzamido)ethyl)-5-(trifluoromethyl) phenyl)acrylate**: The corresponding reaction was run with standard conditions at 90 ^{o}C for 48 h using HFIP as the sole solvent. The two rotamers' ratio is about 65:35. ^{1}H NMR (400 MHz, $CDCl_3$) δ 7.80-7.60 (m, 3.97H), 7.58 (d, *J* = 3.1 Hz, 0.70H), 7.55-7.45 (m, 1.61H), 7.39 (d, *J* = 7.7 Hz, 0.76H), 7.25 (s, 0.32H), 7.15 (s, 0.33H), 6.95-6.88 (m, 0.30H), 6.53 (d, *J* = 16.1 Hz, 0.59H), 6.43 (d, *J* = 16.0 Hz, 0.34H), 4.34-4.22 (m, 2.00H), 3.89-3.78 (m, 1.30H), 3.51 (t, *J* = 7.0 Hz, 0.71H), 3.22 (s, 1.03H), 3.14 (dd, *J* = 8.8, 6.6 Hz, 1.31H), 2.95-2.83 (m, 2.74H), 1.40-1.30 (m, 3.00H); ^{13}C NMR (101 MHz, $CDCl_3$) δ 167.9, 167.8, 166.5, 166.3, 142.6, 142.2, 140.7, 140.4, 140.1, 139.6, 136.0, 135.8, 133.2, 133.0, 133.0, 132.9, 131.9, 131.7, 131.6, 131.5, 131.3, 129.7, 129.6, 129.0, 127.4, 127.4, 127.1 (d, $J_{C\text{-}F}$ = 3.7 Hz), 126.7 (q, $J_{C\text{-}F}$ = 3.6 Hz), 125.6, 123.8

(d, $J_{C\text{-}F}$ = 273.7 Hz), 123.6 (d, $J_{C\text{-}F}$ = 272.7 Hz), 123.1 (q, $J_{C\text{-}F}$ = 4.1 Hz), 122.95 (q, $J_{C\text{-}F}$ = 3.6 Hz), 120.9, 120.6, 116.8, 116.7, 110.0, 109.9, 60.9, 60.8, 52.1, 49.1, 37.4, 34.3, 33.0, 33.0, 14.3; HRMS (m/z, ESI-TOF): Calcd for $C_{23}H_{22}F_3N_2O_3^+$ [M+H$^+$] 431.1577, found 431.1586.

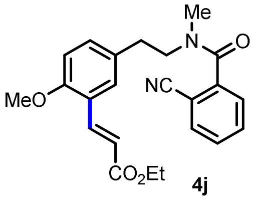

4j **(*E*)-ethyl-3-(5-(2-(2-cyano-*N*-methylbenzamido)ethyl)-2-methoxyphenyl) acrylate**: The corresponding reaction was run with standard conditions and DMF (5 equiv) for 48 h. The two rotamers' ratio is about 55:45. ^{1}H NMR (400 MHz, CDCl$_3$) δ 7.97 (d, *J* = 16.2 Hz, 0.54H), 7.86 (d, *J* = 16.2 Hz, 0.45H), 7.71 (d, *J* = 7.8 Hz, 0.56H), 7.69-7.59 (m, 1.03H), 7.55-7.41 (m, 2.09H), 7.37 (d, *J* = 7.7 Hz, 0.54H), 7.32 (d, *J* = 8.4 Hz, 0.47H), 7.00-6.78 (m, 2.42H), 6.56 (d, *J* = 16.2 Hz, 0.54H), 6.36 (d, *J* = 16.2 Hz, 0.45H), 4.26 (q, *J* = 7.1 Hz, 2.06H), 3.88 (s, 3.03H), 3.78 (dd, *J* = 8.8, 6.5 Hz, 1.10H), 3.43 (t, *J* = 6.9 Hz, 0.90H), 3.19 (s, 1.40H), 2.99 (t, *J* = 7.6 Hz, 1.10H), 2.84 (s, 1.60H), 2.77 (t, *J* = 6.9 Hz, 0.90H), 1.34 (td, *J* = 7.1, 3.0 Hz, 3.03H); ^{13}C NMR (101 MHz, CDCl$_3$) δ 168.0, 167.6, 167.6, 167.4, 157.4, 157.2, 140.7, 140.3, 139.9, 139.6, 133.2, 133.0, 132.9, 132.8, 132.0, 131.8, 130.9, 129.7, 129.5, 129.4, 129.3, 129.1, 127.7, 127.4, 123.8, 123.6, 119.4, 119.1, 116.9, 116.8, 111.6, 111.6, 110.1, 109.9, 60.5, 60.4, 55.8, 55.7, 52.7, 49.6, 37.4, 33.6, 33.0, 32.5, 14.5; HRMS (m/z, ESI-TOF): Calcd for $C_{23}H_{25}N_2O_4^+$ [M+H$^+$] 393.1809, found 393.1804.

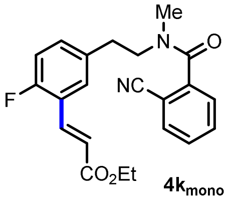

4k$_{mono}$ **(*E*)-ethyl-3-(5-(2-(2-cyano-*N*-methylbenzamido)ethyl)-2-fluorophenyl) acrylate**: The corresponding reaction was run with standard conditions at 90 °C for 36 h. The two rotamers' ratio is about 3:2. ^{1}H NMR (400 MHz, CDCl$_3$) δ 7.79 (d, *J* = 16.2 Hz, 0.58H), 7.75-7.58 (m, 2.00H), 7.56-7.42 (m, 2.00H), 7.41-7.29 (m, 1.20H), 7.11-6.90 (m, 2.20H), 6.57 (d, *J* = 16.2 Hz, 0.57H), 6.41 (d, *J* = 16.2 Hz, 0.38H), 4.27 (q, *J* = 7.0 Hz, 2.00H), 3.80 (t, *J* = 7.4 Hz, 1.21H), 3.45 (t, *J* = 6.6 Hz, 0.77H), 3.20 (s, 1.19H), 3.03 (t, *J* = 7.3 Hz, 1.25H), 2.91-2.75 (m, 2.66H), 1.34 (t, *J* = 6.9 Hz, 3.08H); ^{13}C NMR (101 MHz, CDCl$_3$) δ 167.9, 167.7, 166.9, 166.6, 160.3 (d, $J_{C\text{-}F}$ = 254 Hz), 160.3 (d, $J_{C\text{-}F}$ = 254 Hz), 140.5, 140.2, 137.1, 136.7, 135.0 (d, $J_{C\text{-}F}$ = 4 Hz), 134.0 (d, $J_{C\text{-}F}$ = 4 Hz), 133.2, 133.0, 133.0, 132.8, 132.2 (d, $J_{C\text{-}F}$ = 9 Hz), 131.9 (d, $J_{C\text{-}F}$ = 9 Hz), 129.6, 129.5, 129.33 (d, $J_{C\text{-}F}$ = 3 Hz), 129.14 (d, $J_{C\text{-}F}$ = 3 Hz), 127.5, 127.4, 122.8 (d, $J_{C\text{-}F}$ = 12 Hz), 122.5 (d, $J_{C\text{-}F}$ = 12 Hz), 121.4 (d, $J_{C\text{-}F}$ = 7 Hz), 121.1 (d, $J_{C\text{-}F}$ = 7 Hz), 116.8, 116.7 (d, $J_{C\text{-}F}$ = 5 Hz), 116.5 (d, $J_{C\text{-}F}$ = 3 Hz), 116.3, 110.0, 109.9, 60.8, 60.7, 52.5, 49.4, 37.4, 33.7, 33.0, 32.5, 14.4; HRMS (m/z, ESI-TOF): Calcd for $C_{22}H_{22}FN_2O_3^+$ [M+H$^+$] 381.1609, found 381.1605.

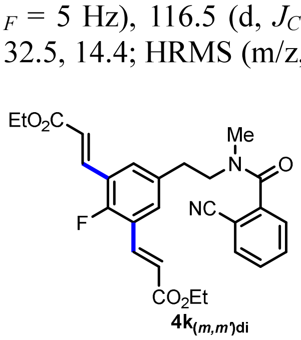

4k$_{(m,m')di}$ **(2*E*,2'*E*)-diethyl-3,3'-(5-(2-(2-cyano-*N*-methylbenzamido)ethyl)-2-fluoro-1,3-phenylene)diacrylate**: The two rotamers' ratio is about 2:1. ^{1}H NMR (400 MHz,

$CDCl_3$) δ 7.82 (d, *J* = 16.2 Hz, 1.30H), 7.77-7.61 (m, 2.50H), 7.57-7.46 (m, 2.75H), 7.39 (d, *J* = 7.7 Hz, 0.70H), 7.08 (d, *J* = 6.4 Hz, 0.70H), 7.01 (d, *J* = 7.3 Hz, 0.33H), 6.58 (d, *J* = 16.2 Hz, 1.27H), 6.42 (d, *J* = 16.2 Hz, 0.64H), 4.34-4.24 (m, 4.11H), 3.81 (t, *J* = 7.6 Hz, 1.34H), 3.49 (t, *J* = 6.9 Hz, 0.66H), 3.21 (s, 1.05H), 3.05 (t, *J* = 7.5 Hz, 1.37H), 2.92-2.80 (m, 2.84H), 1.40-1.31 (m, 6.21H); ^{13}C NMR (101 MHz, $CDCl_3$) δ 167.9, 167.8, 166.7, 166.5, 158.6 (d, $J_{C\text{-}F}$ = 263 Hz), 158.5 (d, $J_{C\text{-}F}$ = 262 Hz), 140.5, 140.2, 136.4 (d, $J_{C\text{-}F}$ = 4 Hz), 136.0 (d, $J_{C\text{-}F}$ = 3 Hz), 135.2 (d, $J_{C\text{-}F}$ = 4 Hz), 134.1 (d, $J_{C\text{-}F}$ = 3 Hz), 133.3, 133.1, 133.0, 133.0, 130.7 (d, $J_{C\text{-}F}$ = 3 Hz), 130.3 (d, $J_{C\text{-}F}$ = 3 Hz), 129.7, 127.7, 127.4, 123.8 (d, $J_{C\text{-}F}$ = 12 Hz), 123.6 (d, $J_{C\text{-}F}$ = 13 Hz), 122.3 (d, $J_{C\text{-}F}$ = 7 Hz), 122.0 (d, $J_{C\text{-}F}$ = 6 Hz), 116.9, 116.8, 110.0, 61.0, 60.8, 52.3, 49.3, 37.4, 33.9, 33.1, 32.6, 14.4; HRMS (m/z, ESI-TOF): Calcd for $C_{27}H_{28}FN_2O_5^+$ [M+H$^+$] 479.1977, found 479.1966.

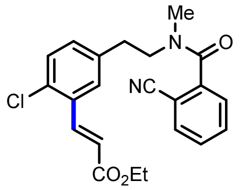

4l$_{mono}$

(*E*)-ethyl-3-(2-chloro-5-(2-(2-cyano-*N*-methylbenzamido)ethyl)phenyl) acrylate: The corresponding reaction was run with standard conditions at 90 °C for 36 h. The NMR sample contains about 6% isomer. The two rotamers' ratio is about 5:3. ^{1}H NMR (400 MHz, $CDCl_3$) δ 8.06 (d, *J* = 16.0 Hz, 0.57H), 7.99 (d, *J* = 16.0 Hz, 0.36H), 7.77-7.60 (m, 1.76H), 7.58 (s, 0.65H), 7.55-7.45 (m, 1.44H), 7.38 (d, *J* = 8.7 Hz, 1.21H), 7.34-7.25 (m, 1.23H), 7.13 (s, 0.36H), 6.92 (t, *J* = 9.7 Hz, 0.75H), 6.49 (d, *J* = 16.0 Hz, 0.57H), 6.24 (d, *J* = 16.0 Hz, 0.33H), 4.28 (q, *J* = 6.8 Hz, 2.02H), 3.81 (t, *J* = 7.5 Hz, 1.25H), 3.46 (t, *J* = 6.8 Hz, 0.75H), 3.20 (s, 1.16H), 3.04 (t, *J* = 7.5 Hz, 1.25H), 2.86 (s, 1.83H), 2.82 (t, *J* = 6.6 Hz, 0.83H), 1.39-1.30 (m, 3.03H); ^{13}C NMR (101 MHz, $CDCl_3$) δ 167.9, 167.7, 166.6, 166.3, 140.5, 140.2, 140.1, 139.9, 138.0, 136.9, 133.5, 133.2, 133.2, 133.1, 133.0, 132.9, 132.8, 131.7, 131.4, 130.5, 130.4, 129.6, 129.5, 128.1, 127.9, 127.6, 127.4, 121.4, 121.2, 116.8, 116.8, 110.0, 109.9, 60.8, 60.7, 52.4, 49.2, 37.4, 34.0, 33.1, 32.7, 14.4; HRMS (m/z, ESI-TOF): Calcd for $C_{22}H_{22}ClN_2O_3^+$ [M+H$^+$] 397.1313, found 397.1309.

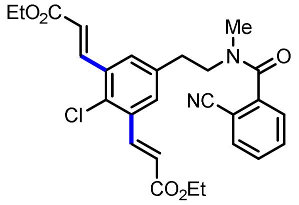

4l$_{(m,m')di}$

(2*E*,2'*E*)-diethyl-3,3'-(2-chloro-5-(2-(2-cyano-*N*-methylbenzamido) ethyl)-1,3-phenylene)diacrylate: The two rotamers' ratio is about 65:35. ^{1}H NMR (400 MHz, $CDCl_3$) δ 8.11 (d, *J* = 16.0 Hz, 1.23H), 8.04 (d, *J* = 16.0 Hz, 0.63H), 7.76-7.63 (m, 1.88H), 7.60 (s, 1.23H), 7.56-7.47 (m, 1.38H), 7.39 (d, *J* = 7.6 Hz, 0.71H), 7.15 (s, 0.65H), 6.98 (d, *J* = 7.3 Hz, 0.30H), 6.47 (d, *J* = 16.0 Hz, 1.29H), 6.24 (d, *J* = 16.0 Hz, 0.67H), 4.29 (q, *J* = 7.1 Hz, 4.00H), 3.83 (t, *J* = 7.6 Hz, 1.30H), 3.49 (t, *J* = 6.8 Hz, 0.70H), 3.22 (s, 1.00H), 3.06 (t, *J* = 7.7 Hz, 1.30H), 2.90 (s, 2.00H), 2.84 (t, *J* = 7.2 Hz, 0.73H), 1.41-1.31 (m, 6.02H); ^{13}C NMR (101 MHz, $CDCl_3$) δ 167.9, 167.8, 166.4, 166.2, 140.4, 140.4, 140.1, 140.0, 137.9, 136.9, 134.6, 134.4, 133.6, 133.4, 133.3, 133.1, 133.0, 132.9, 129.7, 129.5, 129.1, 127.7, 127.4, 122.4, 122.2, 116.8, 116.8, 110.0, 110.0, 61.0, 60.9, 52.3, 49.1, 37.4, 34.1, 33.1, 32.8, 14.4; HRMS (m/z, ESI-TOF): Calcd for $C_{27}H_{28}ClN_2O_5^+$ [M+H$^+$] 495.1681, found 495.1669.

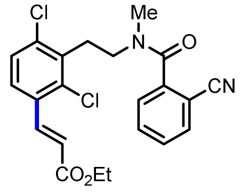

$4m_{mono}$ **(*E*)-ethyl-3-(2,4-dichloro-3-(2-(2-cyano-*N*-methylbenzamido)ethyl)phenyl) acrylate**: The corresponding reaction was run with standard conditions at 90 °C for 48 h using HFIP as the sole solvent. The two rotamers' ratio is about 62:38. ^{1}H NMR (400 MHz, $CDCl_3$) δ 8.08 (d, *J* = 16.0 Hz, 0.36H), 7.90 (d, *J* = 16.0 Hz, 0.59H), 7.75-7.63 (m, 1.45H), 7.58 (td, *J* = 7.7, 1.4 Hz, 0.65H), 7.55-7.38 (m, 2.47H), 7.35 (d, *J* = 8.4 Hz, 0.40H), 7.32-7.21 (m, 1.34H), 6.44-6.30 (m, 0.97H), 4.33-4.22 (m, 2.00H), 3.82 (dd, *J* = 8.7, 6.3 Hz, 0.79H), 3.50-3.36 (m, 2H), 3.30 (s, 1.90H), 3.20 (dd, *J* = 9.4, 6.2 Hz, 1.30H), 2.96 (s, 1.16H), 1.39-1.30 (m, 3.03H); ^{13}C NMR (101 MHz, $CDCl_3$) δ 167.8, 167.6, 166.2, 166.1, 140.5, 140.2, 139.7, 137.1, 136.8, 136.1, 135.8, 135.8, 134.2, 133.1, 132.9, 132.9, 132.9, 132.6, 132.4, 129.5, 129.4, 128.3, 128.3, 127.4, 126.7, 126.5, 122.0, 121.7, 116.8, 110.1, 109.9, 60.8, 60.8, 48.6, 45.9, 37.1, 33.3, 30.6, 29.3, 14.3; HRMS (m/z, ESI-TOF): Calcd for $C_{22}H_{21}Cl_2N_2O_3^+$ [M+H$^+$] 431.0924, found 431.0934.

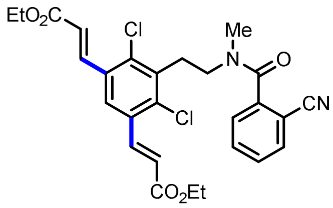

$4m_{(m,m')di}$ **(2*E*,2'*E*)-diethyl-3,3'-(4,6-dichloro-5-(2-(2-cyano-*N*-methylbenz amido)ethyl)-1,3-phenylene)diacrylate**: The two rotamers' ratio is about 60:40. ^{1}H NMR (400 MHz, $CDCl_3$) δ 8.04 (d, *J* = 15.9 Hz, 0.74H), 7.88 (d, *J* = 15.9 Hz, 1.11H), 7.73 (s, 0.40H), 7.71-7.61 (m, 2.12H), 7.57 (t, *J* = 7.4 Hz, 0.63H), 7.49 (t, *J* = 7.6 Hz, 1.06H), 7.43 (d, *J* = 7.8 Hz, 0.40H), 7.29 (d, *J* = 7.6 Hz, 0.58H), 6.46-6.33 (m, 1.80H), 4.32-4.19 (m, 4.00H), 3.80 (dd, *J* = 8.9, 6.2 Hz, 0.80H), 3.47 (dd, *J* = 8.8, 6.3 Hz, 0.80H), 3.39 (dd, *J* = 9.7, 6.2 Hz, 1.20H), 3.28 (s, 1.80H), 3.23 (dd, *J* = 9.9, 6.0 Hz, 1.20H), 2.96 (s, 1.20H), 1.40-1.30 (m, 6.04H); ^{13}C NMR (101 MHz, $CDCl_3$) δ 167.9, 167.7, 166.1, 165.9, 140.5, 140.3, 140.0, 139.5, 137.1, 136.9, 136.7, 135.3, 133.2, 133.0, 133.0, 132.8, 132.6, 129.6, 127.5, 124.8, 124.6, 122.8, 122.5, 116.8, 116.8, 110.3, 110.0, 61.0, 61.0, 48.5, 45.8, 37.2, 33.4, 31.3, 29.9, 14.3; HRMS (m/z, ESI-TOF): Calcd for $C_{27}H_{27}Cl_2N_2O_5^+$ [M+H$^+$] 529.1392, found 529.1297.

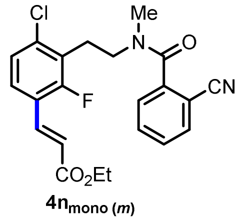

$4n_{mono\ (m)}$ **(*E*)-ethyl-3-(4-chloro-3-(2-(2-cyano-*N*-methylbenzamido)ethyl)-2-fluoro phenyl)acrylate**: The corresponding reaction was run with standard conditions and ethyl acrylate (2.5 equiv) at 90 °C for 48 h using HFIP as the sole solvent. The two rotamers' ratio is about 56:44. ^{1}H NMR (400 MHz, $CDCl_3$) δ 7.78 (d, *J* = 16.3 Hz, 0.43H), 7.73-7.59 (m, 1.98H), 7.59-7.45 (m, 1.73H), 7.44-7.32 (m, 1.39H), 7.23 (d, *J* = 8.2 Hz, 0.42H), 7.14-7.06 (m, 1.04H), 6.52 (d, *J* = 16.2 Hz, 0.41H), 6.44 (d, *J* = 16.1 Hz, 0.52H), 4.28 (q, *J* = 7.0 Hz, 2.00H), 3.83 (t, *J* = 6.9 Hz, 0.89H), 3.45 (t, *J* = 7.0 Hz, 1.17H), 3.33-3.21 (m, 2.61H), 3.04 (t, *J* = 7.0 Hz, 1.16H), 2.93 (s, 1.31H), 1.34 (t, *J* = 7.0 Hz, 3.02H); ^{13}C NMR (101 MHz, $CDCl_3$) δ 167.9, 167.6, 166.6,

166.4, 160.1 (d, J_{C-F} = 257 Hz), 159.8 (d, J_{C-F} = 256 Hz), 140.5, 140.1, 137.2 (d, J_{C-F} = 6 Hz), 136.9 (d, J_{C-F} = 6 Hz), 136.2 (d, J_{C-F} = 3 Hz), 135.7 (d, J_{C-F} = 3 Hz), 133.1, 133.0, 132.9, 132.8, 129.5, 129.5, 127.6 (d, J_{C-F} = 4 Hz), 127.4 (d, J_{C-F} = 3 Hz), 127.4, 125.7, 125.7, 125.6, 125.6, 125.5, 124.14 (d, J_{C-F} = 19 Hz), 121.71 (d, J_{C-F} = 6 Hz), 121.58 (d, J_{C-F} = 14 Hz), 121.4, 121.3 (d, J_{C-F} = 7 Hz), 116.8, 116.7, 110.0, 110.0, 60.8, 60.8, 49.3, 46.4, 37.1, 33.2, 25.6 (d, J_{C-F} = 4 Hz), 24.4 (d, J_{C-F} = 6 Hz), 14.3; HRMS (m/z, ESI-TOF): Calcd for $C_{22}H_{21}ClFN_2O_3^+$ [M+H$^+$] 415.1219, found 415.1227.

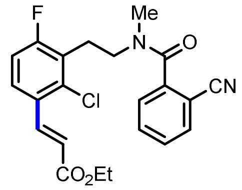

$4n_{mono\ (m')}$ **(*E*)-ethyl-3-(2-chloro-3-(2-(2-cyano-*N*-methylbenzamido)ethyl)-4-fluoro phenyl)acrylate**: The two rotamers' ratio is about 62:38. ^{1}H NMR (400 MHz, $CDCl_3$) δ 8.06 (d, *J* = 16.0 Hz, 0.35H), 7.87 (d, *J* = 15.9 Hz, 0.57H), 7.75-7.58 (m, 1.60H), 7.55-7.43 (m, 2.63H), 7.40 (d, *J* = 7.7 Hz, 0.40H), 7.08-6.93 (m, 1.50H), 6.39-6.27 (m, 0.90H), 4.26 (q, *J* = 7.1 Hz, 2.00H), 3.81 (t, *J* = 7.2 Hz, 0.84H), 3.45 (t, *J* = 7.2 Hz, 1.20H), 3.33-3.19 (m, 2.72H), 3.07-2.99 (m, 1.21H), 2.90 (s, 1.20H), 1.32 (t, *J* = 7.1 Hz, 3.03H); ^{13}C NMR (101 MHz, $CDCl_3$) δ 167.9, 167.7, 166.4, 166.3, 162.4 (d, J_{C-F} = 254 Hz), 162.2 (d, J_{C-F} = 254 Hz), 140.5, 140.2, 140.1, 139.7, 136.2 (d, J_{C-F} = 6 Hz), 136.0 (d, J_{C-F} = 5 Hz), 133.2, 133.0, 132.9, 132.8, 130.0 (d, J_{C-F} = 4 Hz), 129.9 (d, J_{C-F} = 4 Hz), 129.6, 129.5, 127.5, 127.4, 127.3 (d, J_{C-F} = 10.0 Hz), 127.1 (d, J_{C-F} = 10 Hz), 125.8 (d, J_{C-F} = 19 Hz), 124.2 (d, J_{C-F} = 19 Hz), 121.3 (d, J_{C-F} = 2 Hz), 121.0 (d, J_{C-F} = 2 Hz), 116.8, 116.7, 114.7 (d, J_{C-F} = 22 Hz), 114.5 (d, J_{C-F} = 22 Hz), 110.1, 110.0, 60.9, 60.8, 49.1, 46.5, 37.2, 33.2, 25.7 (d, J_{C-F} = 2 Hz), 24.7 (d, J_{C-F} = 2 Hz), 14.4; HRMS (m/z, ESI-TOF): Calcd for $C_{22}H_{21}ClFN_2O_3^+$ [M+H$^+$] 415.1219, found 415.1227.

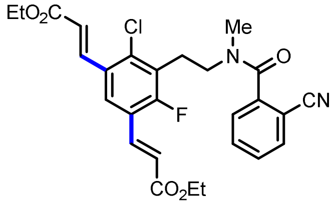

$4n_{(m,m')di}$ **(2*E*,2'*E*)-diethyl-3,3'-(4-chloro-5-(2-(2-cyano-*N*-methylbenzamido) ethyl)-6-fluoro-1,3-phenylene)diacrylate**: The two rotamers' ratio is about 56:44. ^{1}H NMR (400 MHz, $CDCl_3$) δ 8.06 (d, *J* = 15.9 Hz, 0.44H), 7.88 (d, *J* = 15.9 Hz, 0.56H), 7.77 (d, *J* = 16.2 Hz, 0.54H), 7.74-7.61 (m, 2.87H), 7.59-7.45 (m, 1.66H), 7.40 (d, *J* = 7.6 Hz, 0.45H), 7.13-7.06 (m, 0.54H), 6.58 (d, *J* = 16.2 Hz, 0.44H), 6.51 (d, *J* = 16.2 Hz, 0.50H), 6.45-6.33 (m, 0.93H), 4.40-4.20 (m, 4.00H), 3.83 (t, *J* = 7.2 Hz, 0.90H), 3.47 (t, *J* = 7.3 Hz, 1.10H), 3.37-3.23 (m, 2.55H), 3.08 (t, *J* = 6.5 Hz, 1.11H), 2.95 (s, 1.35H), 1.35 (t, *J* = 7.1 Hz, 6.06H); ^{13}C NMR (101 MHz, $CDCl_3$) δ 167.8, 167.7, 166.3, 166.1, 166.0, 160.4 (d, J_{C-F} = 260 Hz), 160.1 (d, J_{C-F} = 260 Hz), 140.3, 140.0, 139.6, 139.1, 137.3 (d, J_{C-F} = 6 Hz), 136.9 (d, J_{C-F} = 6 Hz), 135.7 (d, J_{C-F} = 2 Hz), 135.2 (d, J_{C-F} = 2 Hz), 133.1, 133.0, 132.9, 132.9, 130.9, 130.2 (d, J_{C-F} = 4 Hz), 130.1 (d, J_{C-F} = 4 Hz), 129.6, 129.5, 128.8, 127.4, 127.3, 126.5 (d, J_{C-F} = 19 Hz), 125.9 (d, J_{C-F} = 4 Hz), 125.8 (d, J_{C-F} = 4 Hz), 124.9 (d, J_{C-F} = 19 Hz), 122.6 (d, J_{C-F} = 6 Hz), 122.3 (d, J_{C-F} = 6 Hz), 122.1, 122.0, 121.8, 121.7, 121.6, 116.7, 116.7, 110.1, 110.0, 60.9, 60.8, 49.0, 46.3, 37.1, 33.1, 25.9 (d, J_{C-F} = 2 Hz), 24.7 (d, J_{C-F} = 2 Hz), 14.3; HRMS (m/z, ESI-TOF): Calcd for $C_{27}H_{27}ClFN_2O_5^+$ [M+H$^+$] 513.1587, found 513.1593.

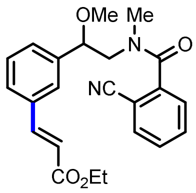

$4o_{mono}$ **(*E*)-ethyl-3-(3-(2-(2-cyano-*N*-methylbenzamido)-1-methoxyethyl) phenyl) acrylate**: The corresponding reaction was run with standard conditions and DMF (5 equiv) for 48 h. The two rotamers' ratio is about 62:38. ^{1}H NMR (400 MHz, $CDCl_3$) δ 7.80-7.59 (m, 3.35H), 7.60-7.37 (m, 4.62H), 7.33 (t, *J* = 7.7 Hz, 0.40H), 7.19 (d, *J* = 7.5 Hz, 0.38H), 7.13 (s, 0.35H), 7.06 (d, *J* = 7.4 Hz, 0.36H), 6.50 (d, *J* = 16.0 Hz, 0.58H), 6.35 (d, *J* = 16.0 Hz, 0.36H), 4.74 (dd, *J* = 8.6, 3.4 Hz, 0.60H), 4.39-4.22 (m, 2.42H), 3.99 (dd, *J* = 13.7, 3.5 Hz, 0.60H), 3.55-3.30 (m, 3.45H), 3.30-3.18 (m, 2.50H), 2.97 (s, 2.00H), 1.34 (t, *J* = 7.1 Hz, 3.06H); ^{13}C NMR (101 MHz, $CDCl_3$) δ 168.7, 168.0, 167.0, 166.8, 144.3, 143.8, 140.7, 140.5, 140.5, 139.6, 135.2, 135.0, 133.1, 133.0, 132.9, 132.6, 129.5, 129.5, 129.4, 129.3, 128.7, 128.4, 128.4, 128.1, 127.9, 127.2, 126.1, 125.8, 119.1, 118.9, 116.9, 116.8, 110.5, 110.1, 82.1, 81.0, 60.7, 60.6, 57.4, 57.3, 57.2, 54.9, 39.3, 34.1, 14.4; HRMS (m/z, ESI-TOF): Calcd for $C_{23}H_{25}N_2O_4^+$ [M+H$^+$] 393.1809, found 393.1807.

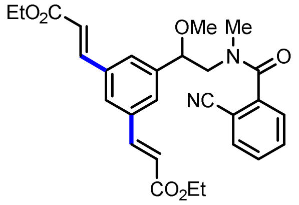

$4o_{(m,m')di}$ **(2*E*,2'*E*)-diethyl-3,3'-(5-(2-(2-cyano-*N*-methylbenzamido)-1-methoxy ethyl)-1,3-phenylene)diacrylate**: The two rotamers' ratio is about 68:32. ^{1}H NMR (400 MHz, $CDCl_3$) δ 7.87-7.46 (m, 7.32H), 7.41 (d, *J* = 7.7 Hz, 0.68H), 7.24 (d, *J* = 7.6 Hz, 0.34H), 7.15 (s, 0.56H), 6.53 (d, *J* = 16.0 Hz, 1.29H), 6.38 (d, *J* = 16.0 Hz, 0.64H), 4.77 (d, *J* = 6.5 Hz, 0.68H), 4.46-4.17 (m, 4.32H), 4.03 (d, *J* = 12.7 Hz, 0.66H), 3.53-3.31 (m, 3.41H), 3.26 (s, 2.00H), 3.11-2.93 (m, 2.06H), 1.35 (t, *J* = 7.0 Hz, 6.07H); ^{13}C NMR (101 MHz, $CDCl_3$) δ 168.7, 168.0, 166.7, 166.5, 143.4, 143.0, 141.5, 140.6, 140.5, 140.4, 136.0, 135.8, 133.2, 133.1, 132.7, 129.6, 129.5, 128.1, 127.5, 127.3, 127.2, 120.2, 119.9, 116.9, 116.9, 110.6, 110.1, 82.1, 81.0, 60.8, 60.7, 57.5, 57.4, 55.0, 54.9, 39.4, 34.2, 14.4; HRMS (m/z, ESI-TOF): Calcd for $C_{28}H_{31}N_2O_6^+$ [M+H$^+$] 491.2177, found 491.2166.

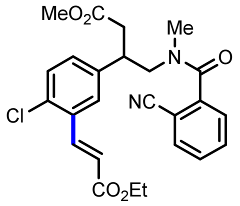

$4p_{mono}$ **(*E*)-methyl-3-(4-chloro-3-(3-ethoxy-3-oxoprop-1-enyl)phenyl)-4-(2-cyano-*N*-methylbenzamido)butanoate**: The corresponding reaction was run with standard conditions at 90 °C for 48 h. The two rotamers' ratio is about 74:26. ^{1}H NMR (400 MHz, $CDCl_3$) δ 8.06 (d, *J* = 16.0 Hz, 0.68H), 7.99 (d, *J* = 16.0 Hz, 0.24H), 7.80-7.55 (m, 2.72H), 7.53-7.44 (m, 1.33H), 7.40 (d, *J* = 8.3 Hz, 0.68H), 7.32 (dd, *J* = 8.3, 2.1 Hz, 0.94H), 7.17 (d, *J* = 7.7 Hz, 0.67H), 7.10 (d, *J* = 1.7 Hz, 0.24H), 6.93 (dd, *J* = 8.2, 1.8 Hz, 0.25H), 6.81 (brs, 0.22H), 6.51 (d, *J* = 16.0 Hz, 0.67H), 6.20 (d, *J* = 16.0 Hz, 0.25H), 4.36-4.23 (m, 2.00H), 3.97 (dd, *J* = 13.0, 6.9 Hz,

0.68H), 3.83-3.36 (m, 5.49H), 3.16 (s, 0.78H), 2.88 (dd, J = 16.3, 5.8 Hz, 0.74H), 2.84-2.69 (m, 2.82H), 2.59-2.44 (m, 0.62H), 1.42-1.31 (m, 3.08H); ^{13}C NMR (101 MHz, $CDCl_3$) δ 172.0, 171.3, 168.2, 167.9, 166.6, 166.3, 140.4, 140.3, 140.2, 139.8, 139.6, 139.5, 134.2, 133.8, 133.4, 133.2, 133.1, 133.1, 133.0, 132.7, 130.8, 130.6, 130.5, 129.7, 129.6, 128.2, 127.3, 127.3, 126.5, 121.8, 121.5, 116.9, 116.7, 110.0, 60.9, 60.8, 56.4, 52.1, 52.1, 51.9, 40.0, 39.7, 38.3, 37.4, 33.6, 14.4; HRMS (m/z, ESI-TOF): Calcd for $C_{25}H_{26}ClN_2O_5^+$ [M+H$^+$] 469.1525, found 469.1530.

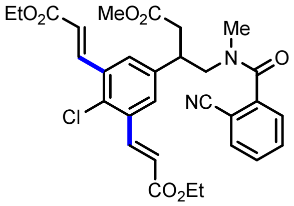

4p$_{(m,m')di}$ **(2E,2'E)-diethyl-3,3'-(2-chloro-5-(1-(2-cyano-N-methylbenzamido)-4-methoxy-4-oxobutan-2-yl)-1,3-phenylene)diacrylate**: The two rotamers' ratio is about 77:23. ^{1}H NMR (400 MHz, $CDCl_3$) δ 8.11 (d, J = 16.0 Hz, 1.44H), 8.05 (d, J = 16.0 Hz, 0.44H), 7.72-7.56 (m, 3.41H), 7.53-7.42 (m, 1.36H), 7.17 (d, J = 7.7 Hz, 0.72H), 7.14 (s, 0.43H), 6.81 (s, 0.18H), 6.49 (d, J = 16.0 Hz, 1.42H), 6.21 (d, J = 16.0 Hz, 0.45H), 4.30-4.23 (m, 4.00H), 3.94 (dd, J = 13.5, 7.5 Hz, 0.71H), 3.85 (dd, J = 13.6, 8.1 Hz, 0.71H), 3.77-3.41 (m, 5.11H), 3.18 (s, 0.72H), 2.90 (dd, J = 16.3, 5.7 Hz, 0.76H), 2.82 (s, 2.05H), 2.75 (dd, J = 16.3, 8.8 Hz, 1.06H), 2.52 (dd, J = 12.9, 6.5 Hz, 0.45H), 1.41-1.32 (m, 6.15H); HRMS (m/z, ESI-TOF): ^{13}C NMR (101 MHz, $CDCl_3$) δ 171.9, 171.1, 168.3, 167.9, 166.4, 166.1, 140.3, 140.2, 139.9, 139.5, 139.5, 134.9, 134.5, 134.2, 134.0, 133.3, 133.1, 132.8, 129.7, 128.6, 128.2, 128.0, 127.3, 122.7, 122.5, 116.9, 116.7, 109.9, 61.1, 60.9, 56.3, 52.2, 52.0, 51.8, 40.1, 39.7, 38.3, 37.4, 37.4, 33.7, 14.4; Calcd for $C_{30}H_{32}ClN_2O_7^+$ [M+H$^+$] 567.1893, found 567.1897.

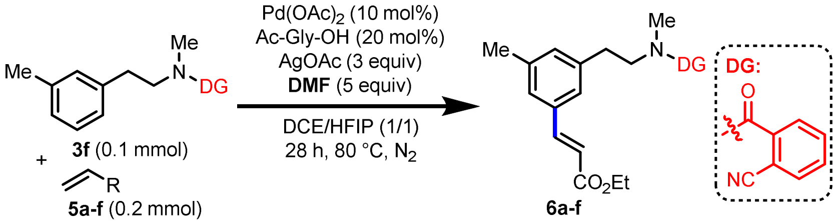

A 50 mL Schlenk sealed tube (with a Teflon cap) equipped with a magnetic stir bar was charged with amide **3f** (0.10 mmol, 1.0 equiv), $Pd(OAc)_2$ (2.3 mg, 0.010 mmol, 10 mol%), Ac-Gly-OH (2.4 mg, 0.020 mmol, 20 mol%) and AgOAc (50 mg, 0.30 mmol, 3 equiv). The tube was then purged with nitrogen. DCE (0.5 mL) was added to the mixture, followed by olefin **5** (2.0 equiv) and DMF (39 μL, 0.5 mmol, 5 equiv). And then 0.5 mL of HFIP was added along the inside wall of the tube. The tube was then capped under a flow of nitrogen and submerged into a preheated 80 °C oil bath. The reaction was stirred for 28 h and cooled to room temperature. The crude reaction mixture was diluted with EtOAc (5 mL) and filtered through a short pad of Celite. The sealed tube and Celite pad were washed with an additional 20 mL of EtOAc. The filtrate was concentrated *in vacuo*, and the resulting residue was purified by preparative thin layer chromatography using petroleum ether/EtOAc as the eluent. The site selectivity was assigned by ^{1}H NMR analysis of the product.

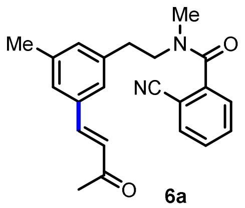

6a **(*E*)-2-cyano-*N*-methyl-*N*-(3-methyl-5-(3-oxobut-1-enyl)phenethyl) benzamide**: The corresponding reaction was run with standard conditions and DMF (5 equiv) for 28 h. The two rotamers' ratio is about 57:43. ^{1}H NMR (400 MHz, $CDCl_3$) δ 7.72 (d, *J* = 7.7 Hz, 0.61H), 7.70-7.60 (m, 1.10H), 7.54-7.43 (m, 2.24H), 7.42-7.34 (m, 1.00H), 7.32 (s, 0.56H), 7.24 (s, 0.94H), 7.20 (s, 0.54H), 6.89 (s, 0.44H), 6.87-6.81 (m, 0.44H), 6.79 (s, 0.43H), 6.74 (d, *J* = 16.3 Hz, 0.59H), 6.61 (d, *J* = 16.3 Hz, 0.43H), 3.80 (dd, *J* = 8.5, 6.9 Hz, 1.14H), 3.47 (t, *J* = 6.9 Hz, 0.86H), 3.20 (s, 1.33H), 3.07-2.99 (m, 1.15H), 2.86 (s, 1.72H), 2.80 (t, *J* = 6.9 Hz, 0.88H), 2.42-2.33 (m, 4.54H), 2.30 (s, 1.39H); ^{13}C NMR (101 MHz, $CDCl_3$) δ 198.6, 198.3, 168.0, 167.6, 143.6, 143.1, 140.6, 140.3, 139.6, 139.3, 139.1, 138.5, 135.0, 134.8, 133.2, 133.0, 132.8, 132.8, 132.2, 131.9, 129.6, 129.4, 127.7, 127.5, 127.4, 127.3, 127.2, 126.2, 125.9, 116.9, 116.8, 110.0, 109.9, 52.4, 49.5, 37.4, 34.3, 33.2, 33.0, 27.7, 21.3, 21.3; HRMS (m/z, ESI-TOF): Calcd for $C_{22}H_{23}N_2O_2^+$ [M+H$^+$] 347.1754, found 347.1765.

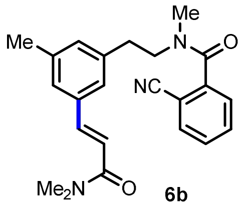

6b **(*E*)-2-cyano-*N*-(3-(3-(dimethylamino)-3-oxoprop-1-enyl)-5-methyl phenethyl)-*N*-methylbenzamide**: The corresponding reaction was run with standard conditions and DMF (5 equiv) for 28 h. The two rotamers' ratio is about 55:45. ^{1}H NMR (400 MHz, $CDCl_3$) δ 7.71 (d, *J* = 7.7 Hz, 0.56H), 7.68-7.58 (m, 1.53H), 7.57-7.41 (m, 1.97H), 7.37 (d, *J* = 7.7 Hz, 0.56H), 7.33 (s, 0.51H), 7.20 (d, *J* = 8.6 Hz, 0.95H), 7.14 (s, 0.51H), 6.93 (d, *J* = 15.4 Hz, 0.53H), 6.87-6.78 (m, 1.32H), 6.75 (s, 0.44H), 3.81 (t, *J* = 7.6 Hz, 1.11H), 3.46 (t, *J* = 6.8 Hz, 0.89H), 3.25-3.13 (m, 4.39H), 3.07 (s, 3.00H), 3.03 (dd, *J* = 8.6, 6.6 Hz, 1.18H), 2.83 (s, 1.65H), 2.79 (t, *J* = 6.8 Hz, 0.88H), 2.36 (s, 1.60H), 2.30 (s, 1.32H); ^{13}C NMR (101 MHz, $CDCl_3$) δ 168.0, 167.6, 166.9, 166.7, 142.4, 142.0, 140.7, 140.3, 139.3, 139.0, 138.9, 138.2, 136.0, 135.8, 133.2, 133.0, 132.9, 132.7, 131.1, 131.0, 129.5, 129.4, 127.7, 127.4, 127.4, 126.9, 125.6, 125.1, 117.9, 117.6, 116.9, 116.9, 110.1, 109.9, 52.5, 49.5, 37.6, 37.6, 37.6, 37.5, 36.0, 34.3, 33.3, 33.0, 21.3; HRMS (m/z, ESI-TOF): Calcd for $C_{23}H_{26}N_3O_2^+$ [M+H$^+$] 376.2020, found 376.2028.

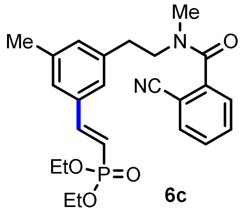

6c **(*E*)-diethyl-3-(2-(2-cyano-*N*-methylbenzamido)ethyl)-5-methylstyryl phosphonate**: The corresponding reaction was run with standard conditions and DMF (5 equiv) for 28 h. The two rotamers' ratio is about 56:44. ^{1}H NMR (400 MHz, $CDCl_3$) δ 7.71 (d, *J* = 7.8 Hz, 0.58H), 7.70-7.61 (m, 1.00H), 7.57-7.43 (m, 2.00H), 7.44-7.28 (m, 1.17H), 7.26 (s, 0.49H), 7.19 (s, 1.52H), 6.86 (d, *J* = 8.1 Hz, 0.45H), 6.81 (s, 0.42H), 6.78 (s, 0.41H), 6.28 (t, *J* = 17.7 Hz, 0.56H), 6.15 (t, *J* = 17.5 Hz, 0.44H), 4.20-4.07 (m, 4.01H), 3.87-3.75 (m, 1.10H), 3.46 (t, *J* = 6.9 Hz, 0.90H), 3.20 (s, 1.34H), 3.08-2.98 (m, 1.13H), 2.85 (s, 1.72H), 2.80 (t, *J* = 6.9 Hz, 0.88H), 2.36 (s, 1.70H), 2.30 (s, 1.34H), 1.44-1.30 (m, 6.08H); ^{13}C NMR (101 MHz, $CDCl_3$) δ 167.9,

167.6, 148.8 (d, $J_{C\text{-}P}$ = 7 Hz), 148.3 (d, $J_{C\text{-}P}$ = 7 Hz), 140.7, 140.3, 139.5, 139.2, 139.0, 138.3, 135.6, 135.4, 135.3, 135.1, 133.2, 133.0, 132.9, 132.8, 131.9, 131.6, 129.6, 129.4, 127.7, 127.4, 126.9, 125.4, 125.3, 116.9, 116.8, 115.5, 115.0, 113.6, 113.1, 110.0, 109.9, 61.9 (d, $J_{C\text{-}P}$ = 5 Hz), 61.9 (d, $J_{C\text{-}P}$ = 5 Hz), 52.4, 49.5, 37.4, 34.3, 33.2, 33.0, 21.3, 21.3, 16.5 (d, $J_{C\text{-}P}$ = 7 Hz); HRMS (m/z, ESI-TOF): Calcd for $C_{24}H_{30}N_2O_4P^+$ [M+H$^+$] 441.1938, found 441.1946.

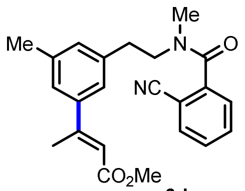

6d **(*E*)-methyl-3-(3-(2-(2-cyano-*N*-methylbenzamido)ethyl)-5-methylphenyl) but-2-enoate**: The corresponding reaction was run with standard conditions and DMF (5 equiv) for 28 h. The two rotamers' ratio is about 52:48. ^{1}H NMR (400 MHz, CDCl$_3$) δ 7.71 (d, *J* = 7.7 Hz, 0.56H), 7.69-7.61 (m, 0.99H), 7.55-7.42 (m, 1.65H), 7.39 (d, *J* = 7.7 Hz, 0.47H), 7.21 (s, 0.57H), 7.19-7.13 (m, 1.39H), 6.86-6.78 (m, 0.47H), 6.77 (d, *J* = 6.7 Hz, 0.82H), 6.14 (q, *J* = 1.4 Hz, 0.46H), 6.00 (q, *J* = 1.4 Hz, 0.42H), 3.84-3.74 (m, 4.16H), 3.45 (t, *J* = 6.9 Hz, 0.90H), 3.21 (s, 1.38H), 3.08-2.98 (m, 1.02H), 2.86 (s, 1.58H), 2.80 (t, *J* = 6.9 Hz, 1.04H), 2.57 (d, *J* = 0.9 Hz, 1.51H), 2.49 (d, *J* = 1.0 Hz, 1.45H), 2.37 (s, 1.56H), 2.30 (s, 1.44H); ^{13}C NMR (101 MHz, CDCl$_3$) δ 168.0, 167.6, 167.4, 167.2, 156.1, 155.6, 142.9, 142.6, 140.7, 140.4, 139.1, 138.8, 138.6, 138.0, 133.2, 133.0, 132.8, 132.7, 130.6, 130.4, 129.6, 129.3, 127.7, 127.5, 125.7, 125.5, 124.1, 124.0, 116.9, 116.9, 116.8, 116.7, 110.1, 109.9, 52.6, 51.3, 51.2, 49.6, 37.4, 34.5, 33.3, 33.0, 21.5, 21.4, 18.2, 18.1; HRMS (m/z, ESI-TOF): Calcd for $C_{23}H_{25}N_2O_3^+$ [M+H$^+$] 377.1860, found 377.1868.

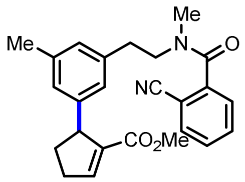

6e **Methyl-5-(3-(2-(2-isocyano-*N*-methylbenzamido)ethyl)-5-methylphenyl) cyclopent-1-ene-1-carboxylate**: The corresponding reaction was run with standard conditions and DMF (5 equiv) for 28 h. The two rotamers' ratio is about 50:50. ^{1}H NMR (400 MHz, CDCl$_3$) δ 7.70 (d, *J* = 7.7 Hz, 0.49H), 7.64 (t, *J* = 7.2 Hz, 1.00H), 7.53-7.38 (m, 1.55H), 7.37 (d, *J* = 7.7 Hz, 0.46H), 6.99 (t, *J* = 2.2 Hz, 0.93H), 6.96 (s, 0.50H), 6.90 (s, 0.49H), 6.84 (s, 0.93H), 6.80 (d, *J* = 7.3 Hz, 0.45H), 6.57 (s, 0.48H), 6.52 (s, 0.48H), 4.16-3.98 (m, 0.96H), 3.78 (dd, *J* = 8.8, 6.5 Hz, 1.00H), 3.60 (s, 1.55H), 3.57 (s, 1.45H), 3.40 (t, *J* = 7.0 Hz, 1.01H), 3.17 (s, 1.49H), 2.96 (t, *J* = 7.6 Hz, 0.98H), 2.78 (s, 1.47H), 2.73 (t, *J* = 7.0 Hz, 1.05H), 2.70-2.43 (m, 3.01H), 2.30 (s, 1.50H), 2.21 (s, 1.50H), 1.93-1.77 (m, 1.04H); ^{13}C NMR (101 MHz, CDCl$_3$) δ 168.0, 167.5, 165.3, 165.2, 145.7, 145.5, 145.2, 145.1, 140.9, 140.6, 139.2, 139.0, 138.8, 138.5, 138.3, 137.7, 133.1, 133.0, 132.8, 132.6, 129.4, 129.1, 127.9, 127.8, 127.6, 127.5, 126.3, 126.0, 124.7, 116.9, 110.1, 109.8, 52.7, 51.5, 51.4, 50.1, 49.9, 49.7, 37.5, 34.5, 34.3, 34.3, 33.3, 33.0, 32.3, 32.3, 21.5, 21.4; HRMS (m/z, ESI-TOF): Calcd for $C_{25}H_{27}N_2O_3^+$ [M+H$^+$] 403.2016, found 403.2024.

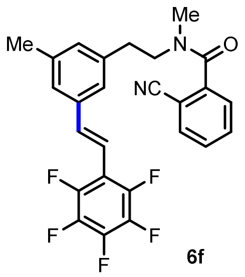
6f

(*E*)-2-cyano-*N*-methyl-*N*-(3-methyl-5-(perfluorostyryl)phenethyl) benzamide: The corresponding reaction was run with standard conditions and DMF (5 equiv) for 28 h. The two rotamers' ratio is about 55:45. ^{1}H NMR (400 MHz, $CDCl_3$) δ 1.72 (d, *J* = 7.7 Hz, 0.56H), 7.69-7.60 (m, 1.12H), 7.55-7.36 (m, 2.69H), 7.29 (t, *J* = 9.1 Hz, 0.98H), 7.25-7.19 (m, 0.96H), 7.14 (s, 0.51H), 6.99 (d, *J* = 16.8 Hz, 0.55H), 6.89-6.80 (m, 1.29H), 6.74 (s, 0.46H), 3.82 (dd, *J* = 8.9, 6.6 Hz, 1.10H), 3.48 (t, *J* = 6.9 Hz, 0.90H), 3.22 (s, 1.39H), 3.04 (t, *J* = 7.6 Hz, 1.10H), 2.87 (s, 1.52H), 2.81 (t, *J* = 6.8 Hz, 0.93H), 2.39 (s, 1.63H), 2.32 (s, 1.33H); ^{13}C NMR (101 MHz, $CDCl_3$, the signal of the peaks of the carbons on the pentafluoroarene are very low and not interpreted.) δ 168.0, 167.7, 140.8, 140.4, 139.5, 139.2, 139.0, 138.4, 137.4, 137.1, 136.9, 133.2, 133.1, 132.8, 132.8, 130.7, 130.5, 129.6, 129.3, 127.8, 127.5, 126.2, 126.0, 124.8, 124.6, 116.9, 116.9, 113.0, 112.7, 110.1, 110.0, 52.6, 49.7, 37.5, 34.4, 33.3, 33.0, 21.4, 21.4; HRMS (m/z, ESI-TOF): Calcd for $C_{26}H_{20}F_5N_2O^+$ [M+H$^+$] 471.1490, found 471.1497.

2.5 Sequential remote-selective *ortho*- and *meta*-C–H bond olefination and characterization of the products.

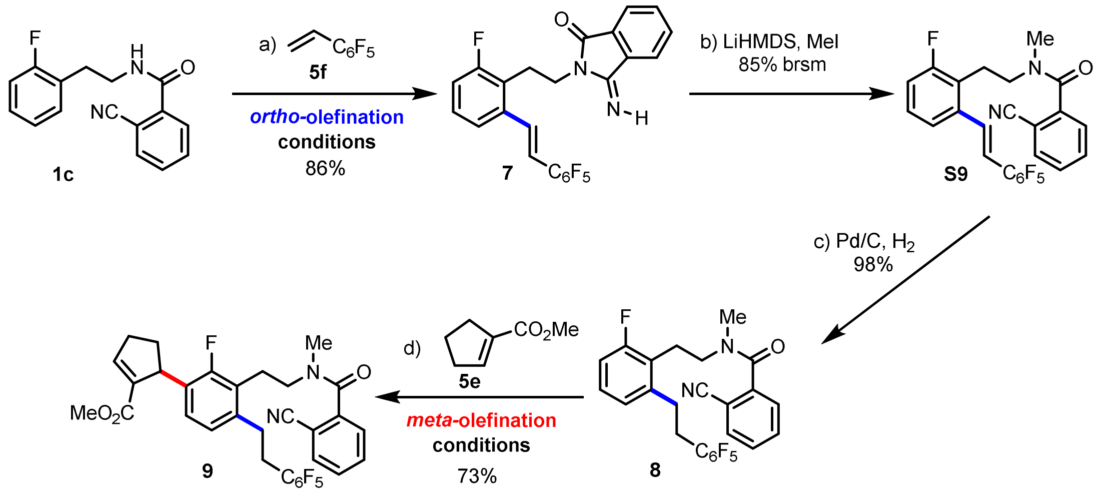

Step a: **7** was obtained in 86% yield using standard *ortho*-olefination conditions in 24 h.

Step b: LHMDS (0.6 mL, 1.0 M in THF) was added to a solution of **7** (110 mg, 0.24 mmol) in anhydrous THF (3 mL) at -15 °C. After stirring for 0.5 h, MeI (45 µL, 0.72 mmol) was added dropwise to the reaction. The reaction mixture was gradually warmed to room temperature and stirred for another 2 h. Then H_2O (10 mL) was added slowly to quench the reaction. The aqueous phase was extracted with ethyl acetate (10 mL × 3). The combined organic phase was dried over anhydrous Na_2SO_4 and concentrated under reduced pressure. The residue was purified by flash silica gel chromatography with petroleum ether/EtOAc (2:1) to afford compound **S9** (66 mg) in 58% yield, and 32% of substrate **7** (35 mg) was also recovered.

Step c: To a solution of compound **S9** (56 mg, 0.12 mmol) in methanol (5 mL) was added Palladium on carbon (10% w/w, 15 mg). The reaction was then stirred at room temperature under hydrogen gas atmosphere (1 atm) for 1 hour. The catalyst was filtered through a short pad of Celite, and the filtrate was concentrated under reduced pressure. The residue was purified by flash silica gel chromatography with petroleum ether/EtOAc (2:1) to obtain compound **8** (56 mg) in 98% yield.

Step d: **9** was obtained in 73% yield using standard *meta*-olefination conditions at 90 °C in 48 h.

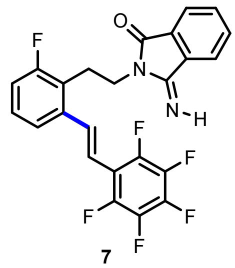

7 **(*E*)-2-(2-fluoro-6-(perfluorostyryl)phenethyl)-3-iminoisoindolin-1-one**: ^{1}H NMR (400 MHz, $CDCl_3$) δ 8.53 (brs, 1H), 7.72-7.50 (m, 5H), 7.32-7.20 (m, 2H), 7.05 (t, *J* = 8.8 Hz, 1H), 6.61 (d, *J* = 16.5 Hz, 1H), 4.05 (t, *J* = 6.8 Hz, 2H), 3.21 (t, *J* = 6.0 Hz, 2H); ^{13}C NMR (101 MHz, $CDCl_3$) δ 167.6, 161.8 (d, *J* = 245 Hz), 146.1 (m), 143.6 (m), 138.75 (d, *J* = 4.5 Hz), 133.8, 132.8, 132.1, 131.0, 128.4 (d, *J* = 9 Hz), 124.8 (d, *J* = 15 Hz), 123.1, 121.7, 120.6 (m), 115.7, 115.5 (d, *J* = 23 Hz), 112.0 (m), 38.1, 24.2 (d, *J* = 4 Hz).

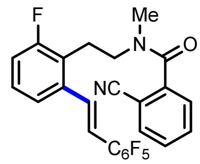

S9 **(*E*)-2-cyano-*N*-(2-fluoro-6-(perfluorostyryl)phenethyl)-*N*-methylbenzamide**: The two rotamers' ratio is about 50:50. ^{1}H NMR (400 MHz, $CDCl_3$) δ 7.84 (d, *J* = 16.5 Hz, 0.48H), 7.69 (d, *J* = 7.7 Hz, 0.47H), 7.64 (t, *J* = 7.7 Hz, 0.51H), 7.58 (d, *J* = 7.6 Hz, 0.51H), 7.54-7.36 (m, 2.71H), 7.36-7.23 (m, 1.94H), 7.07 (t, *J* = 8.9 Hz, 0.49H), 7.02-6.90 (m, 1.47H), 6.67 (d, *J* = 16.5 Hz, 0.50H), 3.83-3.70 (m, 0.95H), 3.43 (t, *J* = 7.0 Hz, 1.02H), 3.32-3.16 (m, 2.49H), 3.01 (t, *J* = 6.9 Hz, 1.02H), 2.93 (s, 1.40H); HRMS (m/z, ESI-TOF): Calcd for $C_{25}H_{17}F_6N_2O^+$ [M+H$^+$] 475.1240, found 475.1247.

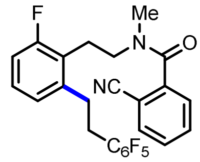

8 **2-Cyano-*N*-(2-fluoro-6-(perfluorophenethyl)phenethyl)-*N*-methylbenzamide**: The two rotamers' ratio is about 57:43. ^{1}H NMR (400 MHz, $CDCl_3$) δ 7.71 (d, *J* = 7.7 Hz, 0.57H), 7.70-7.61 (m, 1.00H), 7.56-7.41 (m, 2.03H), 7.18-7.10 (m, 1.02H), 7.07 (d, *J* = 7.5 Hz, 0.43H), 6.94 (t, *J* = 9.0 Hz, 0.59H), 6.85 (q, *J* = 7.7 Hz, 1.42H), 3.80-3.66 (m, 1.14H), 3.40 (t, *J* = 7.4 Hz, 0.85H), 3.26 (s, 1.29H), 3.18-3.00 (m, 3.51H), 2.98 (s, 1.69H), 2.92 (t, *J* = 7.1 Hz, 0.89H), 2.79 (dd, *J* = 9.6, 6.7 Hz, 0.86H), 2.61 (dd, *J* = 9.9, 6.3 Hz, 0.84H); ^{13}C NMR (101 MHz, $CDCl_3$) δ 167.9, 167.7, 161.9 (d, $J_{C\text{-}F}$ = 244 Hz), 161.8 (d, $J_{C\text{-}F}$ = 246 Hz), 146.5 (m), 143.9 (m), 141.5, 141.5, 140.6, 140.6, 140.3, 138.8 (m), 136.5 (m), 133.1 (d, $J_{C\text{-}F}$ = 27 Hz), 132.9 (d, $J_{C\text{-}F}$ =

9 Hz), 129.6, 129.4, 128.5 (d, $J_{C\text{-}F}$ = 10 Hz), 128.0 (d, $J_{C\text{-}F}$ = 9 Hz), 127.5, 127.5, 125.4 (d, $J_{C\text{-}F}$ = 3 Hz), 125.2 (d, $J_{C\text{-}F}$ = 3 Hz), 124.0 (d, $J_{C\text{-}F}$ = 15 Hz), 123.0 (d, $J_{C\text{-}F}$ = 15 Hz), 116.8, 114.0 (d, $J_{C\text{-}F}$ = 23 Hz), 113.7 (d, $J_{C\text{-}F}$ = 23 Hz), 110.1, 50.8, 47.9, 37.1, 33.2, 32.2, 31.9, 24.4 (d, $J_{C\text{-}F}$ = 2 Hz), 24.2, 24.0, 23.1 (d, $J_{C\text{-}F}$ = 3 Hz); HRMS (m/z, ESI-TOF): Calcd for $C_{25}H_{19}F_6N_2O^+$ [M+H$^+$] 477.1396, found 477.1403.

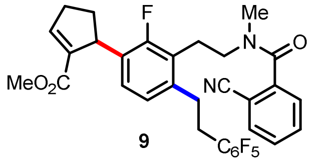
9

Methyl-5-(3-(2-(2-cyano-*N*-methylbenzamido)ethyl)-2-fluoro-4-(perfluorophenethyl)phenyl)cyclopent-1-enecarboxylate: The two rotamers' ratio is about 57:43. ^{1}H NMR (400 MHz, $CDCl_3$) δ 7.71 (d, *J* = 7.7 Hz, 0.57H), 7.69-7.61 (m, 0.99H), 7.55-7.39 (m, 2.01H), 7.07 (d, *J* = 7.6 Hz, 0.44H), 7.03 (t, *J* = 2.2 Hz, 1.00H), 6.84 (td, *J* = 7.8, 3.2 Hz, 0.99H), 6.80-6.69 (m, 0.97H), 4.48-4.38 (m, 0.53H), 4.37-4.27 (m, 0.42H), 3.82-3.68 (m, 1.17H), 3.61 (s, 3.00H), 3.39 (t, *J* = 7.4 Hz, 0.94H), 3.26 (s, 1.30H), 3.13 (t, *J* = 7.5 Hz, 1.08H), 3.01 (s, 2.08 H), 2.97 (s, 1.70H), 2.91 (t, *J* = 7.0 Hz, 0.92H), 2.74 (t, *J* = 8.1 Hz, 0.96H), 2.69-2.42 (m, 3.90H), 1.92-1.72 (m, 1.30H); ^{13}C NMR (101 MHz, $CDCl_3$) δ 167.9, 167.7, 165.1, 165.0, 159.5 (d, $J_{C\text{-}F}$ = 245 Hz), 146.2, 145.9, 143.9 (m), 140.7, 140.5, 139.0 (d, $J_{C\text{-}F}$ = 4 Hz), 138.7 (m), 138.2 (d, $J_{C\text{-}F}$ = 4 Hz), 137.8, 137.4, 136.3 (m), 133.2, 133.0, 132.8, 130.3 (d, $J_{C\text{-}F}$ = 36 Hz), 130.1 (d, $J_{C\text{-}F}$ = 36 Hz), 129.6, 129.3, 127.6, 126.7 (d, $J_{C\text{-}F}$ = 6 Hz), 126.1 (d, $J_{C\text{-}F}$ = 6 Hz), 125.0 (d, $J_{C\text{-}F}$ = 3 Hz), 124.7 (d, $J_{C\text{-}F}$ = 3 Hz), 123.9 (d, $J_{C\text{-}F}$ = 17 Hz), 122.8 (d, $J_{C\text{-}F}$ = 16 Hz), 116.9, 116.8, 114.2 (m), 110.1, 110.0, 77.5, 77.2, 76.8, 51.5, 51.5, 50.9, 48.0, 43.0 (d, $J_{C\text{-}F}$ = 2 Hz), 37.2, 33.2, 33.0, 32.9, 32.2, 32.2, 32.1, 31.7, 24.6 (d, $J_{C\text{-}F}$ = 3 Hz), 24.2, 23.8, 23.4 (d, $J_{C\text{-}F}$ = 4 Hz); HRMS (m/z, ESI-TOF): Calcd for $C_{32}H_{27}F_6N_2O_3^+$ [M+H$^+$] 601.1920, found 601.1927.

2.6 Removal of the directing group and characterization of selected hydrolyzed products.

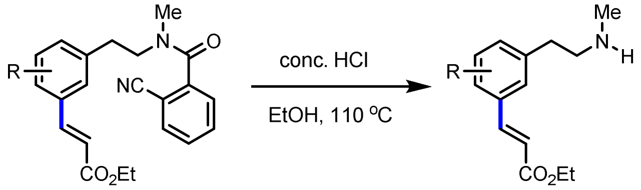

4 and 9 → S10-S16

To a solution of **4** (0.1 mmol) in EtOH (1 mL) was added HCl (aqueous solution, 1 mL, 36 wt%) dropwise at 0 °C. After stirring for 10 min at 0 °C, the reaction mixture was allowed to reflux at 110 °C under nitrogen atmosphere. After the reaction was completed, the reaction mixture was diluted with 5 mL of water. After basification with ammonium hydroxide solution at 0 °C under inert atmosphere until pH value reached about 10, the solution was extracted with EtOAc (20 mL × 2). The combined organic phase was washed with brine, dried over Na_2SO_4 and concentrated *in vacuo*. The crude mixture was purified by preparative thin layer chromatography using EtOAc/Et_3N (50/1) as the eluent to yield the product in 70% to 90% yield.

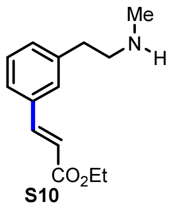

S10 **Ethyl-(*E*)-3-(3-(2-(methylamino)ethyl)phenyl)acrylate**: ^{1}H NMR (400 MHz, $CDCl_3$) δ 7.63 (d, *J* = 16.0 Hz, 1H), 7.43-7.35 (m, 2H), 7.31 (td, *J* = 7.4, 1.1 Hz, 1H), 7.24 (dt, *J* = 7.5, 1.5 Hz, 1H), 6.42 (d, *J* = 16.0 Hz, 1H), 5.63 (brs, 1H), 4.25 (q, *J* = 7.1 Hz, 2H), 3.02 (s, 4H), 2.58 (s, 3H), 1.32 (t, *J* = 7.1 Hz, 3H).

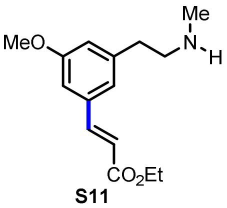

S11 **Ethyl-(*E*)-3-(3-methoxy-5-(2-(methylamino)ethyl)phenyl)acrylate**: Note: one drop of D_2O was added to the sample solution to suppress the NH peak in the NMR spectrum. ^{1}H NMR (400 MHz, $CDCl_3$) δ 7.59 (d, *J* = 16.1 Hz, 1H), 6.98 (s, 1H), 6.90 (t, *J* = 1.7 Hz, 1H), 6.80 (s, 1H), 6.40 (d, *J* = 16.0 Hz, 1H), 4.25 (q, *J* = 7.1 Hz, 2H), 3.80 (s, 3H), 2.99 (s, 4H), 2.56 (s, 3H), 1.32 (t, *J* = 7.1 Hz, 3H).

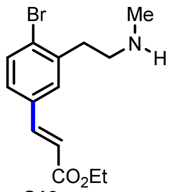

S12 **Ethyl-(*E*)-3-(4-bromo-3-(2-(methylamino)ethyl)phenyl)acrylate**: ^{1}H NMR (400 MHz, $CDCl_3$) δ 7.58 (d, *J* = 16.4 Hz, 1H), 7.55 (d, *J* = 8.6 Hz, 1H), 7.43 (d, *J* = 2.2 Hz, 1H), 7.26-7.22 (m, 1H), 6.43 (d, *J* = 16.0 Hz, 1H), 4.25 (q, *J* = 7.1 Hz, 2H), 3.78 (brs, 1H), 3.11 (dd, *J* = 8.7, 6.3 Hz, 2H), 2.99 (dd, *J* = 9.1, 6.2 Hz, 2H), 2.59 (s, 3H), 1.33 (t, *J* = 7.1 Hz, 3H).

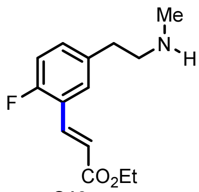

S13 **Ethyl-(*E*)-3-(2-fluoro-5-(2-(methylamino)ethyl)phenyl)acrylate**: ^{1}H NMR (400 MHz, $CDCl_3$) δ 7.74 (d, *J* = 16.2 Hz, 1H), 7.38 (dd, J_{F-H} = 6.9, J_{H-H} = 2.3 Hz, 1H), 7.20 (ddd, J_{F-H} = 4.9, J_{H-H} = 8.4, 2.3 Hz, 1H), 7.02 (dd, J_{F-H} = 10.4, J_{H-H} = 8.4 Hz, 1H), 6.52 (d, *J* = 16.2 Hz, 1H), 5.07 (s, 3H), 4.25 (q, *J* = 7.1 Hz, 2H), 2.98 (s, 4H), 2.57 (s, 3H), 1.32 (t, *J* = 7.1 Hz, 3H).

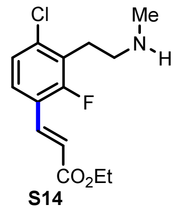

S14 **Ethyl-(*E*)-3-(4-chloro-2-fluoro-3-(2-(methylamino)ethyl)phenyl)acrylate**: ^{1}H NMR (400 MHz, $CDCl_3$) δ 7.75 (d, J = 16.2 Hz, 1H), 7.34 (t, $J_{F\text{-}H\ and\ H\text{-}H}$ = 8.1 Hz, 1H), 7.18 (dd, $J_{H\text{-}H}$ = 8.4, $J_{F\text{-}H}$ = 1.2 Hz, 1H), 6.49 (d, J = 16.2 Hz, 1H), 4.26 (q, J = 7.1 Hz, 2H), 3.05 (td, $J_{H\text{-}H}$ = 7.5, $J_{F\text{-}H}$ = 2.3 Hz, 2H), 2.86 (dd, J = 8.4, 6.6 Hz, 2H), 2.53 (brs, 1H), 2.51 (s, 3H), 1.33 (t, J = 7.1 Hz, 3H).

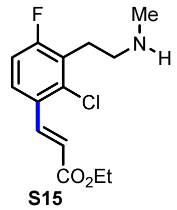

S15 **Ethyl-(*E*)-3-(2-chloro-4-fluoro-3-(2-(methylamino)ethyl)phenyl)acrylate**: ^{1}H NMR (400 MHz, $CDCl_3$) δ 8.05 (d, J = 15.9 Hz, 1H), 7.50 (dd, $J_{H\text{-}H}$ = 8.8, $J_{F\text{-}H}$ = 5.9 Hz, 1H), 7.01 (t, $J_{F\text{-}H\ and\ H\text{-}H}$ = 8.7 Hz, 1H), 6.33 (d, J = 15.9 Hz, 1H), 4.27 (q, J = 7.2 Hz, 2H), 3.72 (s, 1H), 3.14 (td, $J_{H\text{-}H}$ = 8.2, $J_{F\text{-}H}$ = 2.0 Hz, 2H), 2.92 (dd, J = 8.7, 6.6 Hz, 2H), 2.56 (s, 3H), 1.34 (t, J = 7.1 Hz, 3H).

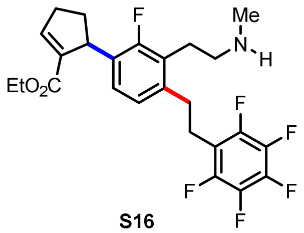

S16 **Ethyl-5-(2-fluoro-3-(2-(methylamino)ethyl)-4-(perfluorophenethyl)phenyl)cyclopent-1-enecarboxylate**: ^{1}H NMR (400 MHz, $CDCl_3$) δ 7.01 (s, 1H), 6.84 (t, $J_{F\text{-}H\ and\ H\text{-}H}$ = 7.7 Hz, 1H), 6.78 (d, J = 8.0 Hz, 1H), 4.41 (d, J = 8.4 Hz, 1H), 4.14-3.99 (m, 2H), 3.01-2.78 (m, 8H), 2.71 – 2.43 (m, 8H), 1.14 (t, J = 7.1 Hz, 3H).

2.7 Hydrolysis of compound $4a_{mono}$ to S17.

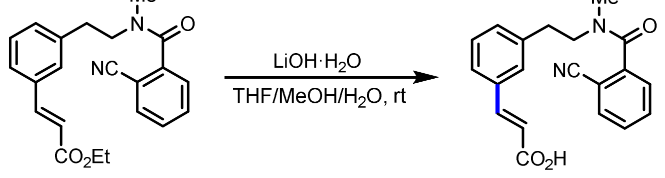

$4a_{mono}$ S17

Compound **$4a_{mono}$** (22 mg, 0.06 mmol) was dissolved in a solution of THF (1.5 mL), MeOH (1.0 mL) and H_2O (0.5 mL). The solution was cooled to 0 °C and LiOH·H_2O (10 mg, 0.24 mmol) was added. The mixture was stirred at room temperature for 2 h and then H_2O (10 mL) was

added. The solution was extracted with Et_2O (5 mL × 2) and the aqueous phase was acidized with 1N HCl (1 mL). The aqueous phase was extracted with CH_2Cl_2 (5 mL × 4). The combined organic phase was dried over anhydrous Na_2SO_4 and concentrated under reduced pressure to give compound **S17** (17 mg) in 85% yield. **S17**: ^{1}H NMR (400 MHz, $CDCl_3$) δ 7.78 (d, *J* = 16.0 Hz, 0.63H), 7.74-7.59 (m, 2.09H), 7.56-7.28 (m, 5.50H), 7.12-6.95 (m, 0.88H), 6.89-6.77 (m, 0.42H), 6.49 (d, *J* = 15.9 Hz, 0.56H), 6.35 (d, *J* = 15.9 Hz, 0.42H), 3.83 (t, *J* = 7.4 Hz, 1.14H), 3.48 (t, *J* = 6.5 Hz, 0.86H), 3.22 (s, 1.36H), 3.08 (t, *J* = 7.3 Hz, 1.26H), 2.89-2.79 (m, 2.61H); ^{13}C NMR (101 MHz, $CDCl_3$) δ 171.3, 171.1, 168.1, 167.8, 146.9, 146.3, 140.6, 140.2, 139.7, 138.6, 134.8, 134.6, 133.3, 133.1, 133.0, 132.8, 131.5, 131.3, 129.65, 129.62, 129.5, 129.4, 129.0, 128.7, 127.7, 127.5, 127.0, 126.8, 117.9, 117.6, 116.9, 116.9, 110.1, 109.9, 52.5, 49.5, 37.5, 34.4, 33.3, 33.1.

2.8 Reference.

[S1] 2-Cyanobenzoic acid was synthesized in large scale as reported: Goossen, L. J.; Melzer, B. *J. Org. Chem.* **2007**, *72*, 7473. 2-Cyanobenzoic acid could be stored as a solid at -18 °C for a few months.

[S2] Karla, R.; Ebert, B.; Thorkildsen, C.; Herdeis, C.; Johansen, T. N.; Nielsen, B.; Krogsgaard-Larsen, P. *J. Med. Chem.* **1999**, *42*, 2053.

3. NMR Spectra of New Compounds

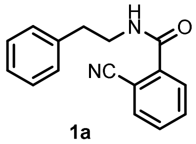

1a

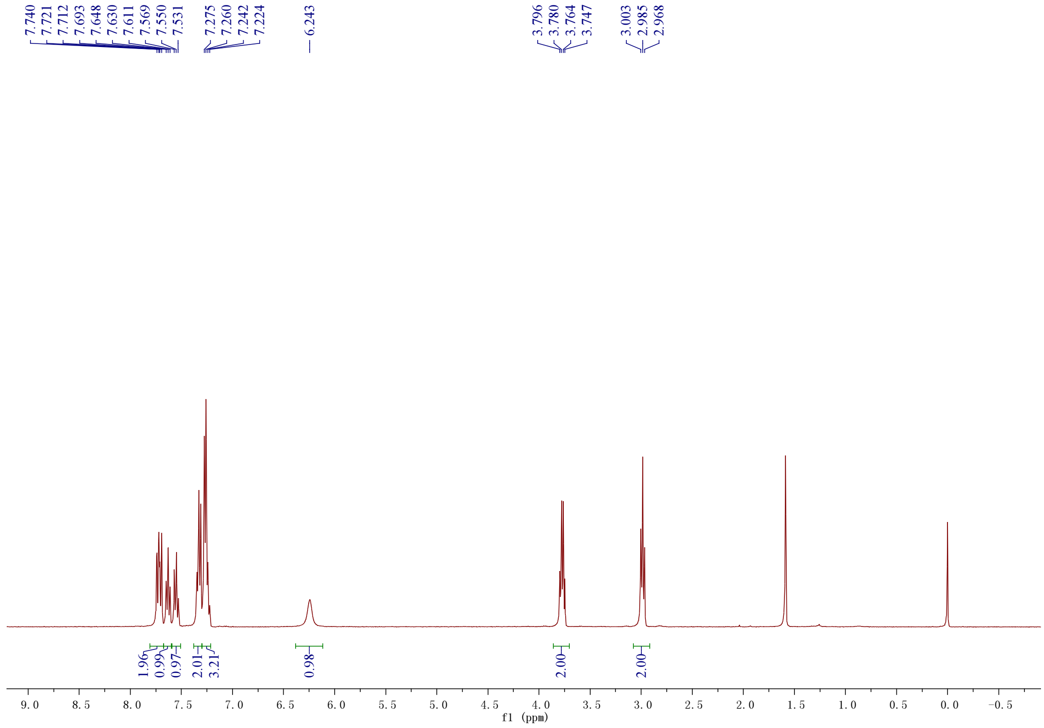

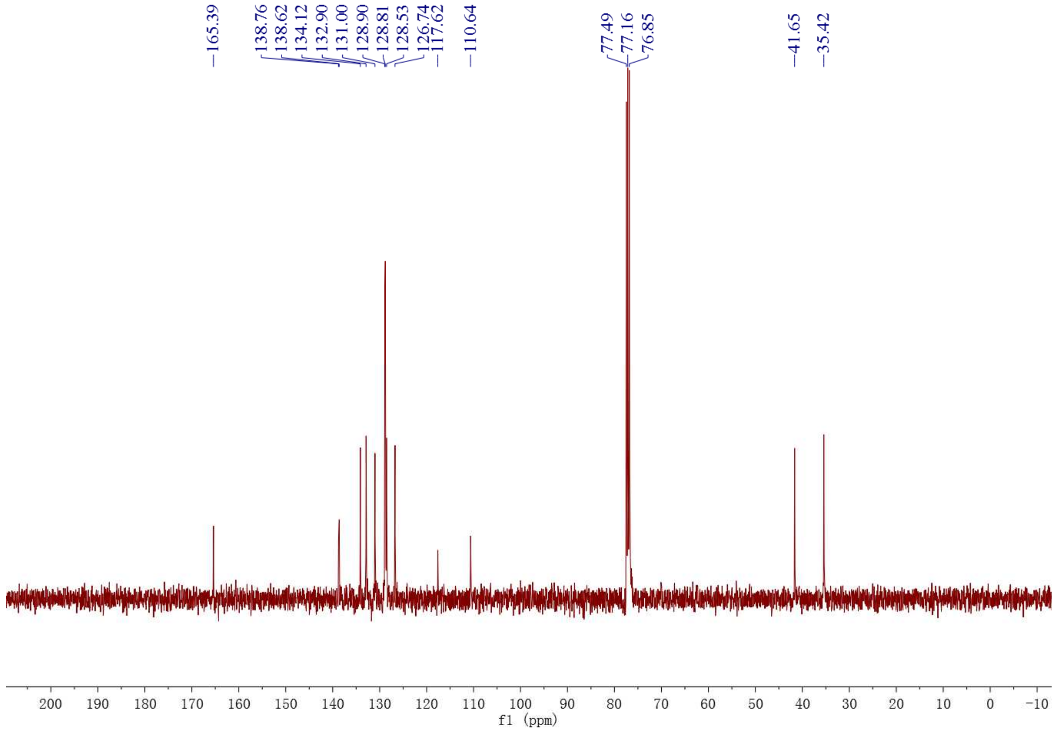

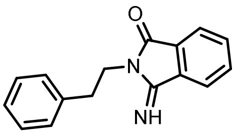
O
N
NH

1a'

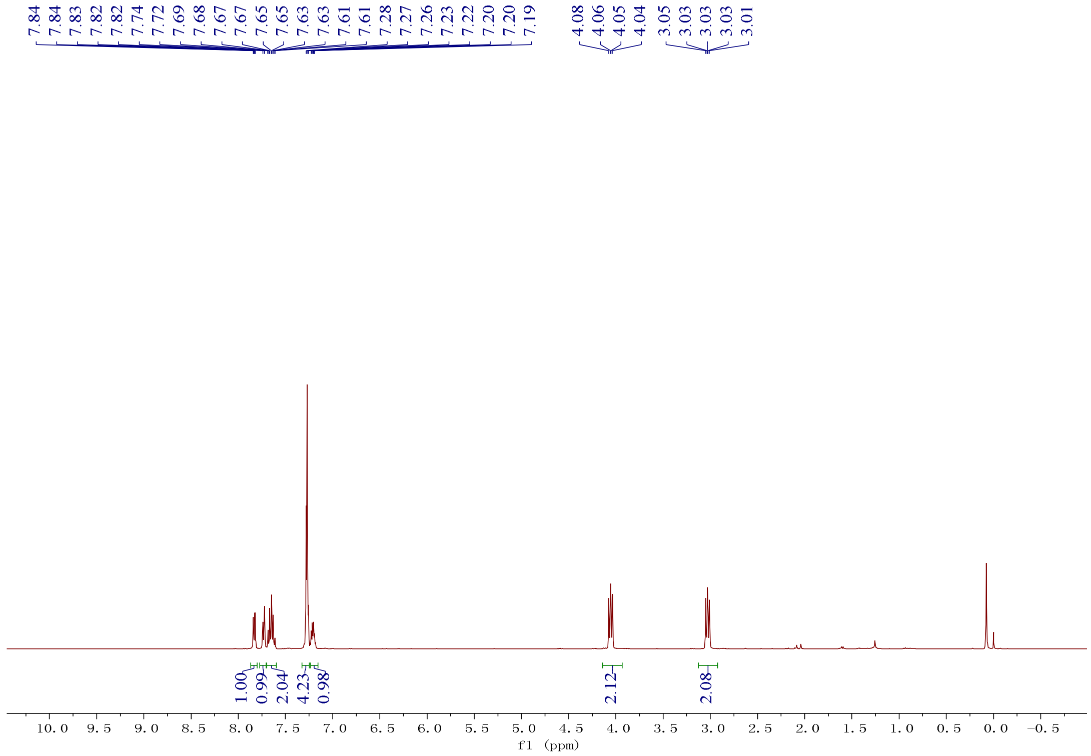
7.84 7.84 7.83 7.82 7.82 7.74 7.72 7.69 7.68 7.67 7.67 7.65 7.65 7.63 7.63 7.61 7.61 7.28 7.27 7.26 7.23 7.22 7.20 7.20 7.19
4.08 4.06 4.05 4.04
3.05 3.03 3.03 3.03 3.01
1.00 0.99 2.04 4.23 0.98
2.12
2.08
10.0 9.5 9.0 8.5 8.0 7.5 7.0 6.5 6.0 5.5 5.0 4.5 4.0 3.5 3.0 2.5 2.0 1.5 1.0 0.5 0.0 -0.5
f1 (ppm)

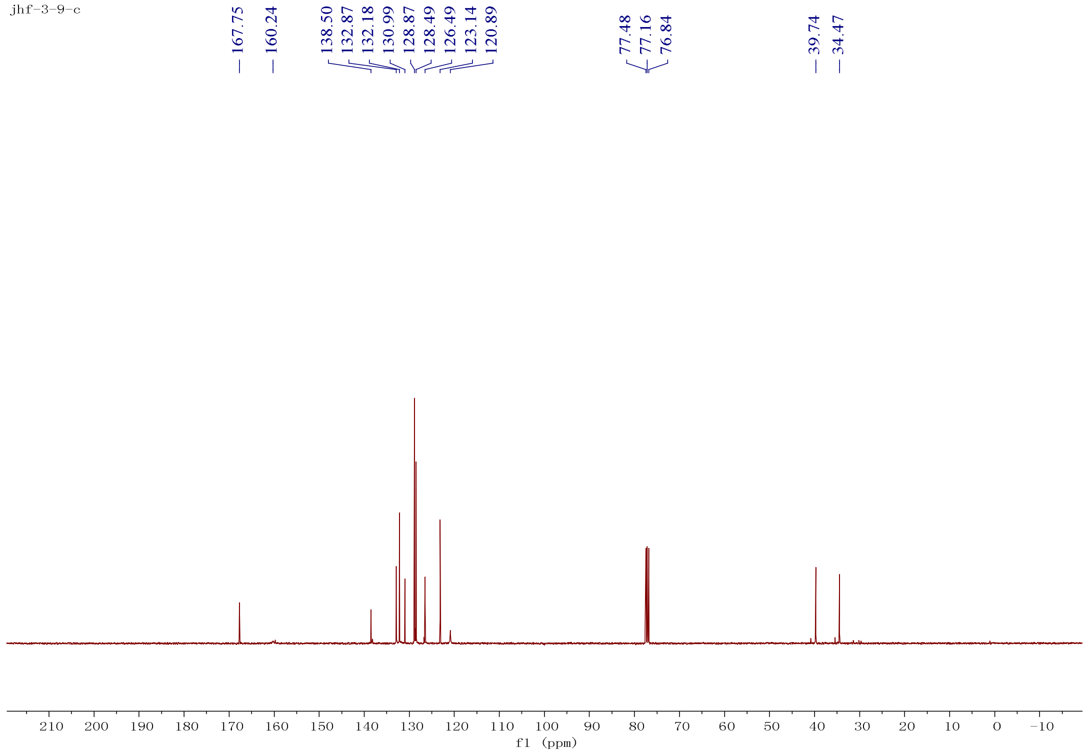
jhf-3-9-c
167.75
160.24
138.50 132.87 132.18 130.99 128.87 128.49 126.49 123.14 120.89
77.48 77.16 76.84
39.74
34.47
210 200 190 180 170 160 150 140 130 120 110 100 90 80 70 60 50 40 30 20 10 0 -10
f1 (ppm)

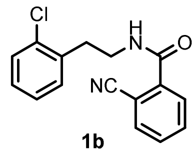
Cl
H
N
O
NC

1b

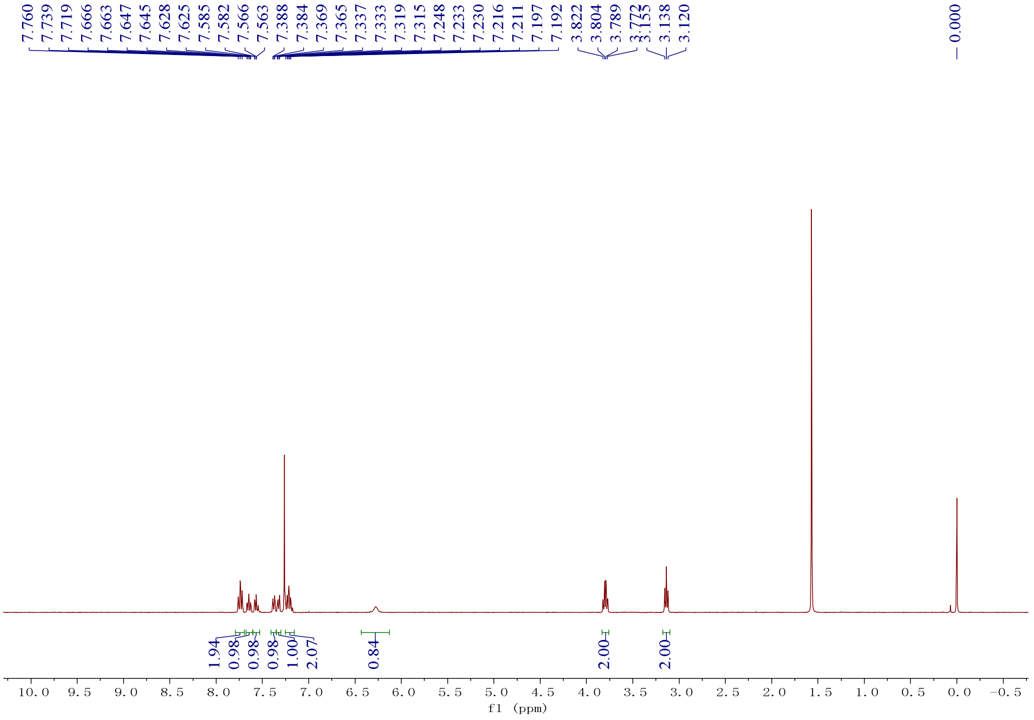
7.760
7.739
7.719
7.666
7.663
7.647
7.645
7.628
7.625
7.585
7.582
7.566
7.563
7.388
7.384
7.369
7.365
7.337
7.333
7.319
7.315
7.248
7.233
7.230
7.216
7.211
7.197
7.192
3.822
3.804
3.789
3.772
3.155
3.138
3.120
0.000
1.94
0.98
0.98
0.98
1.00
2.07
0.84
2.00
2.00
10.0 9.5 9.0 8.5 8.0 7.5 7.0 6.5 6.0 5.5 5.0 4.5 4.0 3.5 3.0 2.5 2.0 1.5 1.0 0.5 0.0 -0.5
f1 (ppm)

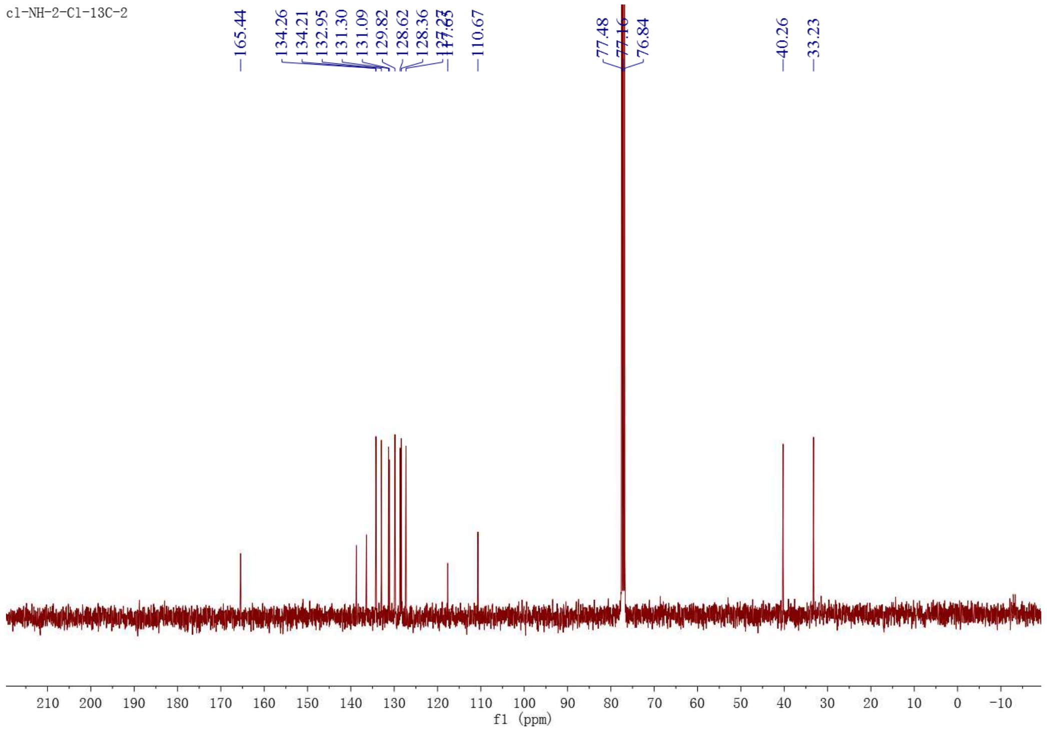
cl-NH-2-Cl-13C-2
165.44
134.26
134.21
132.95
131.30
131.09
129.82
128.62
128.36
110.67
77.48
77.16
76.84
40.26
33.23
210 200 190 180 170 160 150 140 130 120 110 100 90 80 70 60 50 40 30 20 10 0 -10
f1 (ppm)

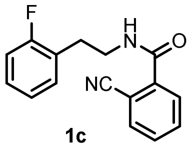
F
H
N
O
NC

1c

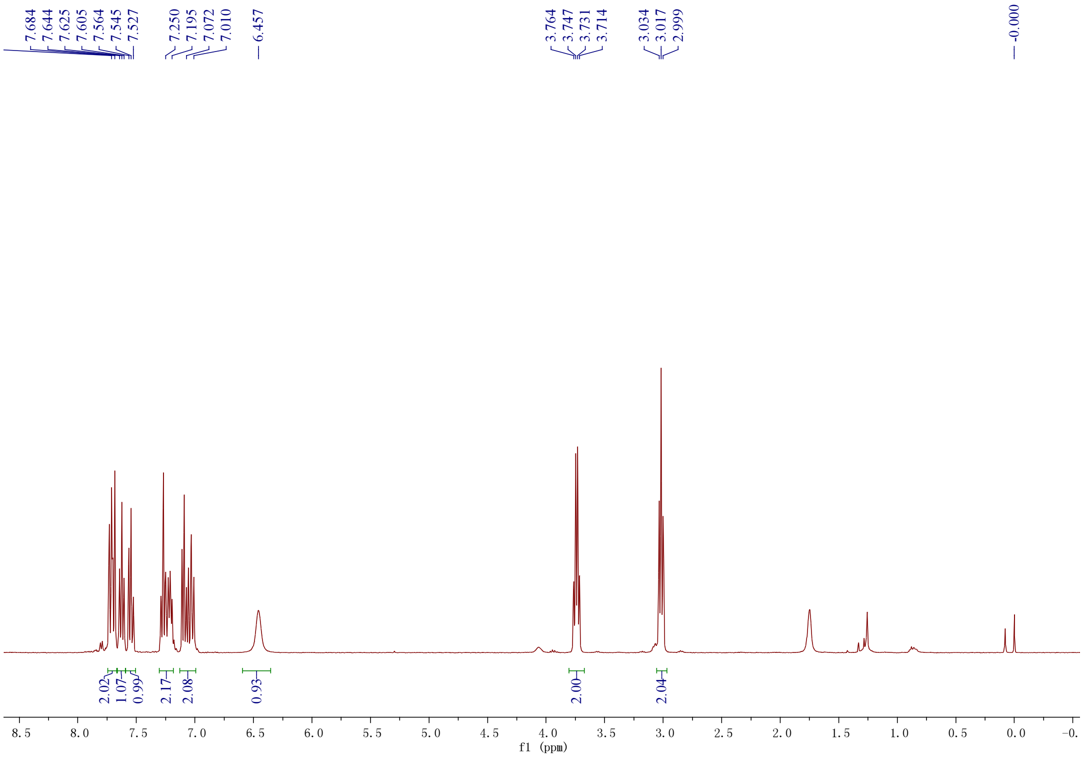
7.684
7.644
7.625
7.605
7.564
7.545
7.527
7.250
7.195
7.072
7.010
6.457
3.764
3.747
3.731
3.714
3.034
3.017
2.999
-0.000
2.02
1.07
0.99
2.17
2.08
0.93
2.00
2.04
f1 (ppm)

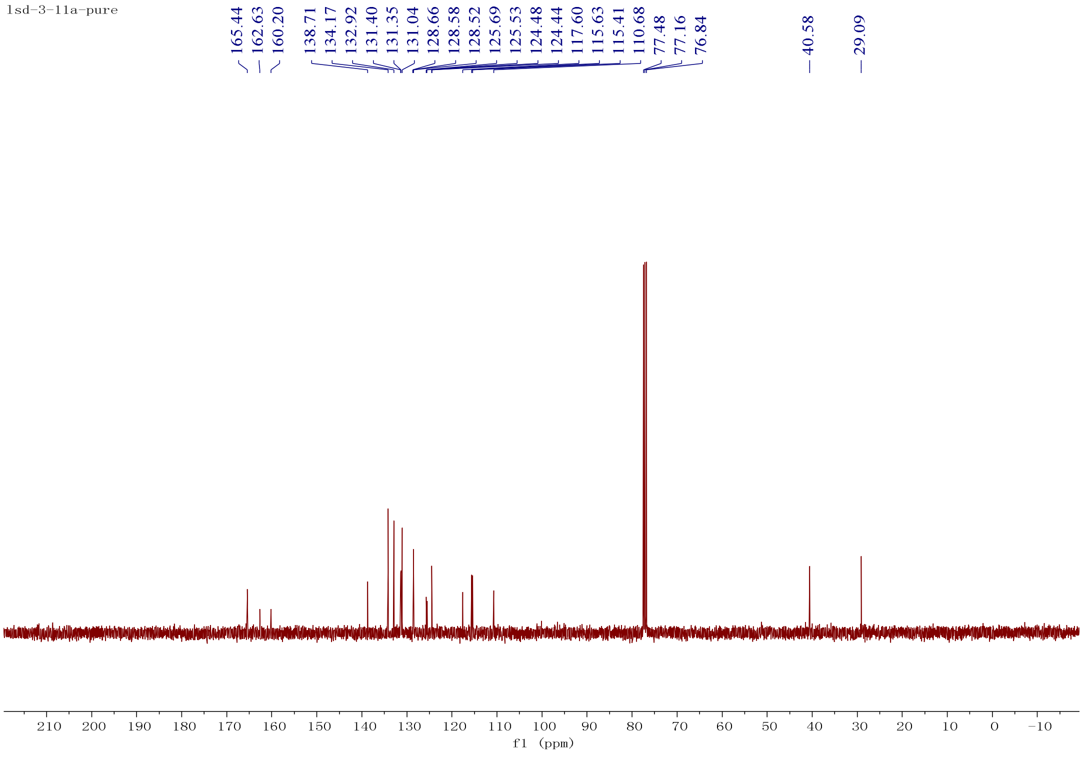
lsd-3-11a-pure
165.44
162.63
160.20
138.71
134.17
132.92
131.40
131.35
131.04
128.66
128.58
128.52
125.69
125.53
124.48
124.44
117.60
115.63
115.41
110.68
77.48
77.16
76.84
40.58
29.09
f1 (ppm)

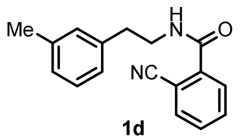
Me
H
N
O
NC

1d

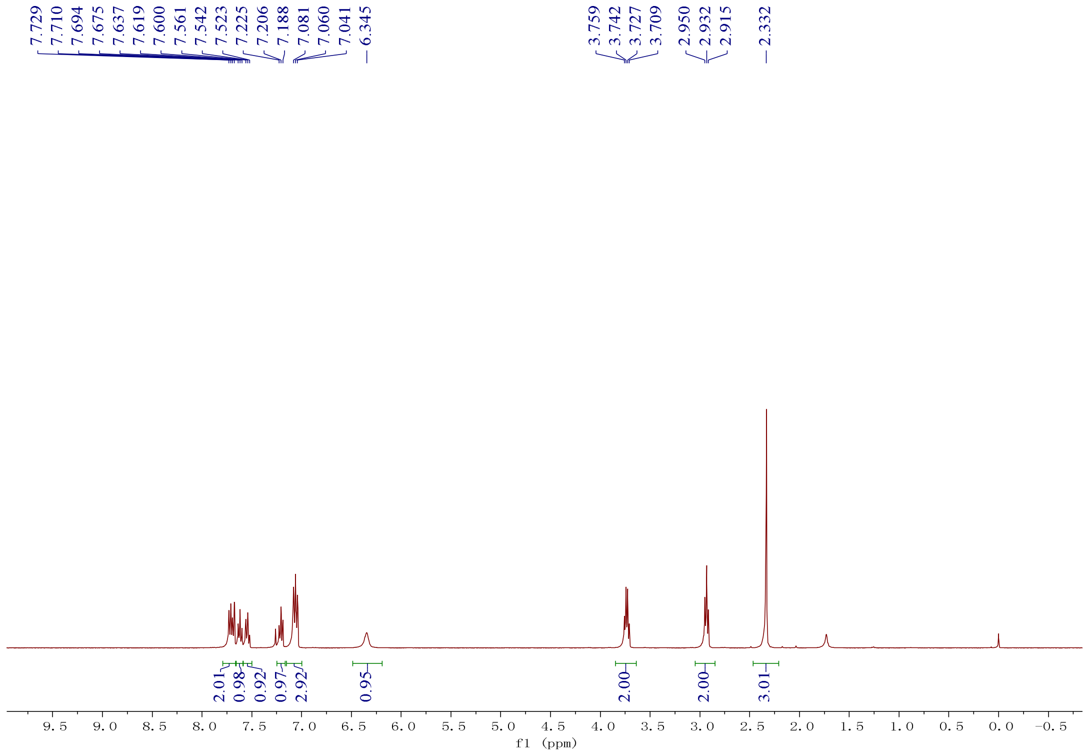
7.729
7.710
7.694
7.675
7.637
7.619
7.600
7.561
7.542
7.523
7.225
7.206
7.188
7.081
7.060
7.041
6.345
3.759
3.742
3.727
3.709
2.950
2.932
2.915
2.332
2.01
0.98
0.92
0.97
2.92
0.95
2.00
2.00
3.01
9.5
9.0
8.5
8.0
7.5
7.0
6.5
6.0
5.5
5.0
4.5
4.0
3.5
3.0
2.5
2.0
1.5
1.0
0.5
0.0
-0.5
f1 (ppm)

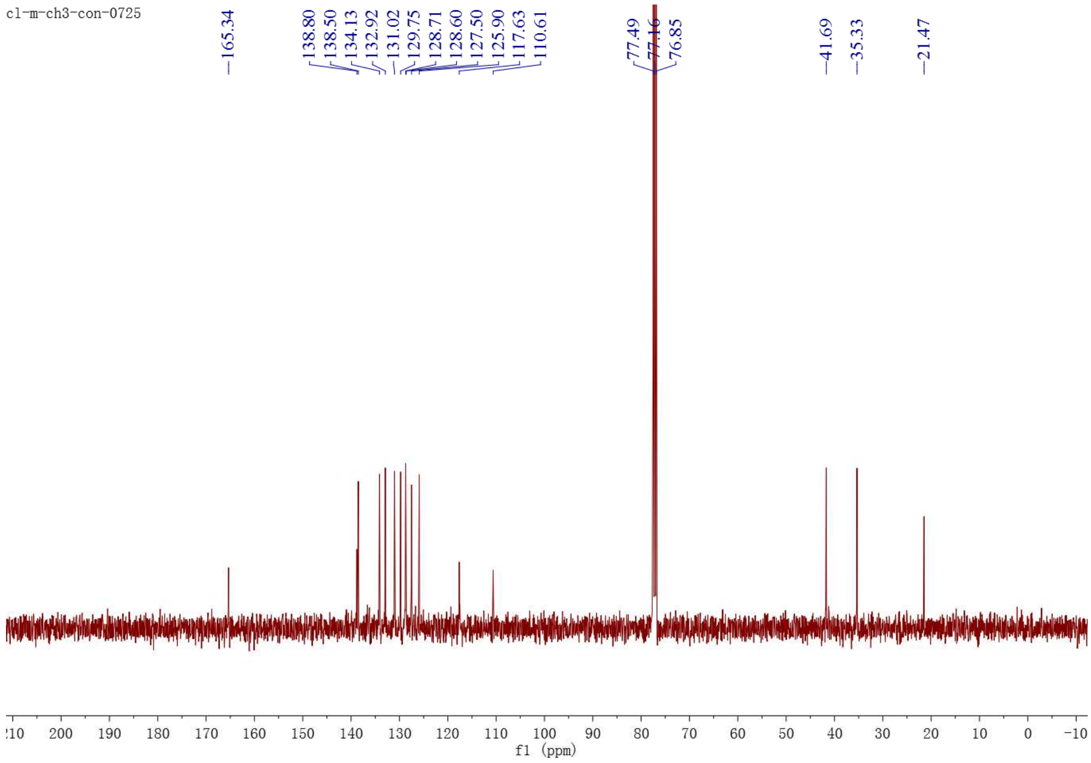
cl-m-ch3-con-0725
165.34
138.80
138.50
134.13
132.92
131.02
129.75
128.71
128.60
127.50
125.90
117.63
110.61
77.49
77.16
76.85
41.69
35.33
21.47
210
200
190
180
170
160
150
140
130
120
110
100
90
80
70
60
50
40
30
20
10
0
-10
f1 (ppm)

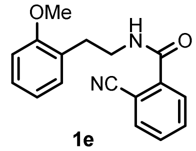
OMe
H
N
O
NC

1e

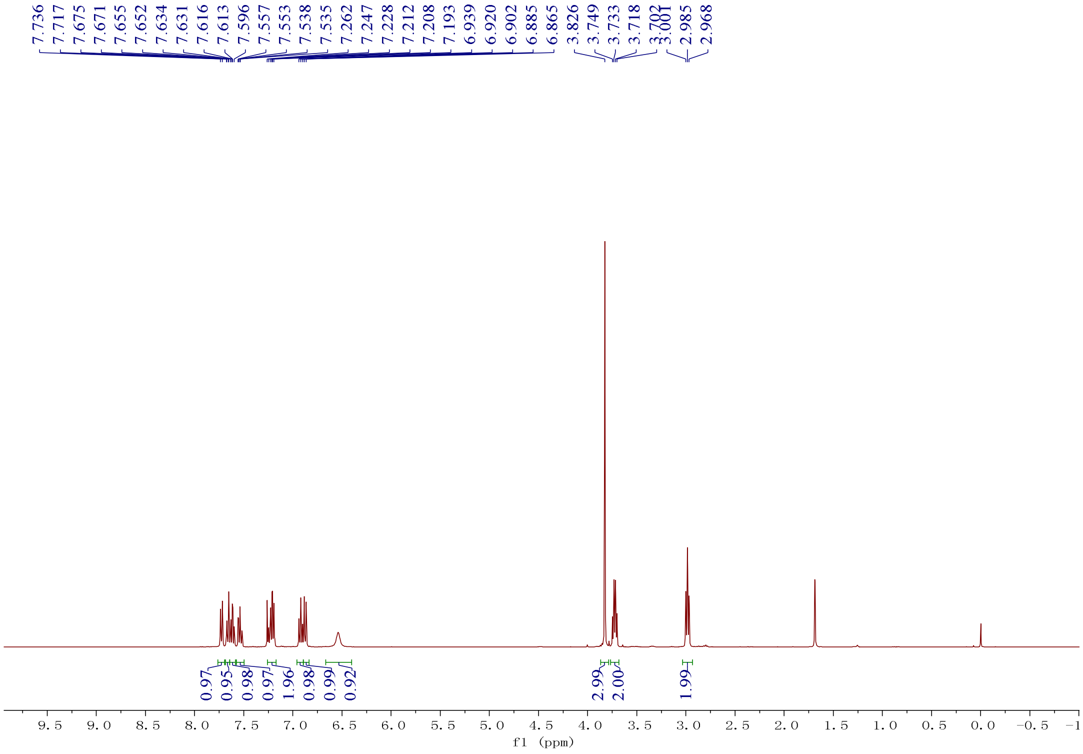
7.736
7.717
7.675
7.671
7.655
7.652
7.634
7.631
7.616
7.613
7.596
7.557
7.553
7.538
7.535
7.262
7.247
7.228
7.212
7.208
7.193
6.939
6.920
6.902
6.885
6.865
3.826
3.749
3.733
3.718
3.702
3.001
2.985
2.968
0.97
0.95
0.98
0.97
1.96
0.98
0.99
0.92
2.99
2.00
1.99
9.5
9.0
8.5
8.0
7.5
7.0
6.5
6.0
5.5
5.0
4.5
4.0
3.5
3.0
2.5
2.0
1.5
1.0
0.5
0.0
-0.5
-1
f1 (ppm)

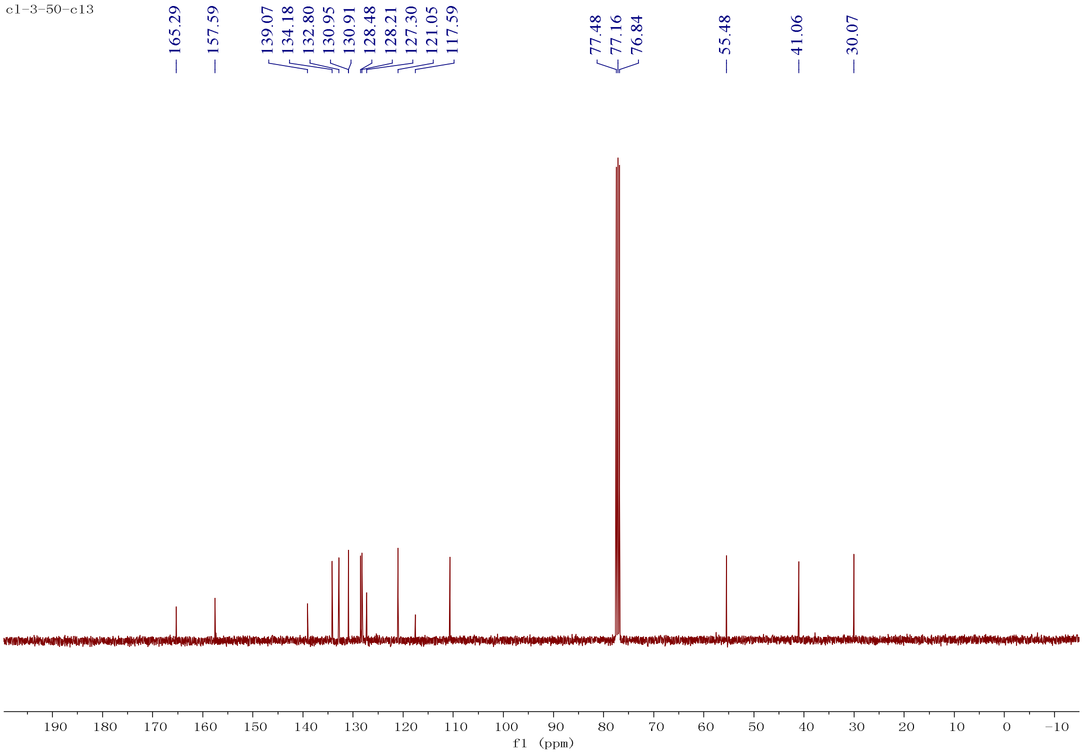
c1-3-50-c13
165.29
157.59
139.07
134.18
132.80
130.95
130.91
128.48
128.21
127.30
121.05
117.59
77.48
77.16
76.84
55.48
41.06
30.07
190
180
170
160
150
140
130
120
110
100
90
80
70
60
50
40
30
20
10
0
-10
f1 (ppm)

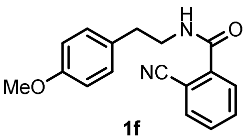
MeO
NC
H
N
O

1f

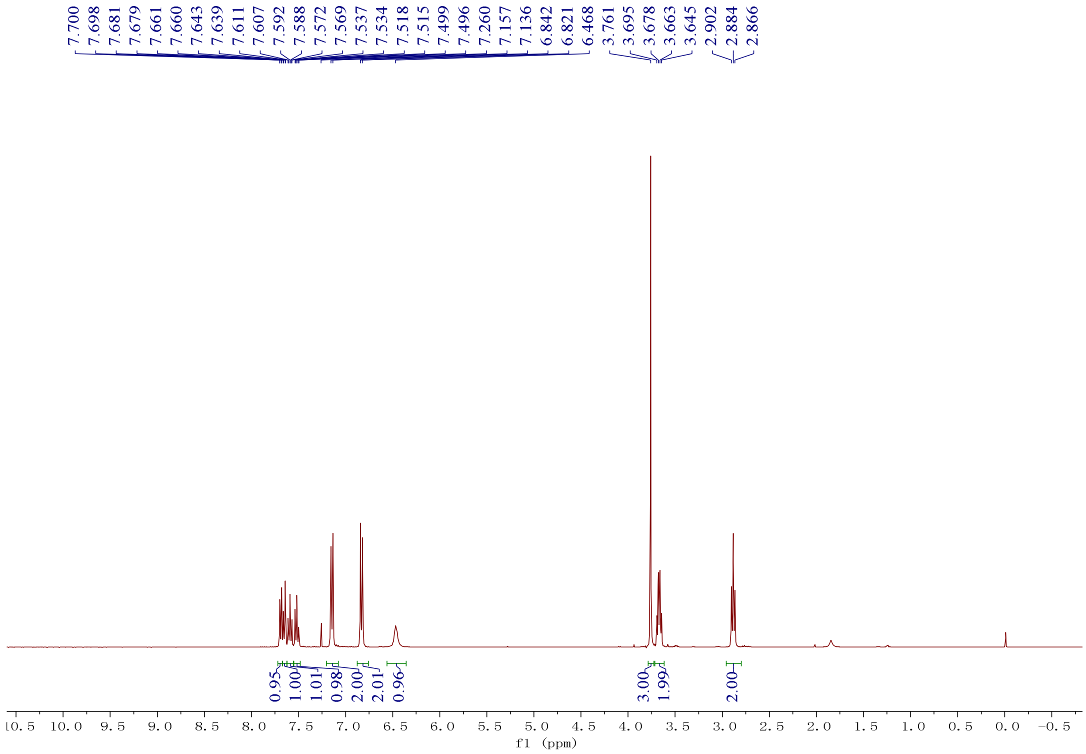
7.700
7.698
7.681
7.679
7.661
7.660
7.643
7.639
7.611
7.607
7.592
7.588
7.572
7.569
7.537
7.534
7.518
7.515
7.499
7.496
7.260
7.157
7.136
6.842
6.821
6.468
3.761
3.695
3.678
3.663
3.645
2.902
2.884
2.866
0.95
1.00
1.01
0.98
2.00
2.01
0.96
3.00
1.99
2.00
10.5 10.0 9.5 9.0 8.5 8.0 7.5 7.0 6.5 6.0 5.5 5.0 4.5 4.0 3.5 3.0 2.5 2.0 1.5 1.0 0.5 0.0 -0.5
f1 (ppm)

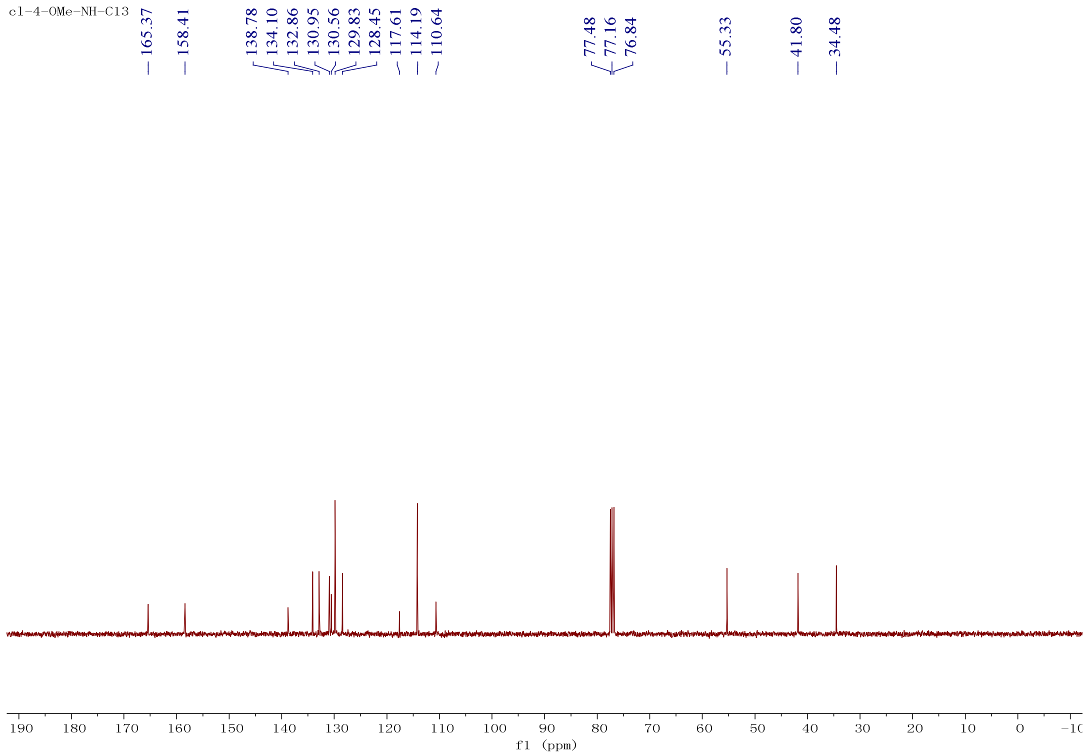
cl-4-OMe-NH-C13
165.37
158.41
138.78
134.10
132.86
130.95
130.56
129.83
128.45
117.61
114.19
110.64
77.48
77.16
76.84
55.33
41.80
34.48
190 180 170 160 150 140 130 120 110 100 90 80 70 60 50 40 30 20 10 0 -10
f1 (ppm)

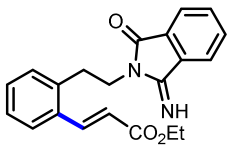
O
N
NH
CO2Et

2a$_{mono}$

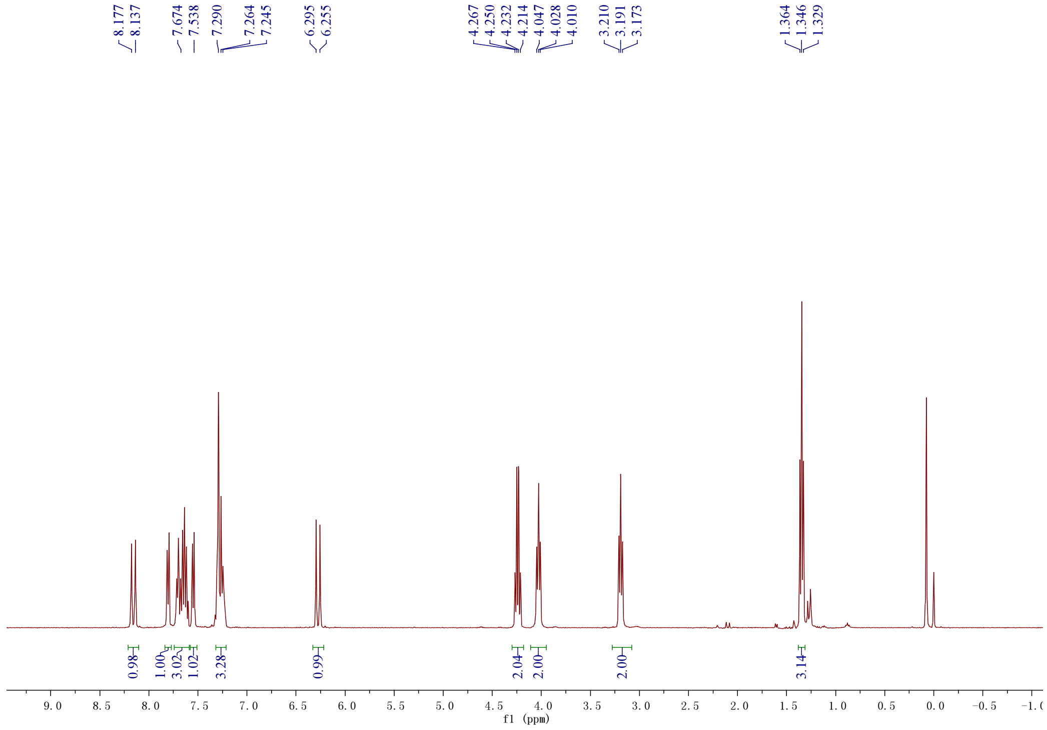
8.177
8.137
7.674
7.538
7.290
7.264
7.245
6.295
6.255
4.267
4.250
4.232
4.214
4.047
4.028
4.010
3.210
3.191
3.173
1.364
1.346
1.329
0.98
1.00
3.02
1.02
3.28
0.99
2.04
2.00
2.00
3.14
9.0 8.5 8.0 7.5 7.0 6.5 6.0 5.5 5.0 4.5 4.0 3.5 3.0 2.5 2.0 1.5 1.0 0.5 0.0 -0.5
f1 (ppm)

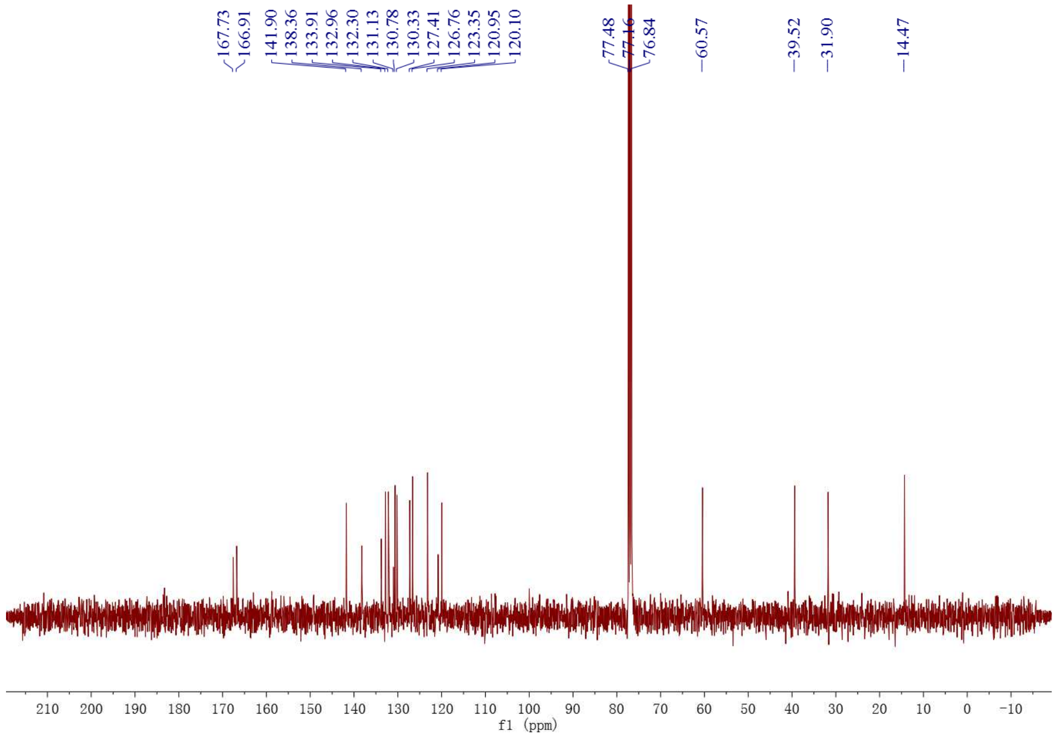
167.73
166.91
141.90
138.36
133.91
132.96
132.30
131.13
130.78
130.33
127.41
126.76
123.35
120.95
120.10
77.48
77.16
76.84
60.57
39.52
31.90
14.47
210 200 190 180 170 160 150 140 130 120 110 100 90 80 70 60 50 40 30 20 10 0 -10
f1 (ppm)

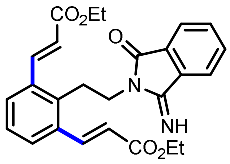
CO2Et
O
N
NH
CO2Et

2a$_{(o,o')di}$

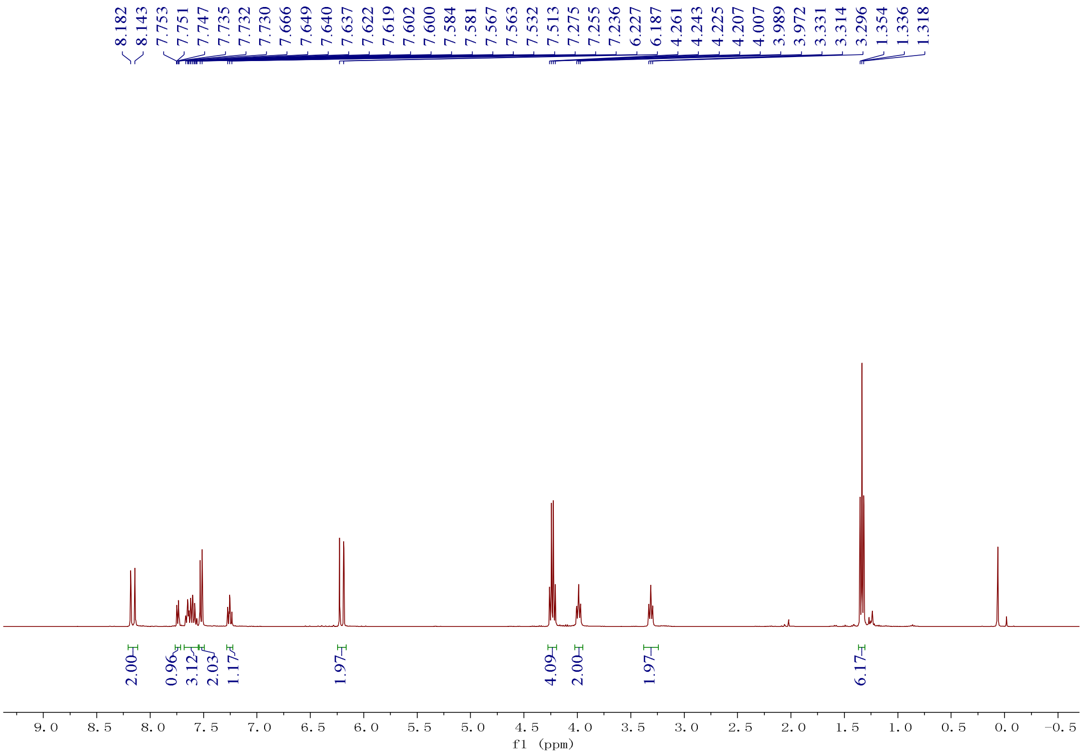
8.182
8.143
7.753
7.751
7.747
7.735
7.732
7.730
7.666
7.649
7.640
7.637
7.622
7.619
7.602
7.600
7.584
7.581
7.567
7.563
7.532
7.513
7.275
7.255
7.236
6.227
6.187
4.261
4.243
4.225
4.207
4.007
3.989
3.972
3.331
3.314
3.296
1.354
1.336
1.318
2.00
0.96
3.12
2.03
1.17
1.97
4.09
2.00
1.97
6.17
f1 (ppm)

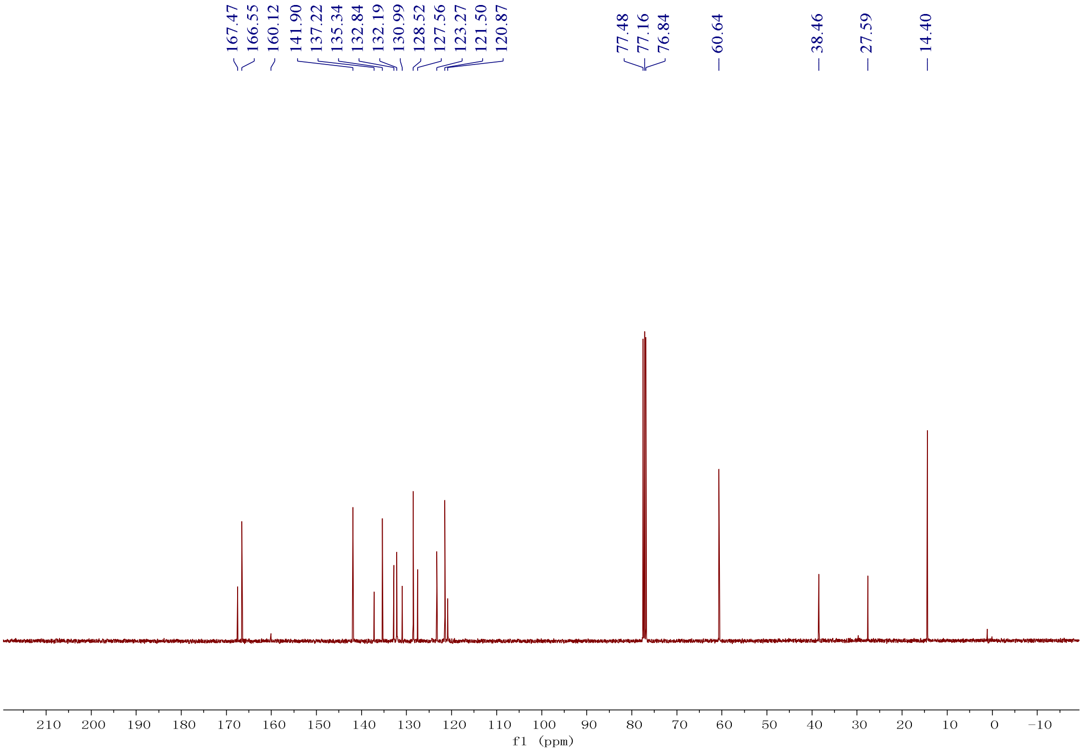
167.47
166.55
160.12
141.90
137.22
135.34
132.84
132.19
130.99
128.52
127.56
123.27
121.50
120.87
77.48
77.16
76.84
60.64
38.46
27.59
14.40
f1 (ppm)

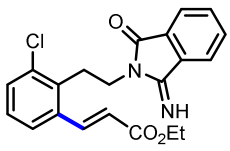
Cl
O
N
NH
CO2Et

2b

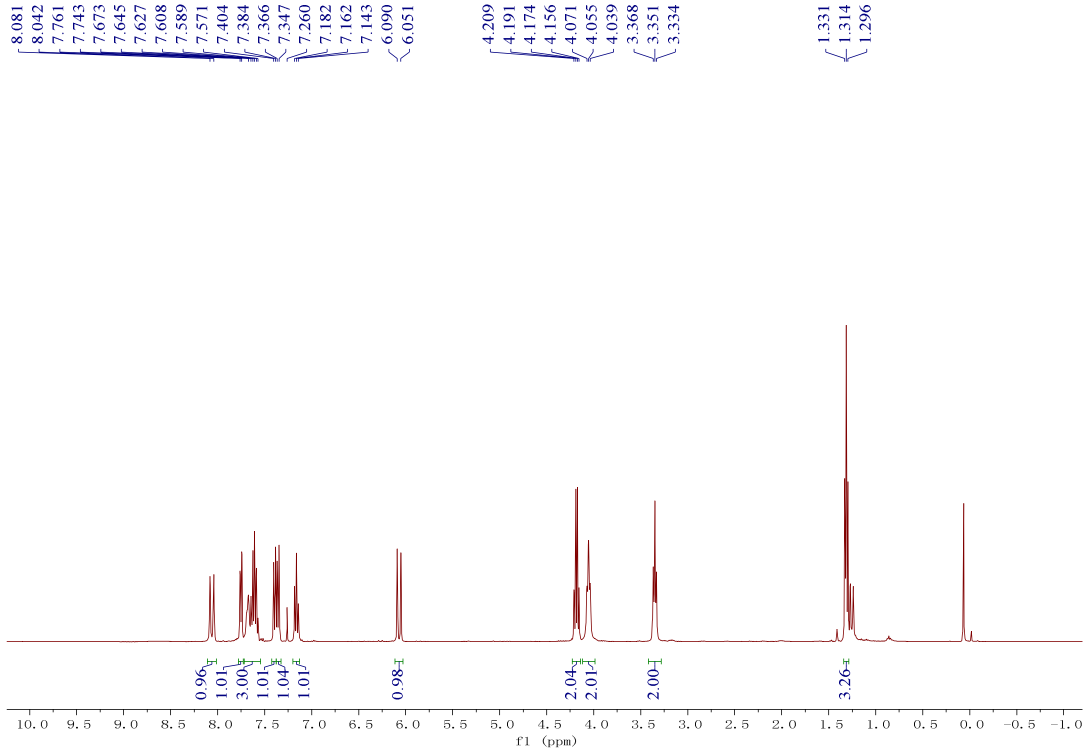
8.081
8.042
7.761
7.743
7.673
7.645
7.627
7.608
7.589
7.571
7.404
7.384
7.366
7.347
7.260
7.182
7.162
7.143
6.090
6.051
4.209
4.191
4.174
4.156
4.071
4.055
4.039
3.368
3.351
3.334
1.331
1.314
1.296
0.96
1.01
3.00
1.01
1.04
1.01
0.98
2.04
2.01
2.00
3.26
10.0 9.5 9.0 8.5 8.0 7.5 7.0 6.5 6.0 5.5 5.0 4.5 4.0 3.5 3.0 2.5 2.0 1.5 1.0 0.5 0.0 -0.5 -1.0
f1 (ppm)

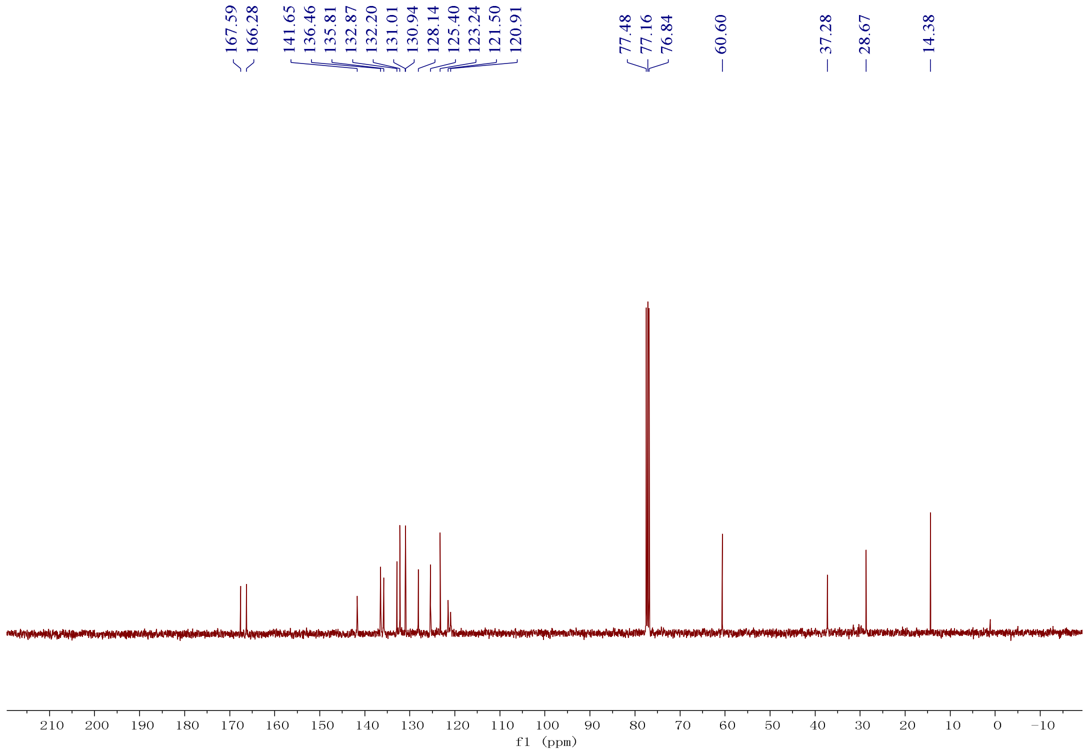
167.59
166.28
141.65
136.46
135.81
132.87
132.20
131.01
130.94
128.14
125.40
123.24
121.50
120.91
77.48
77.16
76.84
60.60
37.28
28.67
14.38
210 200 190 180 170 160 150 140 130 120 110 100 90 80 70 60 50 40 30 20 10 0 -10
f1 (ppm)

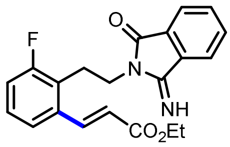

F
O
N
NH
CO_2Et

2c

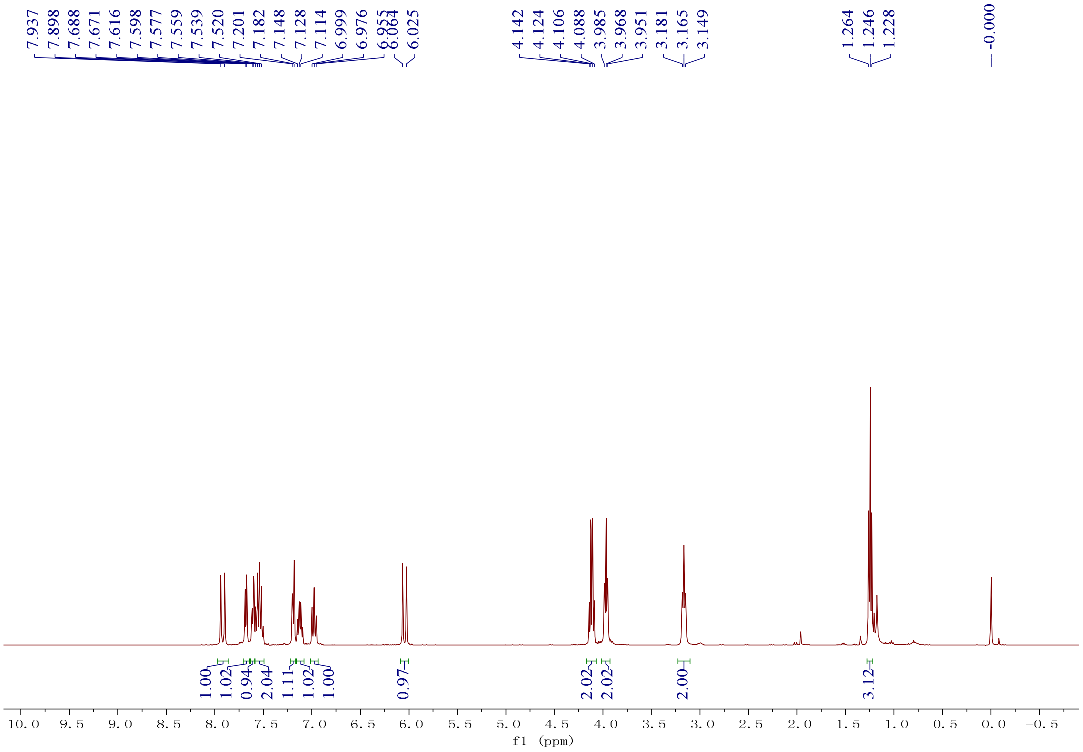

7.937
7.898
7.688
7.671
7.616
7.598
7.577
7.559
7.539
7.520
7.201
7.182
7.148
7.128
7.114
6.999
6.976
6.025
4.142
4.124
4.106
4.088
3.985
3.968
3.951
3.181
3.165
3.149
1.264
1.246
1.228
-0.000
1.00
1.02
0.94
2.04
1.11
1.02
1.00
0.97
2.02
2.02
2.00
3.12
10.0 9.5 9.0 8.5 8.0 7.5 7.0 6.5 6.0 5.5 5.0 4.5 4.0 3.5 3.0 2.5 2.0 1.5 1.0 0.5 0.0 -0.5
f1 (ppm)

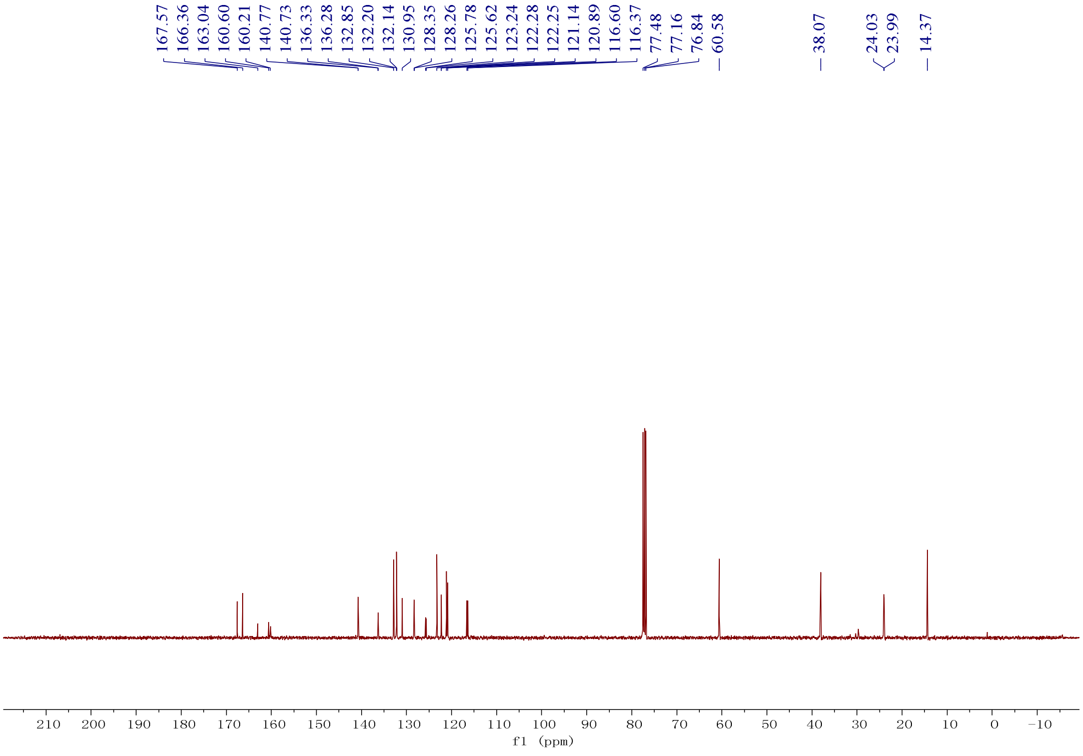

167.57
166.36
163.04
160.60
160.21
140.77
140.73
136.33
136.28
132.85
132.20
132.14
130.95
128.35
128.26
125.78
125.62
123.24
122.28
122.25
121.14
120.89
116.60
116.37
77.48
77.16
76.84
60.58
38.07
24.03
23.99
14.37
210 200 190 180 170 160 150 140 130 120 110 100 90 80 70 60 50 40 30 20 10 0 -10
f1 (ppm)

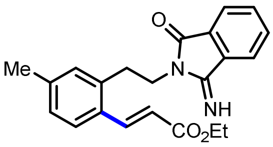
Me
O
N
NH
CO2Et

2d$_{mono}$

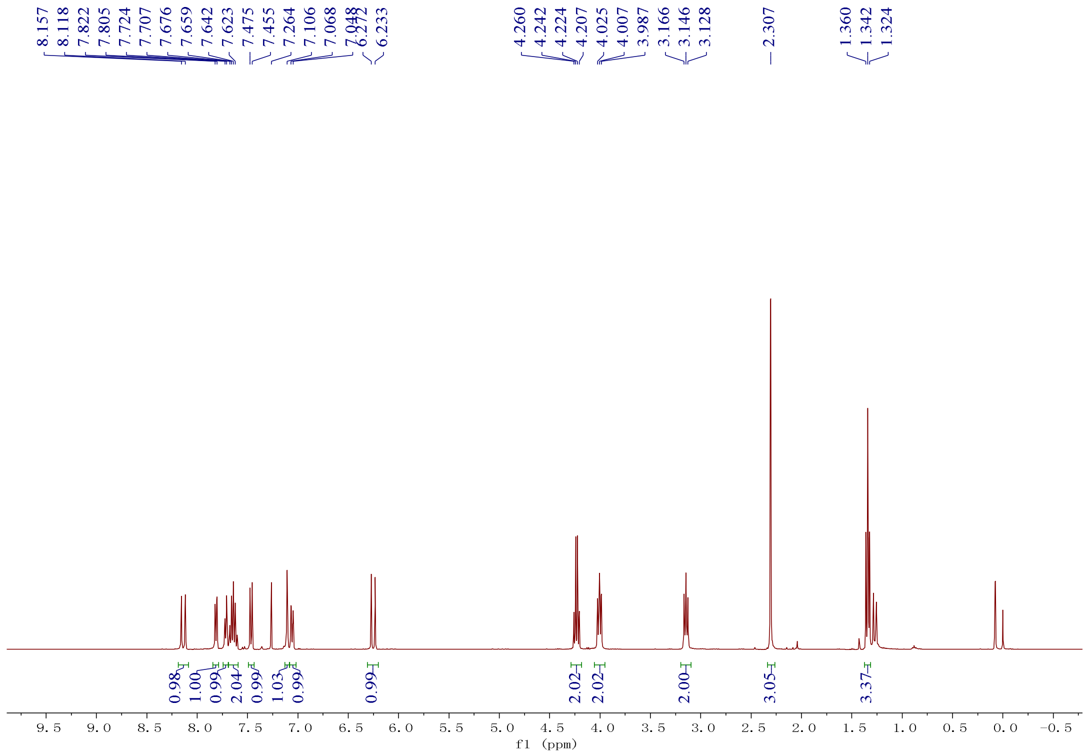
8.157
8.118
7.822
7.805
7.724
7.707
7.676
7.659
7.642
7.623
7.475
7.455
7.264
7.106
7.068
7.048
6.272
6.233
4.260
4.242
4.224
4.207
4.025
4.007
3.987
3.166
3.146
3.128
2.307
1.360
1.342
1.324
0.98
1.00
0.99
2.04
0.99
1.03
0.99
0.99
2.02
2.02
2.00
3.05
3.37
9.5 9.0 8.5 8.0 7.5 7.0 6.5 6.0 5.5 5.0 4.5 4.0 3.5 3.0 2.5 2.0 1.5 1.0 0.5 0.0 -0.5
f1 (ppm)

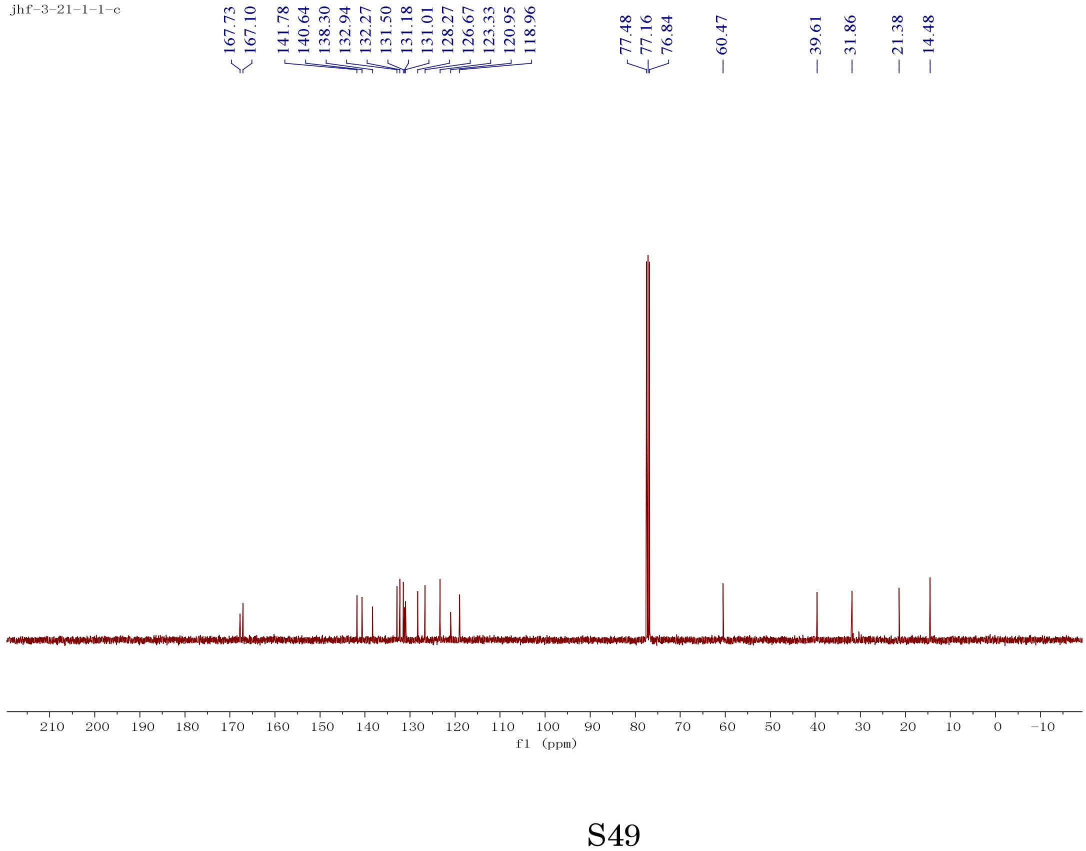
jhf-3-21-1-1-c
167.73
167.10
141.78
140.64
138.30
132.94
132.27
131.50
131.18
131.01
128.27
126.67
123.33
120.95
118.96
77.48
77.16
76.84
60.47
39.61
31.86
21.38
14.48
210 200 190 180 170 160 150 140 130 120 110 100 90 80 70 60 50 40 30 20 10 0 -10
f1 (ppm)

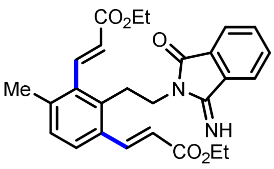

CO2Et
O
Me
N
NH
CO2Et

2d$_{(o,o')di}$

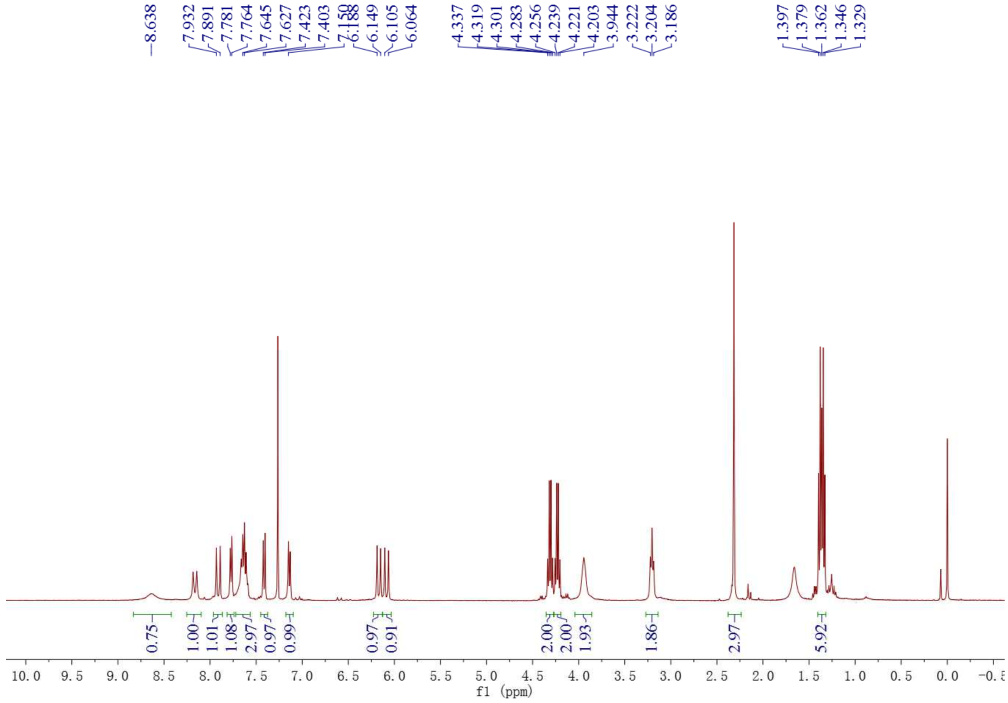

8.638
7.932
7.891
7.781
7.764
7.645
7.627
7.423
7.403
7.150
6.188
6.149
6.105
6.064
4.337
4.319
4.301
4.283
4.256
4.239
4.221
4.203
3.944
3.222
3.204
3.186
1.397
1.379
1.362
1.346
1.329
0.75
1.00
1.01
1.08
2.97
0.97
0.99
0.97
0.91
2.00
2.00
1.93
1.86
2.97
5.92
f1 (ppm)

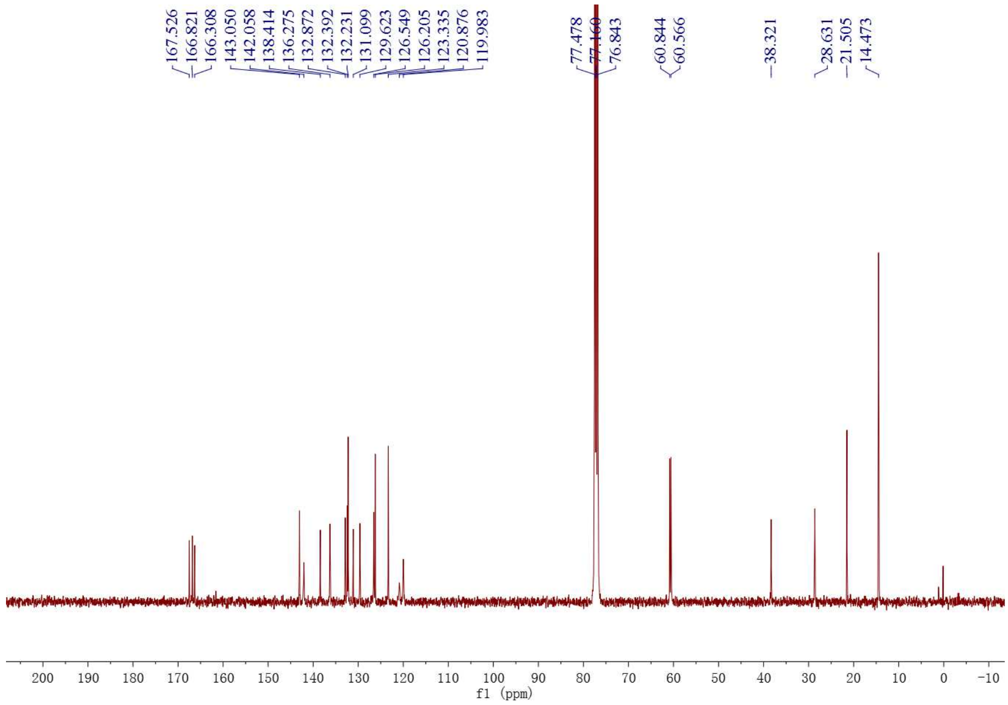

167.526
166.821
166.308
143.050
142.058
138.414
136.275
132.872
132.392
132.231
131.099
129.623
126.549
126.205
123.335
120.876
119.983
77.478
77.160
76.843
60.844
60.566
38.321
28.631
21.505
14.473
f1 (ppm)

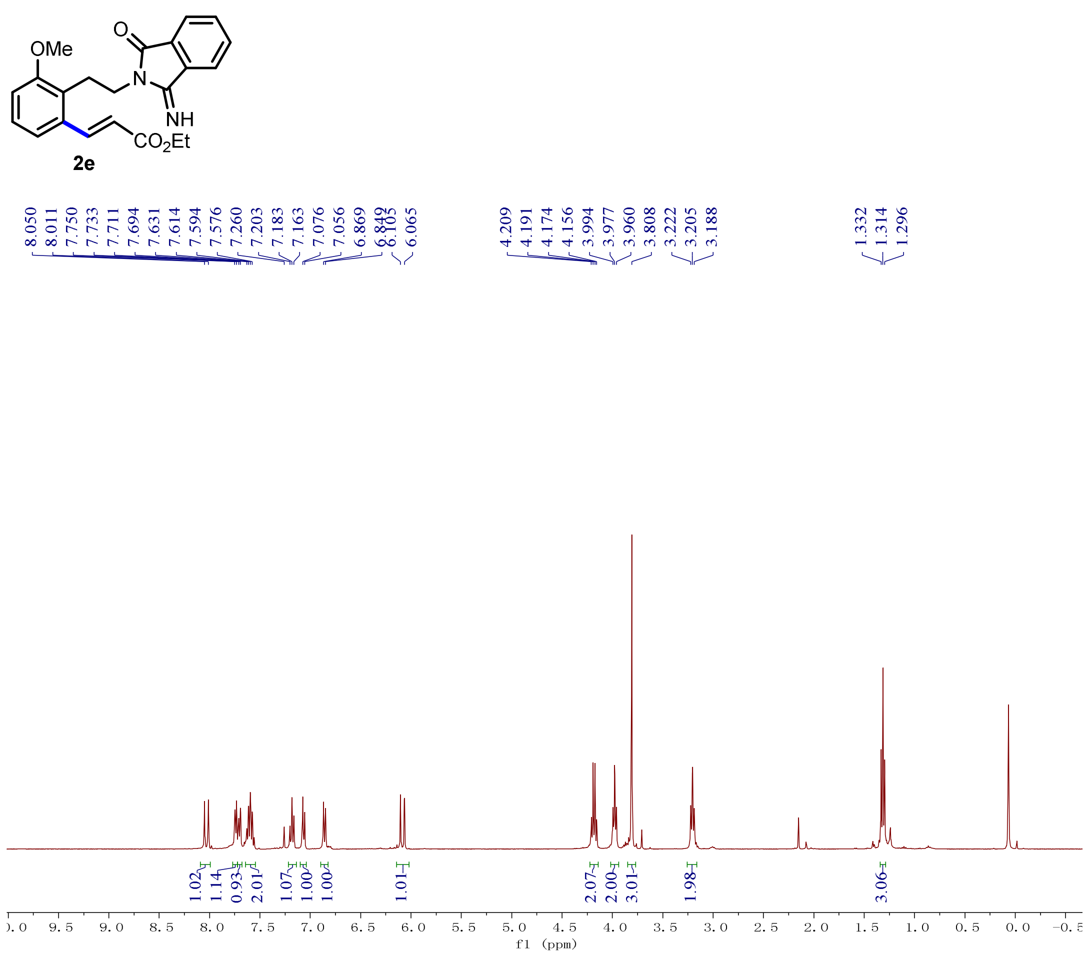

OMe
O
N
NH
CO2Et
2e
8.050
8.011
7.750
7.733
7.711
7.694
7.631
7.614
7.594
7.576
7.260
7.203
7.183
7.163
7.076
7.056
6.869
6.849
6.105
6.065
4.209
4.191
4.174
4.156
3.994
3.977
3.960
3.808
3.222
3.205
3.188
1.332
1.314
1.296
1.02
1.14
0.93
2.01
1.07
1.00
1.00
1.01
2.07
2.00
3.01
1.98
3.06
f1 (ppm)

jhf-4-3-1-p

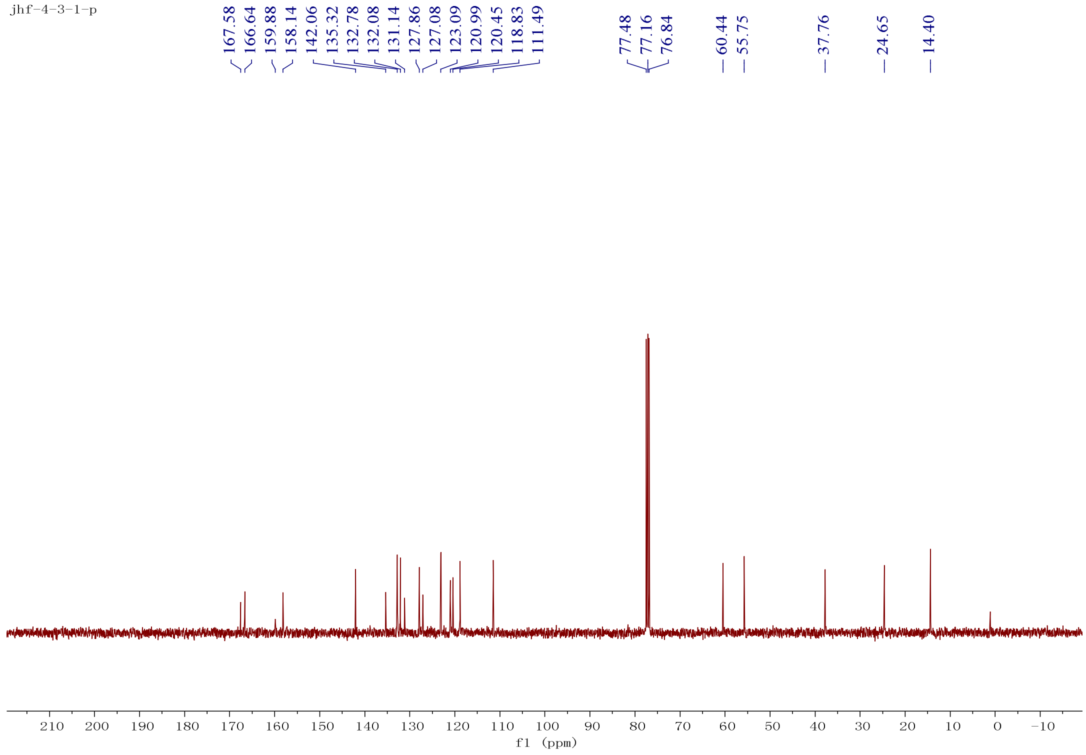

167.58
166.64
159.88
158.14
142.06
135.32
132.78
132.08
131.14
127.86
127.08
123.09
120.99
120.45
118.83
111.49
77.48
77.16
76.84
60.44
55.75
37.76
24.65
14.40
f1 (ppm)

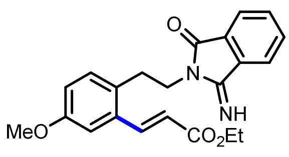
O
N
NH
MeO
CO2Et

$2f_{mono}$

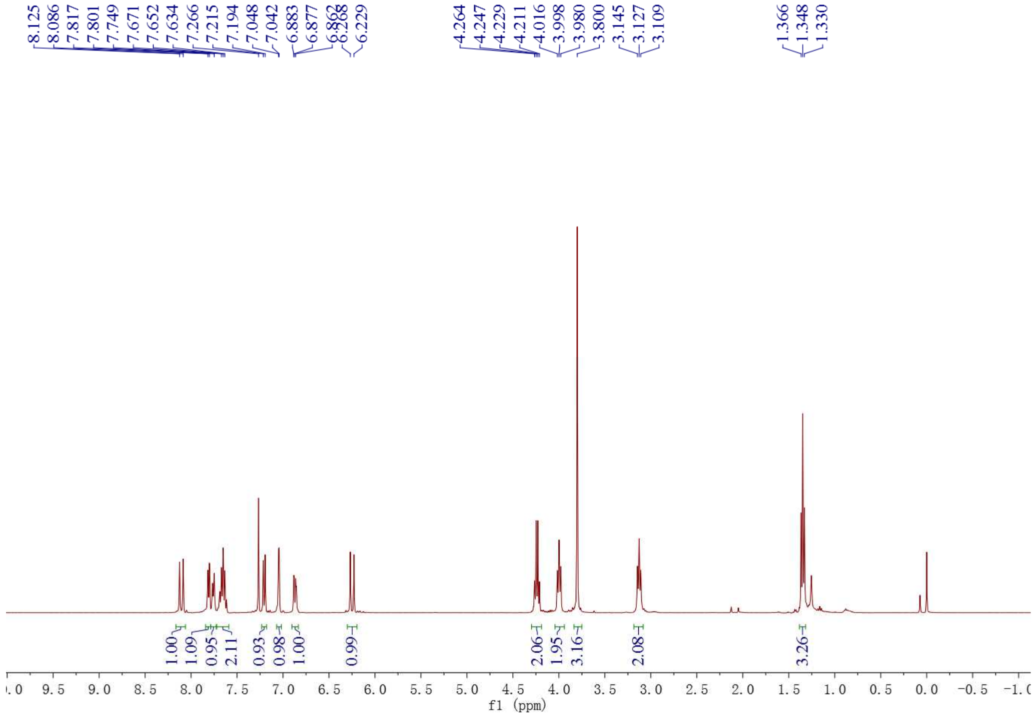
8.125
8.086
7.817
7.801
7.749
7.671
7.652
7.634
7.266
7.215
7.194
7.048
7.042
6.883
6.877
6.862
6.268
6.229
4.264
4.247
4.229
4.211
4.016
3.998
3.980
3.800
3.145
3.127
3.109
1.366
1.348
1.330
1.00
1.09
0.95
2.11
0.93
0.98
1.00
0.99
2.06
1.95
3.16
2.08
3.26
9.5 9.0 8.5 8.0 7.5 7.0 6.5 6.0 5.5 5.0 4.5 4.0 3.5 3.0 2.5 2.0 1.5 1.0 0.5 0.0 −0.5
f1 (ppm)

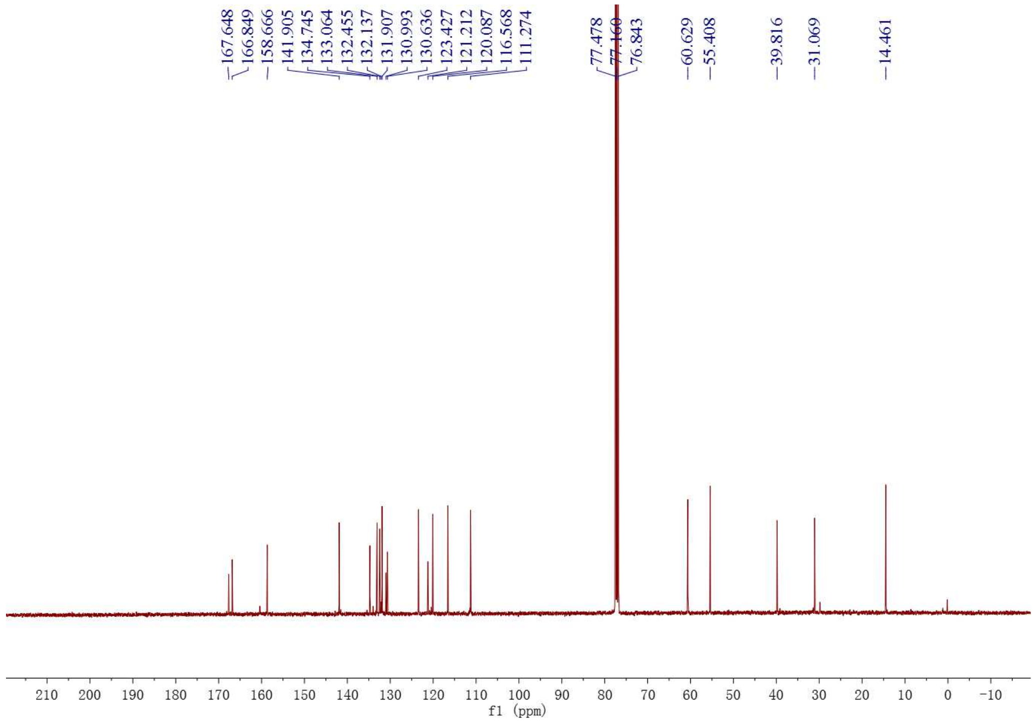
167.648
166.849
158.666
141.905
134.745
133.064
132.455
132.137
131.907
130.993
130.636
123.427
121.212
120.087
116.568
111.274
77.478
77.160
76.843
60.629
55.408
39.816
31.069
14.461
210 200 190 180 170 160 150 140 130 120 110 100 90 80 70 60 50 40 30 20 10 0 −10
f1 (ppm)

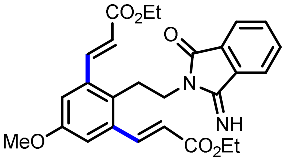
CO2Et
O
N
NH
MeO
CO2Et

2f(o,o')di

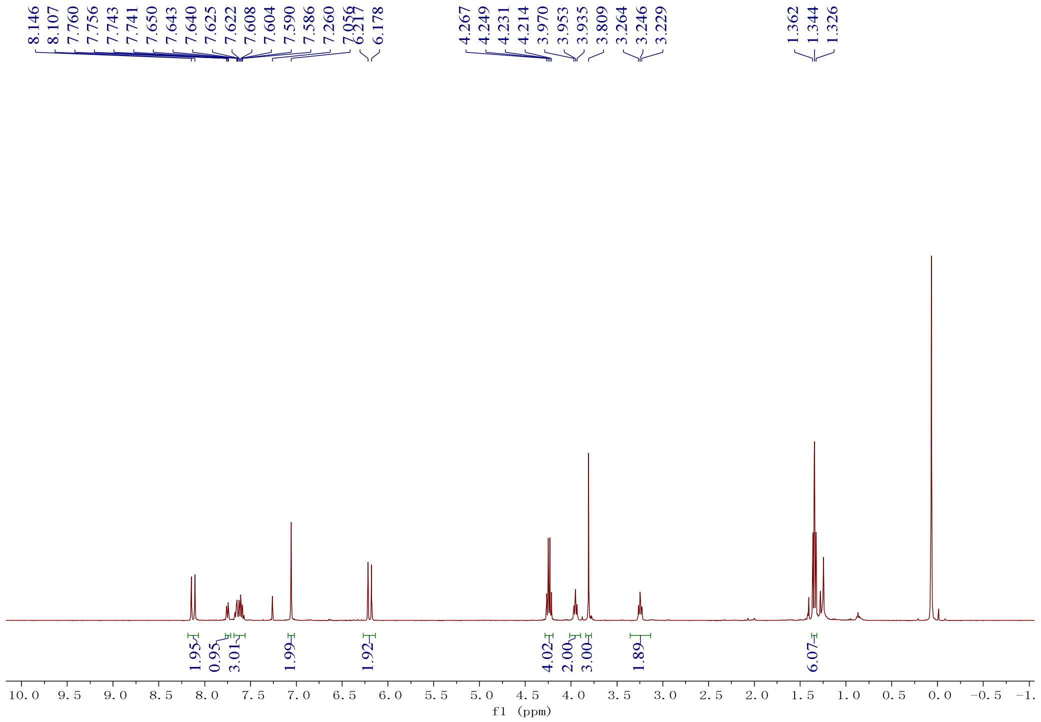
8.146
8.107
7.760
7.756
7.743
7.741
7.650
7.643
7.640
7.625
7.622
7.608
7.604
7.590
7.586
7.260
7.056
6.217
6.178
4.267
4.249
4.231
4.214
3.970
3.953
3.935
3.809
3.264
3.246
3.229
1.362
1.344
1.326
1.95
0.95
3.01
1.99
1.92
4.02
2.00
3.00
1.89
6.07
f1 (ppm)

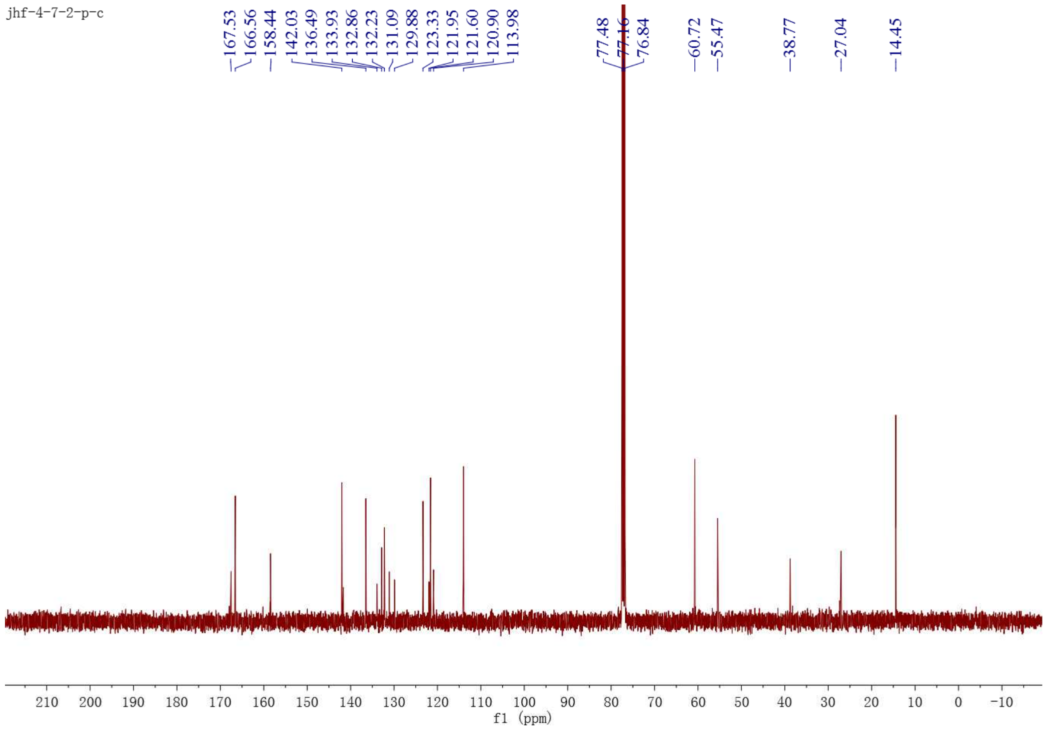
jhf-4-7-2-p-c
167.53
166.56
158.44
142.03
136.49
133.93
132.86
132.23
131.09
129.88
123.33
121.95
121.60
120.90
113.98
77.48
77.16
76.84
60.72
55.47
38.77
27.04
14.45
f1 (ppm)

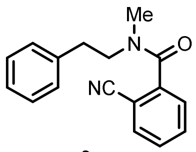
Me
N
O
NC

3a

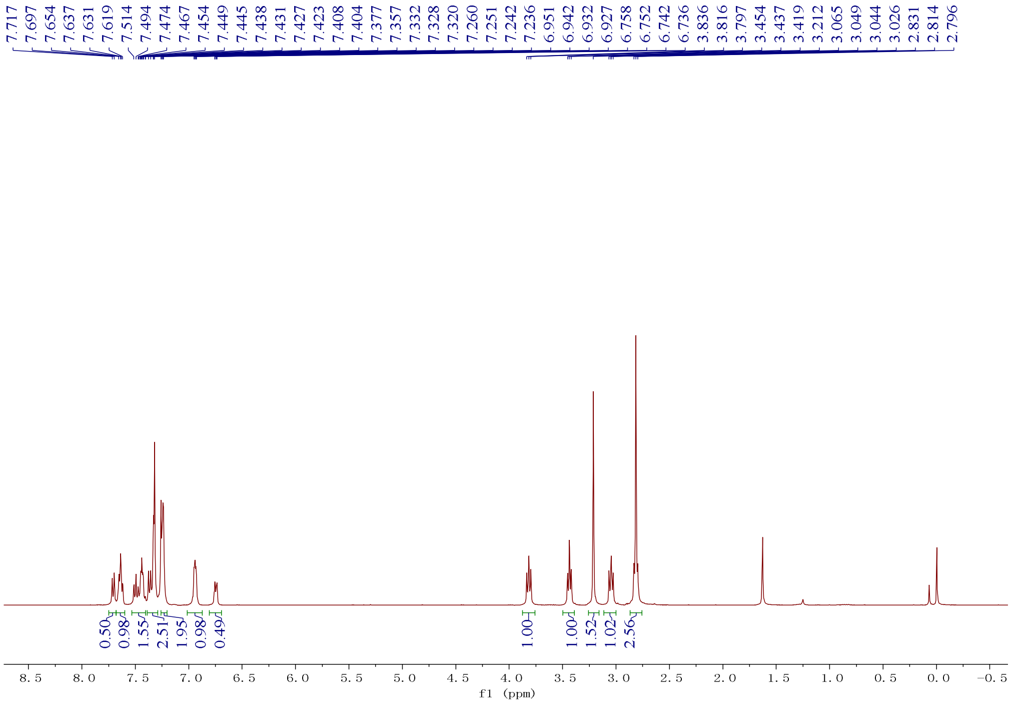
7.717
7.697
7.654
7.637
7.631
7.619
7.514
7.494
7.474
7.467
7.454
7.449
7.445
7.438
7.431
7.427
7.423
7.408
7.404
7.377
7.357
7.332
7.328
7.320
7.260
7.251
7.242
7.236
6.951
6.942
6.932
6.927
6.758
6.752
6.742
6.736
3.836
3.816
3.797
3.454
3.437
3.419
3.212
3.065
3.049
3.044
3.026
2.831
2.814
2.796
0.50
0.98
1.55
2.51
1.95
0.98
0.49
1.00
1.00
1.52
1.02
2.56
8.5 8.0 7.5 7.0 6.5 6.0 5.5 5.0 4.5 4.0 3.5 3.0 2.5 2.0 1.5 1.0 0.5 0.0 -0.5
f1 (ppm)

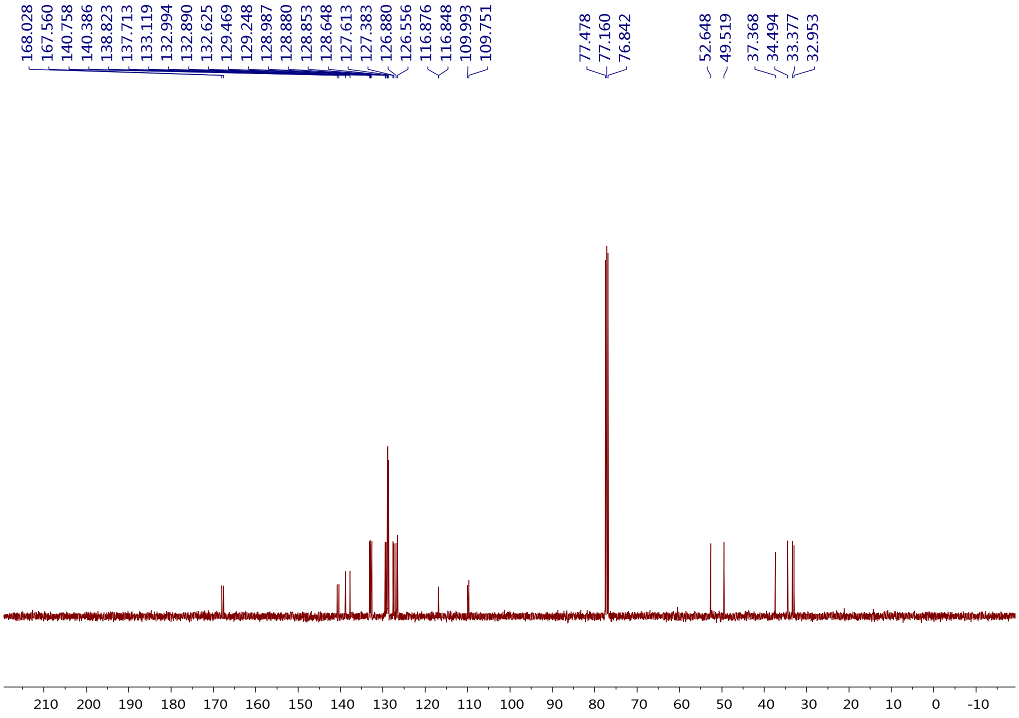
168.028
167.560
140.758
140.386
138.823
137.713
133.119
132.994
132.890
132.625
129.469
129.248
128.987
128.880
128.853
128.648
127.613
127.383
126.880
126.556
116.876
116.848
109.993
109.751
77.478
77.160
76.842
52.648
49.519
37.368
34.494
33.377
32.953
210 200 190 180 170 160 150 140 130 120 110 100 90 80 70 60 50 40 30 20 10 0 -10

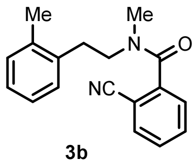
Me
Me
N
O
NC

3b

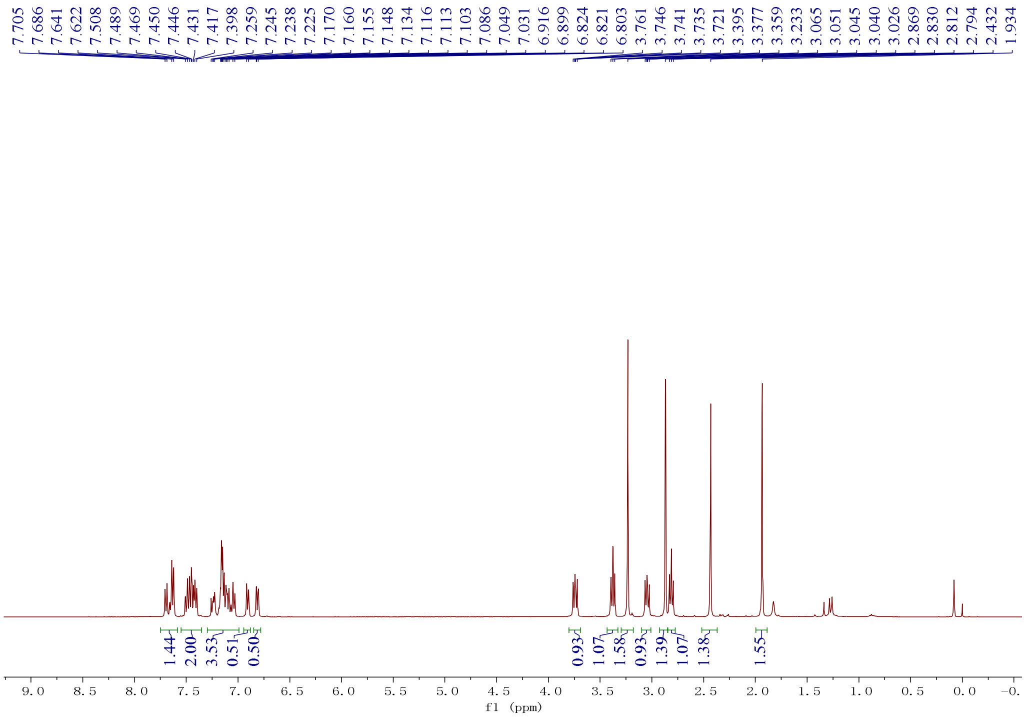
7.705
7.686
7.641
7.622
7.508
7.489
7.469
7.450
7.446
7.431
7.417
7.398
7.259
7.245
7.238
7.225
7.170
7.160
7.155
7.148
7.134
7.116
7.113
7.103
7.086
7.049
7.031
6.916
6.899
6.824
6.821
6.803
3.761
3.746
3.741
3.735
3.721
3.395
3.377
3.359
3.233
3.065
3.051
3.045
3.040
3.026
2.869
2.830
2.812
2.794
2.432
1.934
1.44
2.00
3.53
0.51
0.50
0.93
1.07
1.58
0.93
1.39
1.07
1.38
1.55
9.0
8.5
8.0
7.5
7.0
6.5
6.0
5.5
5.0
4.5
4.0
3.5
3.0
2.5
2.0
1.5
1.0
0.5
0.0
-0.
f1 (ppm)

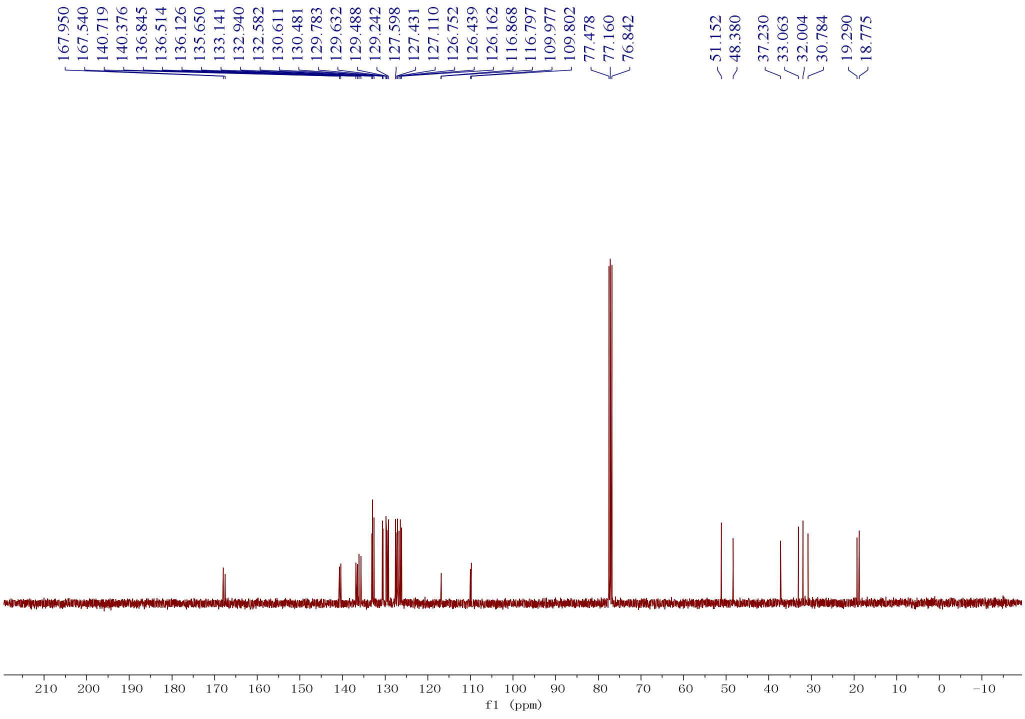
167.950
167.540
140.719
140.376
136.845
136.514
136.126
135.650
133.141
132.940
132.582
130.611
130.481
129.783
129.632
129.488
129.242
127.598
127.431
127.110
126.752
126.439
126.162
116.868
116.797
109.977
109.802
77.478
77.160
76.842
51.152
48.380
37.230
33.063
32.004
30.784
19.290
18.775
210
200
190
180
170
160
150
140
130
120
110
100
90
80
70
60
50
40
30
20
10
0
-10
f1 (ppm)

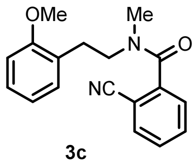
OMe
Me
N
O
NC

3c

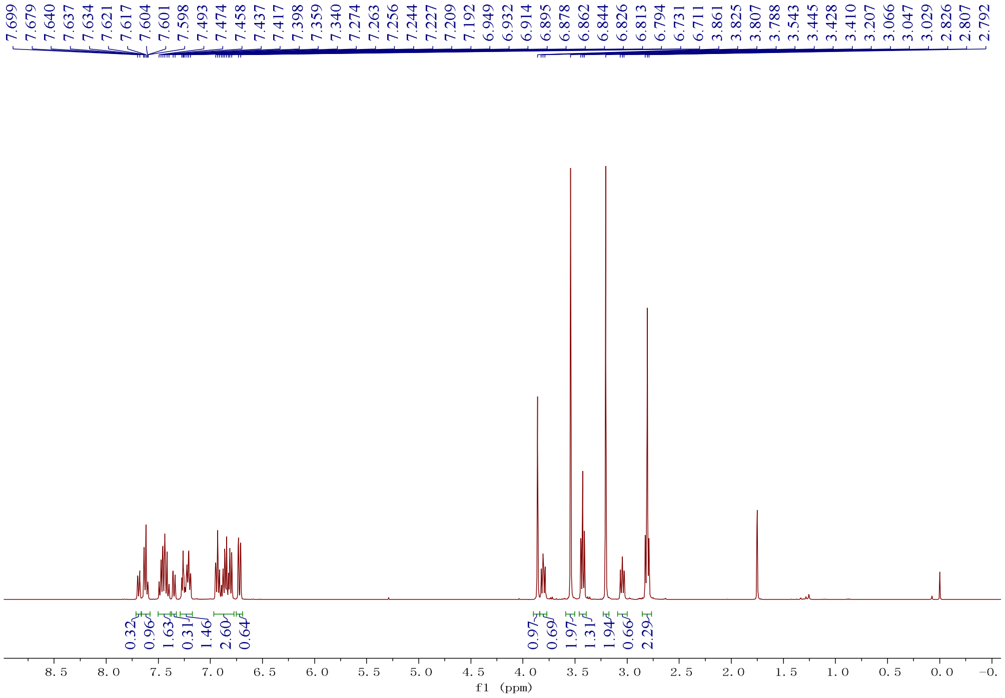
7.699
7.679
7.640
7.637
7.634
7.621
7.617
7.604
7.601
7.598
7.493
7.474
7.458
7.437
7.417
7.398
7.359
7.340
7.274
7.263
7.256
7.244
7.227
7.209
7.192
6.949
6.932
6.914
6.895
6.878
6.862
6.844
6.826
6.813
6.794
6.731
6.711
3.861
3.825
3.807
3.788
3.543
3.445
3.428
3.410
3.207
3.066
3.047
3.029
2.826
2.807
2.792
0.32
0.96
1.63
0.31
1.46
2.60
0.64
0.97
0.69
1.97
1.31
1.94
0.66
2.29
f1 (ppm)

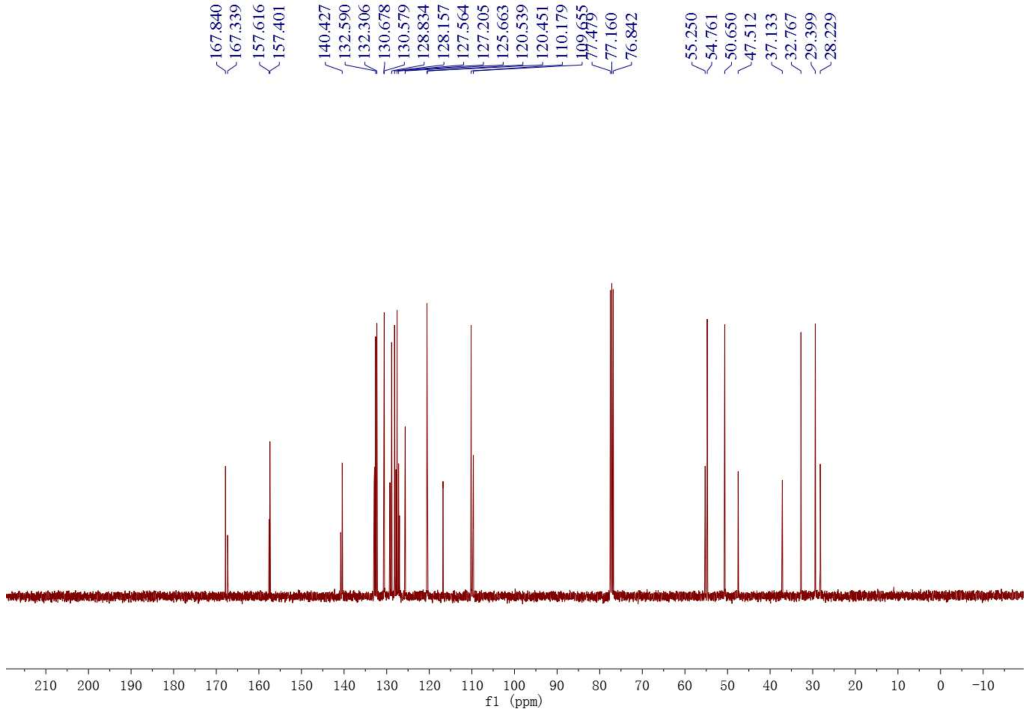
167.840
167.339
157.616
157.401
140.427
132.590
132.306
130.678
130.579
128.834
128.157
127.564
127.205
125.663
120.539
120.451
110.179
109.655
77.479
77.160
76.842
55.250
54.761
50.650
47.512
37.133
32.767
29.399
28.229
f1 (ppm)

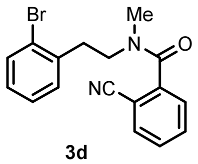
Br
Me
N
O
NC

3d

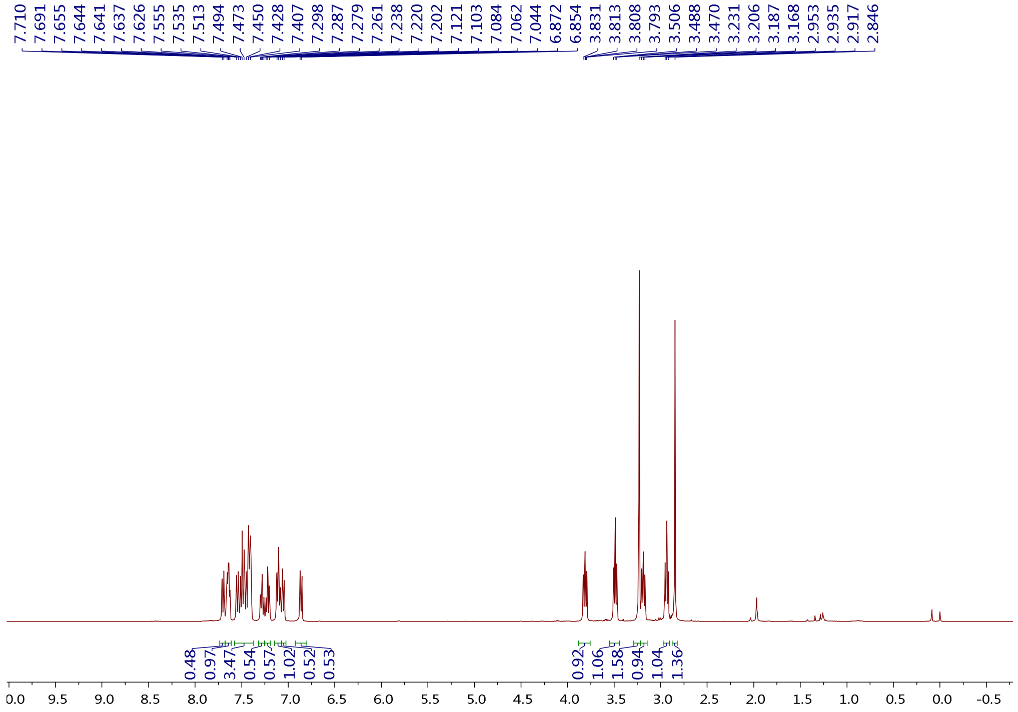
7.710
7.691
7.655
7.644
7.641
7.637
7.626
7.555
7.535
7.513
7.494
7.473
7.450
7.428
7.407
7.298
7.287
7.279
7.261
7.238
7.220
7.202
7.121
7.103
7.084
7.062
7.044
6.872
6.854
3.831
3.813
3.808
3.793
3.506
3.488
3.470
3.231
3.206
3.187
3.168
2.953
2.935
2.917
2.846
0.48
0.97
3.47
0.54
0.57
1.02
0.52
0.53
0.92
1.06
1.58
0.94
1.04
1.36
0.0 9.5 9.0 8.5 8.0 7.5 7.0 6.5 6.0 5.5 5.0 4.5 4.0 3.5 3.0 2.5 2.0 1.5 1.0 0.5 0.0 -0.5

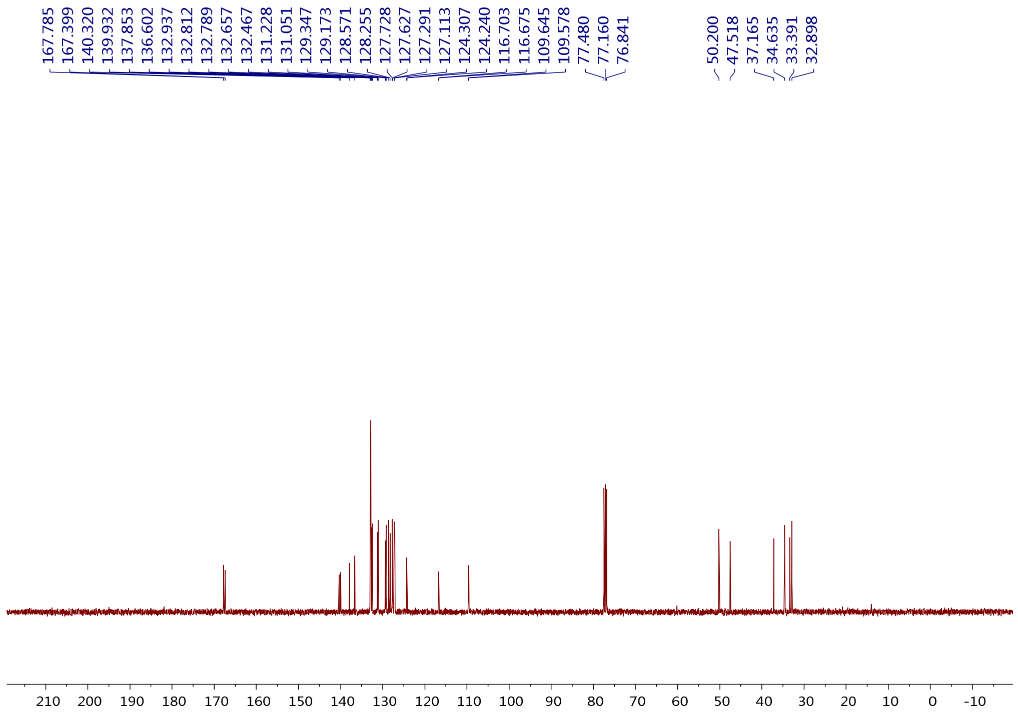
167.785
167.399
140.320
139.932
137.853
136.602
132.937
132.812
132.789
132.657
132.467
131.228
131.051
129.347
129.173
128.571
128.255
127.728
127.627
127.291
127.113
124.307
124.240
116.703
116.675
109.645
109.578
77.480
77.160
76.841
50.200
47.518
37.165
34.635
33.391
32.898
210 200 190 180 170 160 150 140 130 120 110 100 90 80 70 60 50 40 30 20 10 0 -10

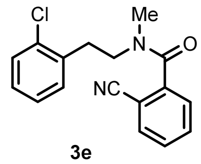
Cl
Me
N
O
NC

3e

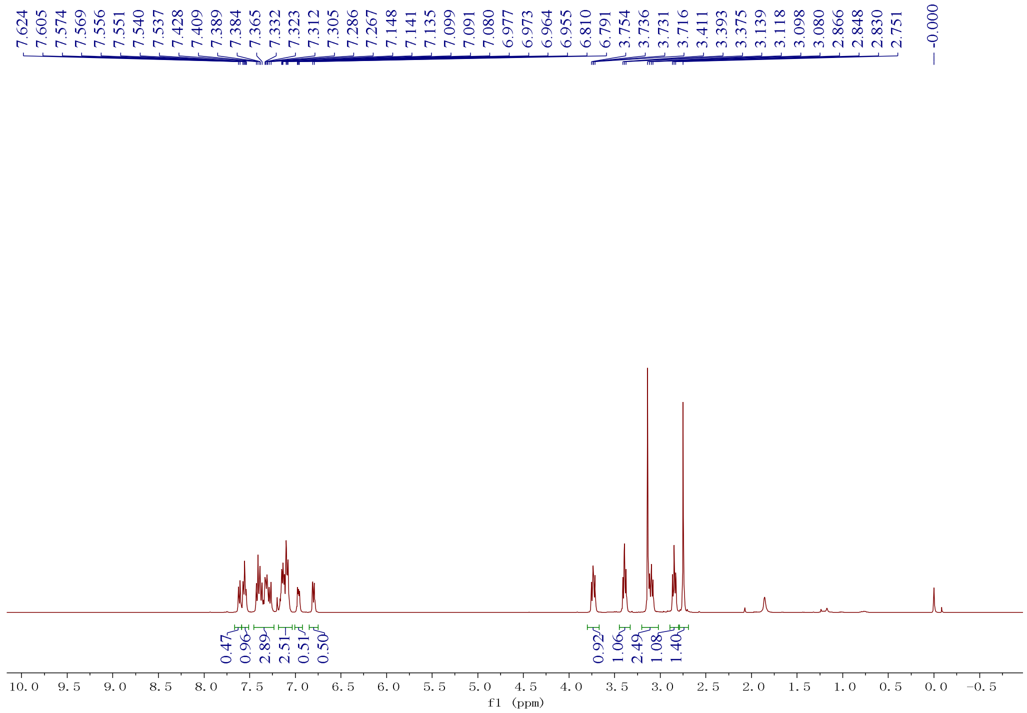
7.624 7.605 7.574 7.569 7.556 7.551 7.540 7.537 7.428 7.409 7.389 7.384 7.365 7.332 7.323 7.312 7.305 7.286 7.267 7.148 7.141 7.135 7.099 7.091 7.080 6.977 6.973 6.964 6.955 6.810 6.791 3.754 3.736 3.731 3.716 3.411 3.393 3.375 3.139 3.118 3.098 3.080 2.866 2.848 2.830 2.751
-0.000
0.47 0.96 2.89 2.51 0.51 0.50
0.92 1.06 2.49 1.08 1.40
10.0 9.5 9.0 8.5 8.0 7.5 7.0 6.5 6.0 5.5 5.0 4.5 4.0 3.5 3.0 2.5 2.0 1.5 1.0 0.5 0.0 -0.5
f1 (ppm)

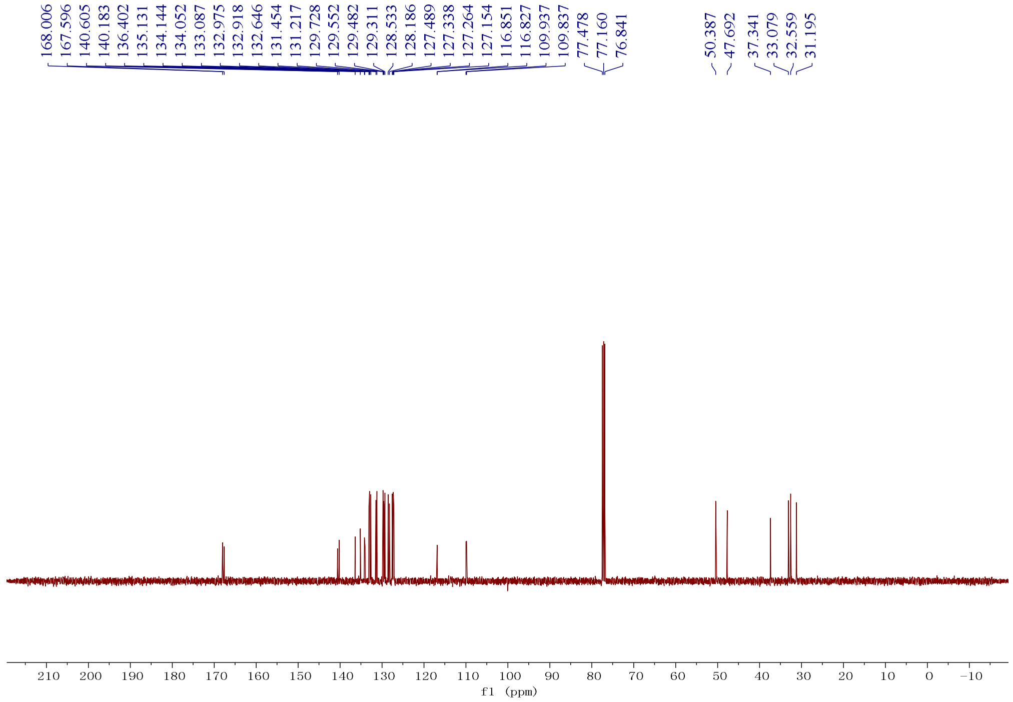
168.006 167.596 140.605 140.183 136.402 135.131 134.144 134.052 133.087 132.975 132.918 132.646 131.454 131.217 129.728 129.552 129.482 129.311 128.533 128.186 127.489 127.338 127.264 127.154 116.851 116.827 109.937 109.837
77.478 77.160 76.841
50.387 47.692
37.341 33.079 32.559 31.195
210 200 190 180 170 160 150 140 130 120 110 100 90 80 70 60 50 40 30 20 10 0 -10
f1 (ppm)

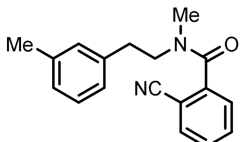
Me
Me
N
O
NC

3f

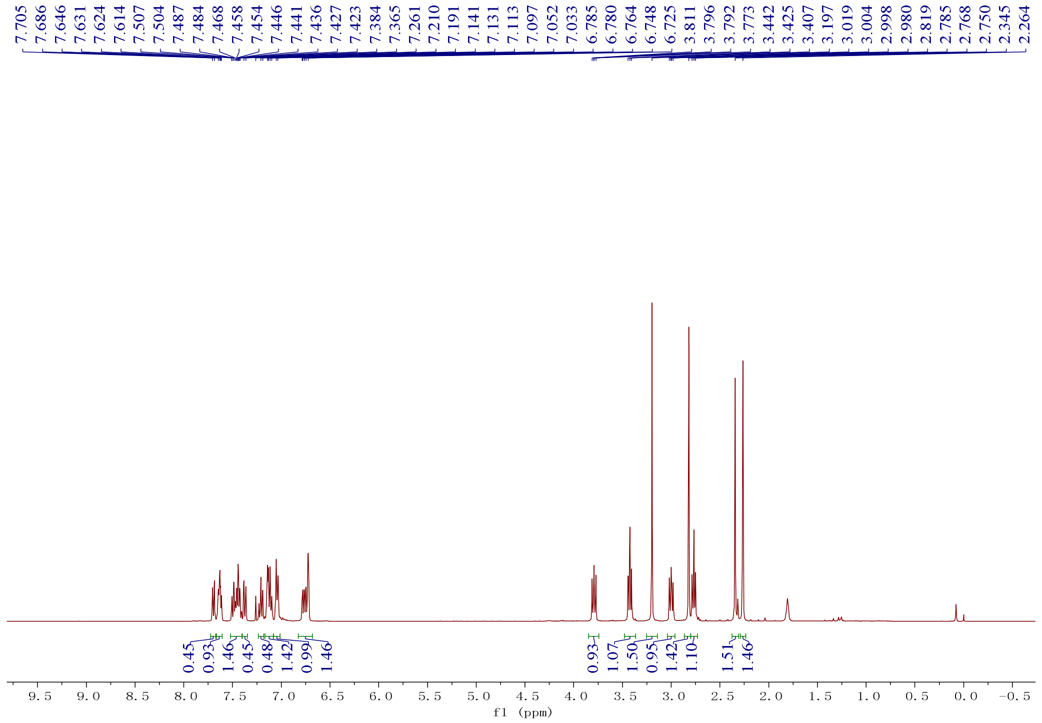
7.705
7.686
7.646
7.631
7.624
7.614
7.507
7.504
7.487
7.484
7.468
7.458
7.454
7.446
7.441
7.436
7.427
7.423
7.384
7.365
7.261
7.210
7.191
7.141
7.131
7.113
7.097
7.052
7.033
6.785
6.780
6.764
6.748
6.725
3.811
3.796
3.792
3.773
3.442
3.425
3.407
3.197
3.019
3.004
2.998
2.980
2.819
2.785
2.768
2.750
2.345
2.264
0.45
0.93
1.46
0.45
0.48
1.42
0.99
1.46
0.93
1.07
1.50
0.95
1.42
1.10
1.51
1.46
9.5 9.0 8.5 8.0 7.5 7.0 6.5 6.0 5.5 5.0 4.5 4.0 3.5 3.0 2.5 2.0 1.5 1.0 0.5 0.0 -0.5
f1 (ppm)

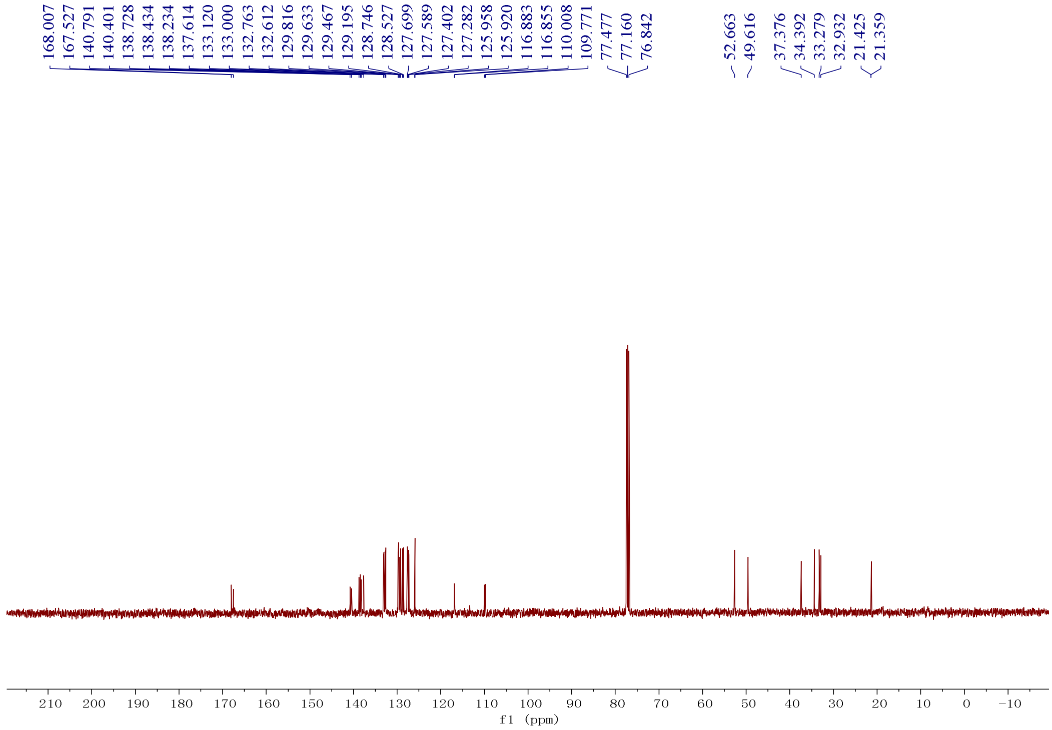
168.007
167.527
140.791
140.401
138.728
138.434
138.234
137.614
133.120
133.000
132.763
132.612
129.816
129.633
129.467
129.195
128.746
128.527
127.699
127.589
127.402
127.282
125.958
125.920
116.883
116.855
110.008
109.771
77.477
77.160
76.842
52.663
49.616
37.376
34.392
33.279
32.932
21.425
21.359
210 200 190 180 170 160 150 140 130 120 110 100 90 80 70 60 50 40 30 20 10 0 -10
f1 (ppm)

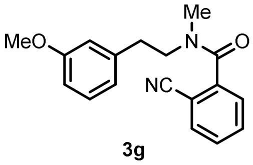
Me
MeO
N
O
NC

3g

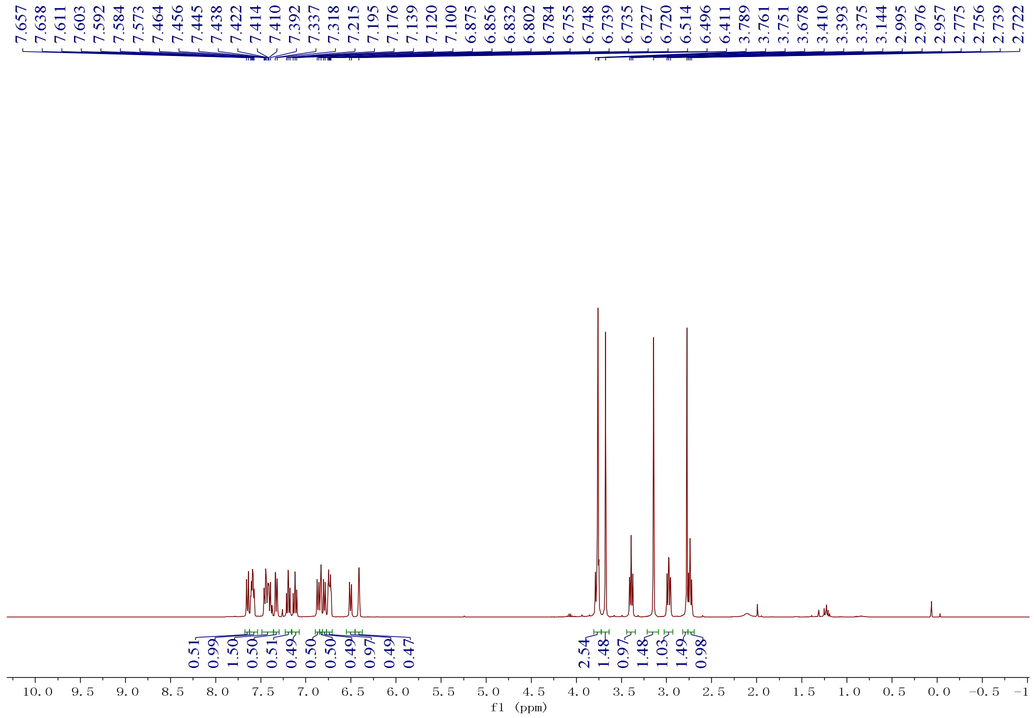
7.657 7.638 7.611 7.603 7.592 7.584 7.573 7.464 7.456 7.445 7.438 7.422 7.414 7.410 7.392 7.337 7.318 7.215 7.195 7.176 7.139 7.120 7.100 6.875 6.856 6.832 6.802 6.784 6.755 6.748 6.739 6.735 6.727 6.720 6.514 6.496 6.411 3.789 3.761 3.751 3.678 3.410 3.393 3.375 3.144 2.995 2.976 2.957 2.775 2.756 2.739 2.722
0.51 0.99 1.50 0.50 0.51 0.49 0.50 0.50 0.49 0.97 0.49 0.47
2.54 1.48 0.97 1.48 1.03 1.49 0.98
10.0 9.5 9.0 8.5 8.0 7.5 7.0 6.5 6.0 5.5 5.0 4.5 4.0 3.5 3.0 2.5 2.0 1.5 1.0 0.5 0.0 -0.5 -1
f1 (ppm)

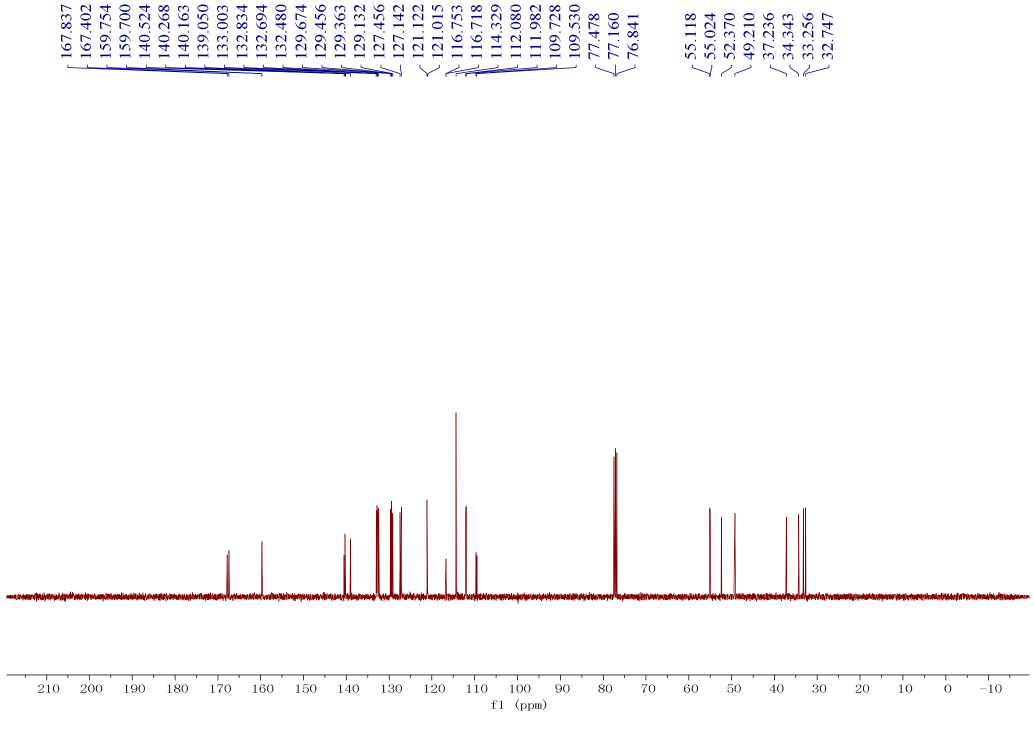
167.837 167.402 159.754 159.700 140.524 140.268 140.163 139.050 133.003 132.834 132.694 132.480 129.674 129.456 129.363 129.132 127.456 127.142 121.122 121.015 116.753 116.718 114.329 112.080 111.982 109.728 109.530 77.478 77.160 76.841
55.118 55.024 52.370 49.210 37.236 34.343 33.256 32.747
210 200 190 180 170 160 150 140 130 120 110 100 90 80 70 60 50 40 30 20 10 0 -10
f1 (ppm)

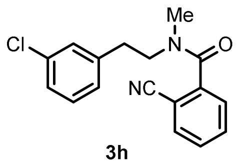
Me
N
O
Cl
NC

3h

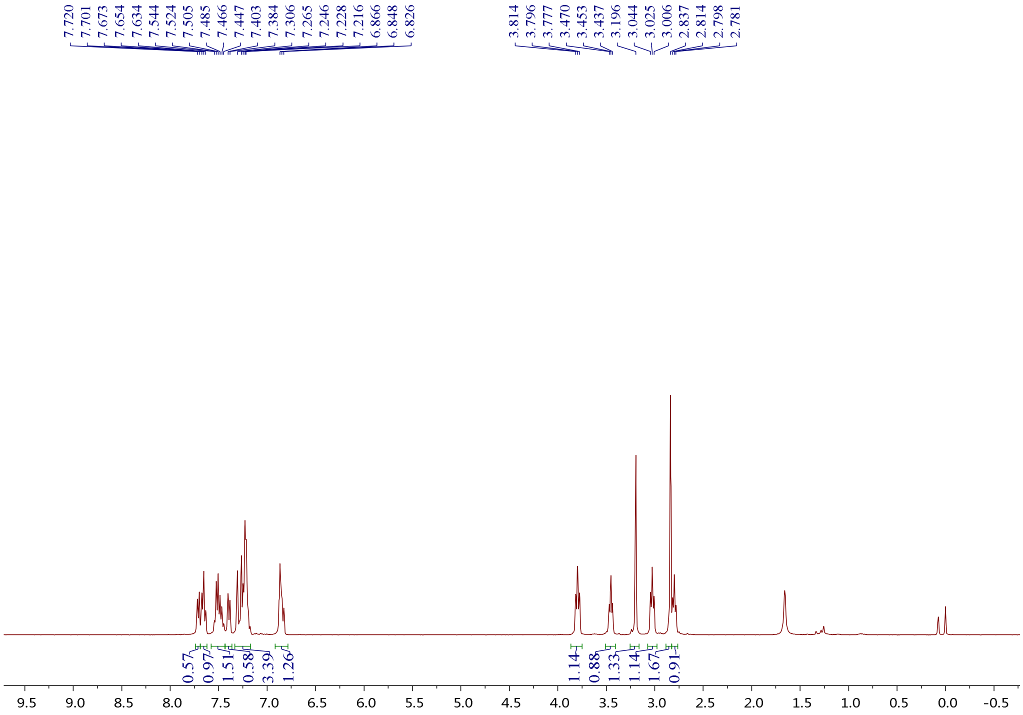
7.720
7.701
7.673
7.654
7.634
7.544
7.524
7.505
7.485
7.466
7.447
7.403
7.384
7.306
7.265
7.246
7.228
7.216
6.866
6.848
6.826
3.814
3.796
3.777
3.470
3.453
3.437
3.196
3.044
3.025
3.006
2.837
2.814
2.798
2.781
0.57
0.97
1.51
0.58
3.39
1.26
1.14
0.88
1.33
1.14
1.67
0.91
9.5 9.0 8.5 8.0 7.5 7.0 6.5 6.0 5.5 5.0 4.5 4.0 3.5 3.0 2.5 2.0 1.5 1.0 0.5 0.0 -0.5

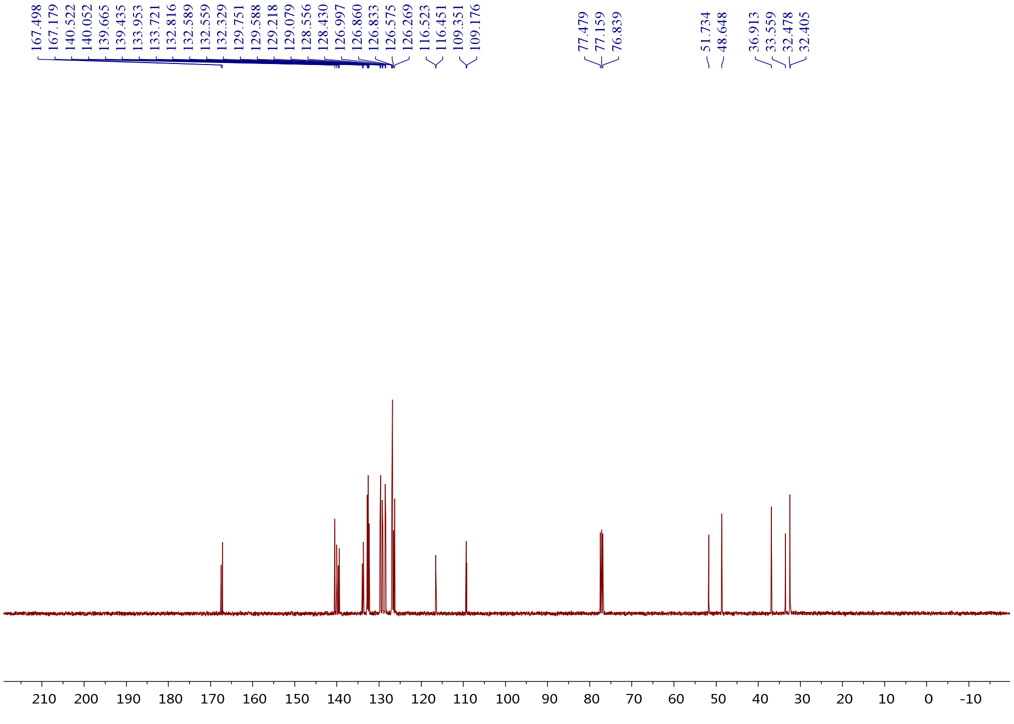
167.498
167.179
140.522
140.052
139.665
139.435
133.953
133.721
132.816
132.589
132.559
132.329
129.751
129.588
129.218
129.079
128.556
128.430
126.997
126.860
126.833
126.575
126.269
116.523
116.451
109.351
109.176
77.479
77.159
76.839
51.734
48.648
36.913
33.559
32.478
32.405
210 200 190 180 170 160 150 140 130 120 110 100 90 80 70 60 50 40 30 20 10 0 -10

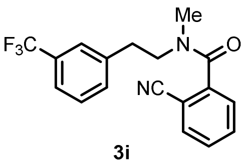
F3C
Me
N
O
NC

3i

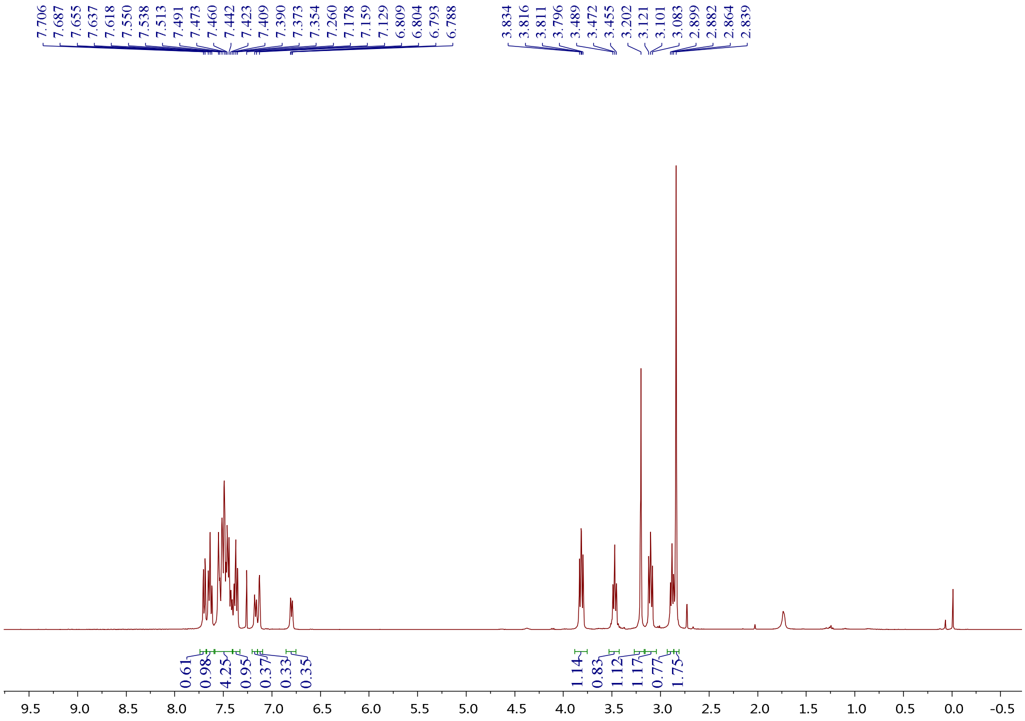
7.706
7.687
7.655
7.637
7.618
7.550
7.538
7.513
7.491
7.473
7.460
7.442
7.423
7.409
7.390
7.373
7.354
7.260
7.178
7.159
7.129
6.809
6.804
6.793
6.788
3.834
3.816
3.811
3.796
3.489
3.472
3.455
3.202
3.121
3.101
3.083
2.899
2.882
2.864
2.839
0.61
0.98
4.25
0.95
0.37
0.33
0.35
1.14
0.83
1.12
1.17
0.77
1.75
9.5
9.0
8.5
8.0
7.5
7.0
6.5
6.0
5.5
5.0
4.5
4.0
3.5
3.0
2.5
2.0
1.5
1.0
0.5
0.0
-0.5

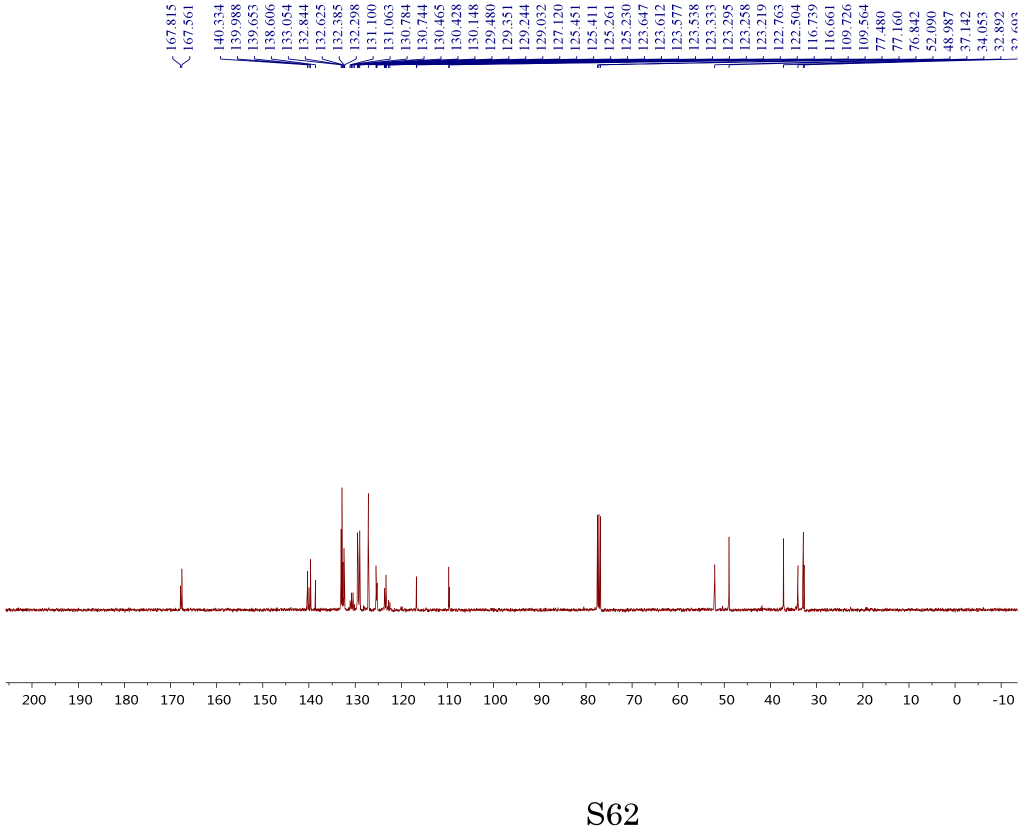
167.815
167.561
140.334
139.988
139.653
138.606
133.054
132.844
132.625
132.385
132.298
131.100
131.063
130.784
130.744
130.465
130.428
130.148
129.480
129.351
129.244
129.032
127.120
125.451
125.411
125.261
125.230
123.647
123.612
123.577
123.538
123.333
123.295
123.258
123.219
122.763
122.504
116.739
116.661
109.726
109.564
77.480
77.160
76.842
52.090
48.987
37.142
34.053
32.892
200
190
180
170
160
150
140
130
120
110
100
90
80
70
60
50
40
30
20
10
0
-10

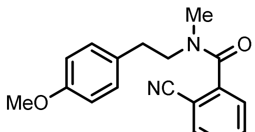
Me
N
O
NC
MeO

3j

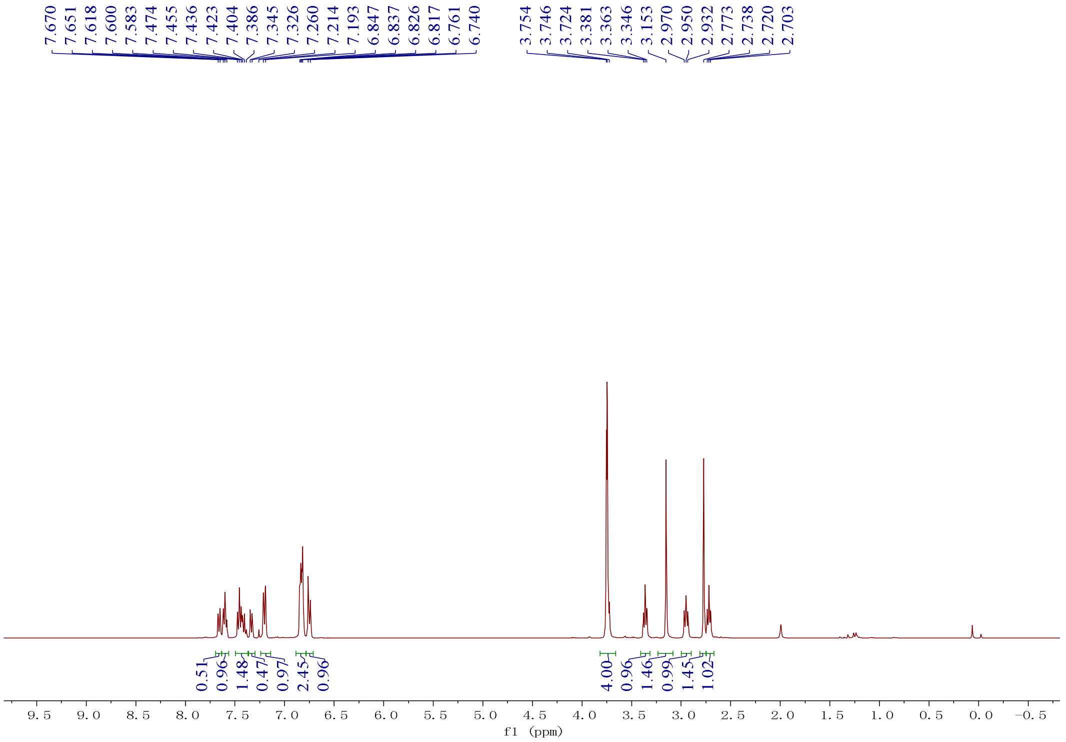
7.670
7.651
7.618
7.600
7.583
7.474
7.455
7.436
7.423
7.404
7.386
7.345
7.326
7.260
7.214
7.193
6.847
6.837
6.826
6.817
6.761
6.740
3.754
3.746
3.724
3.381
3.363
3.346
3.153
2.970
2.950
2.932
2.773
2.738
2.720
2.703
0.51
0.96
1.48
0.47
0.97
2.45
0.96
4.00
0.96
1.46
0.99
1.45
1.02
f1 (ppm)

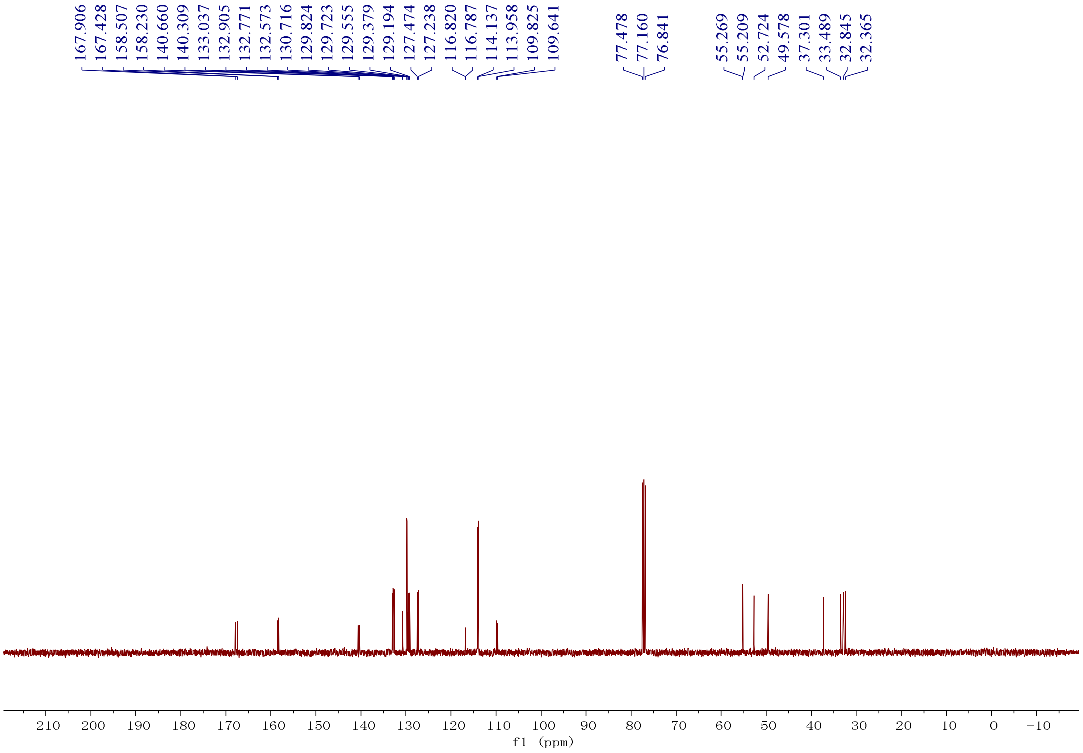
167.906
167.428
158.507
158.230
140.660
140.309
133.037
132.905
132.771
132.573
130.716
129.824
129.723
129.555
129.379
129.194
127.474
127.238
116.820
116.787
114.137
113.958
109.825
109.641
77.478
77.160
76.841
55.269
55.209
52.724
49.578
37.301
33.489
32.845
32.365
f1 (ppm)

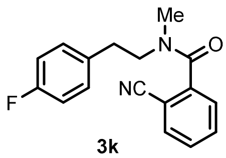

3k

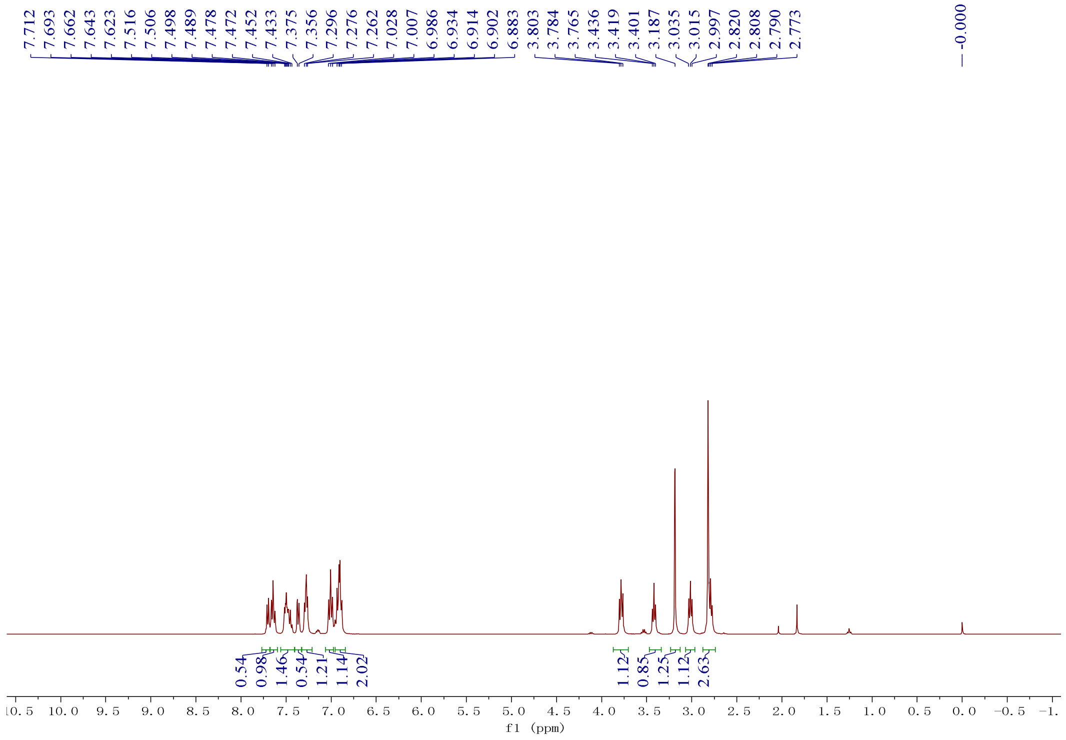

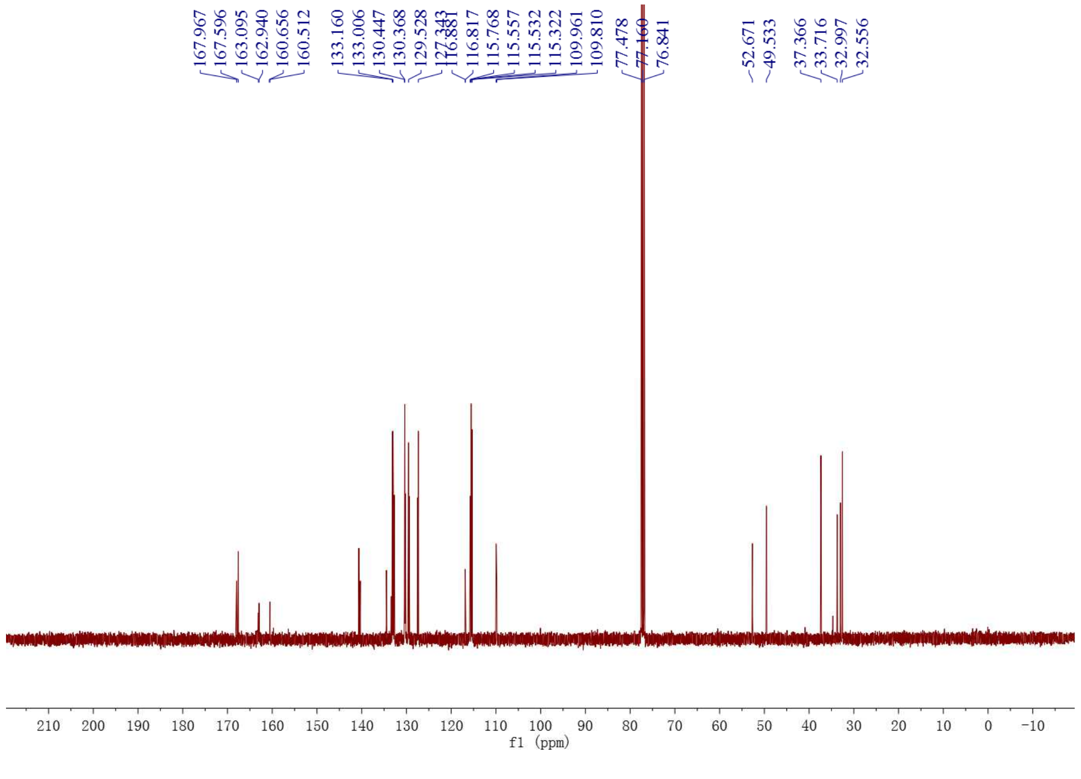

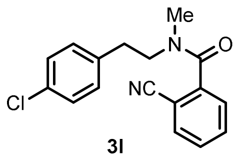
Me
N
O
NC
Cl

3l

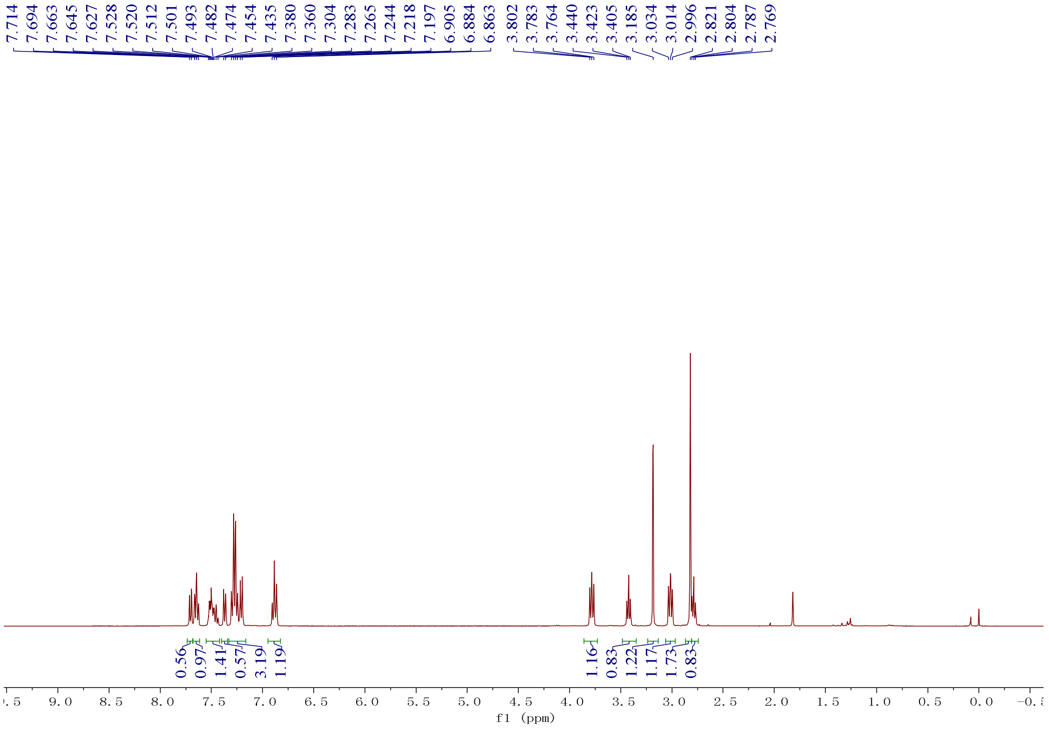
7.714 7.694 7.663 7.645 7.627 7.528 7.520 7.512 7.501 7.493 7.482 7.474 7.454 7.435 7.380 7.360 7.304 7.283 7.265 7.244 7.218 7.197 6.905 6.884 6.863 3.802 3.783 3.764 3.440 3.423 3.405 3.185 3.034 3.014 2.996 2.821 2.804 2.787 2.769
0.56 0.97 1.41 0.57 3.19 1.19 1.16 0.83 1.22 1.17 1.73 0.83
f1 (ppm)

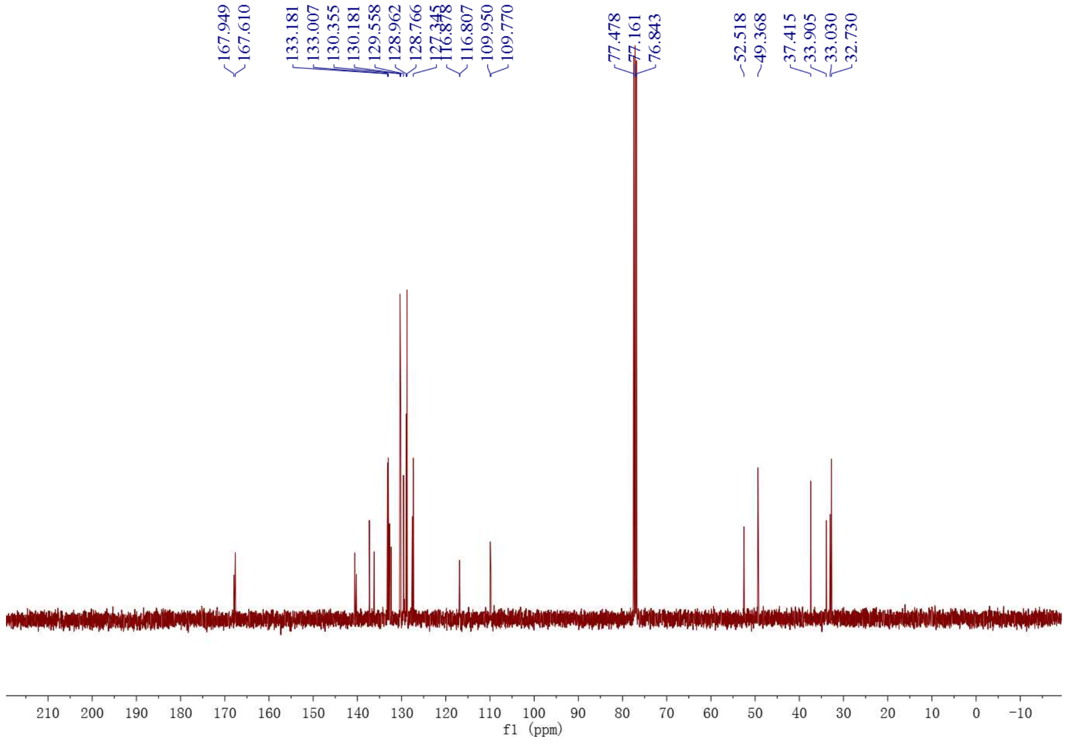
167.949 167.610 133.181 133.007 130.355 130.181 129.558 128.962 128.766 127.345 127.178 116.807 109.950 109.770 77.478 77.161 76.843 52.518 49.368 37.415 33.905 33.030 32.730
f1 (ppm)

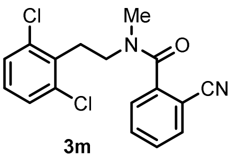
Cl
Me
N
O
CN
Cl

3m

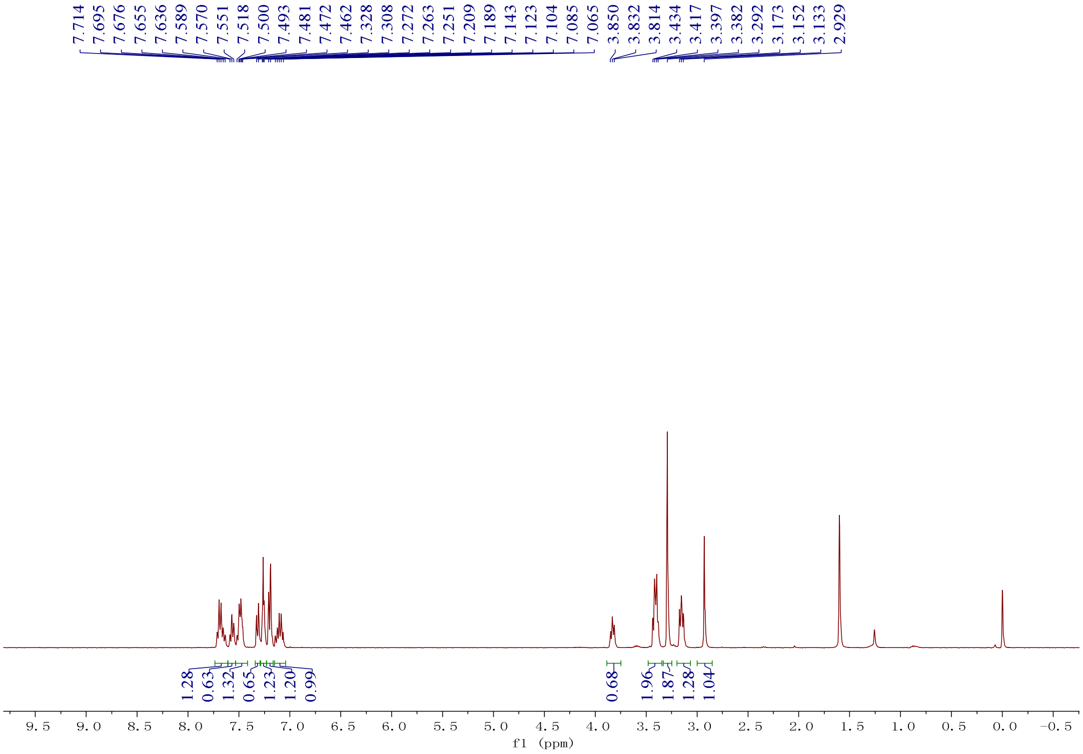
7.714 7.695 7.676 7.655 7.636 7.589 7.570 7.551 7.518 7.500 7.493 7.481 7.472 7.462 7.328 7.308 7.272 7.263 7.251 7.209 7.189 7.143 7.123 7.104 7.085 7.065 3.850 3.832 3.814 3.434 3.417 3.397 3.382 3.292 3.173 3.152 3.133 2.929
1.28 0.63 1.32 0.65 1.23 1.20 0.99 0.68 1.96 1.87 1.28 1.04
9.5 9.0 8.5 8.0 7.5 7.0 6.5 6.0 5.5 5.0 4.5 4.0 3.5 3.0 2.5 2.0 1.5 1.0 0.5 0.0 -0.5
f1 (ppm)

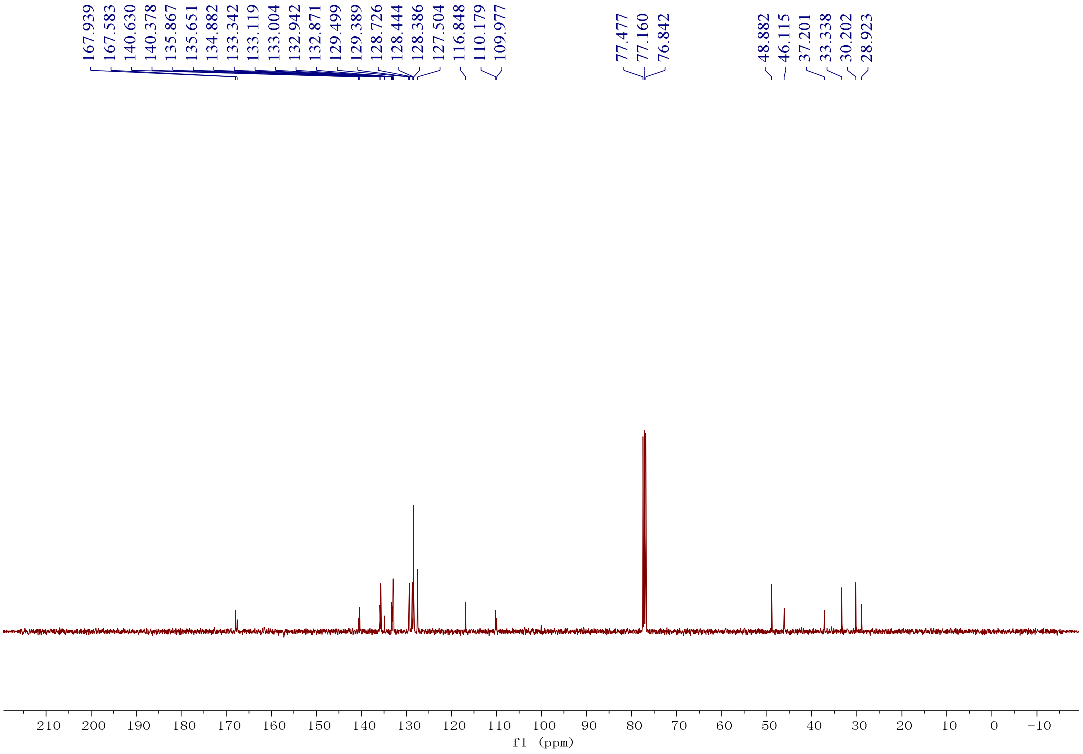
167.939 167.583 140.630 140.378 135.867 135.651 134.882 133.342 133.119 133.004 132.942 132.871 129.499 129.389 128.726 128.444 128.386 127.504 116.848 110.179 109.977
77.477 77.160 76.842
48.882 46.115 37.201 33.338 30.202 28.923
210 200 190 180 170 160 150 140 130 120 110 100 90 80 70 60 50 40 30 20 10 0 -10
f1 (ppm)

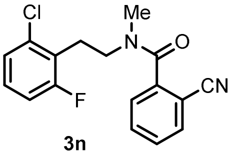
Cl
Me
N
O
CN
F

3n

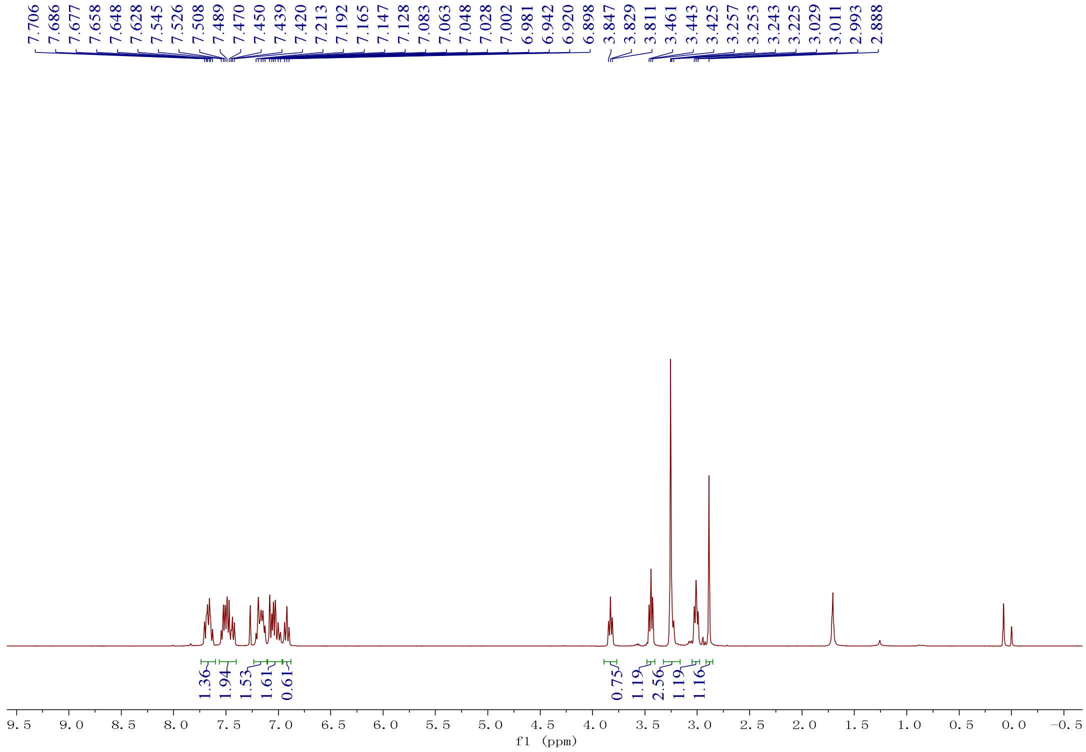
7.706
7.686
7.677
7.658
7.648
7.628
7.545
7.526
7.508
7.489
7.470
7.450
7.439
7.420
7.213
7.192
7.165
7.147
7.128
7.083
7.063
7.048
7.028
7.002
6.981
6.942
6.920
6.898
3.847
3.829
3.811
3.461
3.443
3.425
3.257
3.253
3.243
3.225
3.029
3.011
2.993
2.888
1.36
1.94
1.53
1.61
0.61
0.75
1.19
2.56
1.19
1.16
9.5 9.0 8.5 8.0 7.5 7.0 6.5 6.0 5.5 5.0 4.5 4.0 3.5 3.0 2.5 2.0 1.5 1.0 0.5 0.0 -0.5
f1 (ppm)

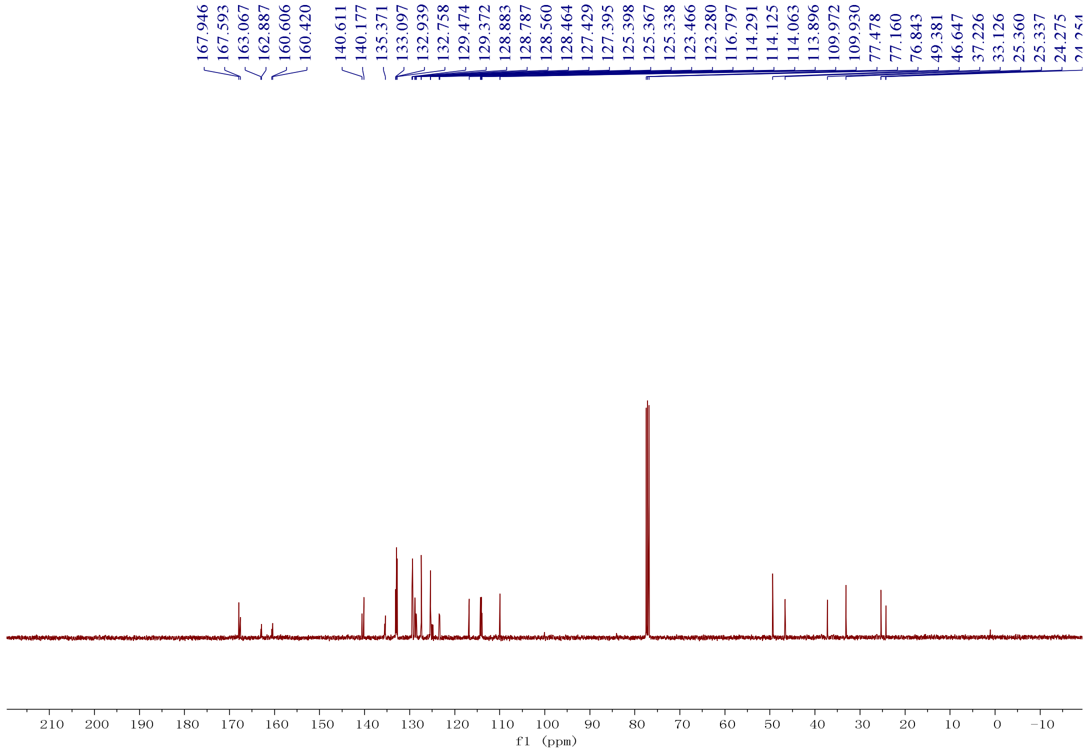
167.946
167.593
163.067
162.887
160.606
160.420
140.611
140.177
135.371
133.097
132.939
132.758
129.474
129.372
128.883
128.787
128.560
128.464
127.429
127.395
125.398
125.367
125.338
123.466
123.280
116.797
114.291
114.125
114.063
113.896
109.972
109.930
77.478
77.160
76.843
49.381
46.647
37.226
33.126
25.360
25.337
24.275
210 200 190 180 170 160 150 140 130 120 110 100 90 80 70 60 50 40 30 20 10 0 -10
f1 (ppm)

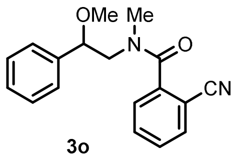
OMe
Me
N
O
CN

3o

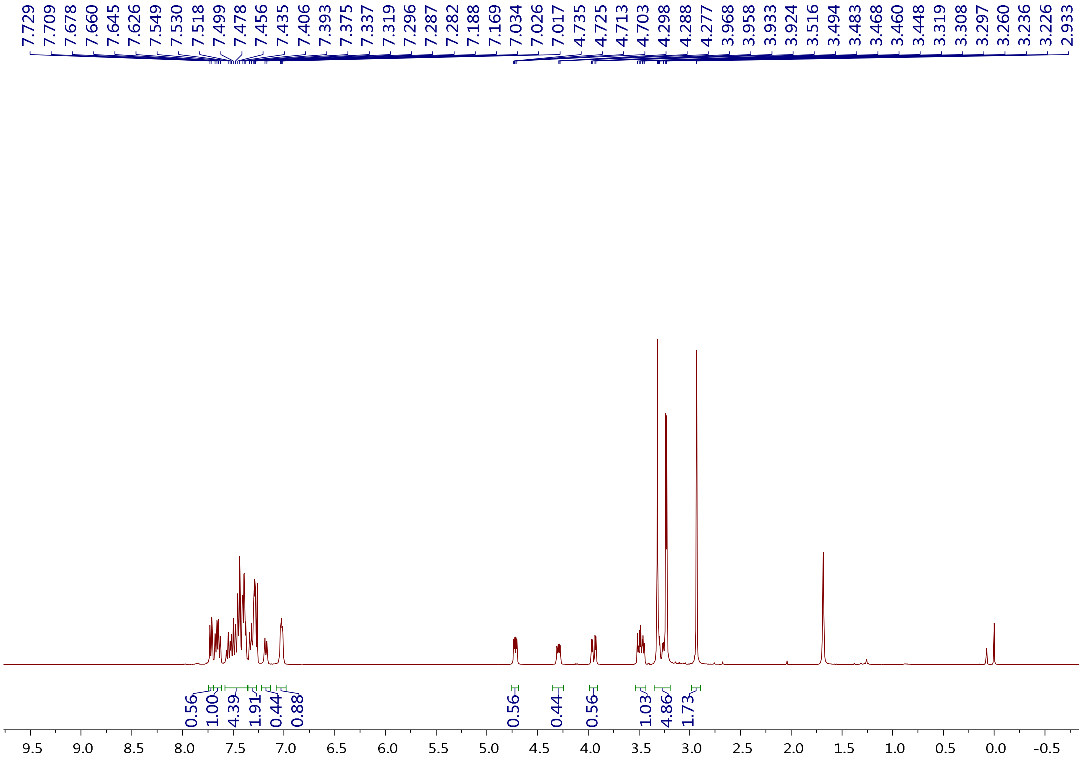
7.729
7.709
7.678
7.660
7.645
7.626
7.549
7.530
7.518
7.499
7.478
7.456
7.435
7.406
7.393
7.375
7.337
7.319
7.296
7.287
7.282
7.188
7.169
7.034
7.026
7.017
4.735
4.725
4.713
4.703
4.298
4.288
4.277
3.968
3.958
3.933
3.924
3.516
3.494
3.483
3.468
3.460
3.448
3.319
3.308
3.297
3.260
3.236
3.226
2.933
0.56
1.00
4.39
1.91
0.44
0.88
0.56
0.44
0.56
1.03
4.86
1.73
9.5
9.0
8.5
8.0
7.5
7.0
6.5
6.0
5.5
5.0
4.5
4.0
3.5
3.0
2.5
2.0
1.5
1.0
0.5
0.0
-0.5

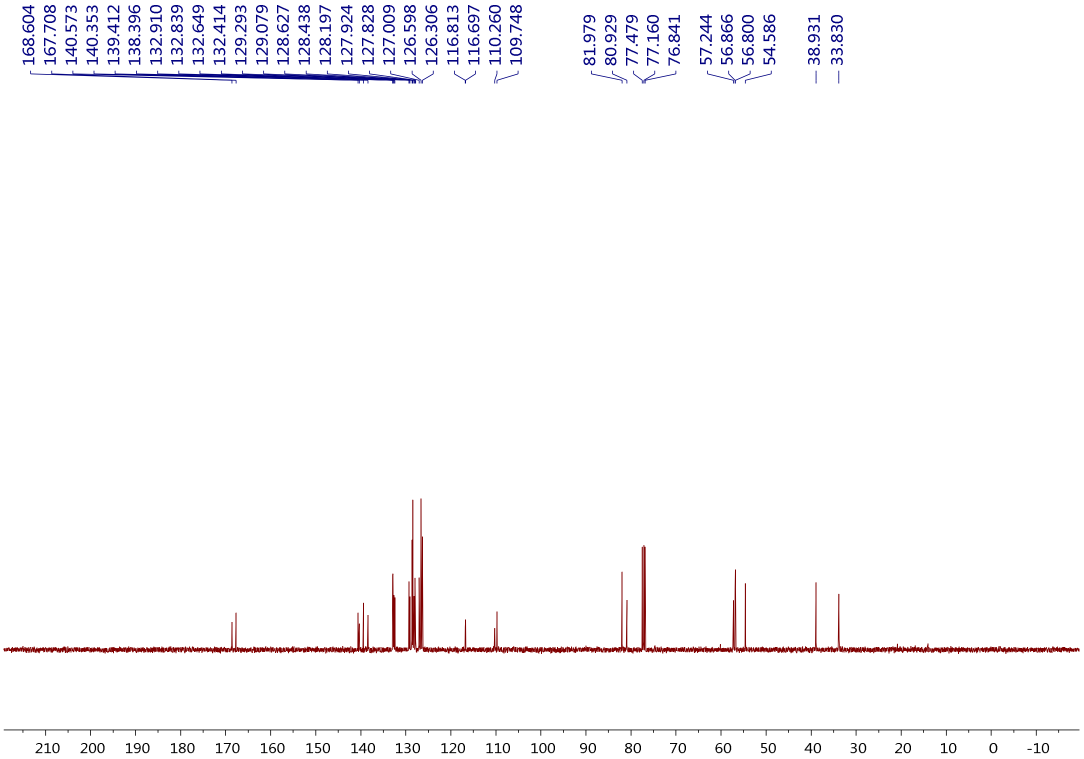
168.604
167.708
140.573
140.353
139.412
138.396
132.910
132.839
132.649
132.414
129.293
129.079
128.627
128.438
128.197
127.924
127.828
127.009
126.598
126.306
116.813
116.697
110.260
109.748
81.979
80.929
77.479
77.160
76.841
57.244
56.866
56.800
54.586
38.931
33.830
210
200
190
180
170
160
150
140
130
120
110
100
90
80
70
60
50
40
30
20
10
0
-10

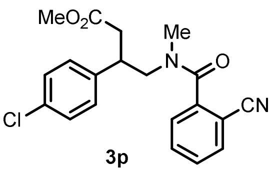
MeO2C
Me
N
O
CN
Cl

3p

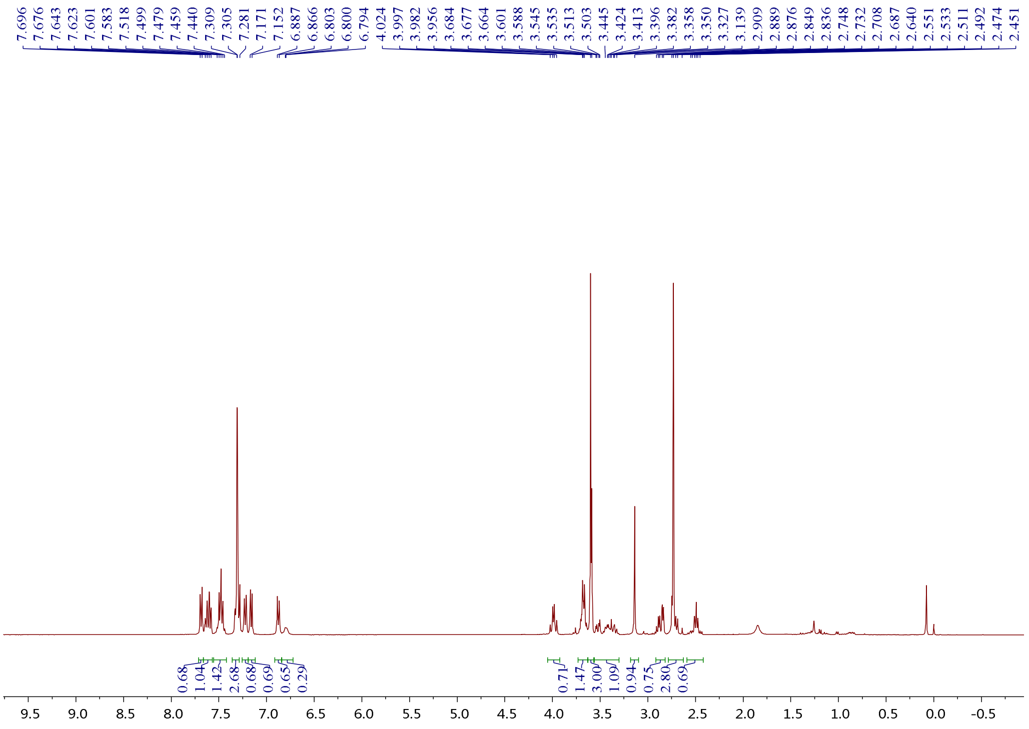
7.696
7.676
7.643
7.623
7.601
7.583
7.518
7.499
7.479
7.459
7.440
7.309
7.305
7.281
7.171
7.152
6.887
6.866
6.803
6.800
6.794
4.024
3.997
3.982
3.956
3.684
3.677
3.664
3.601
3.588
3.545
3.535
3.513
3.503
3.445
3.424
3.413
3.396
3.382
3.358
3.350
3.327
3.139
2.909
2.889
2.876
2.849
2.836
2.748
2.732
2.708
2.687
2.640
2.551
2.533
2.511
2.492
2.474
2.451
0.68
1.04
1.42
2.68
0.68
0.69
0.65
0.29
0.71
1.47
3.00
1.09
0.94
0.75
2.80
0.69
9.5 9.0 8.5 8.0 7.5 7.0 6.5 6.0 5.5 5.0 4.5 4.0 3.5 3.0 2.5 2.0 1.5 1.0 0.5 0.0 -0.5

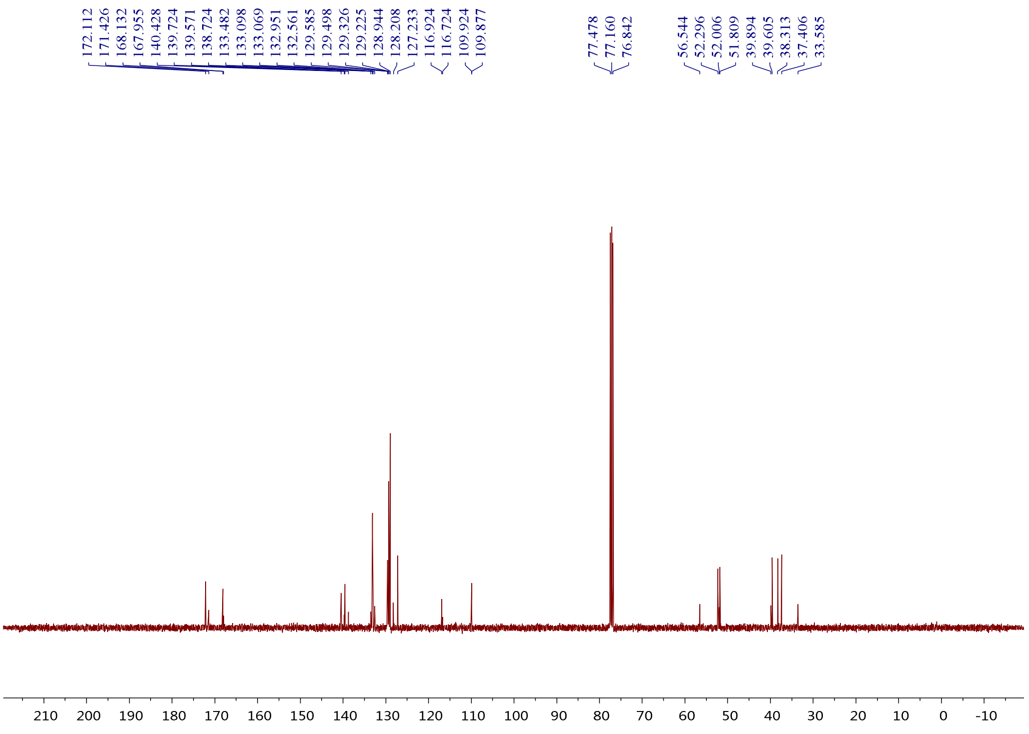
172.112
171.426
168.132
167.955
140.428
139.724
139.571
138.724
133.482
133.098
133.069
132.951
132.561
129.585
129.498
129.326
129.225
128.944
128.208
127.233
116.924
116.724
109.924
109.877
77.478
77.160
76.842
56.544
52.296
52.006
51.809
39.894
39.605
38.313
37.406
33.585
210 200 190 180 170 160 150 140 130 120 110 100 90 80 70 60 50 40 30 20 10 0 -10

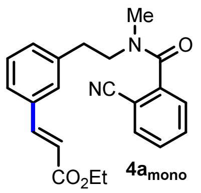
Me
N
O
NC
CO2Et

4a$_{mono}$

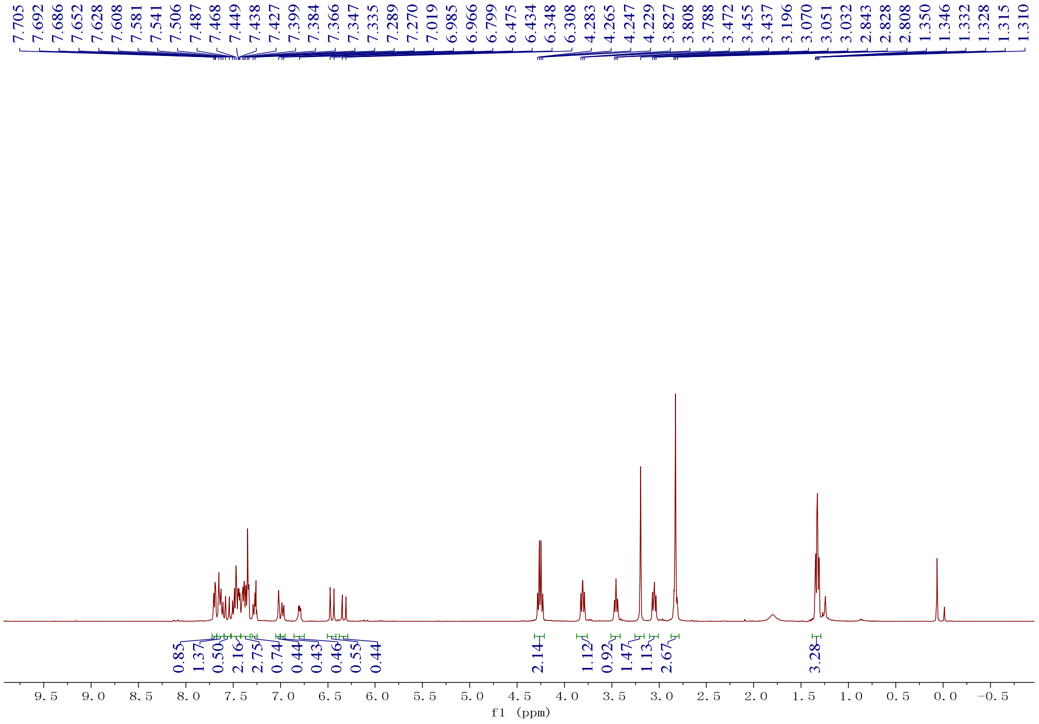
7.705
7.692
7.686
7.652
7.628
7.608
7.581
7.541
7.506
7.487
7.468
7.449
7.438
7.427
7.399
7.384
7.366
7.347
7.335
7.289
7.270
7.019
6.985
6.966
6.799
6.475
6.434
6.348
6.308
4.283
4.265
4.247
4.229
3.827
3.808
3.788
3.472
3.455
3.437
3.196
3.070
3.051
3.032
2.843
2.828
2.808
1.350
1.346
1.332
1.328
1.315
1.310
0.85
1.37
0.50
2.16
2.75
0.74
0.44
0.43
0.46
0.55
0.44
2.14
1.12
0.92
1.47
1.13
2.67
3.28
9.5
9.0
8.5
8.0
7.5
7.0
6.5
6.0
5.5
5.0
4.5
4.0
3.5
3.0
2.5
2.0
1.5
1.0
0.5
0.0
-0.5
f1 (ppm)

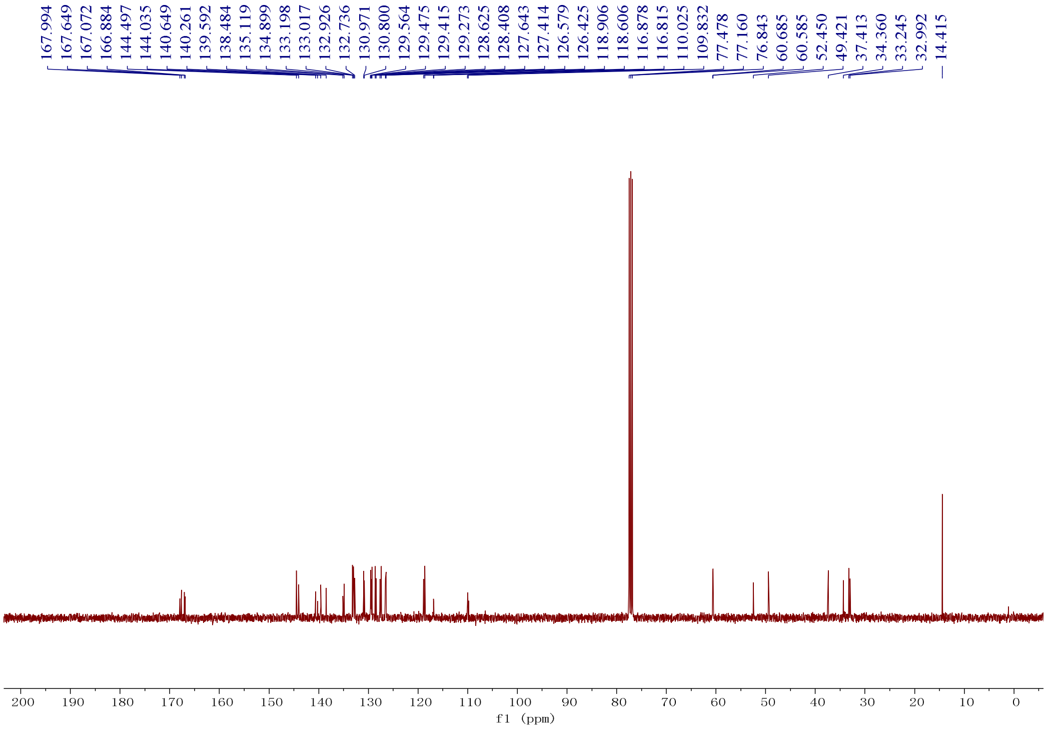
167.994
167.649
167.072
166.884
144.497
144.035
140.649
140.261
139.592
138.484
135.119
134.899
133.198
133.017
132.926
132.736
130.971
130.800
129.564
129.475
129.415
129.273
128.625
128.408
127.643
127.414
126.579
126.425
118.906
118.606
116.878
116.815
110.025
109.832
77.478
77.160
76.843
60.685
60.585
52.450
49.421
37.413
34.360
33.245
32.992
14.415
200
190
180
170
160
150
140
130
120
110
100
90
80
70
60
50
40
30
20
10
0
f1 (ppm)

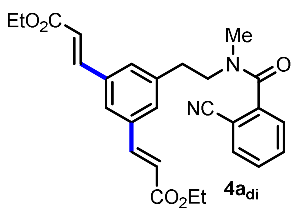
EtO2C
Me
N
O
NC
CO2Et

4a$_{di}$

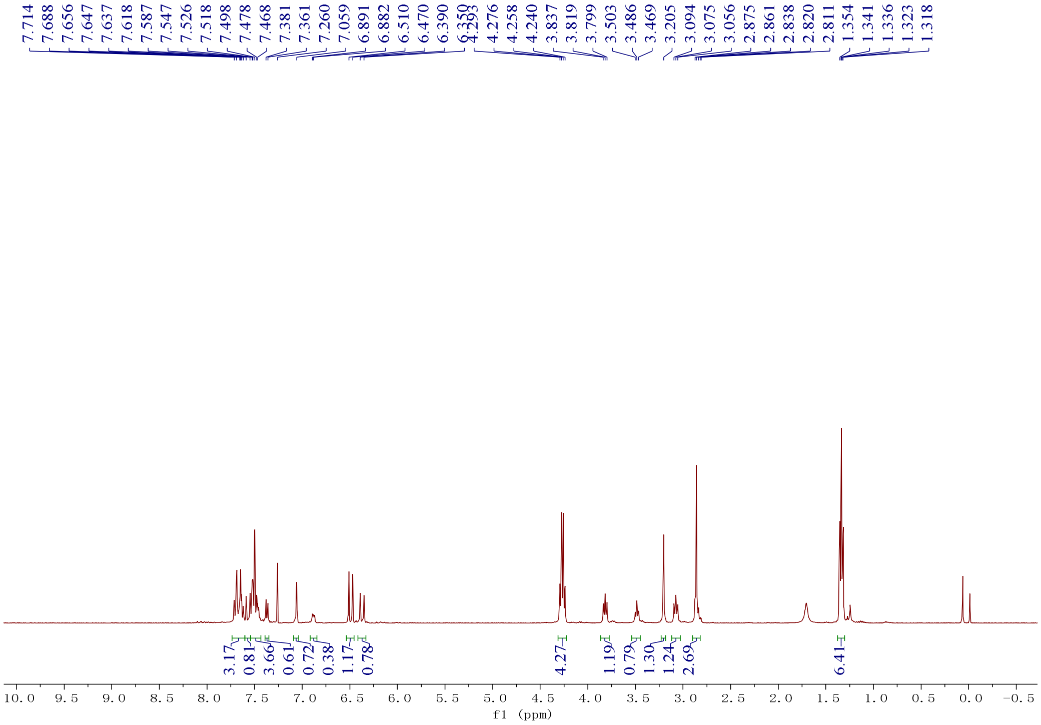
7.714
7.688
7.656
7.647
7.637
7.618
7.587
7.547
7.526
7.518
7.498
7.478
7.468
7.381
7.361
7.260
7.059
6.891
6.882
6.510
6.470
6.390
6.350
4.293
4.276
4.258
4.240
3.837
3.819
3.799
3.503
3.486
3.469
3.205
3.094
3.075
3.056
2.875
2.861
2.838
2.820
2.811
1.354
1.341
1.336
1.323
1.318
3.17
0.81
3.66
0.61
0.72
0.38
1.17
0.78
4.27
1.19
0.79
1.30
1.24
2.69
6.41
10.0 9.5 9.0 8.5 8.0 7.5 7.0 6.5 6.0 5.5 5.0 4.5 4.0 3.5 3.0 2.5 2.0 1.5 1.0 0.5 0.0 -0.5
f1 (ppm)

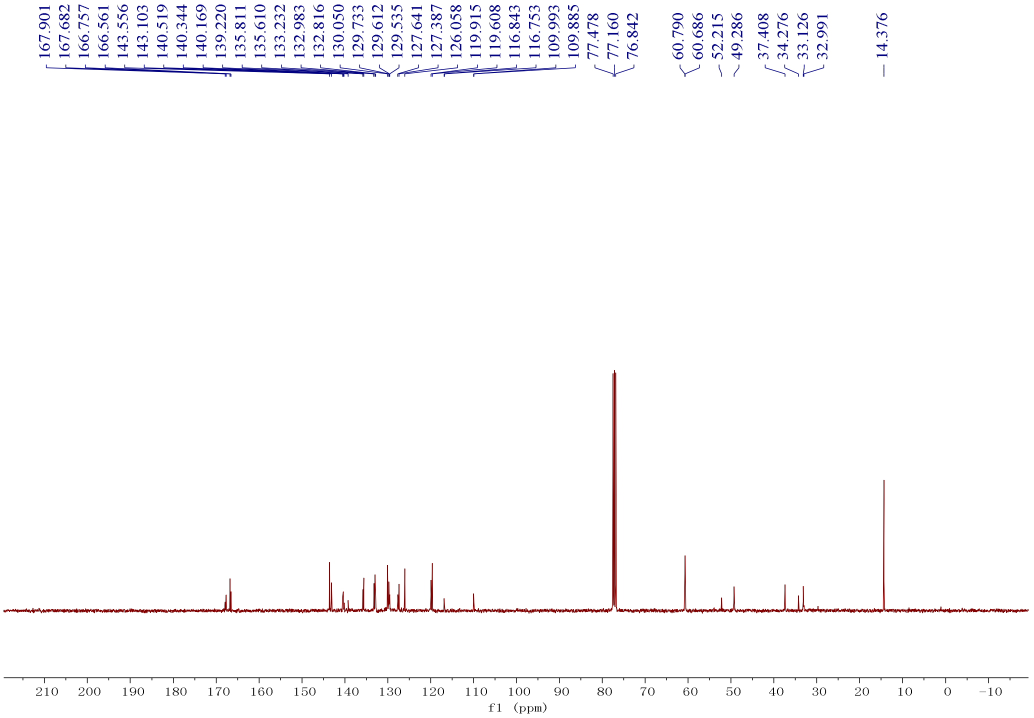
167.901
167.682
166.757
166.561
143.556
143.103
140.519
140.344
140.169
139.220
135.811
135.610
133.232
132.983
132.816
130.050
129.733
129.612
129.535
127.641
127.387
126.058
119.915
119.608
116.843
116.753
109.993
109.885
77.478
77.160
76.842
60.790
60.686
52.215
49.286
37.408
34.276
33.126
32.991
14.376
210 200 190 180 170 160 150 140 130 120 110 100 90 80 70 60 50 40 30 20 10 0 -10
f1 (ppm)

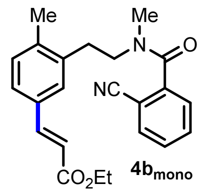

Me
Me
N
O
NC
CO2Et

$4b_{mono}$

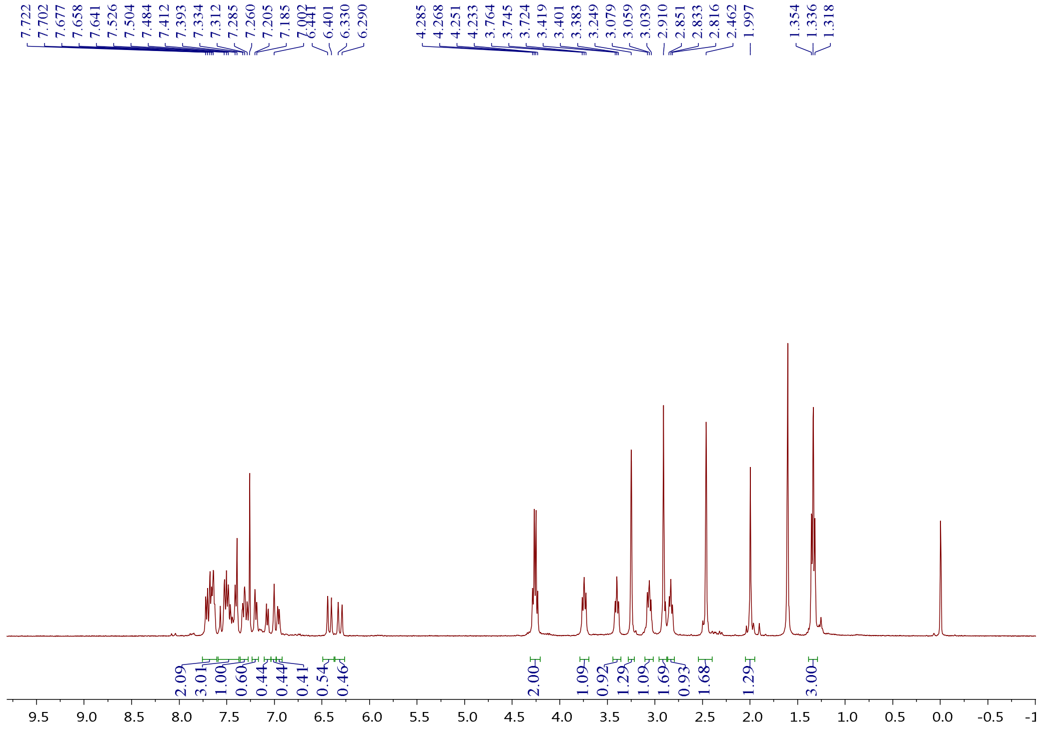

7.722
7.702
7.677
7.658
7.641
7.526
7.504
7.484
7.412
7.393
7.334
7.312
7.285
7.260
7.205
7.185
7.002
6.441
6.401
6.330
6.290
4.285
4.268
4.251
4.233
3.764
3.745
3.724
3.419
3.401
3.383
3.249
3.079
3.059
3.039
2.910
2.851
2.833
2.816
2.462
1.997
1.354
1.336
1.318
2.09
3.01
1.00
0.60
0.44
0.44
0.41
0.54
0.46
2.00
1.09
0.92
1.29
1.09
1.69
0.93
1.68
1.29
3.00
9.5 9.0 8.5 8.0 7.5 7.0 6.5 6.0 5.5 5.0 4.5 4.0 3.5 3.0 2.5 2.0 1.5 1.0 0.5 0.0 -0.5 -1

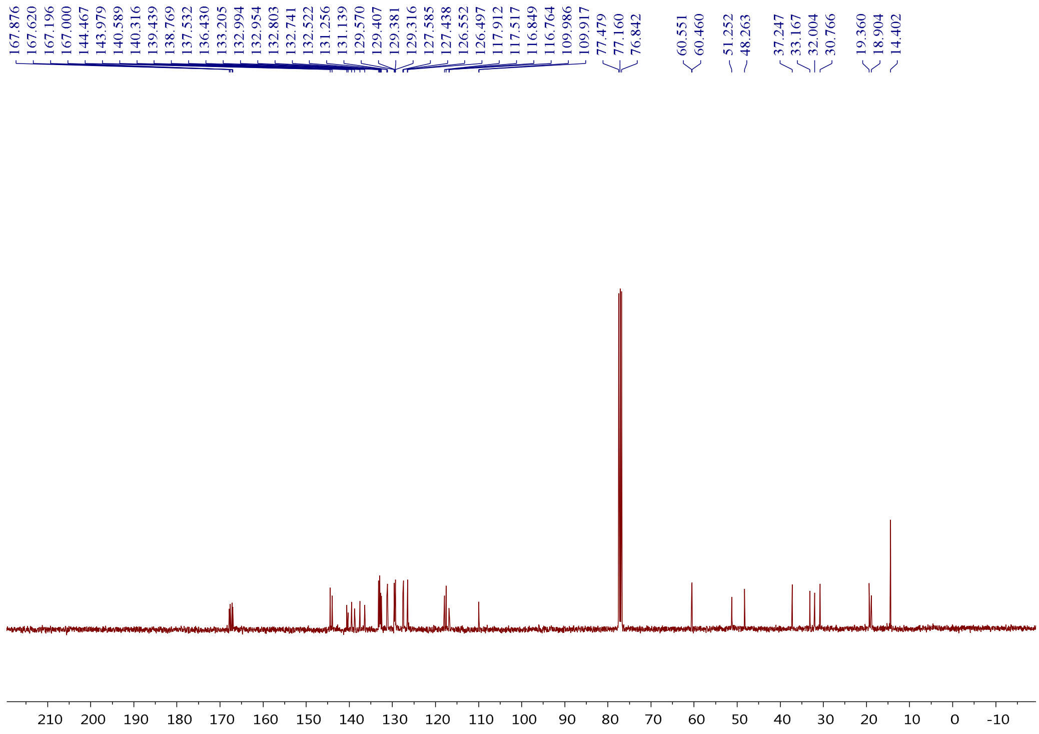

167.876
167.620
167.196
167.000
144.467
143.979
140.589
140.316
139.439
138.769
137.532
136.430
133.205
132.994
132.954
132.803
132.741
132.522
131.256
131.139
129.570
129.407
129.381
129.316
127.585
127.438
126.552
126.497
117.912
117.517
116.849
116.764
109.986
109.917
77.479
77.160
76.842
60.551
60.460
51.252
48.263
37.247
33.167
32.004
30.766
19.360
18.904
14.402
210 200 190 180 170 160 150 140 130 120 110 100 90 80 70 60 50 40 30 20 10 0 -10

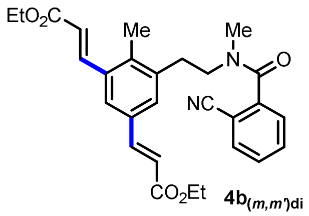
EtO2C
Me
Me
N
O
NC
CO2Et

4b$_{(m,m')di}$

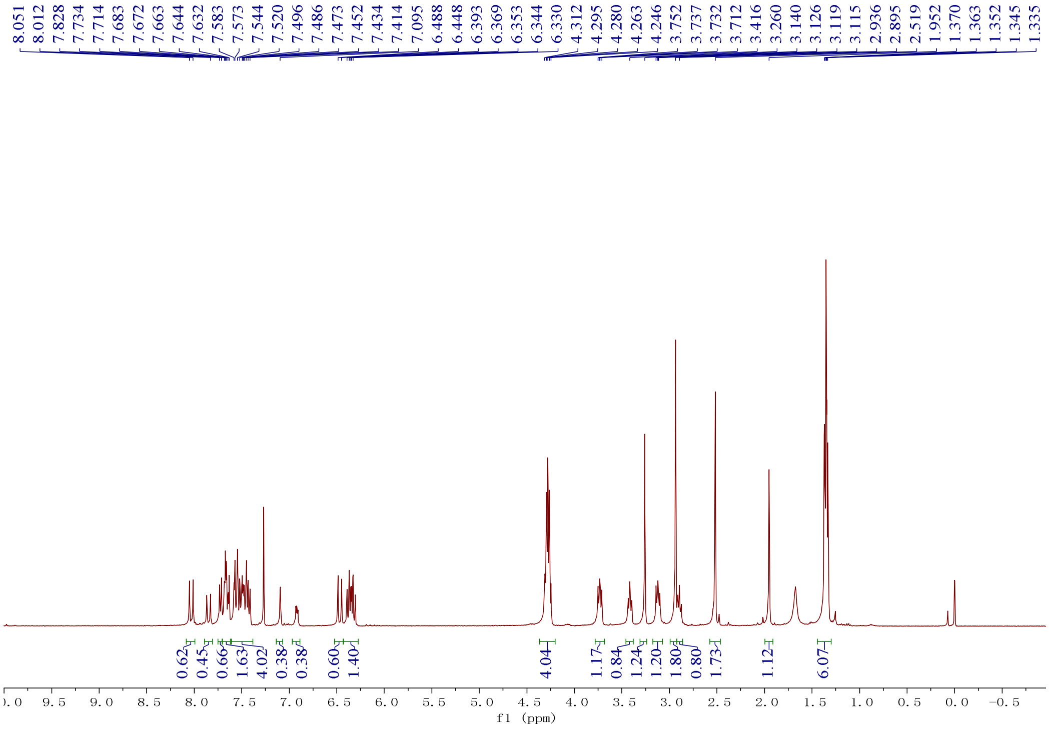
8.051
8.012
7.828
7.734
7.714
7.683
7.672
7.663
7.644
7.632
7.583
7.573
7.544
7.520
7.496
7.486
7.473
7.452
7.434
7.414
7.095
6.488
6.448
6.393
6.369
6.353
6.344
6.330
4.312
4.295
4.280
4.263
4.246
3.752
3.737
3.732
3.712
3.416
3.260
3.140
3.126
3.119
3.115
2.936
2.895
2.519
1.952
1.370
1.363
1.352
1.345
1.335
0.62
0.45
0.66
1.63
4.02
0.38
0.38
0.60
1.40
4.04
1.17
0.84
1.24
1.20
1.80
0.80
1.73
1.12
6.07
f1 (ppm)

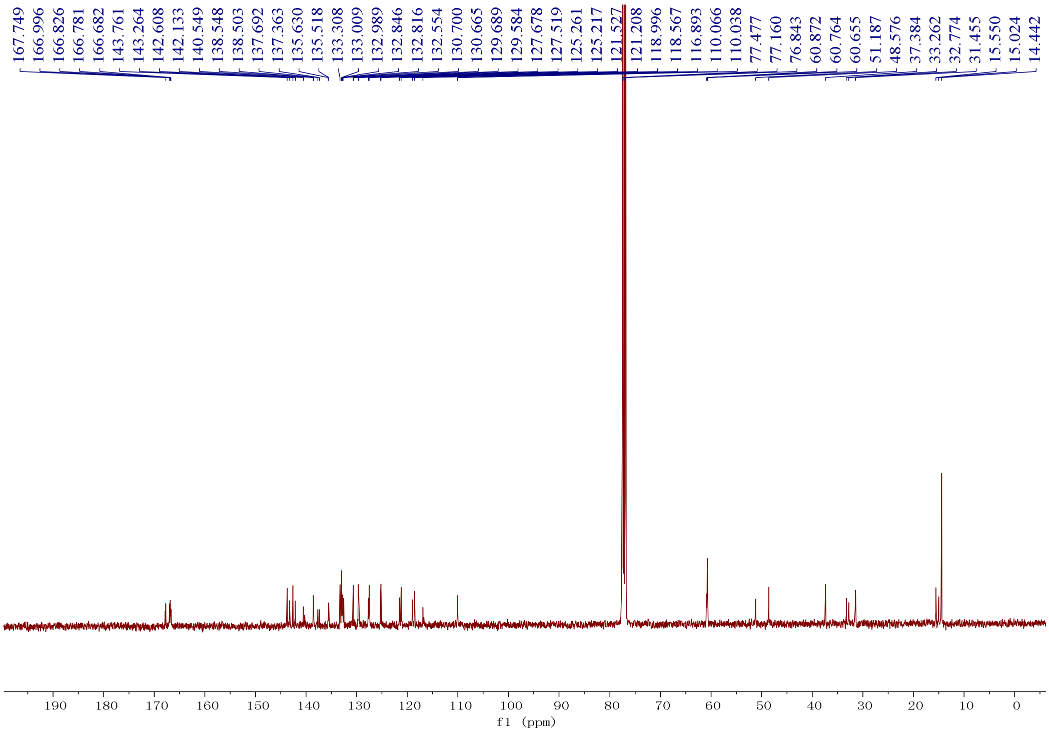
167.749
166.996
166.826
166.781
166.682
143.761
143.264
142.608
142.133
140.549
138.548
138.503
137.692
137.363
135.630
135.518
133.308
133.009
132.989
132.846
132.816
132.554
130.700
130.665
129.689
129.584
127.678
127.519
125.261
125.217
121.527
121.208
118.996
118.567
116.893
110.066
110.038
77.477
77.160
76.843
60.872
60.764
60.655
51.187
48.576
37.384
33.262
32.774
31.455
15.550
15.024
14.442
f1 (ppm)

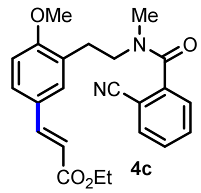

OMe
Me
N
O
NC
CO2Et

4c

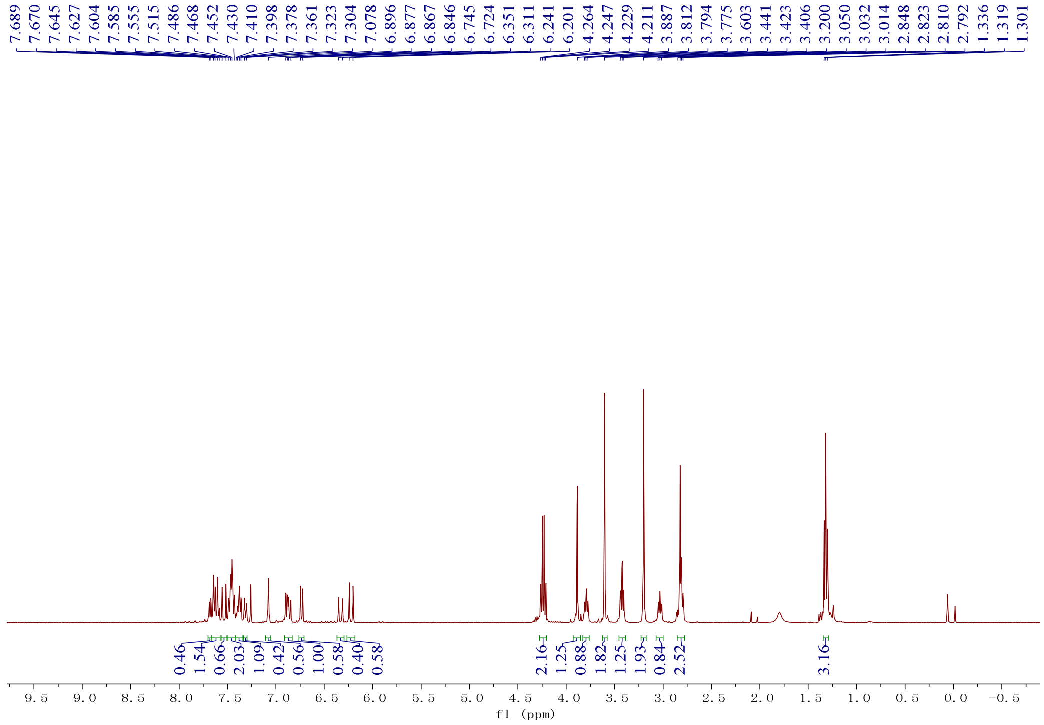

7.689
7.670
7.645
7.627
7.604
7.585
7.555
7.515
7.486
7.468
7.452
7.430
7.410
7.398
7.378
7.361
7.323
7.304
7.078
6.896
6.877
6.867
6.846
6.745
6.724
6.351
6.311
6.241
6.201
4.264
4.247
4.229
4.211
3.887
3.812
3.794
3.775
3.603
3.441
3.423
3.406
3.200
3.050
3.032
3.014
2.848
2.823
2.810
2.792
1.336
1.319
1.301
0.46
1.54
0.66
2.03
1.09
0.42
0.56
1.00
0.58
0.40
0.58
2.16
1.25
0.88
1.82
1.25
1.93
0.84
2.52
3.16
9.5 9.0 8.5 8.0 7.5 7.0 6.5 6.0 5.5 5.0 4.5 4.0 3.5 3.0 2.5 2.0 1.5 1.0 0.5 0.0 -0.5
f1 (ppm)

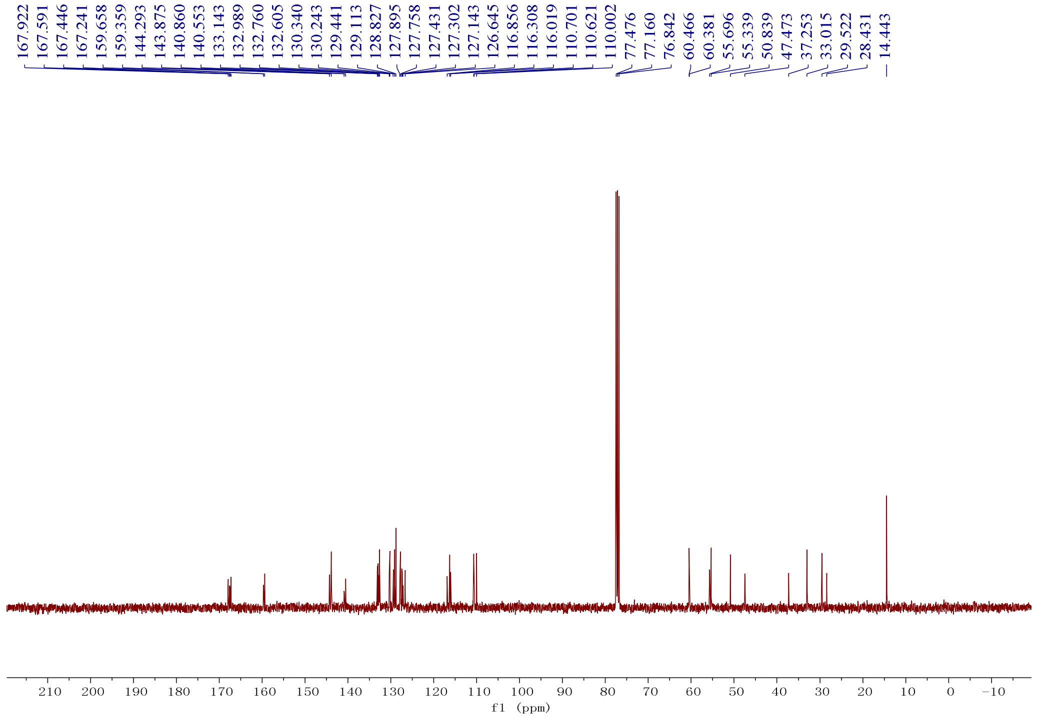

167.922
167.591
167.446
167.241
159.658
159.359
144.293
143.875
140.860
140.553
133.143
132.989
132.760
132.605
130.340
130.243
129.441
129.113
128.827
127.895
127.758
127.431
127.302
127.143
126.645
116.856
116.308
116.019
110.701
110.621
110.002
77.476
77.160
76.842
60.466
60.381
55.696
55.339
50.839
47.473
37.253
33.015
29.522
28.431
14.443
210 200 190 180 170 160 150 140 130 120 110 100 90 80 70 60 50 40 30 20 10 0 -10
f1 (ppm)

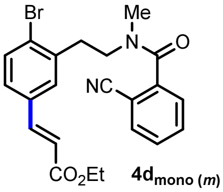

4d$_{\mathbf{mono}\ (m)}$

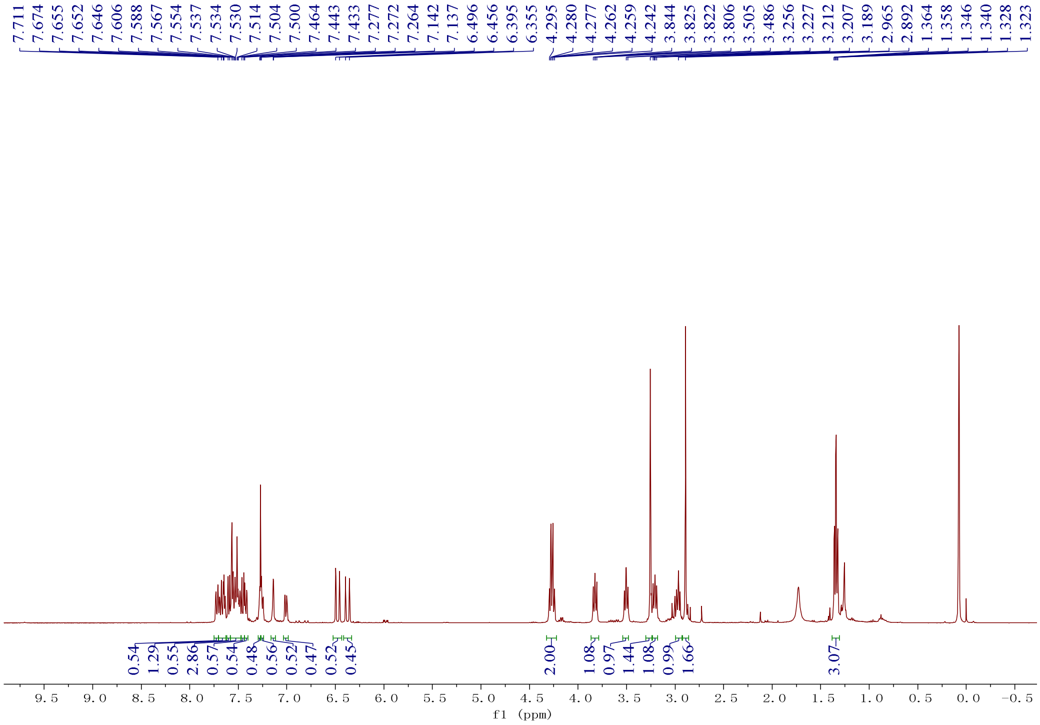

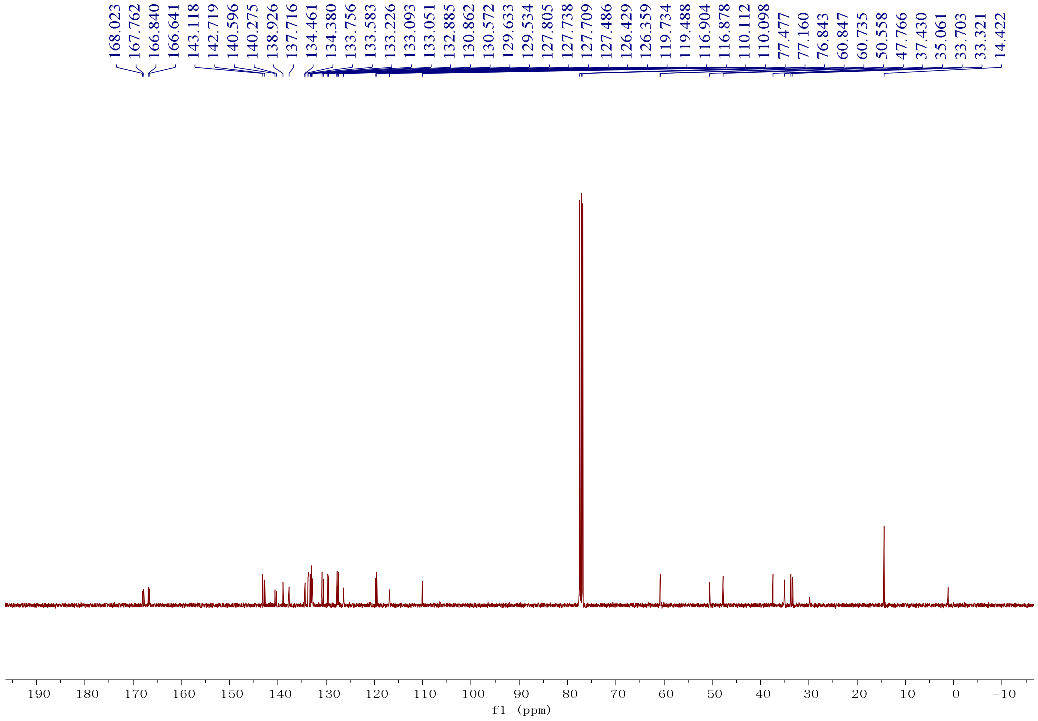

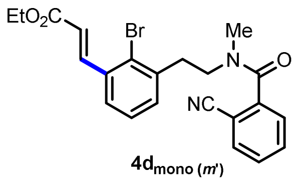

EtO2C
Br
Me
N
O
NC

4d$_{mono\ (m')}$

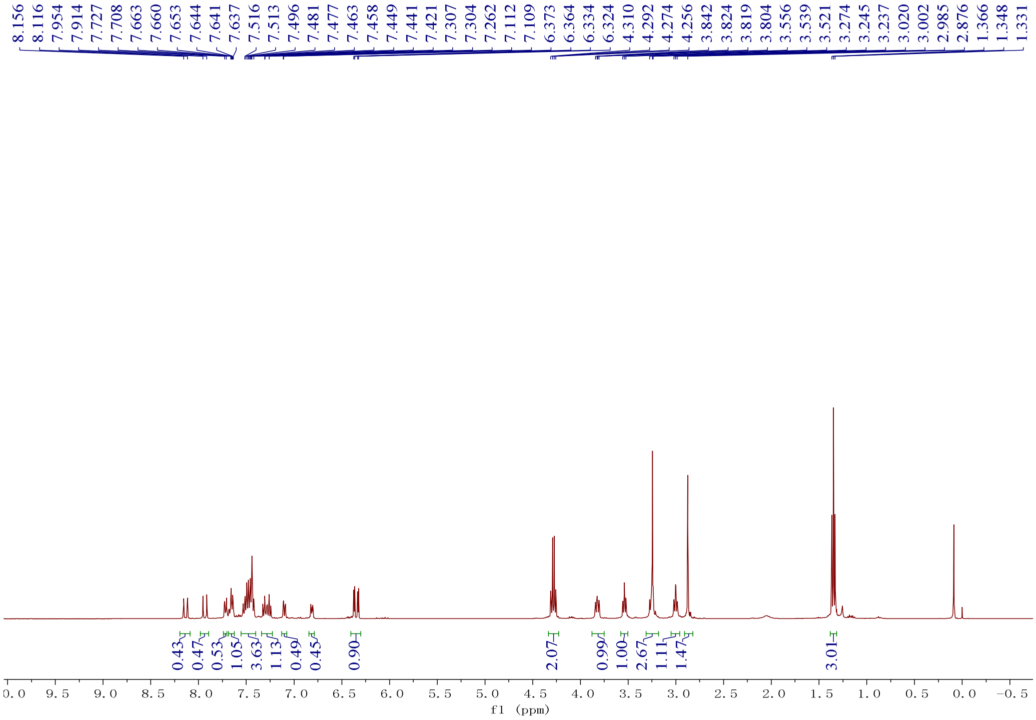

8.156
8.116
7.954
7.914
7.727
7.708
7.663
7.660
7.653
7.644
7.641
7.637
7.516
7.513
7.496
7.481
7.477
7.463
7.458
7.449
7.441
7.421
7.307
7.304
7.262
7.112
7.109
6.373
6.364
6.334
6.324
4.310
4.292
4.274
4.256
3.842
3.824
3.819
3.804
3.556
3.539
3.521
3.274
3.245
3.237
3.020
3.002
2.985
2.876
1.366
1.348
1.331
0.43
0.47
0.53
1.05
3.63
1.13
0.49
0.45
0.90
2.07
0.99
1.00
2.67
1.11
1.47
3.01
0.0 9.5 9.0 8.5 8.0 7.5 7.0 6.5 6.0 5.5 5.0 4.5 4.0 3.5 3.0 2.5 2.0 1.5 1.0 0.5 0.0 -0.5
f1 (ppm)

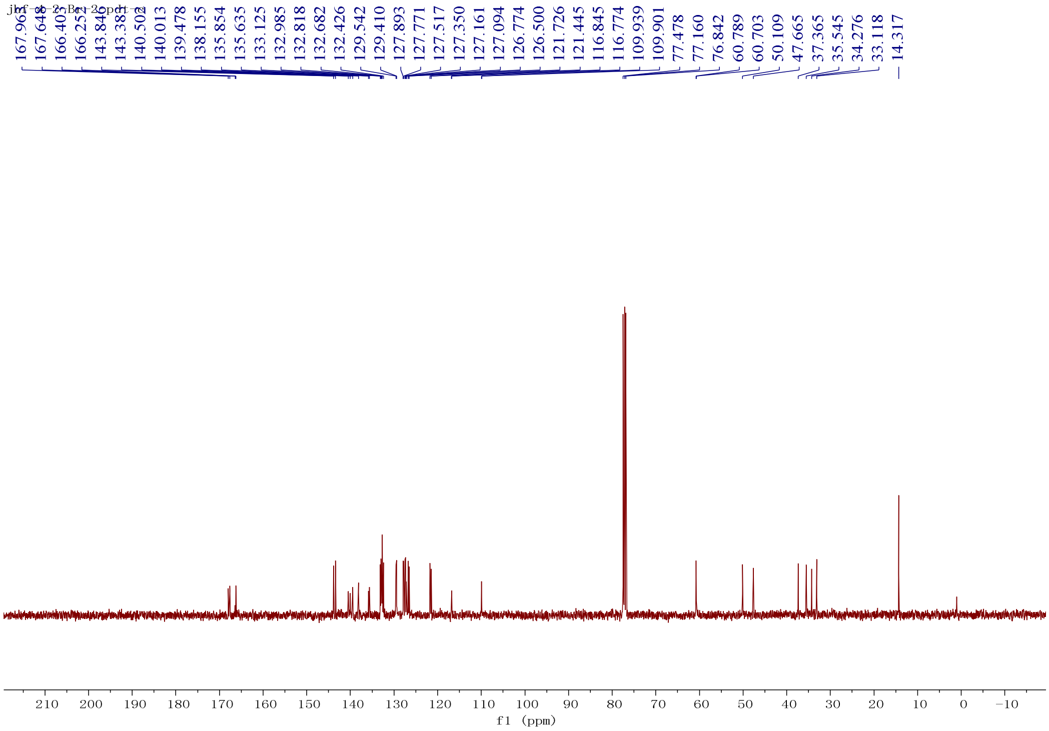

167.963
167.648
166.403
166.252
143.846
143.382
140.502
140.013
139.478
138.155
135.854
135.635
133.125
132.985
132.818
132.682
132.426
129.542
129.410
127.893
127.771
127.517
127.350
127.161
127.094
126.774
126.500
121.726
121.445
116.845
116.774
109.939
109.901
77.478
77.160
76.842
60.789
60.703
50.109
47.665
37.365
35.545
34.276
33.118
14.317
210 200 190 180 170 160 150 140 130 120 110 100 90 80 70 60 50 40 30 20 10 0 -10
f1 (ppm)

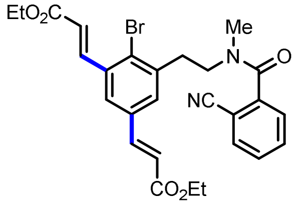
EtO2C
Br
Me
N
O
NC
CO2Et

4d$_{(m,m')di}$

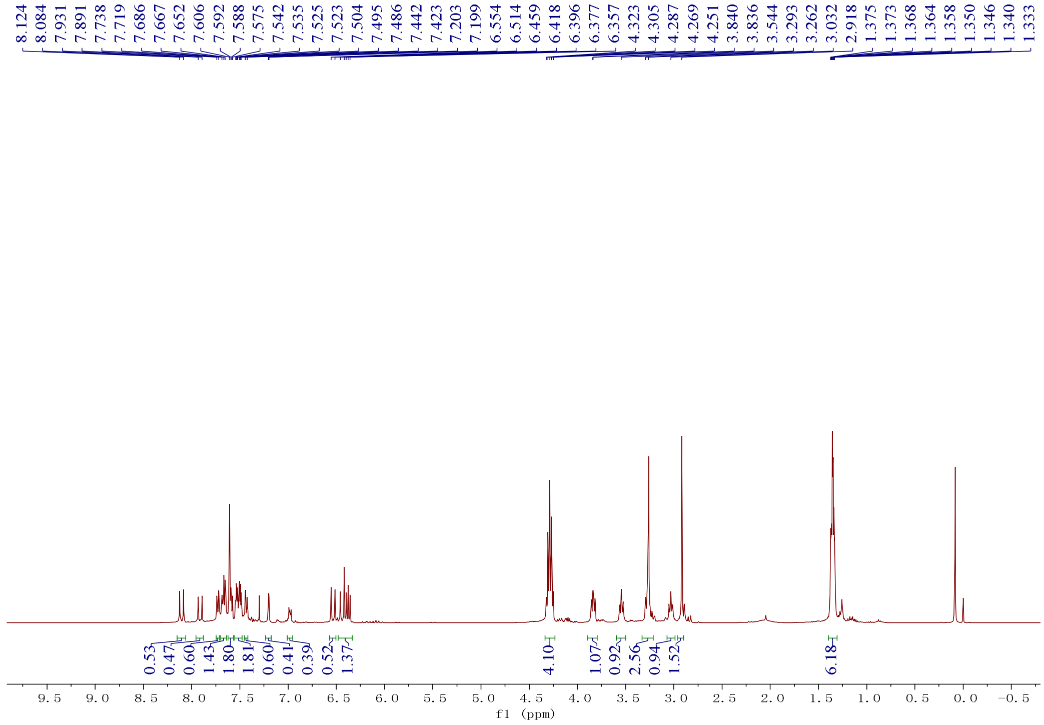
8.124
8.084
7.931
7.891
7.738
7.719
7.686
7.667
7.652
7.606
7.592
7.588
7.575
7.542
7.535
7.525
7.523
7.504
7.495
7.486
7.442
7.423
7.203
7.199
6.554
6.514
6.459
6.418
6.396
6.377
6.357
4.323
4.305
4.287
4.269
4.251
3.840
3.836
3.544
3.293
3.262
3.032
2.918
1.375
1.373
1.368
1.364
1.358
1.350
1.346
1.340
1.333
0.53
0.47
0.60
1.43
1.80
1.81
0.60
0.41
0.39
0.52
1.37
4.10
1.07
0.92
2.56
0.94
1.52
6.18
9.5 9.0 8.5 8.0 7.5 7.0 6.5 6.0 5.5 5.0 4.5 4.0 3.5 3.0 2.5 2.0 1.5 1.0 0.5 0.0 -0.5
f1 (ppm)

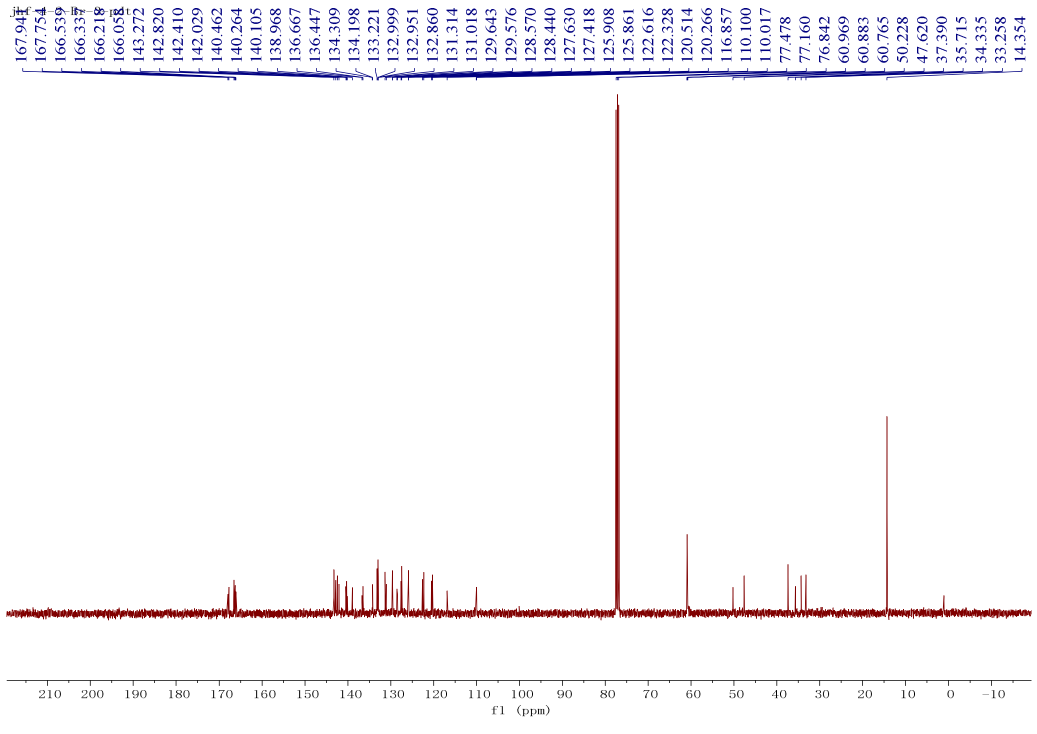
167.941
167.754
166.539
166.337
166.218
166.058
143.272
142.820
142.410
142.029
140.462
140.264
140.105
138.968
136.667
136.447
134.309
134.198
133.221
132.999
132.951
132.860
131.314
131.018
129.643
129.576
128.570
128.440
127.630
127.418
125.908
125.861
122.616
122.328
120.514
120.266
116.857
110.100
110.017
77.478
77.160
76.842
60.969
60.883
60.765
50.228
47.620
37.390
35.715
34.335
33.258
14.354
210 200 190 180 170 160 150 140 130 120 110 100 90 80 70 60 50 40 30 20 10 0 -10
f1 (ppm)

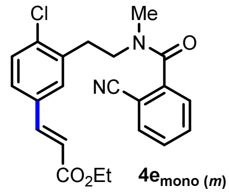
Cl
Me
N
O
NC
CO_2Et

4e$_{mono\ (m)}$

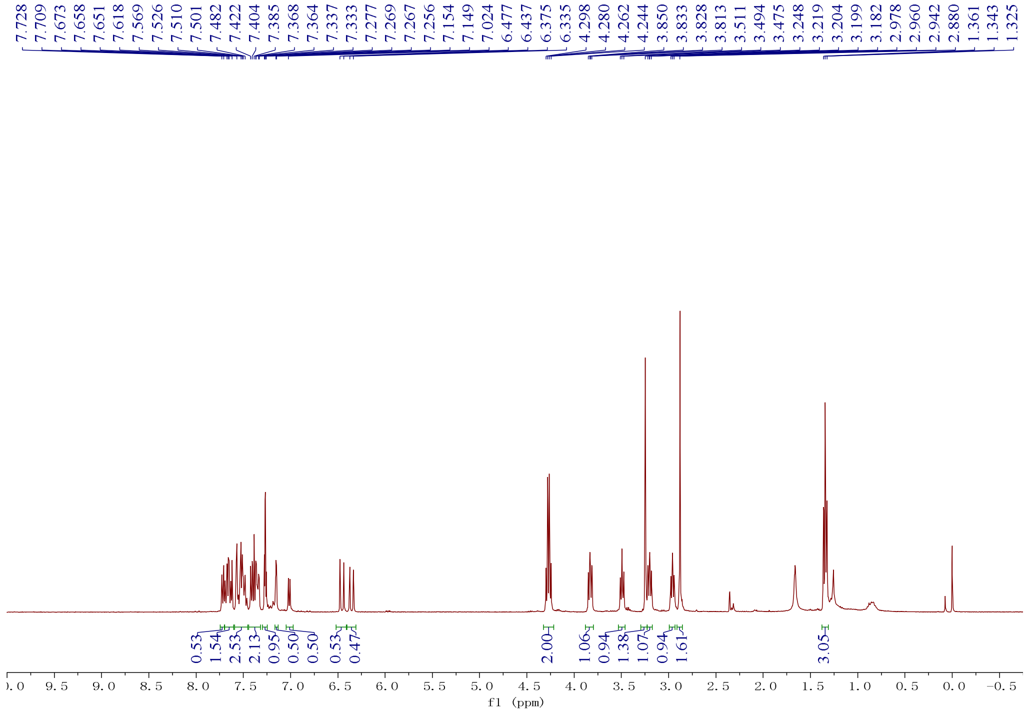
7.728
7.709
7.673
7.658
7.651
7.618
7.569
7.526
7.510
7.501
7.482
7.422
7.404
7.385
7.368
7.364
7.337
7.333
7.277
7.269
7.267
7.256
7.154
7.149
7.024
6.477
6.437
6.375
6.335
4.298
4.280
4.262
4.244
3.850
3.833
3.828
3.813
3.511
3.494
3.475
3.248
3.219
3.204
3.199
3.182
2.978
2.960
2.942
2.880
1.361
1.343
1.325
0.53
1.54
2.53
2.13
0.95
0.50
0.50
0.53
0.47
2.00
1.06
0.94
1.38
1.07
0.94
1.61
3.05
f1 (ppm)

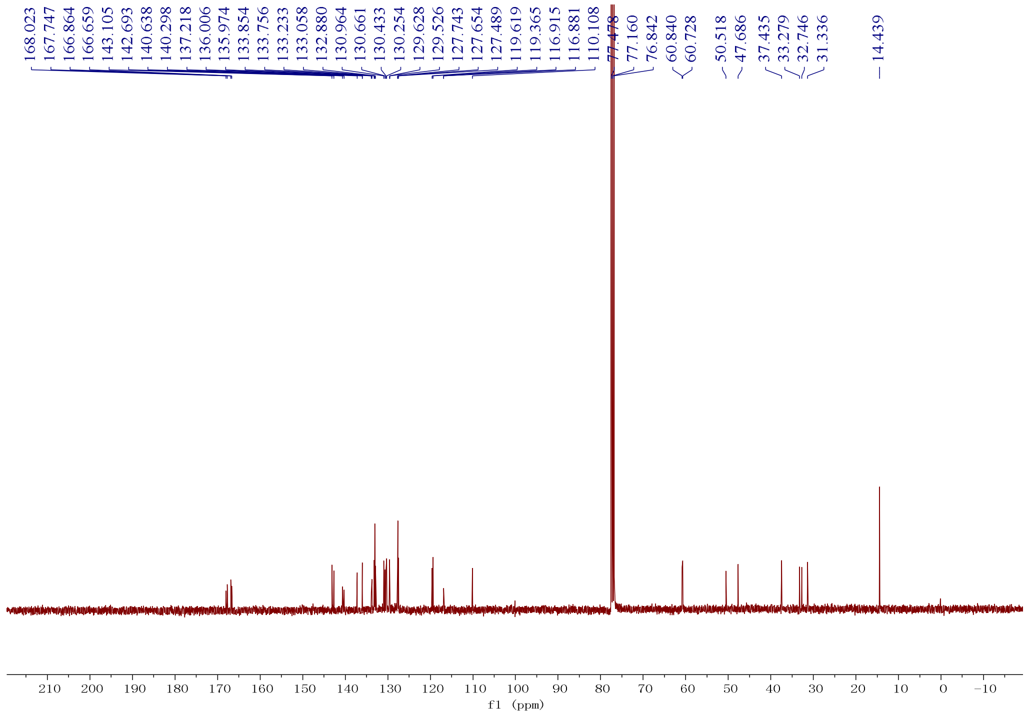
168.023
167.747
166.864
166.659
143.105
142.693
140.638
140.298
137.218
136.006
135.974
133.854
133.756
133.233
133.058
132.880
130.964
130.661
130.433
130.254
129.628
129.526
127.743
127.654
127.489
119.619
119.365
116.915
116.881
110.108
77.478
77.160
76.842
60.840
60.728
50.518
47.686
37.435
33.279
32.746
31.336
14.439
f1 (ppm)

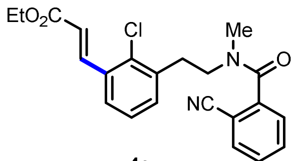
EtO2C
Cl
Me
N
O
NC

4e$_{mono}$ (*m'*)

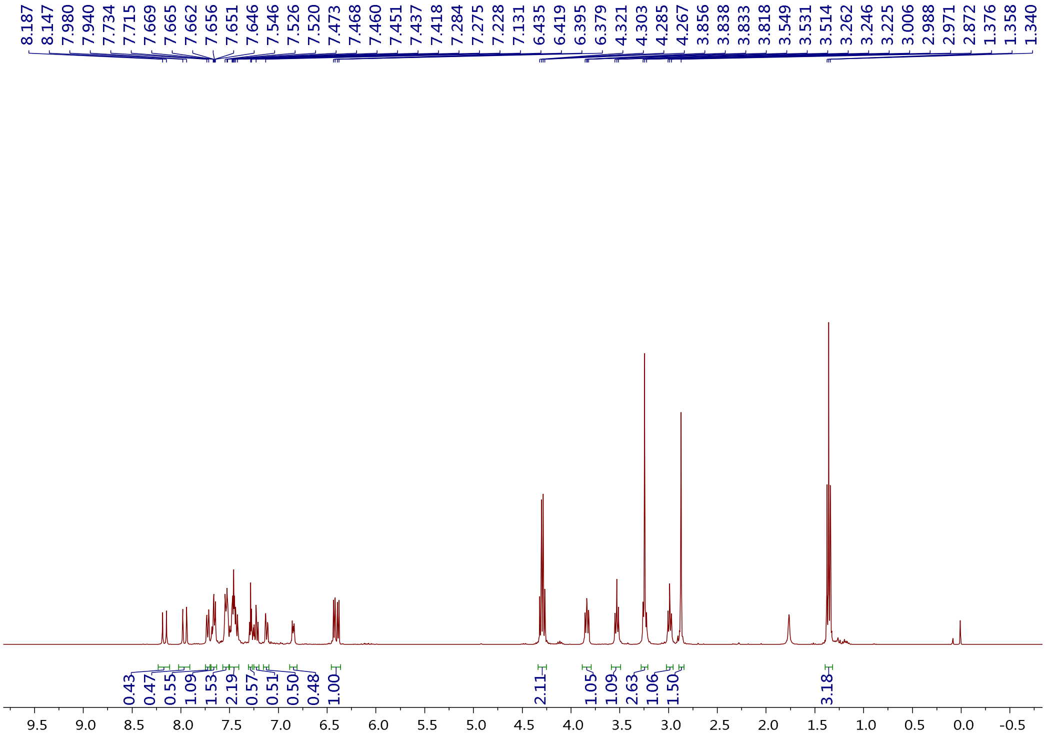
8.187 8.147 7.980 7.940 7.734 7.715 7.669 7.665 7.662 7.656 7.651 7.646 7.546 7.526 7.520 7.473 7.468 7.460 7.451 7.437 7.418 7.284 7.275 7.228 7.131 6.435 6.419 6.395 6.379 4.321 4.303 4.285 4.267 3.856 3.838 3.833 3.818 3.549 3.531 3.514 3.262 3.246 3.225 3.006 2.988 2.971 2.872 1.376 1.358 1.340
0.43 0.47 0.55 1.09 1.53 2.19 0.57 0.51 0.50 0.48 1.00 2.11 1.05 1.09 2.63 1.06 1.50 3.18
9.5 9.0 8.5 8.0 7.5 7.0 6.5 6.0 5.5 5.0 4.5 4.0 3.5 3.0 2.5 2.0 1.5 1.0 0.5 0.0 -0.5

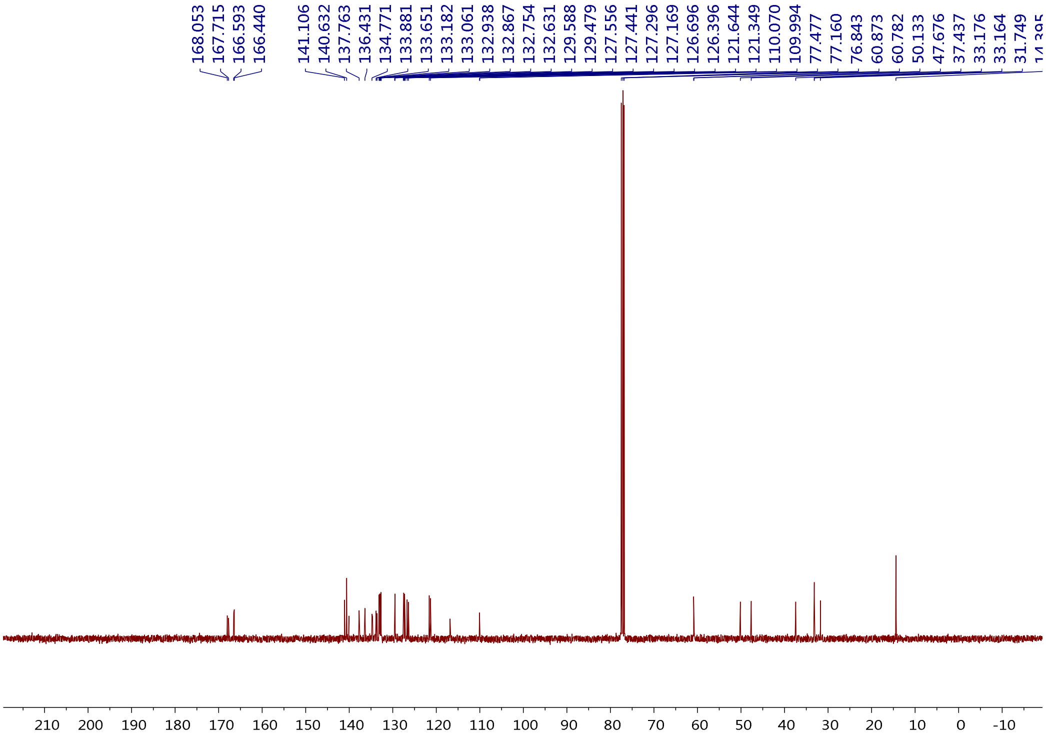
168.053 167.715 166.593 166.440 141.106 140.632 137.763 136.431 134.771 133.881 133.651 133.182 133.061 132.938 132.867 132.754 132.631 129.588 129.479 127.556 127.441 127.296 127.169 126.696 126.396 121.644 121.349 110.070 109.994 77.477 77.160 76.843 60.873 60.782 50.133 47.676 37.437 33.176 33.164 31.749
210 200 190 180 170 160 150 140 130 120 110 100 90 80 70 60 50 40 30 20 10 0 -10

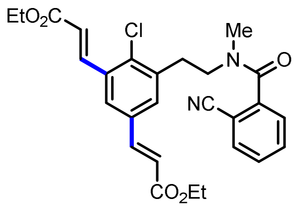
EtO2C
Cl
Me
N
O
NC
CO2Et

4e$_{(m,m')di}$

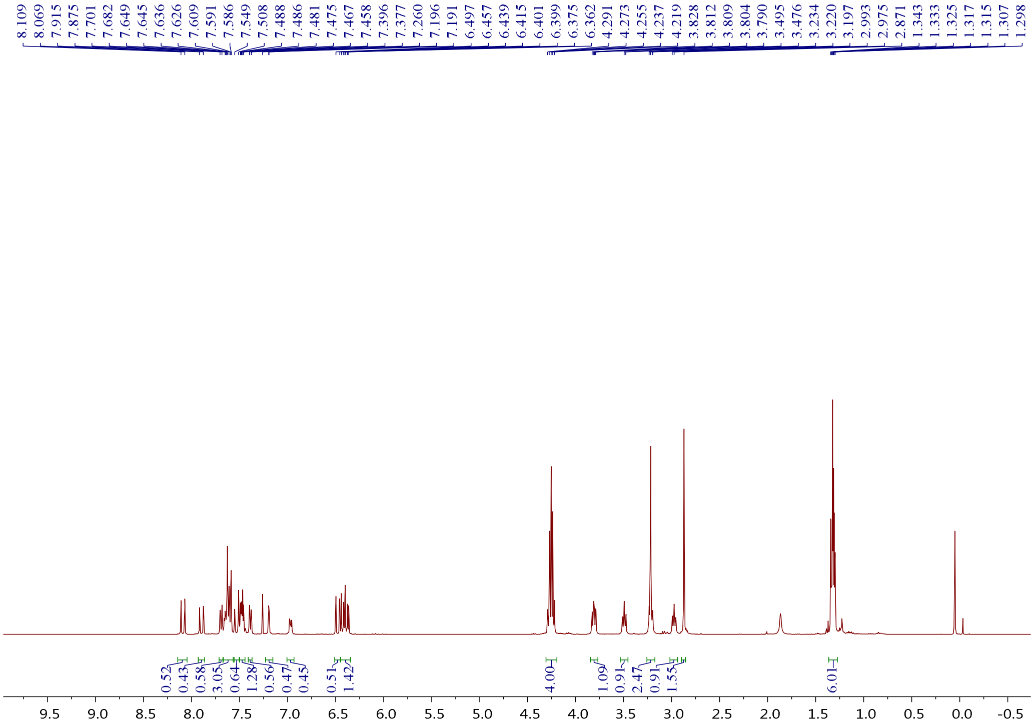
8.109 8.069 7.915 7.875 7.701 7.682 7.649 7.645 7.636 7.626 7.609 7.591 7.586 7.549 7.508 7.488 7.486 7.481 7.475 7.467 7.458 7.396 7.377 7.260 7.196 7.191 6.497 6.457 6.439 6.415 6.401 6.399 6.375 6.362 4.291 4.273 4.255 4.237 4.219 3.828 3.812 3.809 3.804 3.790 3.495 3.476 3.234 3.220 3.197 2.993 2.975 2.871 1.343 1.333 1.325 1.317 1.315 1.307 1.298
0.52 0.43 0.58 3.05 0.64 1.28 0.56 0.47 0.45 0.51 1.42 4.00 1.09 0.91 2.47 0.91 1.55 6.01
9.5 9.0 8.5 8.0 7.5 7.0 6.5 6.0 5.5 5.0 4.5 4.0 3.5 3.0 2.5 2.0 1.5 1.0 0.5 0.0 -0.5

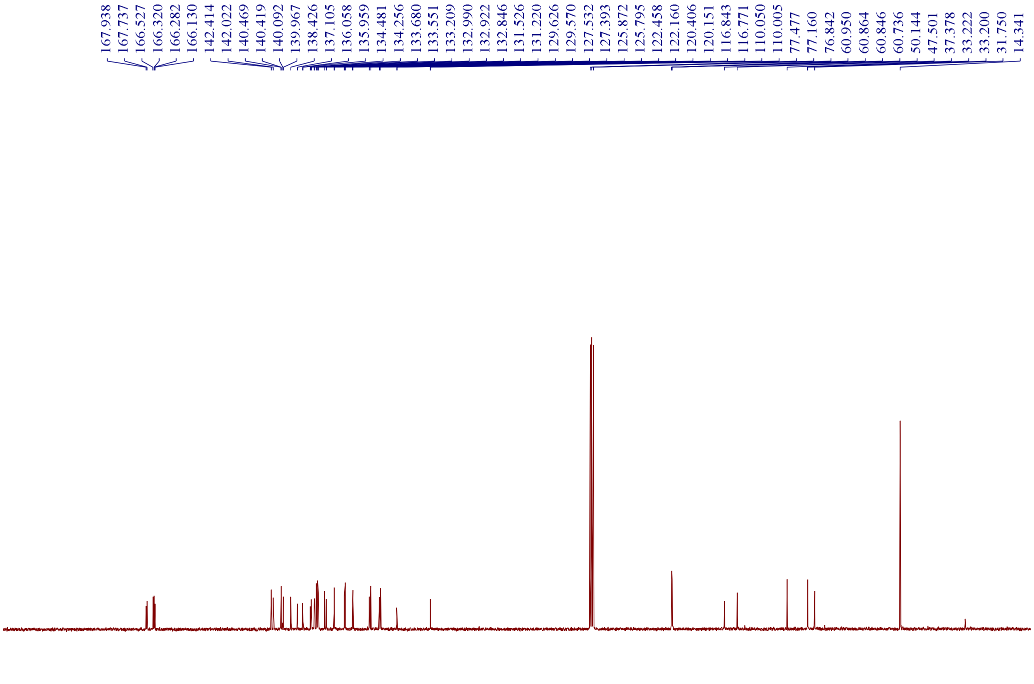
167.938 167.737 166.527 166.320 166.282 166.130 142.414 142.022 140.469 140.419 140.092 139.967 138.426 137.105 136.058 135.959 134.481 134.256 133.680 133.551 133.209 132.990 132.922 132.846 131.526 131.220 129.626 129.570 127.532 127.393 125.872 125.795 122.458 122.160 120.406 120.151 116.843 116.771 110.050 110.005 77.477 77.160 76.842 60.950 60.864 60.846 60.736 50.144 47.501 37.378 33.222 33.200 31.750 14.341
190 180 170 160 150 140 130 120 110 100 90 80 70 60 50 40 30 20 10 0 -10

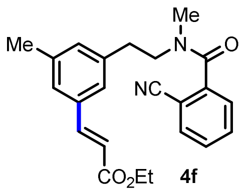

Me
Me
N
O
NC
CO2Et

4f

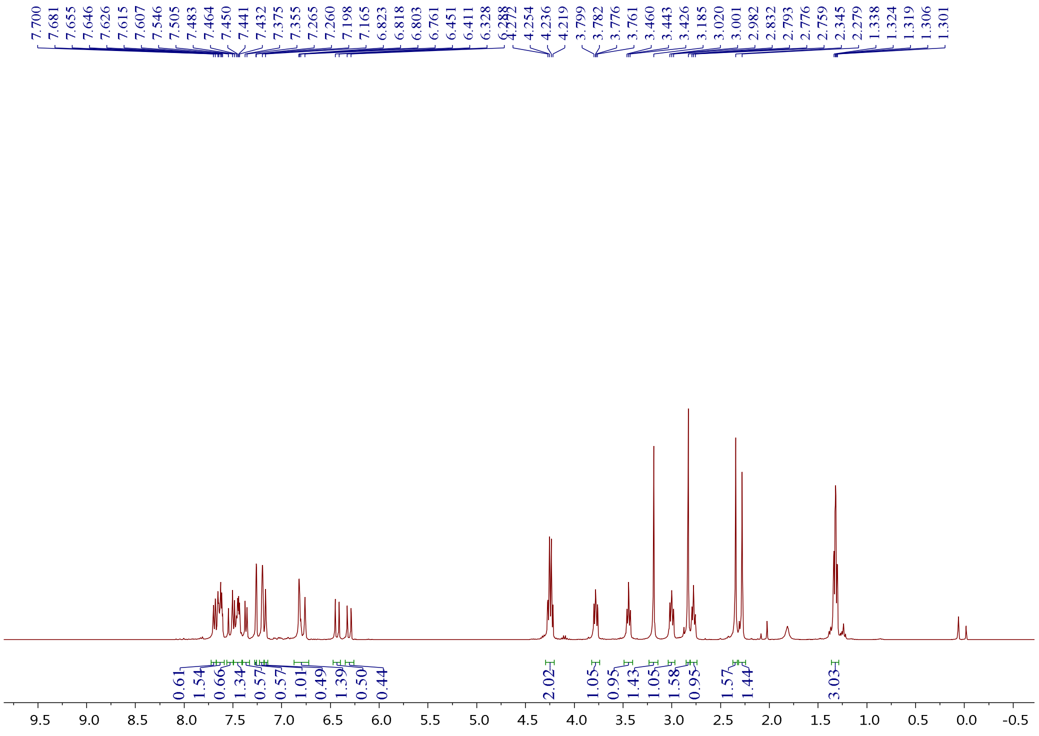

7.700
7.681
7.655
7.646
7.626
7.615
7.607
7.546
7.505
7.483
7.464
7.450
7.441
7.432
7.375
7.355
7.265
7.260
7.198
7.165
6.823
6.818
6.803
6.761
6.451
6.411
6.328
4.254
4.236
4.219
3.799
3.782
3.776
3.761
3.460
3.443
3.426
3.185
3.020
3.001
2.982
2.832
2.793
2.776
2.759
2.345
2.279
1.338
1.324
1.319
1.306
1.301
0.61
1.54
0.66
1.34
0.57
0.57
1.01
0.49
1.39
0.50
0.44
2.02
1.05
0.95
1.43
1.05
1.58
0.95
1.57
1.44
3.03
9.5
9.0
8.5
8.0
7.5
7.0
6.5
6.0
5.5
5.0
4.5
4.0
3.5
3.0
2.5
2.0
1.5
1.0
0.5
0.0
-0.5

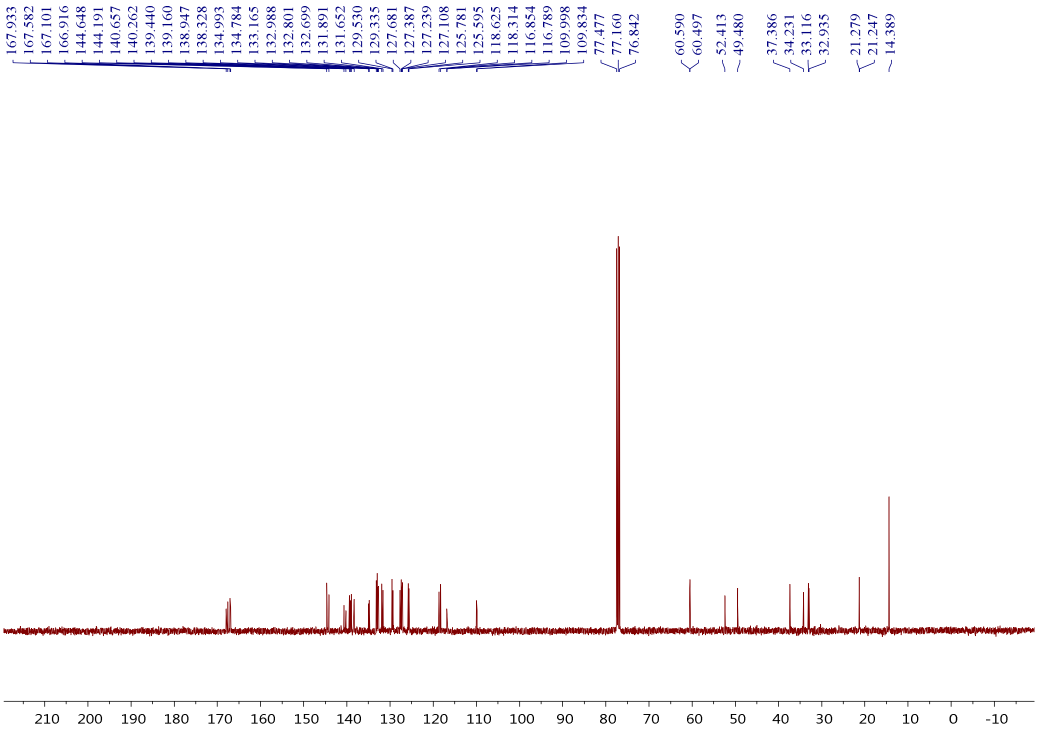

167.933
167.582
167.101
166.916
144.648
144.191
140.657
140.262
139.440
139.160
138.947
138.328
134.993
134.784
133.165
132.988
132.801
132.699
131.891
131.652
129.530
129.335
127.681
127.387
127.239
127.108
125.781
125.595
118.625
118.314
116.854
116.789
109.998
109.834
77.477
77.160
76.842
60.590
60.497
52.413
49.480
37.386
34.231
33.116
32.935
21.279
21.247
14.389
210
200
190
180
170
160
150
140
130
120
110
100
90
80
70
60
50
40
30
20
10
0
-10

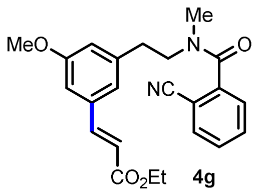
MeO
Me
N
O
NC
CO_2Et

4g

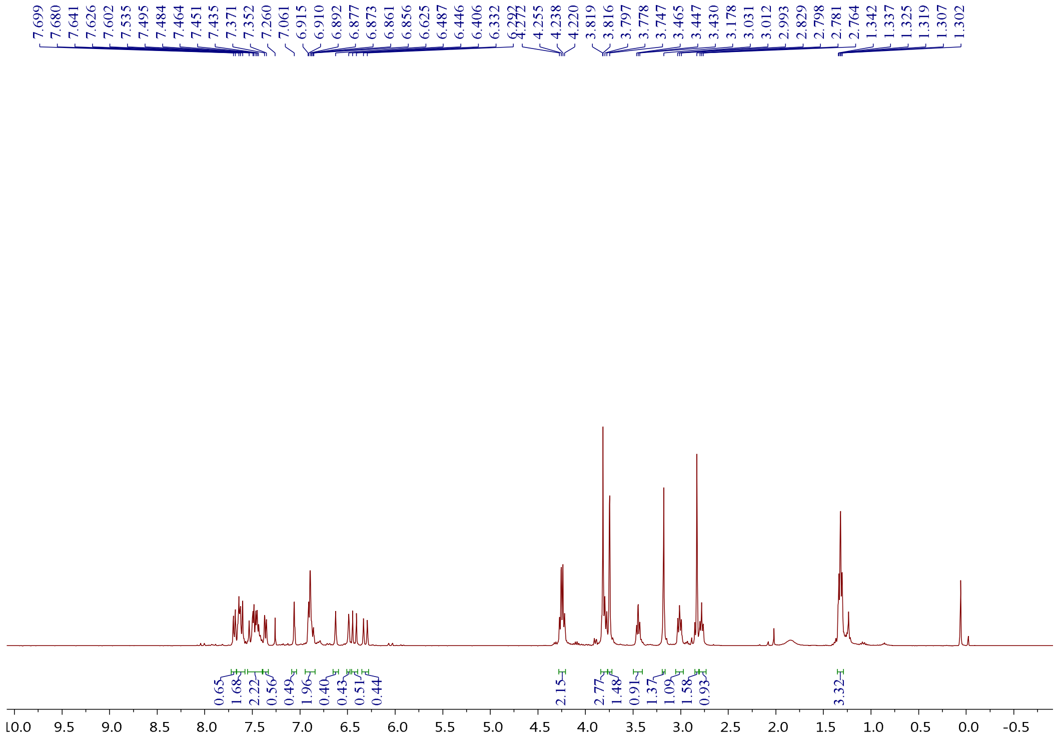
7.699
7.680
7.641
7.626
7.602
7.535
7.495
7.484
7.464
7.451
7.435
7.371
7.352
7.260
7.061
6.915
6.910
6.892
6.877
6.873
6.861
6.856
6.625
6.487
6.446
6.406
6.332
6.292
4.272
4.255
4.238
4.220
3.819
3.816
3.797
3.778
3.747
3.465
3.447
3.430
3.178
3.031
3.012
2.993
2.829
2.798
2.781
2.764
1.342
1.337
1.325
1.319
1.307
1.302
0.65
1.68
2.22
0.56
0.49
1.96
0.40
0.43
0.51
0.44
2.15
2.77
1.48
0.91
1.37
1.09
1.58
0.93
3.32
10.0 9.5 9.0 8.5 8.0 7.5 7.0 6.5 6.0 5.5 5.0 4.5 4.0 3.5 3.0 2.5 2.0 1.5 1.0 0.5 0.0 -0.5

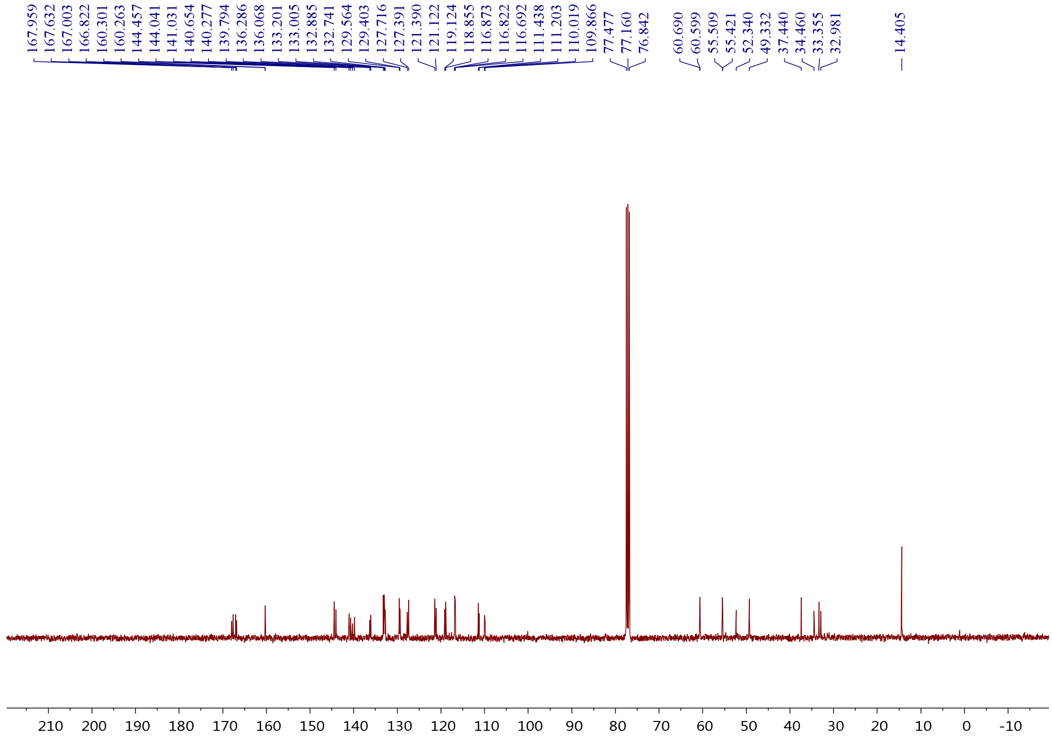
167.959
167.632
167.003
166.822
160.301
160.263
144.457
144.041
141.031
140.654
140.277
139.794
136.286
136.068
133.201
133.005
132.885
132.741
129.564
129.403
127.716
127.391
121.390
121.122
119.124
118.855
116.873
116.822
116.692
111.438
111.203
110.019
109.866
77.477
77.160
76.842
60.690
60.599
55.509
55.421
52.340
49.332
37.440
34.460
33.355
32.981
14.405
210 200 190 180 170 160 150 140 130 120 110 100 90 80 70 60 50 40 30 20 10 0 -10

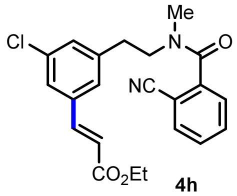
Cl
Me
N
O
NC
CO2Et

4h

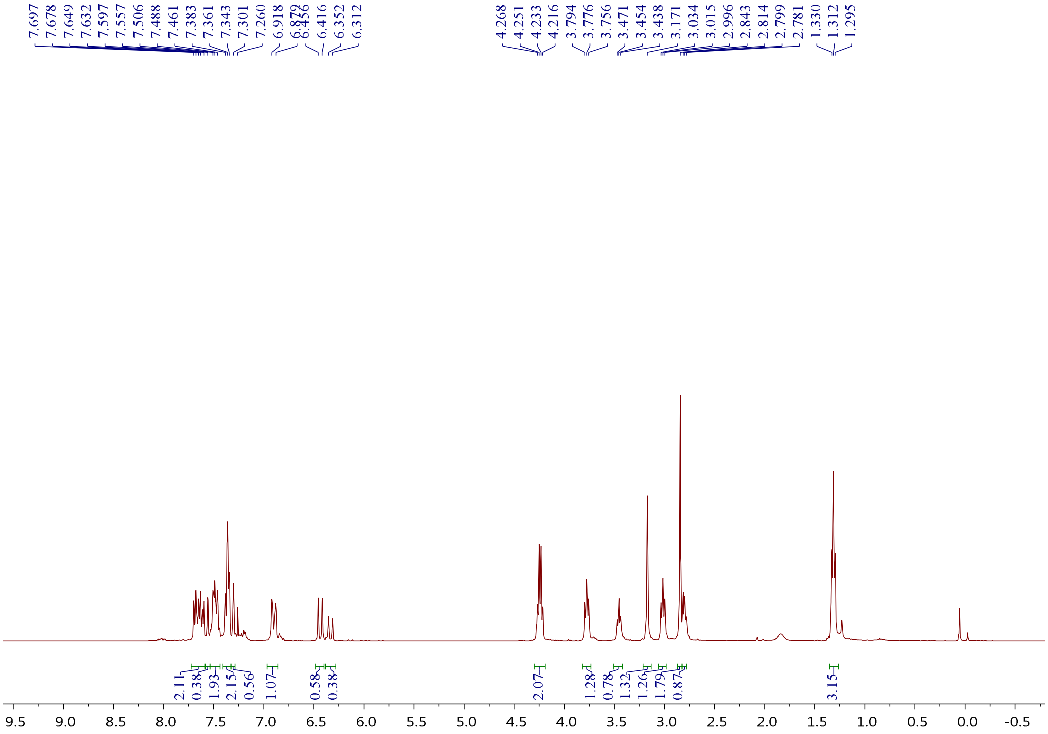
7.697
7.678
7.649
7.632
7.597
7.557
7.506
7.488
7.461
7.383
7.361
7.343
7.301
7.260
6.918
6.416
6.352
6.312
4.268
4.251
4.233
4.216
3.794
3.776
3.756
3.471
3.454
3.438
3.171
3.034
3.015
2.996
2.843
2.814
2.799
2.781
1.330
1.312
1.295
2.11
0.38
1.93
2.15
0.56
1.07
0.58
0.38
2.07
1.28
0.78
1.32
1.26
1.79
0.87
3.15
9.5
9.0
8.5
8.0
7.5
7.0
6.5
6.0
5.5
5.0
4.5
4.0
3.5
3.0
2.5
2.0
1.5
1.0
0.5
0.0
-0.5

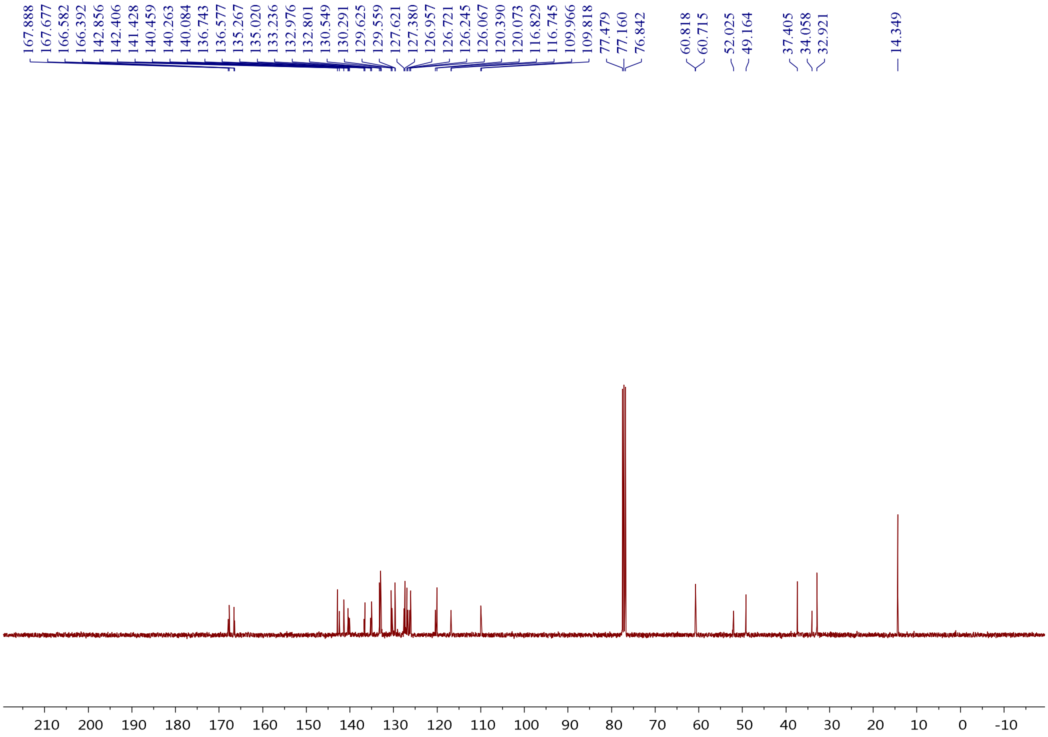
167.888
167.677
166.582
166.392
142.856
142.406
141.428
140.459
140.263
140.084
136.743
136.577
135.267
135.020
133.236
132.976
132.801
130.549
130.291
129.625
129.559
127.621
127.380
126.957
126.721
126.245
126.067
120.390
120.073
116.829
116.745
109.966
109.818
77.479
77.160
76.842
60.818
60.715
52.025
49.164
37.405
34.058
32.921
14.349
210
200
190
180
170
160
150
140
130
120
110
100
90
80
70
60
50
40
30
20
10
0
-10

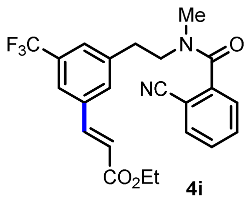

Me
F3C
N
O
NC
CO2Et

4i

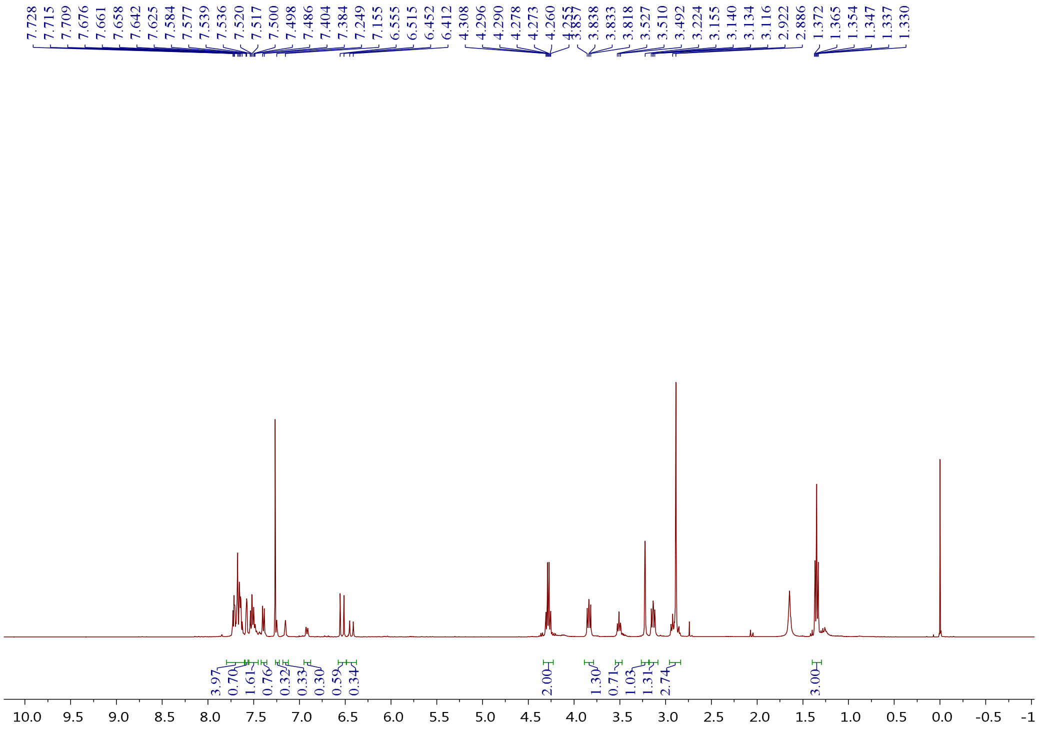

7.728 7.715 7.709 7.676 7.661 7.658 7.642 7.625 7.584 7.577 7.539 7.536 7.520 7.517 7.500 7.498 7.486 7.404 7.384 7.249 7.155 6.555 6.515 6.452 6.412 4.308 4.296 4.290 4.278 4.273 4.260 4.255 3.857 3.838 3.833 3.818 3.527 3.510 3.492 3.224 3.155 3.140 3.134 3.116 2.922 2.886 1.372 1.365 1.354 1.347 1.337 1.330
3.97 0.70 1.61 0.76 0.32 0.33 0.30 0.59 0.34 2.00 1.30 0.71 1.03 1.31 2.74 3.00
10.0 9.5 9.0 8.5 8.0 7.5 7.0 6.5 6.0 5.5 5.0 4.5 4.0 3.5 3.0 2.5 2.0 1.5 1.0 0.5 0.0 -0.5 -1

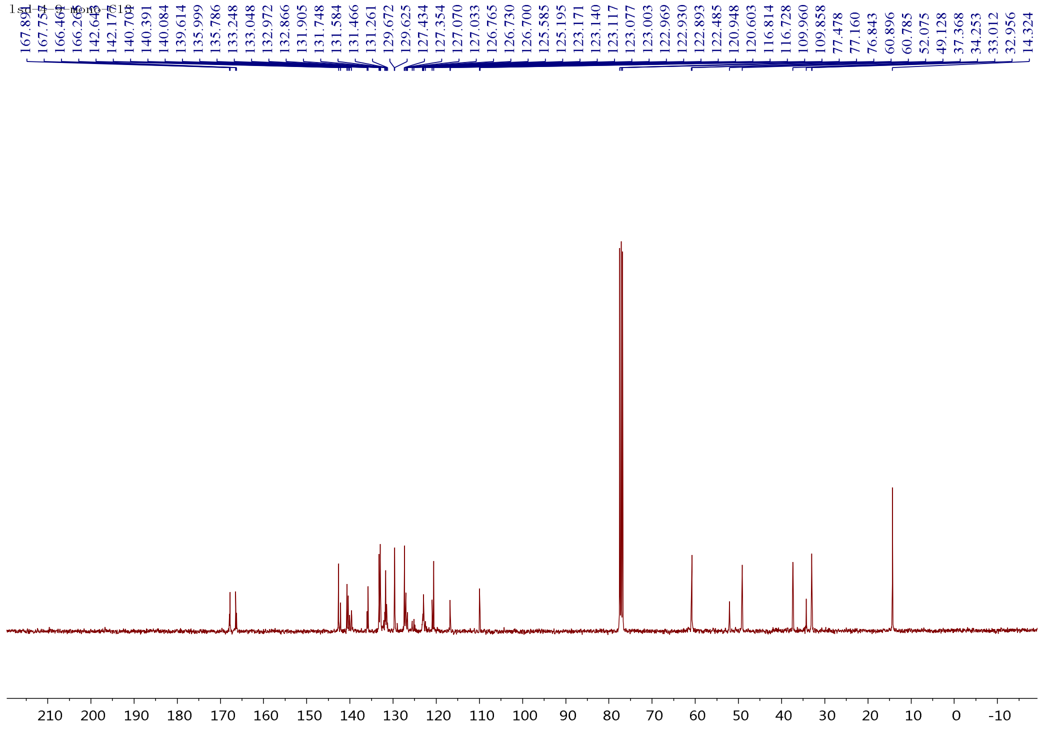

167.754 166.464 166.262 142.647 142.177 140.703 140.391 140.084 139.614 135.999 135.786 133.248 133.048 132.972 132.866 131.905 131.748 131.584 131.466 131.261 129.672 129.625 127.434 127.354 127.070 127.033 126.765 126.730 126.700 125.585 125.195 123.171 123.140 123.117 123.077 123.003 122.969 122.930 122.893 122.485 120.948 120.603 116.814 116.728 109.960 109.858 77.478 77.160 76.843 60.896 60.785 52.075 49.128 37.368 34.253 33.012 32.956 14.324
210 200 190 180 170 160 150 140 130 120 110 100 90 80 70 60 50 40 30 20 10 0 -10

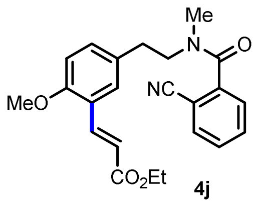
Me
N
O
NC
MeO
CO2Et

4j

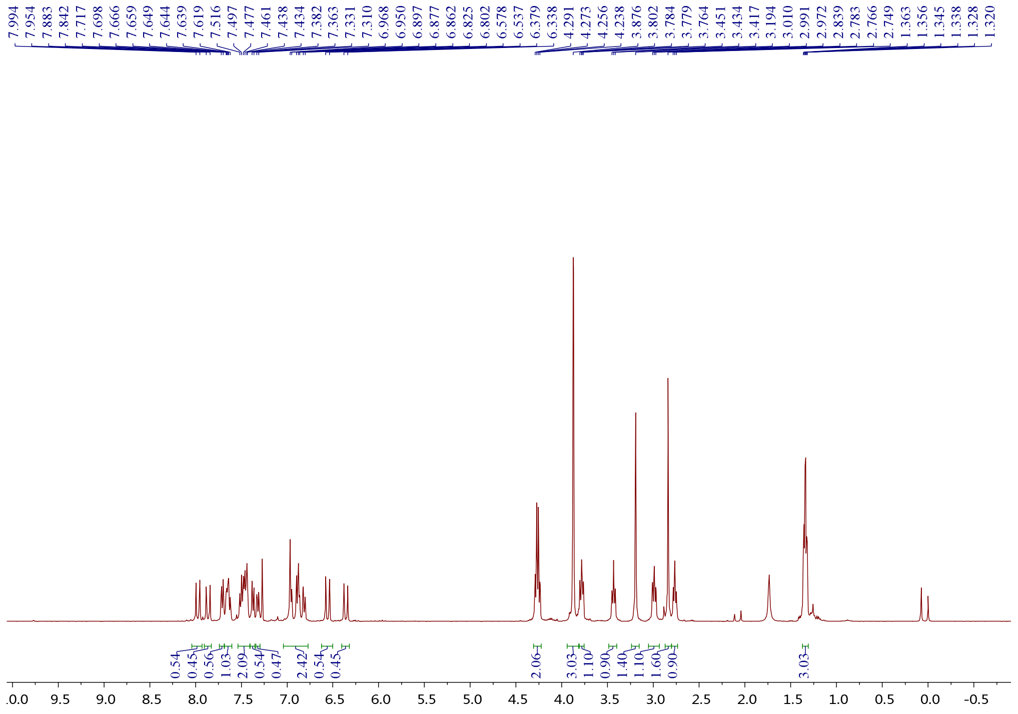
7.994
7.954
7.883
7.842
7.717
7.698
7.666
7.659
7.649
7.644
7.639
7.619
7.516
7.497
7.477
7.461
7.438
7.434
7.382
7.363
7.331
7.310
6.968
6.950
6.897
6.877
6.862
6.825
6.802
6.578
6.537
6.379
6.338
4.291
4.273
4.256
4.238
3.876
3.802
3.784
3.779
3.764
3.451
3.434
3.417
3.194
3.010
2.991
2.972
2.839
2.783
2.766
2.749
1.363
1.356
1.345
1.338
1.328
1.320
0.54
0.45
0.56
1.03
2.09
0.54
0.47
2.42
0.54
0.45
2.06
3.03
1.10
0.90
1.40
1.10
1.60
0.90
3.03
.0.0 9.5 9.0 8.5 8.0 7.5 7.0 6.5 6.0 5.5 5.0 4.5 4.0 3.5 3.0 2.5 2.0 1.5 1.0 0.5 0.0 -0.5

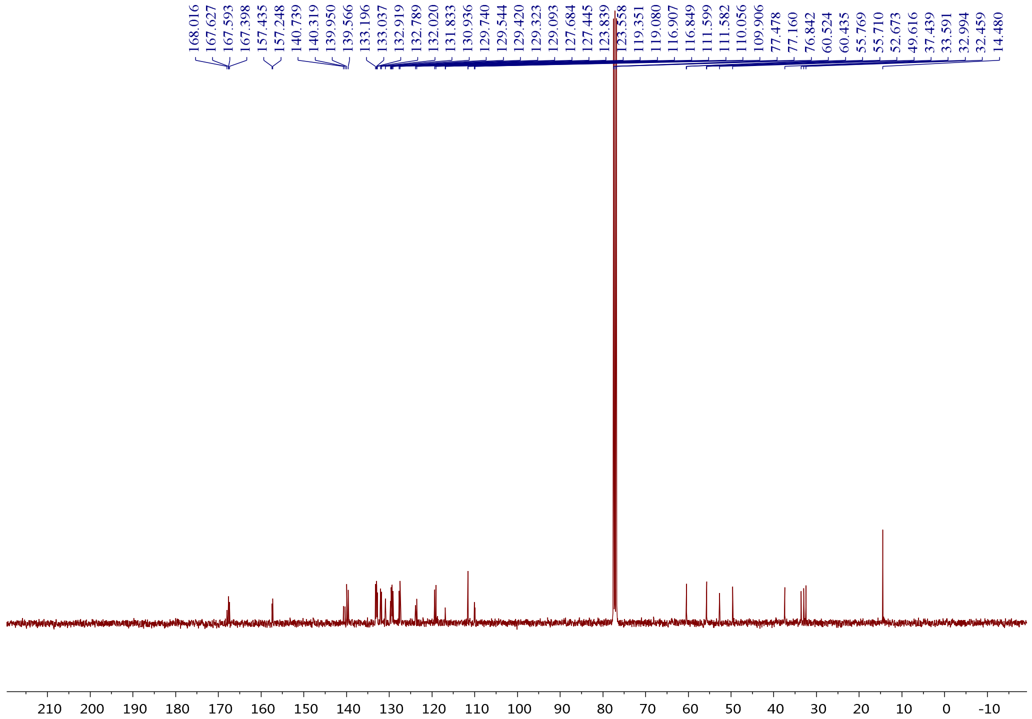
168.016
167.627
167.593
167.398
157.435
157.248
140.739
140.319
139.950
139.566
133.196
133.037
132.919
132.789
132.020
131.833
130.936
129.740
129.544
129.420
129.323
129.093
127.684
127.445
123.839
123.558
119.351
119.080
116.907
116.849
111.599
111.582
110.056
109.906
77.478
77.160
76.842
60.524
60.435
55.769
55.710
52.673
49.616
37.439
33.591
32.994
32.459
14.480
210 200 190 180 170 160 150 140 130 120 110 100 90 80 70 60 50 40 30 20 10 0 -10

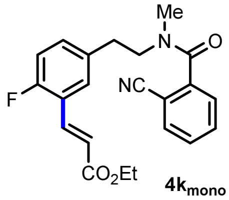
Me
N
O
NC
F
CO2Et

4k$_{mono}$

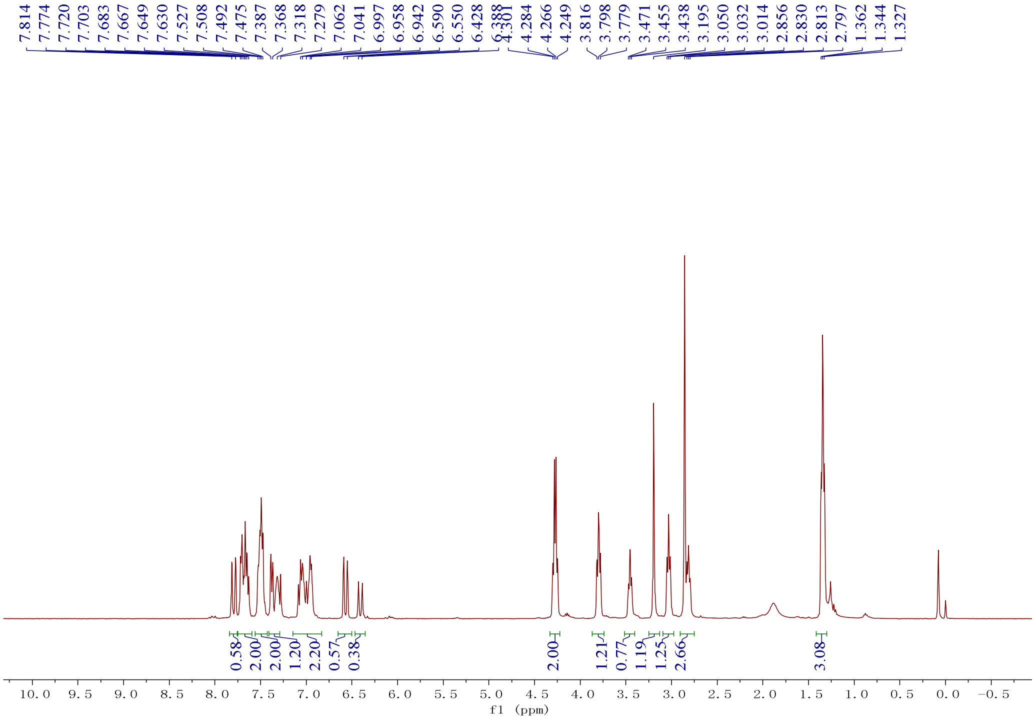
7.814
7.774
7.720
7.703
7.683
7.667
7.649
7.630
7.527
7.508
7.492
7.475
7.387
7.368
7.318
7.279
7.062
7.041
6.997
6.958
6.942
6.590
6.550
6.428
6.388
4.301
4.284
4.266
4.249
3.816
3.798
3.779
3.471
3.455
3.438
3.195
3.050
3.032
3.014
2.856
2.830
2.813
2.797
1.362
1.344
1.327
0.58
2.00
2.00
1.20
2.20
0.57
0.38
2.00
1.21
0.77
1.19
1.25
2.66
3.08
f1 (ppm)

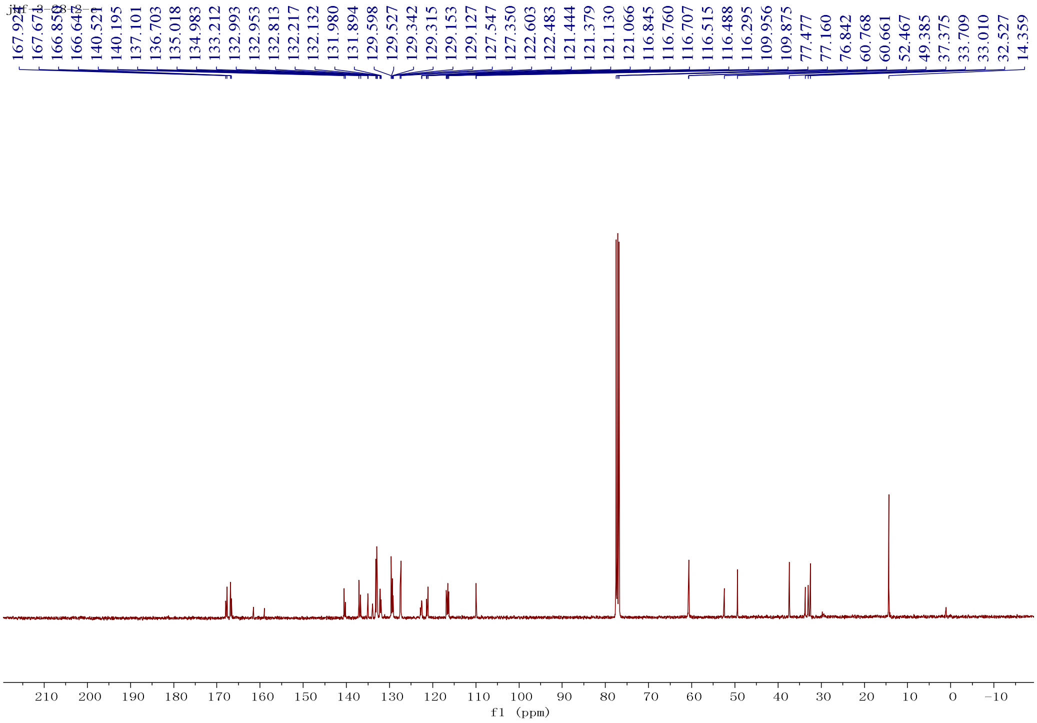
167.924
167.671
166.850
166.643
140.521
140.195
137.101
136.703
135.018
134.983
133.212
132.993
132.953
132.813
132.217
132.132
131.980
131.894
129.598
129.527
129.342
129.315
129.153
129.127
127.547
127.350
122.603
122.483
121.444
121.379
121.130
121.066
116.845
116.760
116.707
116.515
116.488
116.295
109.956
109.875
77.477
77.160
76.842
60.768
60.661
52.467
49.385
37.375
33.709
33.010
32.527
14.359
f1 (ppm)

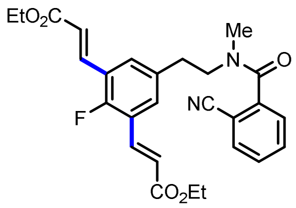
EtO2C
Me
N
O
NC
F
CO2Et

4k$_{(m,m')di}$

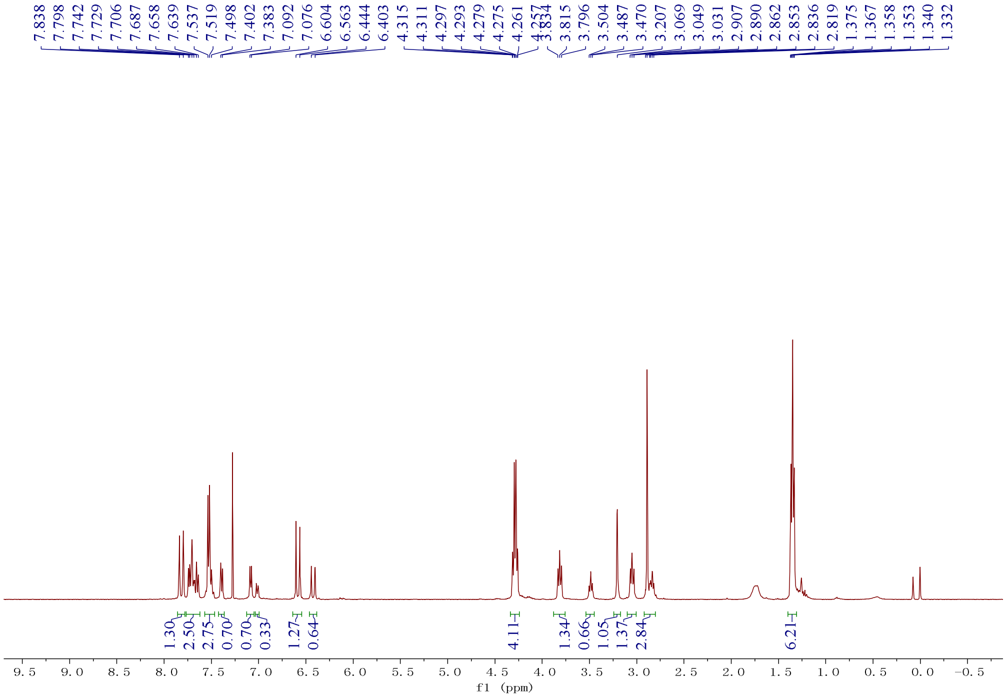
7.838
7.798
7.742
7.729
7.706
7.687
7.658
7.639
7.537
7.519
7.498
7.402
7.383
7.092
7.076
6.604
6.563
6.444
6.403
4.315
4.311
4.297
4.293
4.279
4.275
4.261
4.257
3.834
3.815
3.796
3.504
3.487
3.470
3.207
3.069
3.049
3.031
2.907
2.890
2.862
2.853
2.836
2.819
1.375
1.367
1.358
1.353
1.340
1.332
1.30
2.50
2.75
0.70
0.70
0.33
1.27
0.64
4.11
1.34
0.66
1.05
1.37
2.84
6.21
9.5 9.0 8.5 8.0 7.5 7.0 6.5 6.0 5.5 5.0 4.5 4.0 3.5 3.0 2.5 2.0 1.5 1.0 0.5 0.0 -0.5
f1 (ppm)

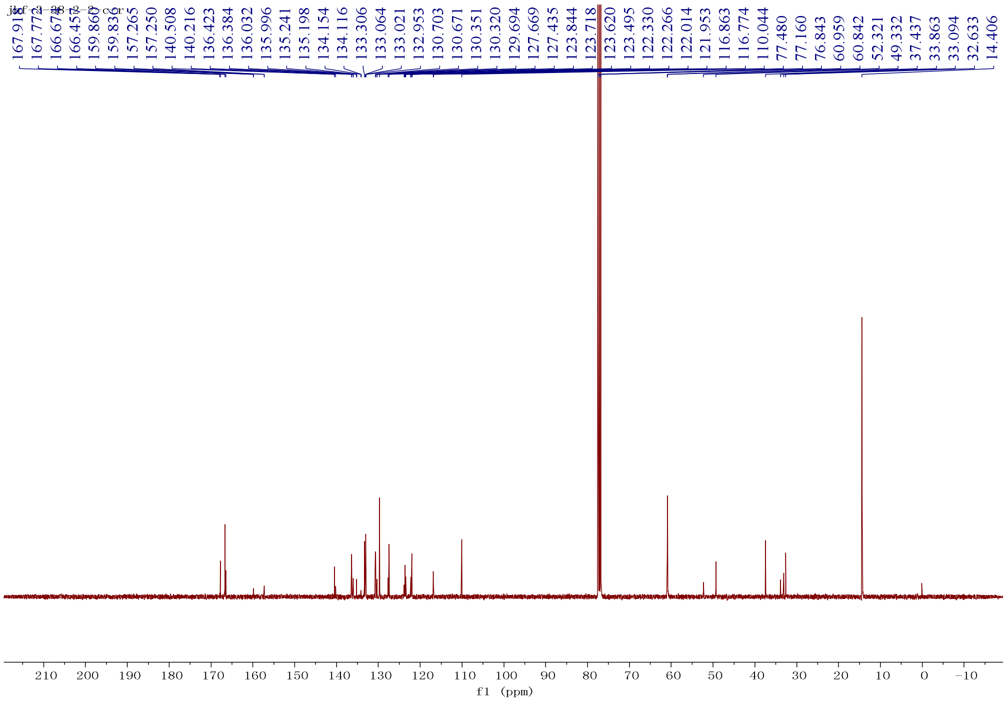
167.91
166.67
166.457
159.860
159.836
157.265
157.250
140.508
140.216
136.423
136.384
136.032
135.996
135.241
135.198
134.154
134.116
133.306
133.064
133.021
132.953
130.703
130.671
130.351
130.320
129.694
127.669
127.435
123.844
123.718
123.620
123.495
122.330
122.266
122.014
121.953
116.863
116.774
110.044
77.480
77.160
76.843
60.959
60.842
52.321
49.332
37.437
33.863
33.094
32.633
14.406
210 200 190 180 170 160 150 140 130 120 110 100 90 80 70 60 50 40 30 20 10 0 -10
f1 (ppm)

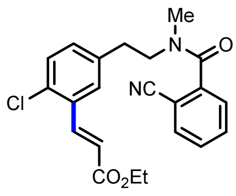
Me
N
O
NC
Cl
CO2Et

4l$_{mono}$

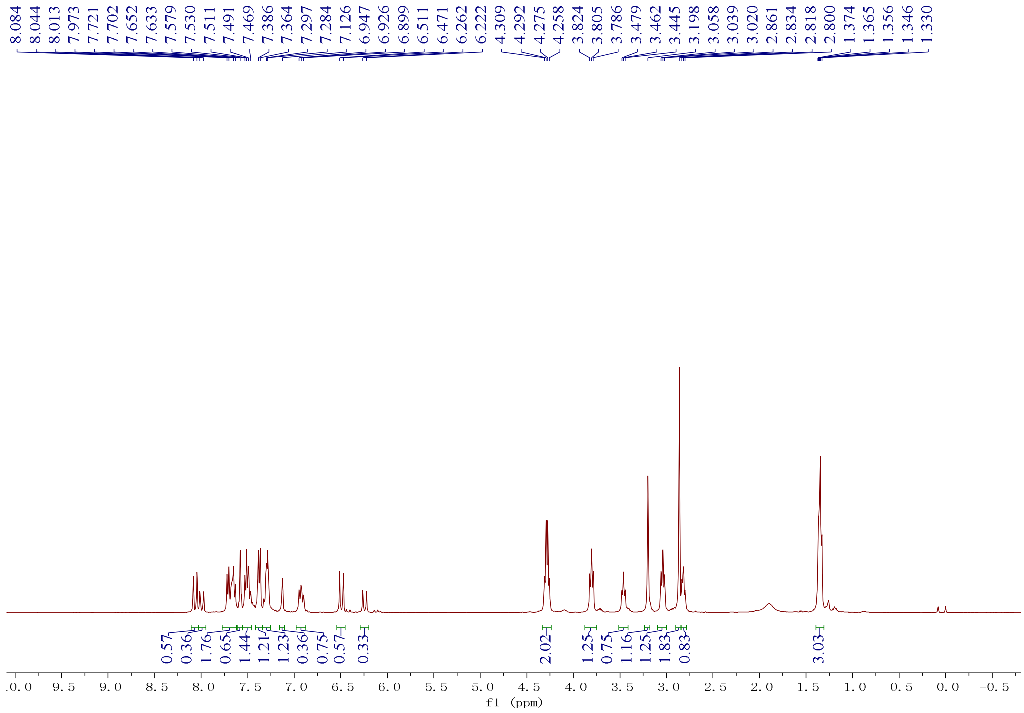
8.084 8.044 8.013 7.973 7.721 7.702 7.652 7.633 7.579 7.530 7.511 7.491 7.469 7.386 7.364 7.297 7.284 7.126 6.947 6.926 6.899 6.511 6.471 6.262 6.222 4.309 4.292 4.275 4.258 3.824 3.805 3.786 3.479 3.462 3.445 3.198 3.058 3.039 3.020 2.861 2.834 2.818 2.800 1.374 1.365 1.356 1.346 1.330
0.57 0.36 1.76 0.65 1.44 1.21 1.23 0.36 0.75 0.57 0.33 2.02 1.25 0.75 1.16 1.25 1.83 0.83 3.03
f1 (ppm)

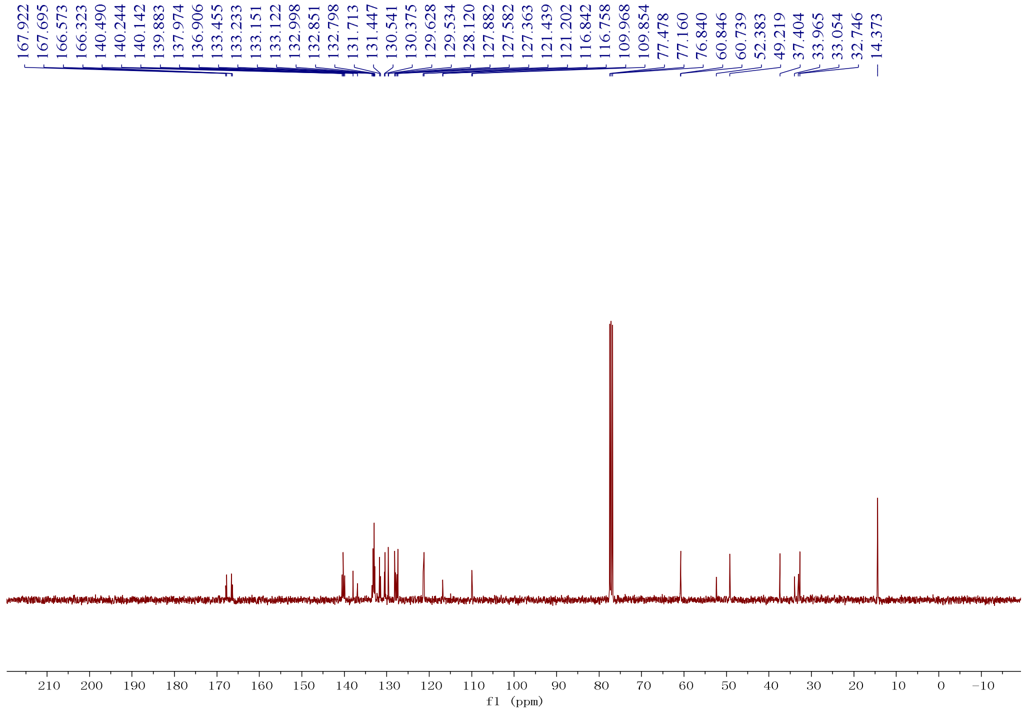
167.922 167.695 166.573 166.323 140.490 140.244 140.142 139.883 137.974 136.906 133.455 133.233 133.151 133.122 132.998 132.851 132.798 131.713 131.447 130.541 130.375 129.628 129.534 128.120 127.882 127.582 127.363 121.439 121.202 116.842 116.758 109.968 109.854 77.478 77.160 76.840 60.846 60.739 52.383 49.219 37.404 33.965 33.054 32.746 14.373
f1 (ppm)

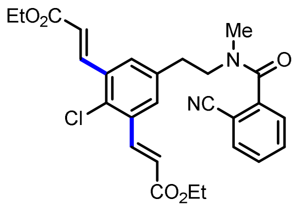
EtO$_2$C
Me
N
O
NC
Cl
CO$_2$Et

4l$_{(m,m')di}$

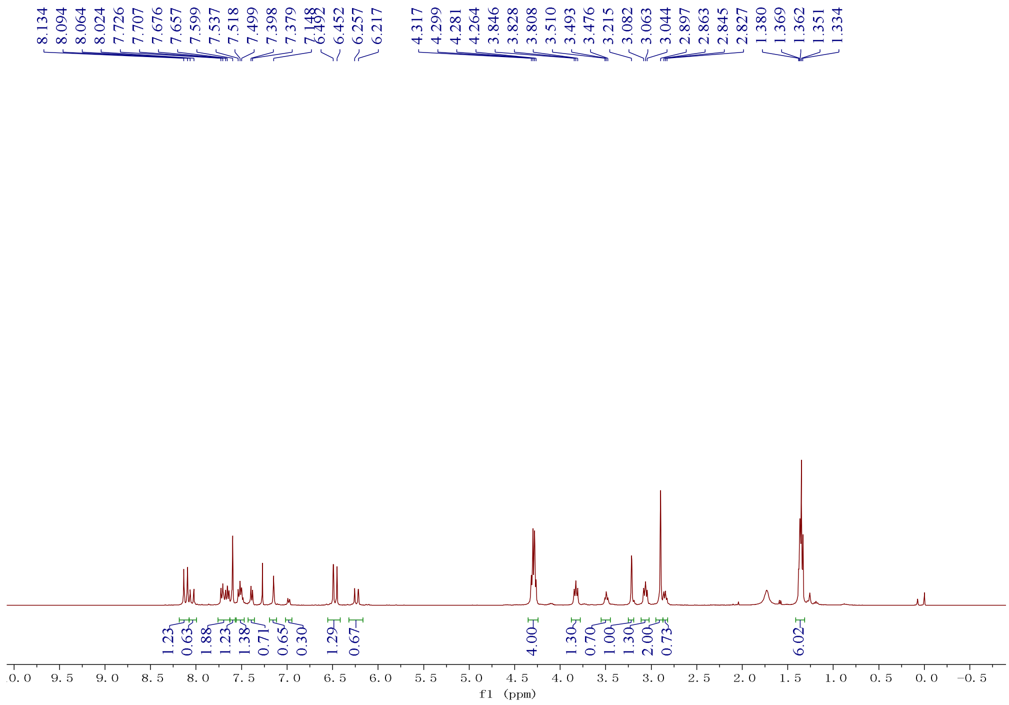
8.134 8.094 8.064 8.024 7.726 7.707 7.676 7.657 7.599 7.537 7.518 7.499 7.398 7.379 7.148 6.492 6.452 6.257 6.217 4.317 4.299 4.281 4.264 3.846 3.828 3.808 3.510 3.493 3.476 3.215 3.082 3.063 3.044 2.897 2.863 2.845 2.827 1.380 1.369 1.362 1.351 1.334
1.23 0.63 1.88 1.23 1.38 0.71 0.65 0.30 1.29 0.67 4.00 1.30 0.70 1.00 1.30 2.00 0.73 6.02
0.0 9.5 9.0 8.5 8.0 7.5 7.0 6.5 6.0 5.5 5.0 4.5 4.0 3.5 3.0 2.5 2.0 1.5 1.0 0.5 0.0 -0.5
f1 (ppm)

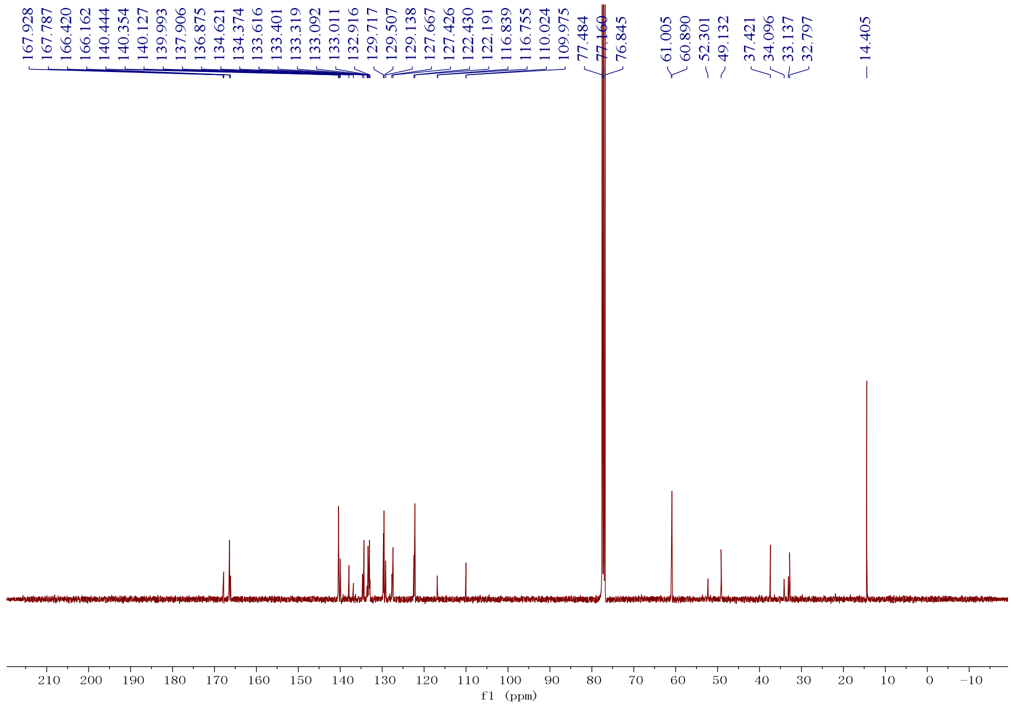
167.928 167.787 166.420 166.162 140.444 140.354 140.127 139.993 137.906 136.875 134.621 134.374 133.616 133.401 133.319 133.092 133.011 132.916 129.717 129.507 129.138 127.667 127.426 122.430 122.191 116.839 116.755 110.024 109.975 77.484 77.160 76.845 61.005 60.890 52.301 49.132 37.421 34.096 33.137 32.797 14.405
210 200 190 180 170 160 150 140 130 120 110 100 90 80 70 60 50 40 30 20 10 0 -10
f1 (ppm)

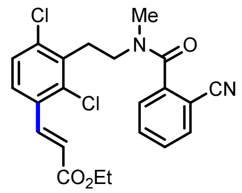
Cl
Me
N
O
Cl
CN
CO_2Et

$4m_{mono}$

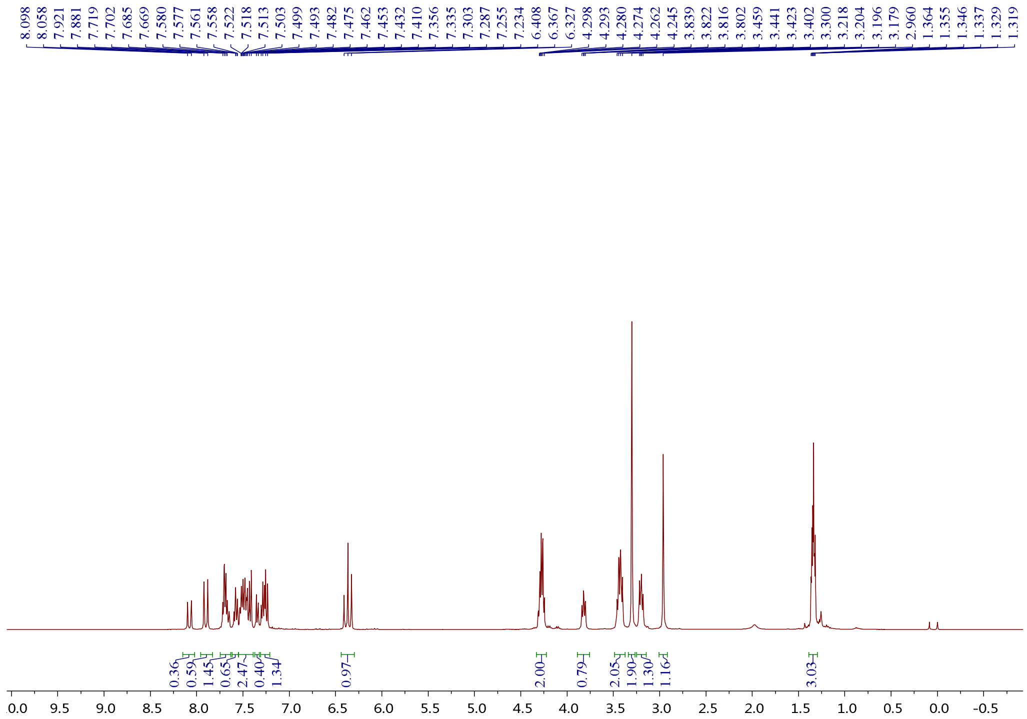
8.098
8.058
7.921
7.881
7.719
7.702
7.685
7.669
7.580
7.577
7.561
7.558
7.522
7.518
7.513
7.503
7.499
7.493
7.482
7.475
7.462
7.453
7.432
7.410
7.356
7.335
7.303
7.287
7.255
7.234
6.408
6.367
6.327
4.298
4.293
4.280
4.274
4.262
4.245
3.839
3.822
3.816
3.802
3.459
3.441
3.423
3.402
3.300
3.218
3.204
3.196
3.179
2.960
1.364
1.355
1.346
1.337
1.329
1.319
0.36
0.59
1.45
0.65
2.47
0.40
1.34
0.97
2.00
0.79
2.05
1.90
1.30
1.16
3.03
0.0 9.5 9.0 8.5 8.0 7.5 7.0 6.5 6.0 5.5 5.0 4.5 4.0 3.5 3.0 2.5 2.0 1.5 1.0 0.5 0.0 -0.5

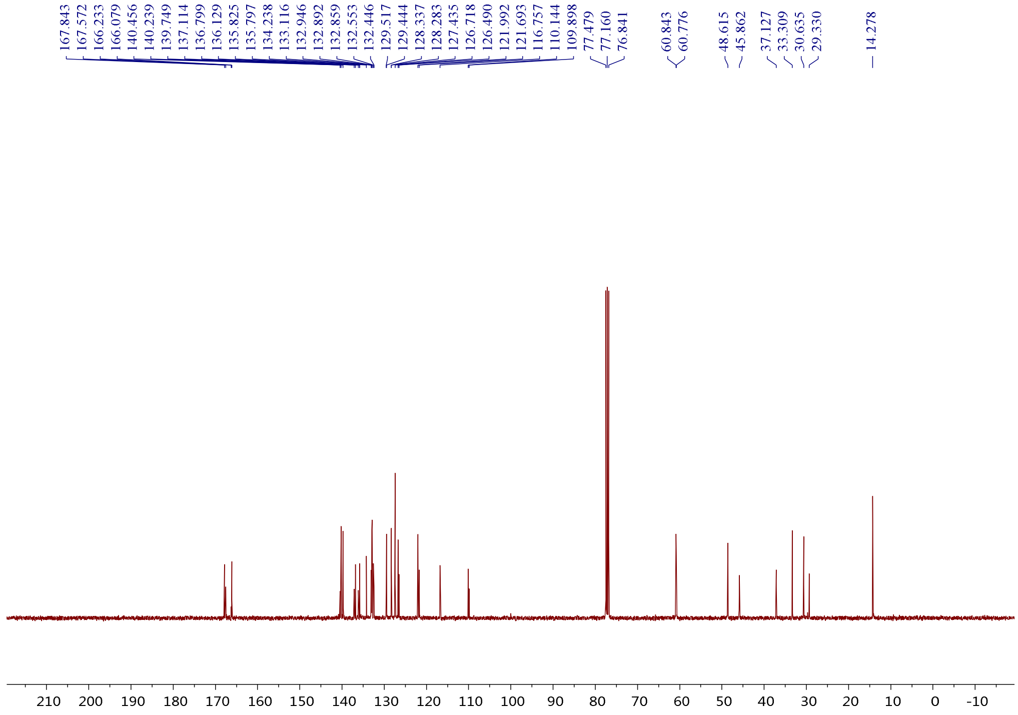
167.843
167.572
166.233
166.079
140.456
140.239
139.749
137.114
136.799
136.129
135.825
135.797
134.238
133.116
132.946
132.892
132.859
132.553
132.446
129.517
129.444
128.337
128.283
127.435
126.718
126.490
121.992
121.693
116.757
110.144
109.898
77.479
77.160
76.841
60.843
60.776
48.615
45.862
37.127
33.309
30.635
29.330
14.278
210 200 190 180 170 160 150 140 130 120 110 100 90 80 70 60 50 40 30 20 10 0 -10

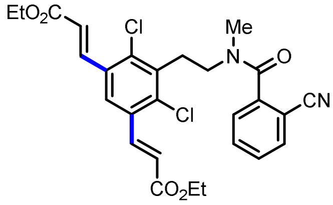

4m$_{(m,m')\mathrm{di}}$

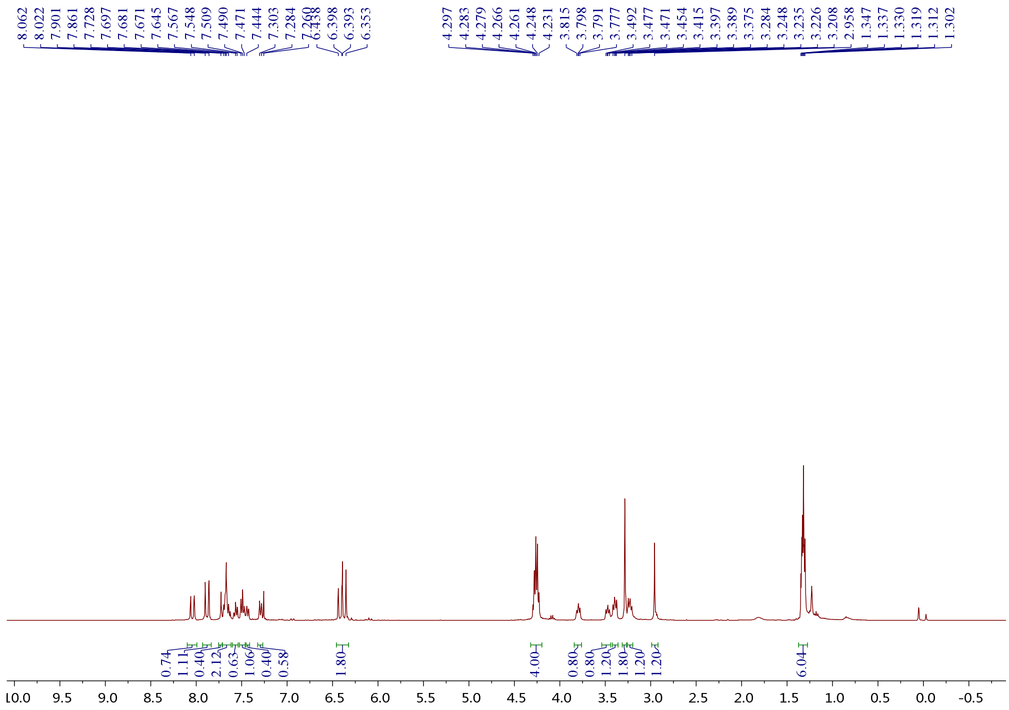

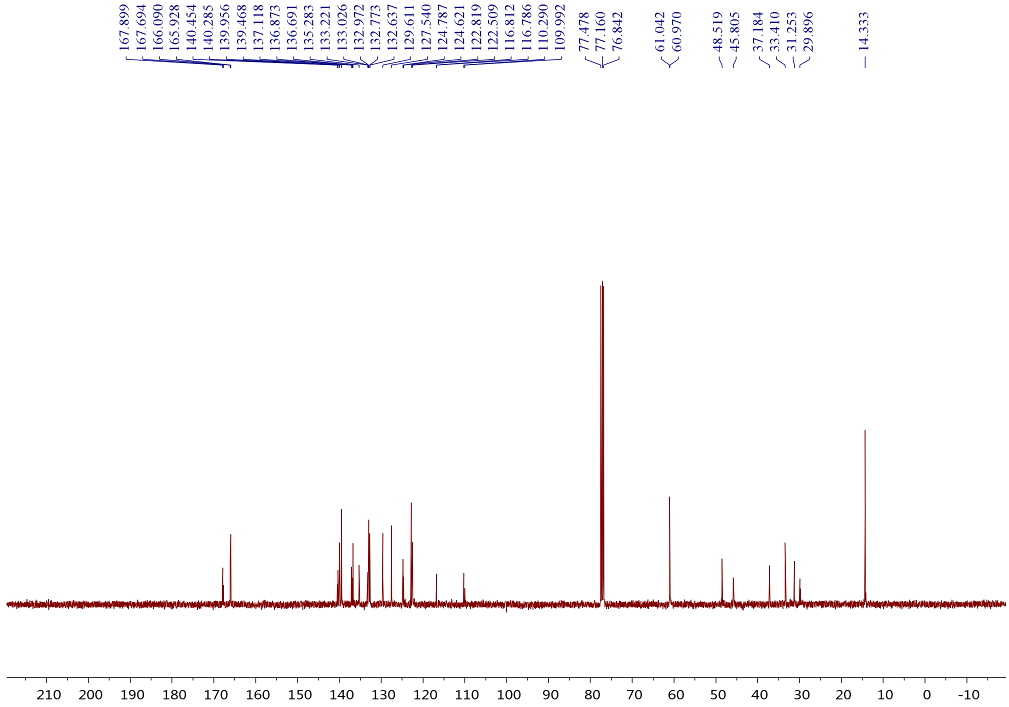

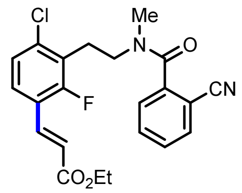
Cl
Me
N
O
CN
F
CO2Et

$4n_{mono\ (m)}$

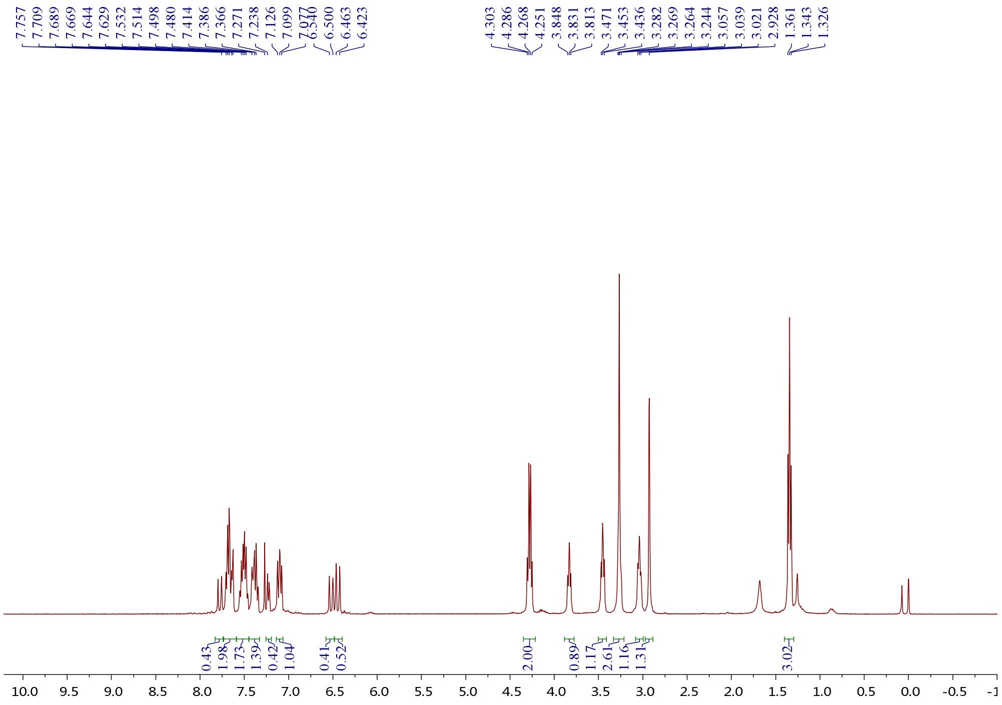
7.757
7.709
7.689
7.669
7.644
7.629
7.532
7.514
7.498
7.480
7.414
7.386
7.366
7.271
7.238
7.126
7.099
7.077
6.540
6.500
6.463
6.423
4.303
4.286
4.268
4.251
3.848
3.831
3.813
3.471
3.453
3.436
3.282
3.269
3.264
3.244
3.057
3.039
3.021
2.928
1.361
1.343
1.326
0.43
1.98
1.73
1.39
0.42
1.04
0.41
0.52
2.00
0.89
1.17
2.61
1.16
1.31
3.02
10.0 9.5 9.0 8.5 8.0 7.5 7.0 6.5 6.0 5.5 5.0 4.5 4.0 3.5 3.0 2.5 2.0 1.5 1.0 0.5 0.0 -0.5 -1

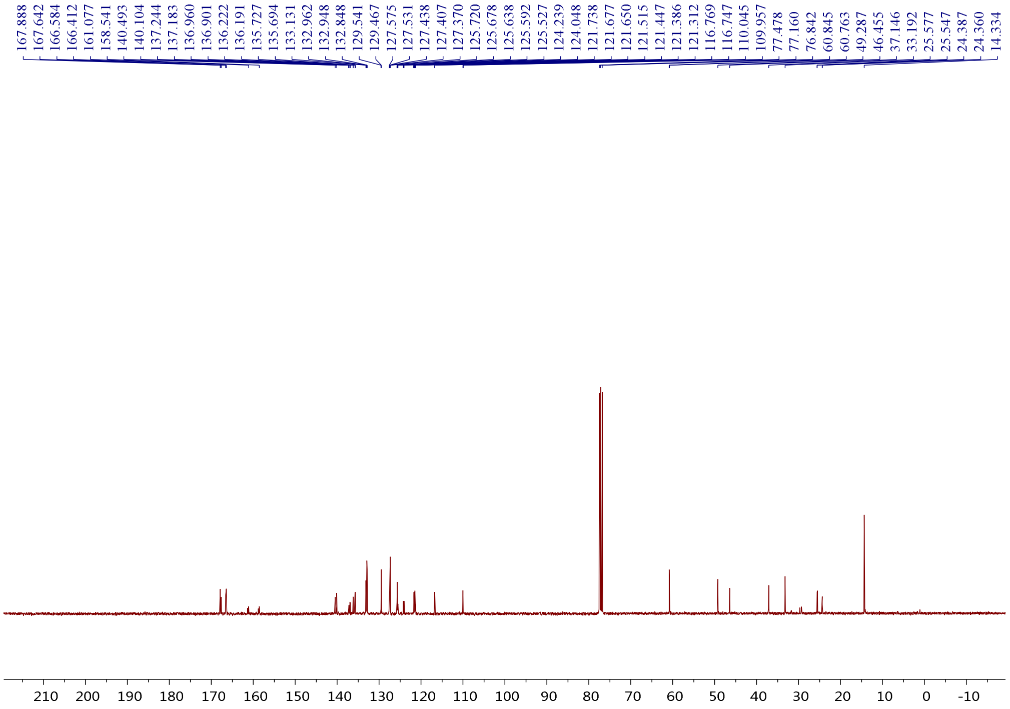
167.888
167.642
166.584
166.412
161.077
158.541
140.493
140.104
137.244
137.183
136.960
136.901
136.222
136.191
135.727
135.694
133.131
132.962
132.948
132.848
129.541
129.467
127.575
127.531
127.438
127.407
127.370
125.720
125.678
125.638
125.592
125.527
124.239
124.048
121.738
121.677
121.650
121.515
121.447
121.386
121.312
116.769
116.747
110.045
109.957
77.478
77.160
76.842
60.845
60.763
49.287
46.455
37.146
33.192
25.577
25.547
24.387
24.360
14.334
210 200 190 180 170 160 150 140 130 120 110 100 90 80 70 60 50 40 30 20 10 0 -10

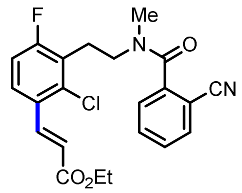

4n$_{\mathbf{mono}\ (m')}$

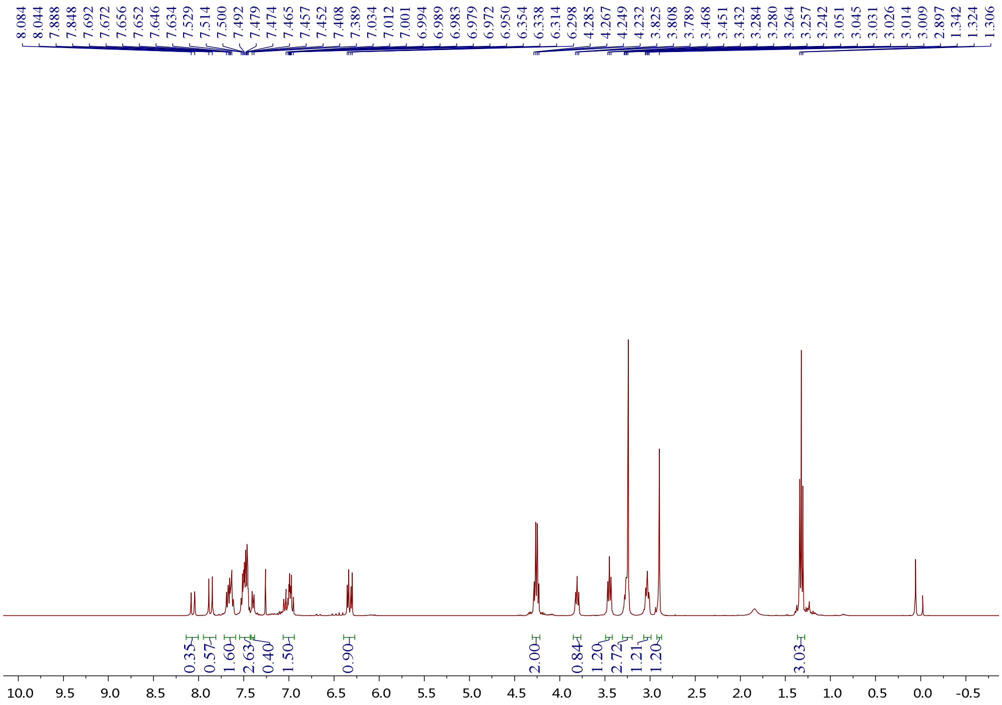

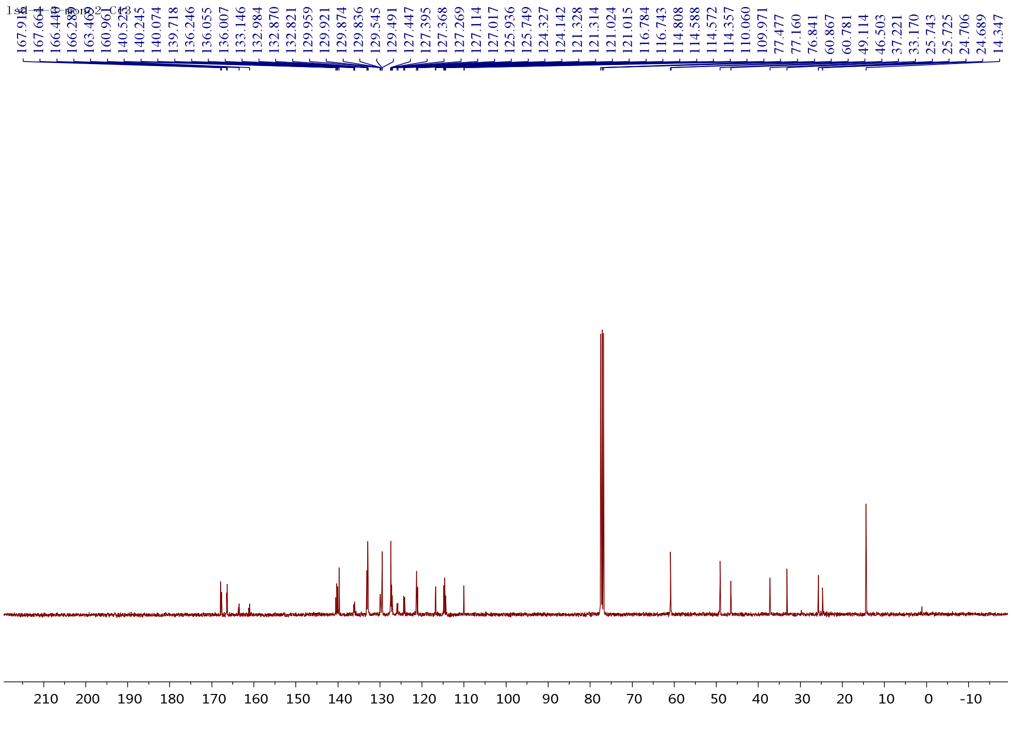

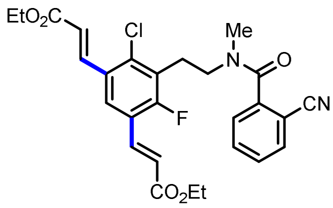
EtO2C
Cl
Me
N
O
CN
F
CO2Et

4n$_{(m,m')di}$

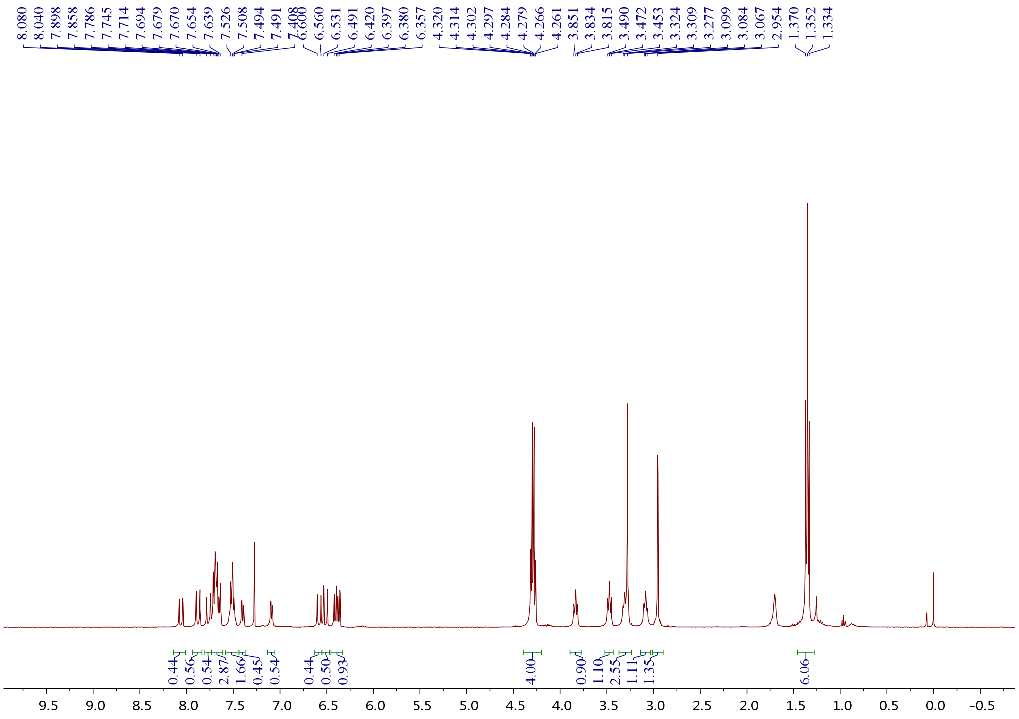
8.080 8.040 7.898 7.858 7.786 7.745 7.714 7.694 7.679 7.670 7.654 7.639 7.526 7.508 7.494 7.491 7.408 6.600 6.560 6.531 6.491 6.420 6.397 6.380 6.357 4.320 4.314 4.302 4.297 4.284 4.279 4.266 4.261 3.851 3.834 3.815 3.490 3.472 3.453 3.324 3.309 3.277 3.099 3.084 3.067 2.954 1.370 1.352 1.334
0.44 0.56 0.54 2.87 1.66 0.45 0.54 0.44 0.50 0.93 4.00 0.90 1.10 2.55 1.11 1.35 6.06
9.5 9.0 8.5 8.0 7.5 7.0 6.5 6.0 5.5 5.0 4.5 4.0 3.5 3.0 2.5 2.0 1.5 1.0 0.5 0.0 -0.5

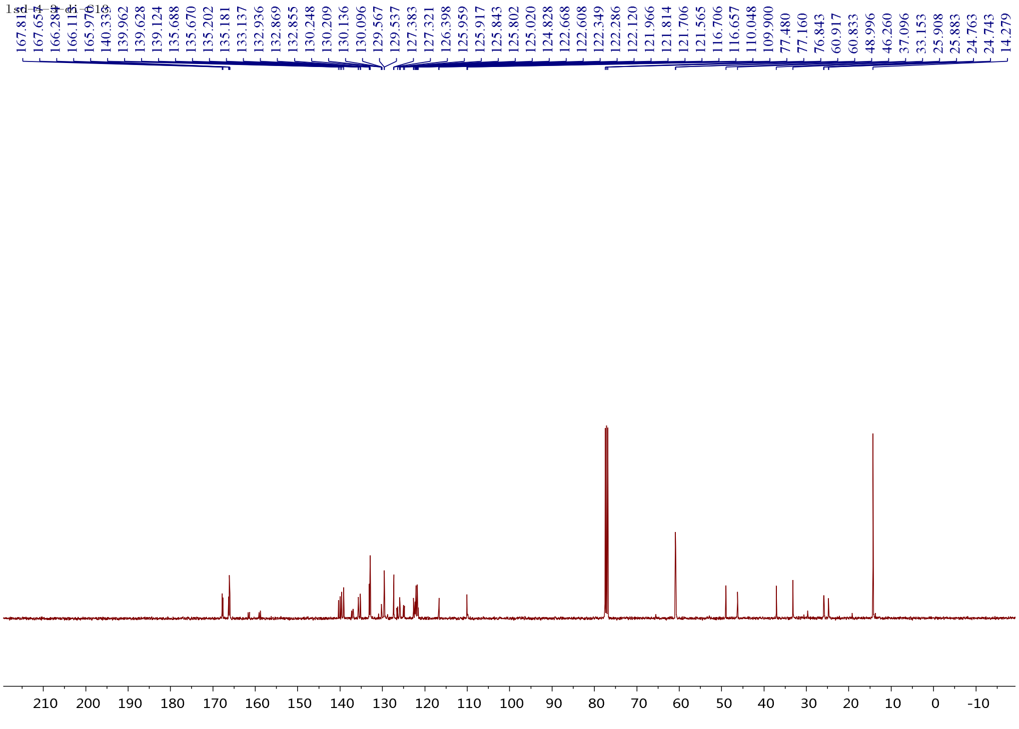
139.962 139.628 139.124 135.688 135.670 135.202 135.181 133.137 132.936 132.869 132.855 130.248 130.209 130.136 130.096 129.567 129.537 127.383 127.321 126.398 125.959 125.917 125.843 125.802 125.020 124.828 122.668 122.608 122.349 122.286 122.120 121.966 121.814 121.706 121.565 116.706 116.657 110.048 109.900 77.480 77.160 76.843 60.917 60.833 48.996 46.260 37.096 33.153 25.908 25.883 24.763 24.743 14.279
210 200 190 180 170 160 150 140 130 120 110 100 90 80 70 60 50 40 30 20 10 0 -10

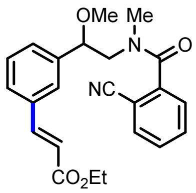
OMe
Me
N
O
NC
CO2Et

4o$_{mono}$

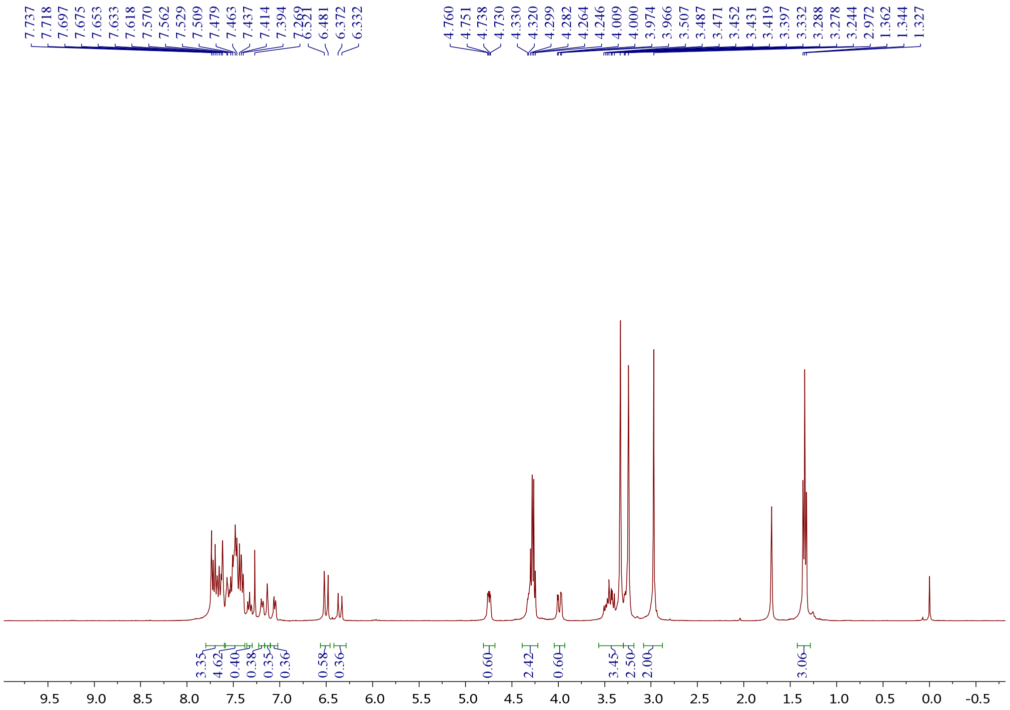
7.737
7.718
7.697
7.675
7.653
7.633
7.618
7.570
7.562
7.529
7.509
7.479
7.463
7.437
7.414
7.394
7.260
6.521
6.481
6.372
6.332
4.760
4.751
4.738
4.730
4.330
4.320
4.299
4.282
4.264
4.246
4.009
4.000
3.974
3.966
3.507
3.487
3.471
3.452
3.431
3.419
3.397
3.332
3.288
3.278
3.244
2.972
1.362
1.344
1.327
3.35
4.62
0.40
0.38
0.35
0.36
0.58
0.36
0.60
2.42
0.60
3.45
2.50
2.00
3.06
9.5 9.0 8.5 8.0 7.5 7.0 6.5 6.0 5.5 5.0 4.5 4.0 3.5 3.0 2.5 2.0 1.5 1.0 0.5 0.0 -0.5

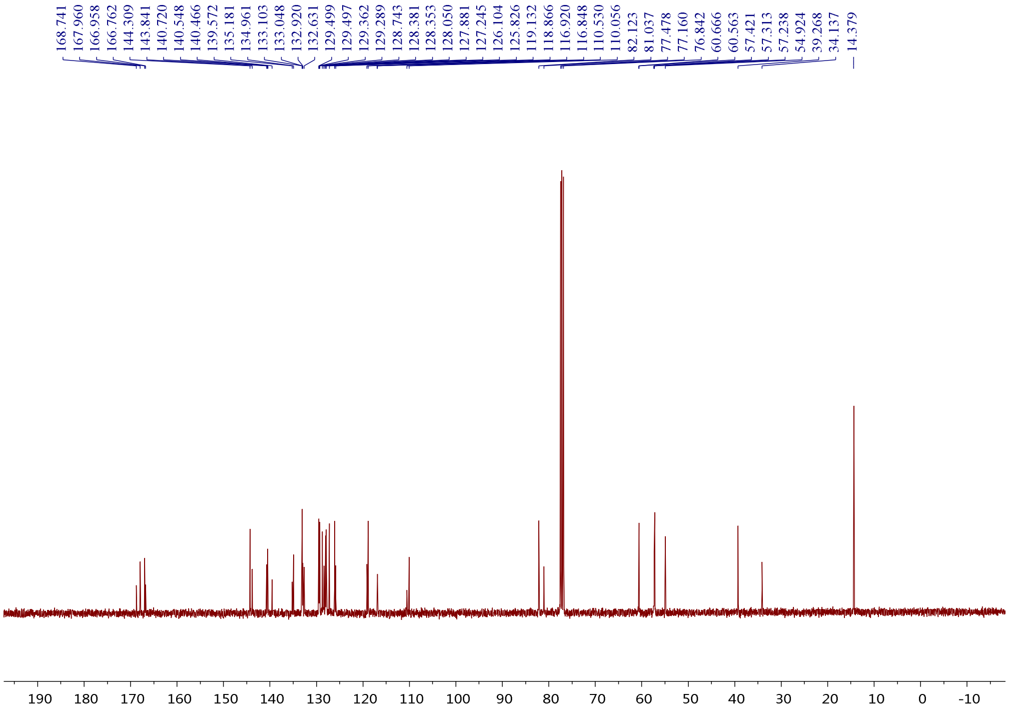
168.741
167.960
166.958
166.762
144.309
143.841
140.720
140.548
140.466
139.572
135.181
134.961
133.103
133.048
132.920
132.631
129.499
129.497
129.362
129.289
128.743
128.381
128.353
128.050
127.881
127.245
126.104
125.826
119.132
118.866
116.920
116.848
110.530
110.056
82.123
81.037
77.478
77.160
76.842
60.666
60.563
57.421
57.313
57.238
54.924
39.268
34.137
14.379
190 180 170 160 150 140 130 120 110 100 90 80 70 60 50 40 30 20 10 0 -10

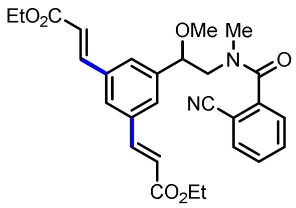
EtO2C
OMe
Me
N
O
NC
CO2Et

4o$_{(m,m')di}$

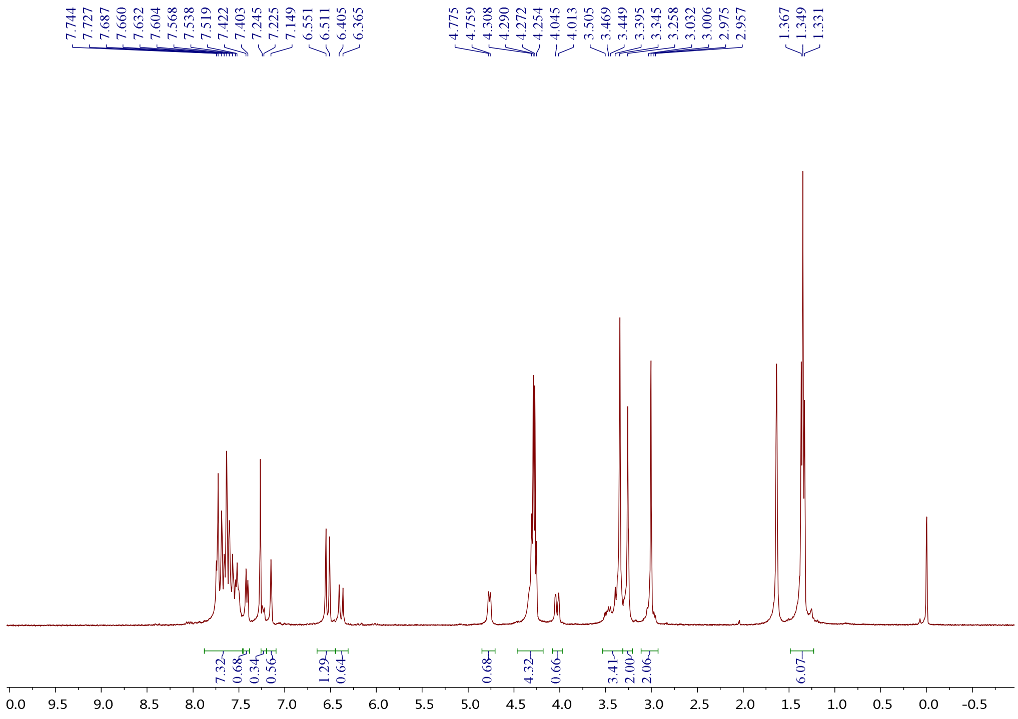
7.744
7.727
7.687
7.660
7.632
7.604
7.568
7.538
7.519
7.422
7.403
7.245
7.225
7.149
6.551
6.511
6.405
6.365
4.775
4.759
4.308
4.290
4.272
4.254
4.045
4.013
3.505
3.469
3.449
3.395
3.345
3.258
3.032
3.006
2.975
2.957
1.367
1.349
1.331
7.32
0.68
0.34
0.56
1.29
0.64
0.68
4.32
0.66
3.41
2.00
2.06
6.07
0.0 9.5 9.0 8.5 8.0 7.5 7.0 6.5 6.0 5.5 5.0 4.5 4.0 3.5 3.0 2.5 2.0 1.5 1.0 0.5 0.0 -0.5

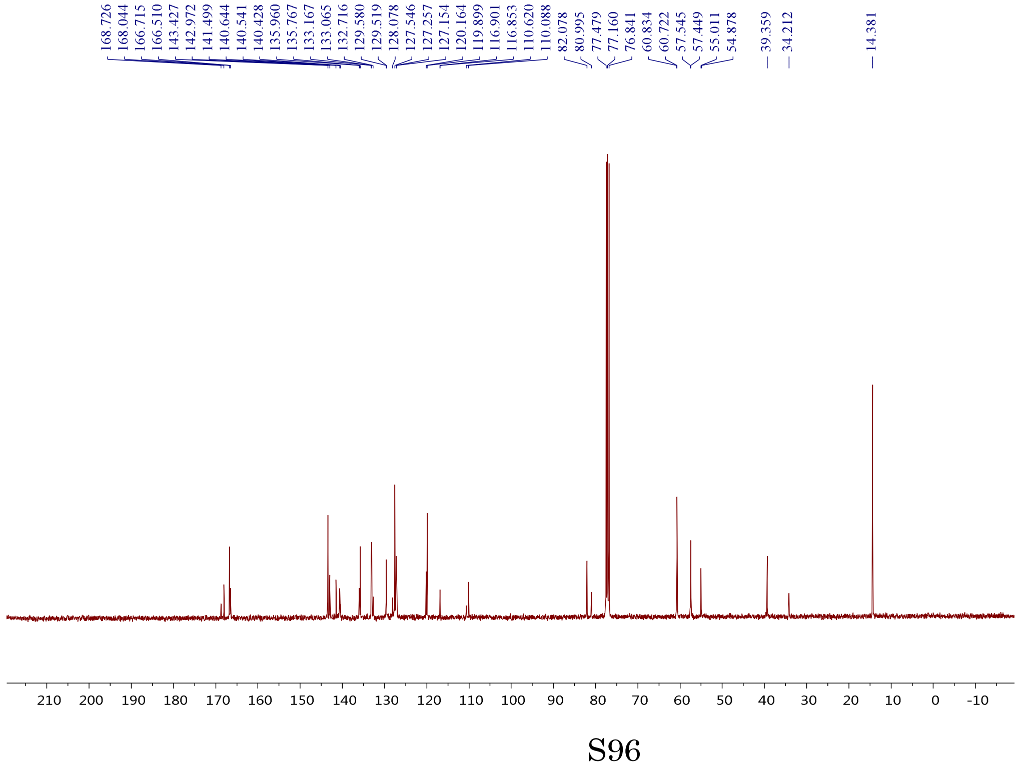
168.726
168.044
166.715
166.510
143.427
142.972
141.499
140.644
140.541
140.428
135.960
135.767
133.167
133.065
132.716
129.580
129.519
128.078
127.546
127.257
127.154
120.164
119.899
116.901
116.853
110.620
110.088
82.078
80.995
77.479
77.160
76.841
60.834
60.722
57.545
57.449
55.011
54.878
39.359
34.212
14.381
210 200 190 180 170 160 150 140 130 120 110 100 90 80 70 60 50 40 30 20 10 0 -10

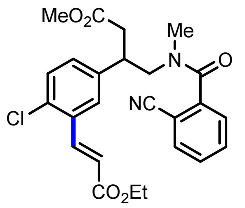
MeO2C
Me
N
O
NC
Cl
CO2Et

4p$_{mono}$

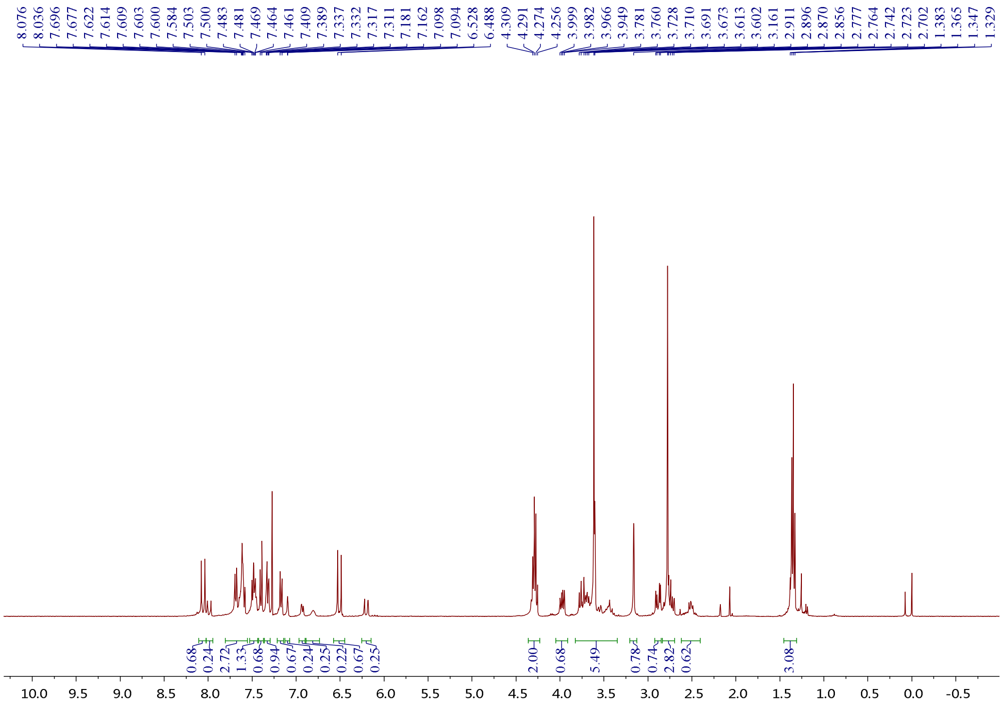
8.076
8.036
7.696
7.677
7.622
7.614
7.609
7.603
7.600
7.584
7.503
7.500
7.483
7.481
7.469
7.464
7.461
7.409
7.389
7.337
7.332
7.317
7.311
7.181
7.162
7.098
7.094
6.528
6.488
4.309
4.291
4.274
4.256
3.999
3.982
3.966
3.949
3.781
3.760
3.728
3.710
3.691
3.673
3.613
3.602
3.161
2.911
2.896
2.870
2.856
2.777
2.764
2.742
2.723
2.702
1.383
1.365
1.347
1.329
0.68
0.24
2.72
1.33
0.68
0.94
0.67
0.24
0.25
0.22
0.67
0.25
2.00
0.68
5.49
0.78
0.74
2.82
0.62
3.08
10.0 9.5 9.0 8.5 8.0 7.5 7.0 6.5 6.0 5.5 5.0 4.5 4.0 3.5 3.0 2.5 2.0 1.5 1.0 0.5 0.0 -0.5

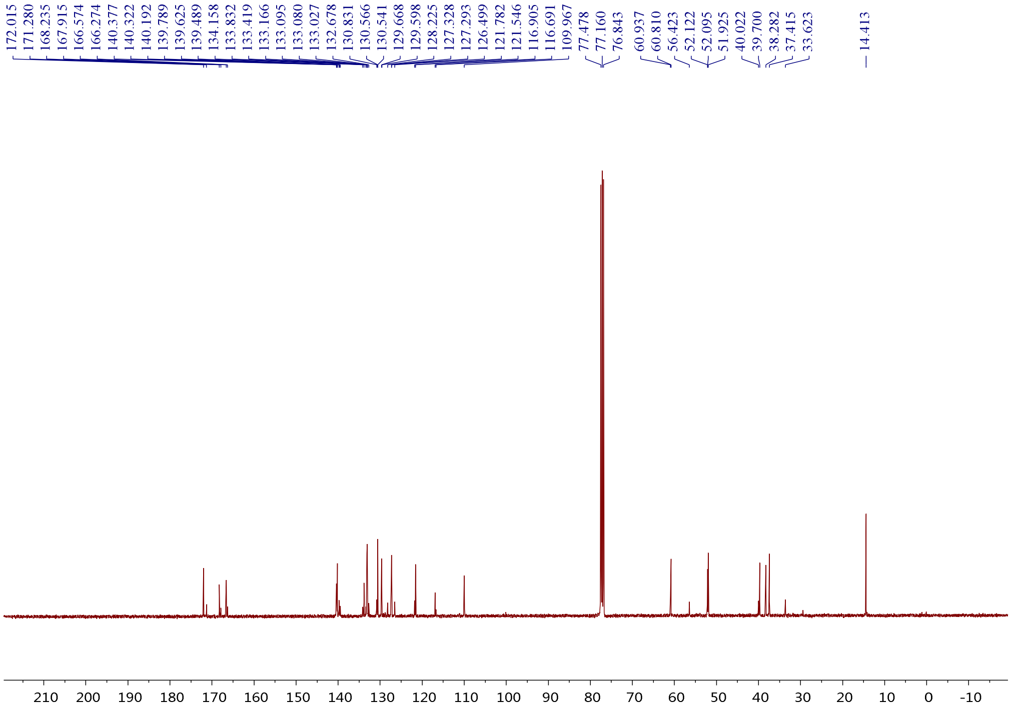
172.015
171.280
168.235
167.915
166.574
166.274
140.377
140.322
140.192
139.789
139.625
139.489
134.158
133.832
133.419
133.166
133.095
133.080
133.027
132.678
130.831
130.566
130.541
129.668
129.598
128.225
127.328
127.293
126.499
121.782
121.546
116.905
116.691
109.967
77.478
77.160
76.843
60.937
60.810
56.423
52.122
52.095
51.925
40.022
39.700
38.282
37.415
33.623
14.413
210 200 190 180 170 160 150 140 130 120 110 100 90 80 70 60 50 40 30 20 10 0 -10

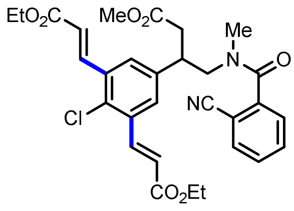
EtO2C
MeO2C
Me
N
O
NC
Cl
CO2Et

4p$_{(m,m')di}$

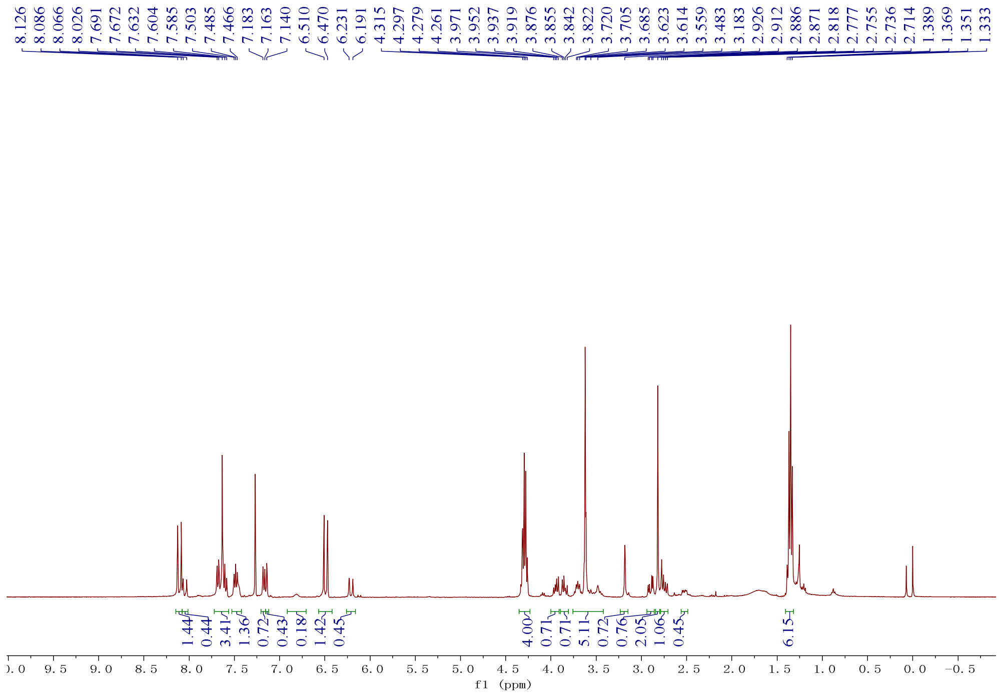
8.126 8.086 8.066 8.026 7.691 7.672 7.632 7.604 7.585 7.503 7.485 7.466 7.183 7.163 7.140 6.510 6.470 6.231 6.191 4.315 4.297 4.279 4.261 3.971 3.952 3.937 3.919 3.876 3.855 3.842 3.822 3.720 3.705 3.685 3.623 3.614 3.559 3.483 3.183 2.926 2.912 2.886 2.871 2.818 2.777 2.755 2.736 2.714 1.389 1.369 1.351 1.333
1.44 0.44 3.41 1.36 0.72 0.43 0.18 1.42 0.45 4.00 0.71 0.71 5.11 0.72 0.76 2.05 1.06 0.45 6.15
9.5 9.0 8.5 8.0 7.5 7.0 6.5 6.0 5.5 5.0 4.5 4.0 3.5 3.0 2.5 2.0 1.5 1.0 0.5 0.0 -0.5
f1 (ppm)

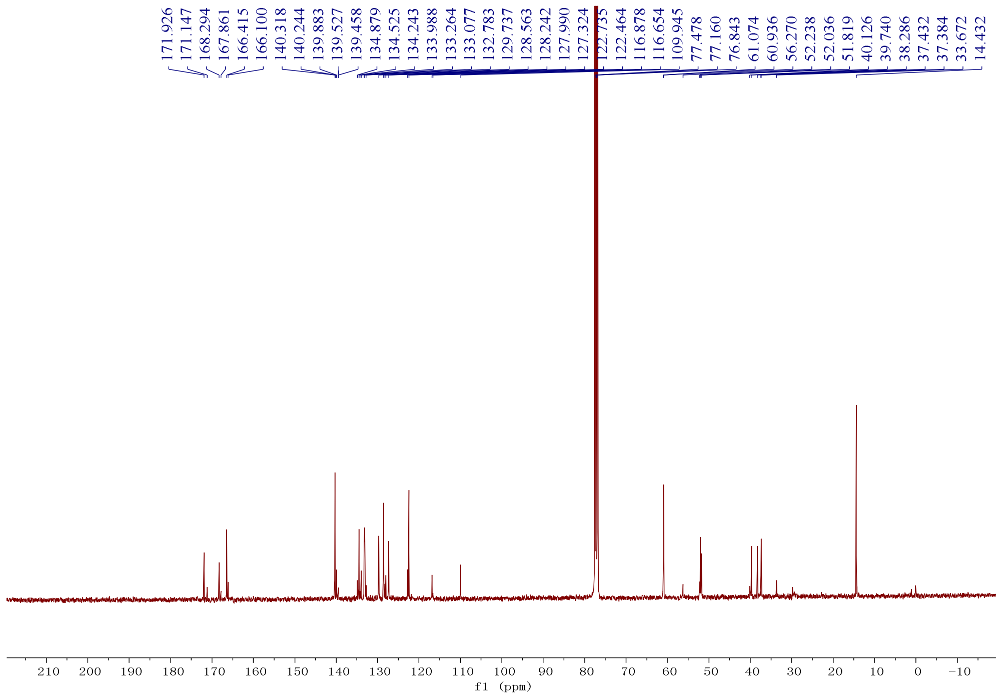
171.926 171.147 168.294 167.861 166.415 166.100 140.318 140.244 139.883 139.527 139.458 134.879 134.525 134.243 133.988 133.264 133.077 132.783 129.737 128.563 128.242 127.990 127.324 122.735 122.464 116.878 116.654 109.945 77.478 77.160 76.843 61.074 60.936 56.270 52.238 52.036 51.819 40.126 39.740 38.286 37.432 37.384 33.672 14.432
210 200 190 180 170 160 150 140 130 120 110 100 90 80 70 60 50 40 30 20 10 0 -10
f1 (ppm)

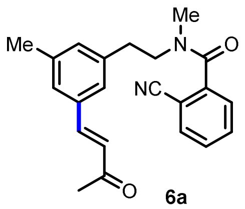
Me
Me
N
O
NC
O

6a

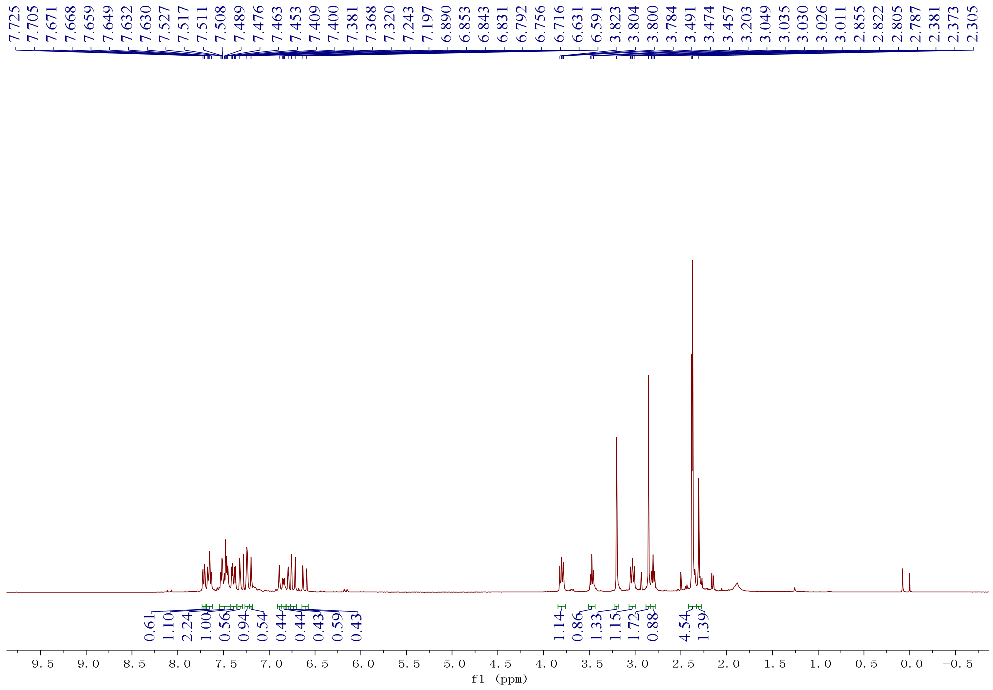
7.725
7.705
7.671
7.668
7.659
7.649
7.632
7.630
7.527
7.517
7.511
7.508
7.489
7.476
7.463
7.453
7.409
7.400
7.381
7.368
7.320
7.243
7.197
6.890
6.853
6.843
6.831
6.792
6.756
6.716
6.631
6.591
3.823
3.804
3.800
3.784
3.491
3.474
3.457
3.203
3.049
3.035
3.030
3.026
3.011
2.855
2.822
2.805
2.787
2.381
2.373
2.305
0.61
1.10
2.24
1.00
0.56
0.94
0.54
0.44
0.44
0.43
0.59
0.43
1.14
0.86
1.33
1.15
1.72
0.88
4.54
1.39
9.5 9.0 8.5 8.0 7.5 7.0 6.5 6.0 5.5 5.0 4.5 4.0 3.5 3.0 2.5 2.0 1.5 1.0 0.5 0.0 -0.5
f1 (ppm)

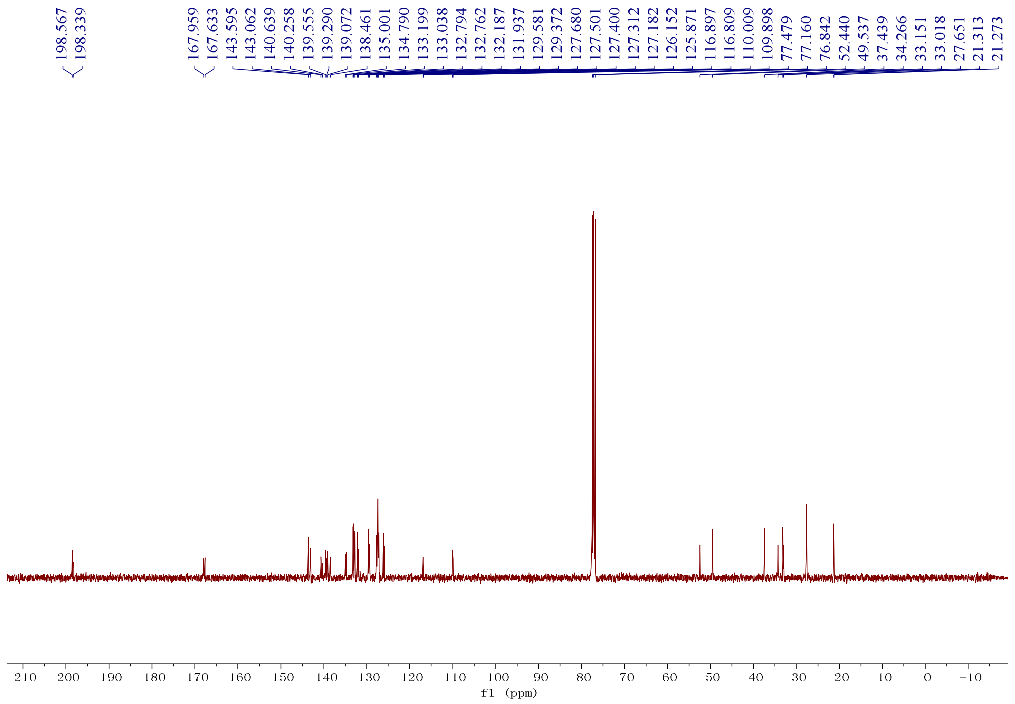
198.567
198.339
167.959
167.633
143.595
143.062
140.639
140.258
139.555
139.290
139.072
138.461
135.001
134.790
133.199
133.038
132.794
132.762
132.187
131.937
129.581
129.372
127.680
127.501
127.400
127.312
127.182
126.152
125.871
116.897
116.809
110.009
109.898
77.479
77.160
76.842
52.440
49.537
37.439
34.266
33.151
33.018
27.651
21.313
21.273
210 200 190 180 170 160 150 140 130 120 110 100 90 80 70 60 50 40 30 20 10 0 -10
f1 (ppm)

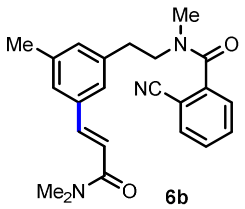
Me
Me
N
O
NC
Me2N
O

6b

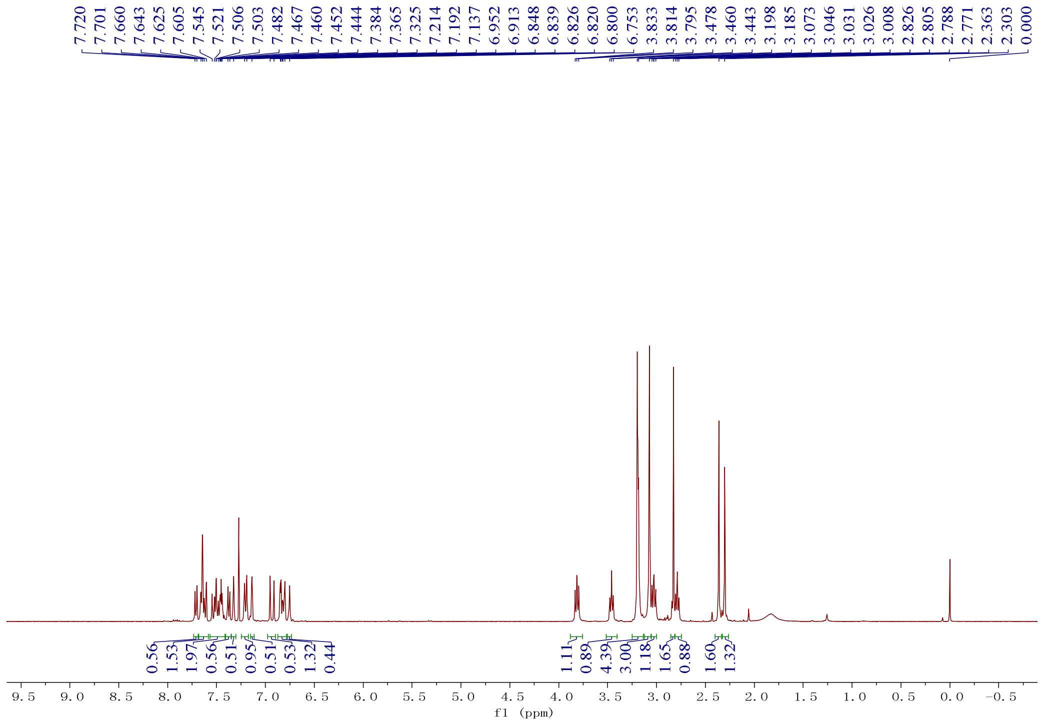
7.720 7.701 7.660 7.643 7.625 7.605 7.545 7.521 7.506 7.503 7.482 7.467 7.460 7.452 7.444 7.384 7.365 7.325 7.214 7.192 7.137 6.952 6.913 6.848 6.839 6.826 6.820 6.800 6.753 3.833 3.814 3.795 3.478 3.460 3.443 3.198 3.185 3.073 3.046 3.031 3.026 3.008 2.826 2.805 2.788 2.771 2.363 2.303 0.000
0.56 1.53 1.97 0.56 0.51 0.95 0.51 0.53 1.32 0.44
1.11 0.89 4.39 3.00 1.18 1.65 0.88 1.60 1.32
9.5 9.0 8.5 8.0 7.5 7.0 6.5 6.0 5.5 5.0 4.5 4.0 3.5 3.0 2.5 2.0 1.5 1.0 0.5 0.0 −0.5
f1 (ppm)

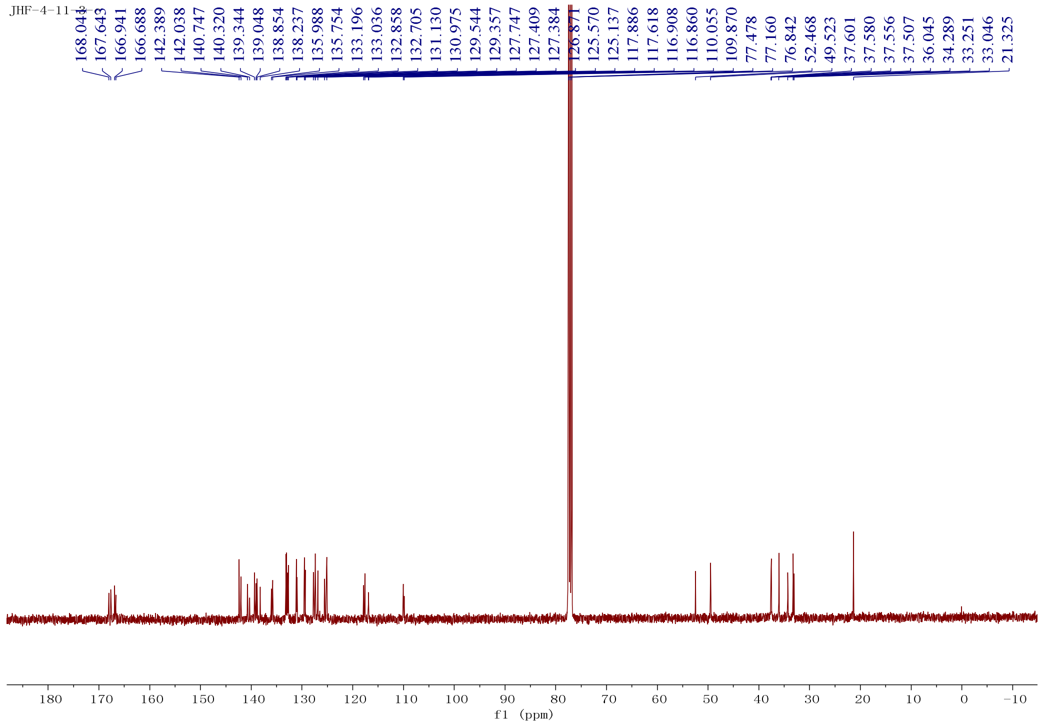
JHF-4-11-[illegible]
168.044 167.643 166.941 166.688 142.389 142.038 140.747 140.320 139.344 139.048 138.854 138.237 135.988 135.754 133.196 133.036 132.858 132.705 131.130 130.975 129.544 129.357 127.747 127.409 127.384 126.871 125.570 125.137 117.886 117.618 116.908 116.860 110.055 109.870 77.478 77.160 76.842 52.468 49.523 37.601 37.580 37.556 37.507 36.045 34.289 33.251 33.046 21.325
180 170 160 150 140 130 120 110 100 90 80 70 60 50 40 30 20 10 0 −10
f1 (ppm)

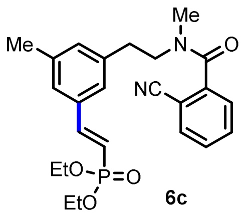
Me
Me
N
O
NC
EtO
P=O
EtO

6c

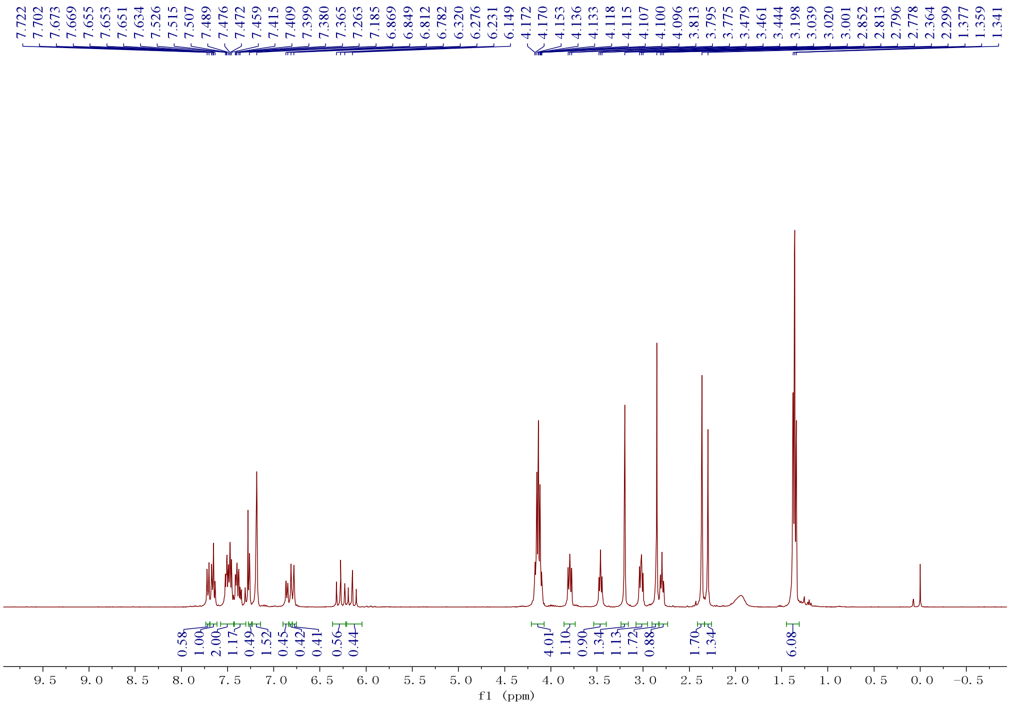
7.722
7.702
7.673
7.669
7.655
7.653
7.651
7.634
7.526
7.515
7.507
7.489
7.476
7.472
7.459
7.415
7.409
7.399
7.380
7.365
7.263
7.185
6.869
6.849
6.812
6.782
6.320
6.276
6.231
6.149
4.172
4.170
4.153
4.136
4.133
4.118
4.115
4.107
4.100
4.096
3.813
3.795
3.775
3.479
3.461
3.444
3.198
3.039
3.020
3.001
2.852
2.813
2.796
2.778
2.364
2.299
1.377
1.359
1.341
0.58
1.00
2.00
1.17
0.49
1.52
0.45
0.42
0.41
0.56
0.44
4.01
1.10
0.90
1.34
1.13
1.72
0.88
1.70
1.34
6.08
f1 (ppm)

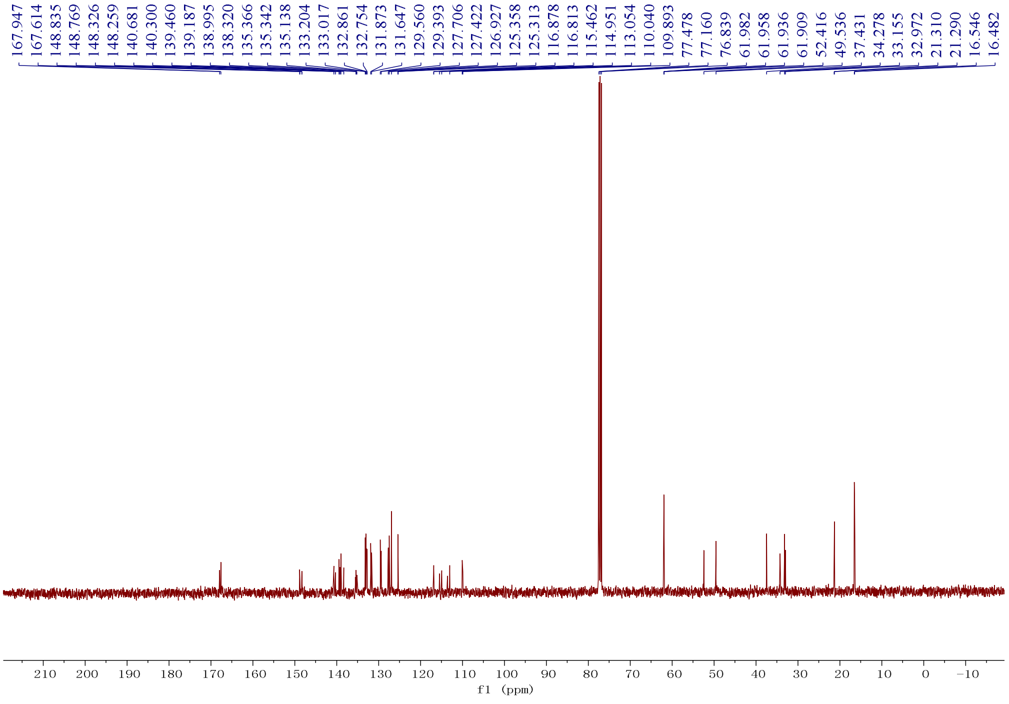
167.947
167.614
148.835
148.769
148.326
148.259
140.681
140.300
139.460
139.187
138.995
138.320
135.366
135.342
135.138
133.204
133.017
132.861
132.754
131.873
131.647
129.560
129.393
127.706
127.422
126.927
125.358
125.313
116.878
116.813
115.462
114.951
113.054
110.040
109.893
77.478
77.160
76.839
61.982
61.958
61.936
61.909
52.416
49.536
37.431
34.278
33.155
32.972
21.310
21.290
16.546
16.482
f1 (ppm)

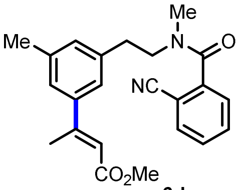
Me
Me
N
O
NC
CO_2Me

6d

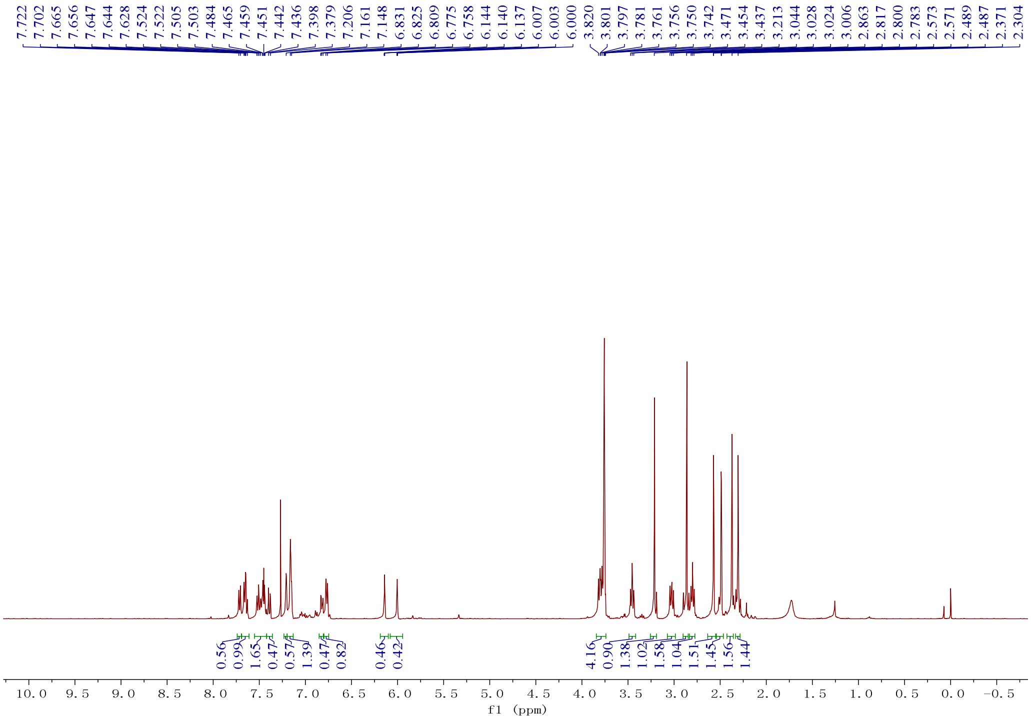
7.722
7.702
7.665
7.656
7.647
7.644
7.628
7.524
7.522
7.505
7.503
7.484
7.465
7.459
7.451
7.442
7.436
7.398
7.379
7.206
7.161
7.148
6.831
6.825
6.809
6.775
6.758
6.144
6.140
6.137
6.007
6.003
6.000
3.820
3.801
3.797
3.781
3.761
3.756
3.750
3.742
3.471
3.454
3.437
3.213
3.044
3.028
3.024
3.006
2.863
2.817
2.800
2.783
2.573
2.571
2.489
2.487
2.371
2.304
0.56
0.99
1.65
0.47
0.57
1.39
0.47
0.82
0.46
0.42
4.16
0.90
1.38
1.02
1.58
1.04
1.51
1.45
1.56
1.44
10.0 9.5 9.0 8.5 8.0 7.5 7.0 6.5 6.0 5.5 5.0 4.5 4.0 3.5 3.0 2.5 2.0 1.5 1.0 0.5 0.0 -0.5
f1 (ppm)

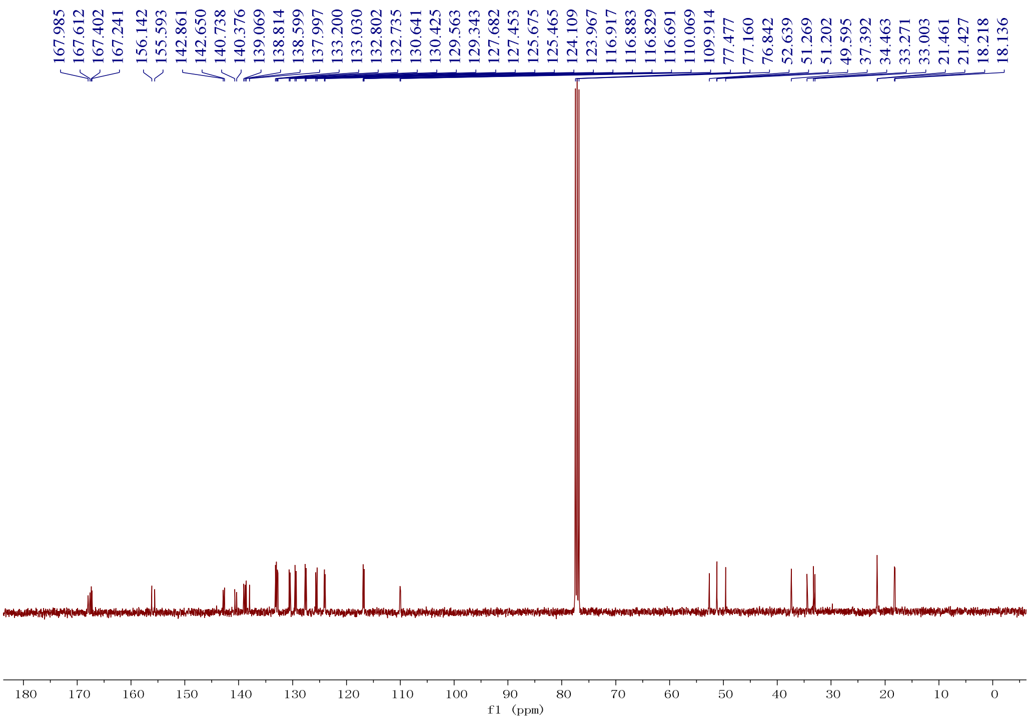
167.985
167.612
167.402
167.241
156.142
155.593
142.861
142.650
140.738
140.376
139.069
138.814
138.599
137.997
133.200
133.030
132.802
132.735
130.641
130.425
129.563
129.343
127.682
127.453
125.675
125.465
124.109
123.967
116.917
116.883
116.829
116.691
110.069
109.914
77.477
77.160
76.842
52.639
51.269
51.202
49.595
37.392
34.463
33.271
33.003
21.461
21.427
18.218
18.136
180 170 160 150 140 130 120 110 100 90 80 70 60 50 40 30 20 10 0
f1 (ppm)

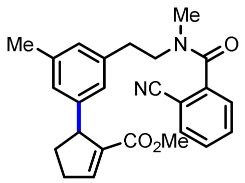

Me
Me
N
O
NC
CO2Me

6e

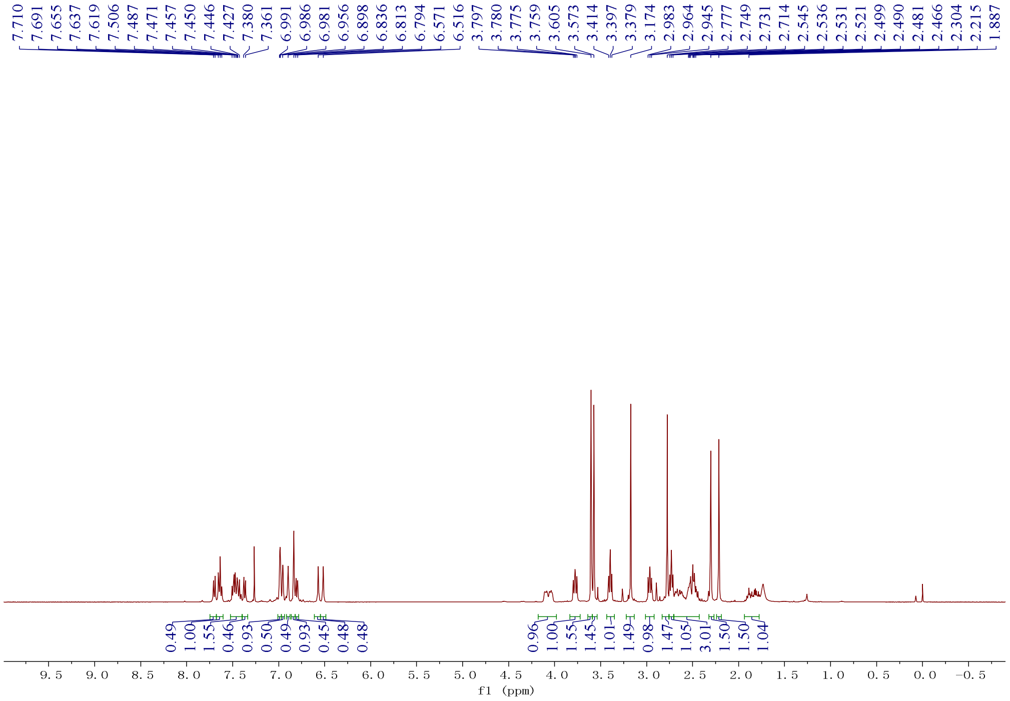

7.710
7.691
7.655
7.637
7.619
7.506
7.487
7.471
7.457
7.450
7.446
7.427
7.380
7.361
6.991
6.986
6.981
6.956
6.898
6.836
6.813
6.794
6.571
6.516
3.797
3.780
3.775
3.759
3.605
3.573
3.414
3.397
3.379
3.174
2.983
2.964
2.945
2.777
2.749
2.731
2.714
2.545
2.536
2.531
2.521
2.499
2.490
2.481
2.466
2.304
2.215
1.887
0.49
1.00
1.55
0.46
0.93
0.50
0.49
0.93
0.45
0.48
0.48
0.96
1.00
1.55
1.45
1.01
1.49
0.98
1.47
1.05
3.01
1.50
1.50
1.04
f1 (ppm)

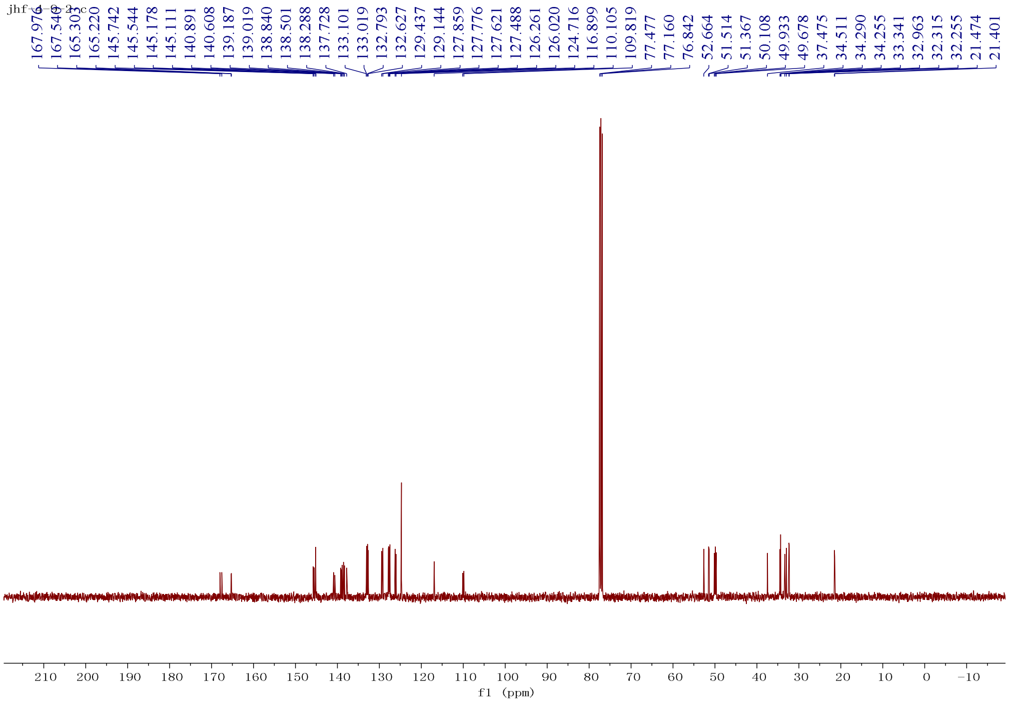

167.974
167.540
165.305
165.220
145.742
145.544
145.178
145.111
140.891
140.608
139.187
139.019
138.840
138.501
138.288
137.728
133.101
133.019
132.793
132.627
129.437
129.144
127.859
127.776
127.621
127.488
126.261
126.020
124.716
116.899
110.105
109.819
77.477
77.160
76.842
52.664
51.514
51.367
50.108
49.933
49.678
37.475
34.511
34.290
34.255
33.341
32.963
32.315
32.255
21.474
21.401
f1 (ppm)

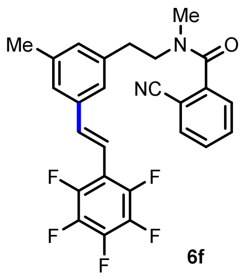
Me
Me
N
O
NC
F
F
F
F
F

6f

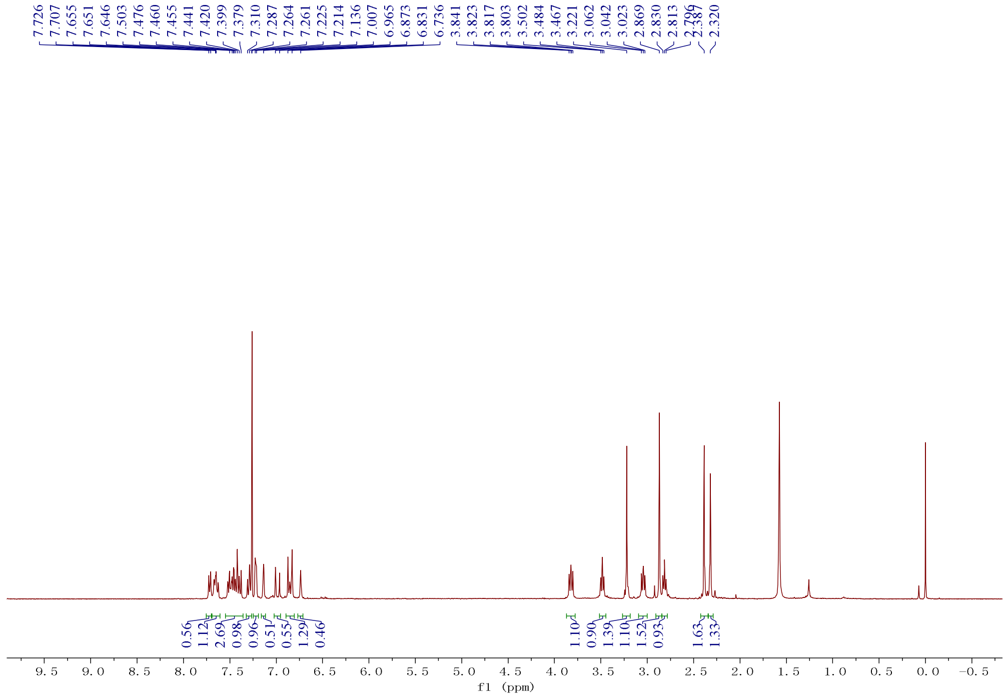
7.726
7.707
7.655
7.651
7.646
7.503
7.476
7.460
7.455
7.441
7.420
7.399
7.379
7.310
7.287
7.264
7.261
7.225
7.214
7.136
7.007
6.965
6.873
6.831
6.736
3.841
3.823
3.817
3.803
3.502
3.484
3.467
3.221
3.062
3.042
3.023
2.869
2.830
2.813
2.387
2.320
0.56
1.12
2.69
0.98
0.96
0.51
0.55
1.29
0.46
1.10
0.90
1.39
1.10
1.52
0.93
1.63
1.33
f1 (ppm)

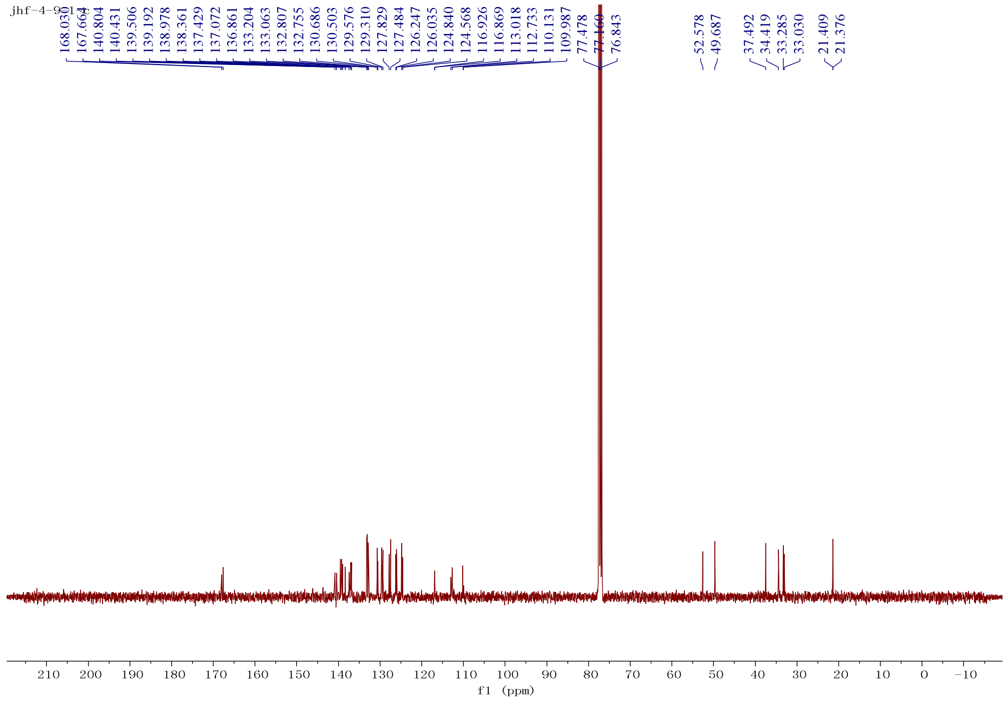
168.031
167.664
140.804
140.431
139.506
139.192
138.978
138.361
137.429
137.072
136.861
133.204
133.063
132.807
132.755
130.686
130.503
129.576
129.310
127.829
127.484
126.247
126.035
124.840
124.568
116.926
116.869
113.018
112.733
110.131
109.987
77.478
76.843
52.578
49.687
37.492
34.419
33.285
33.030
21.409
21.376
f1 (ppm)

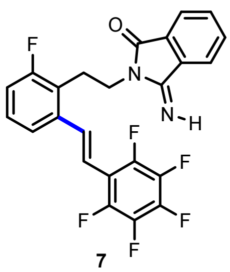
F
O
N
N
H
F
F
F
F
F

7

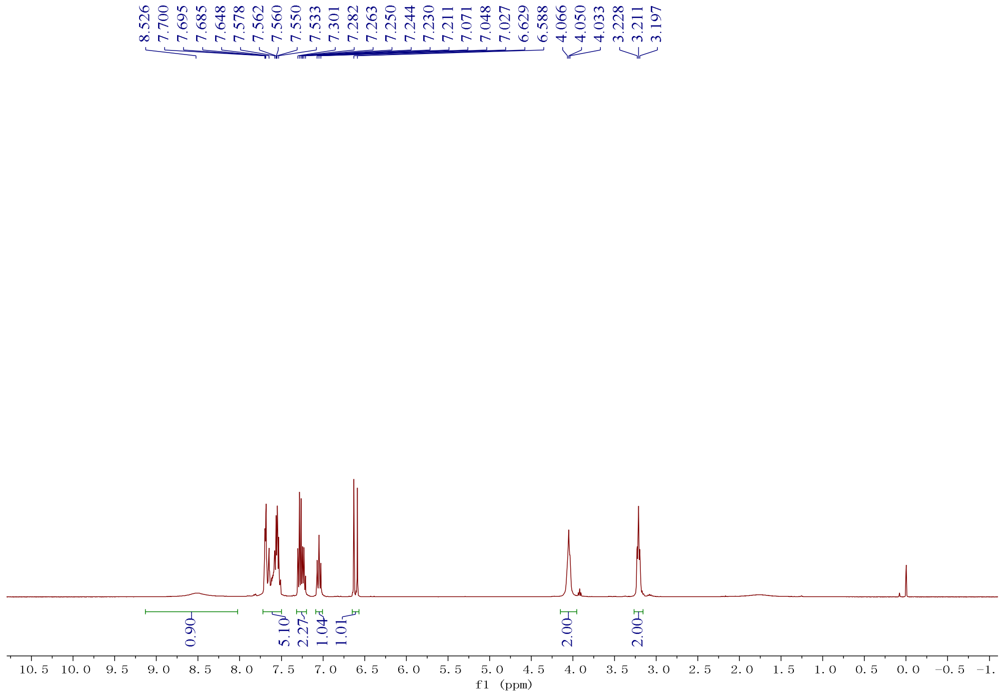
8.526
7.700
7.695
7.685
7.648
7.578
7.562
7.560
7.550
7.533
7.301
7.282
7.263
7.250
7.244
7.230
7.211
7.071
7.048
7.027
6.629
6.588
4.066
4.050
4.033
3.228
3.211
3.197
0.90
5.10
2.27
1.04
1.01
2.00
2.00
f1 (ppm)

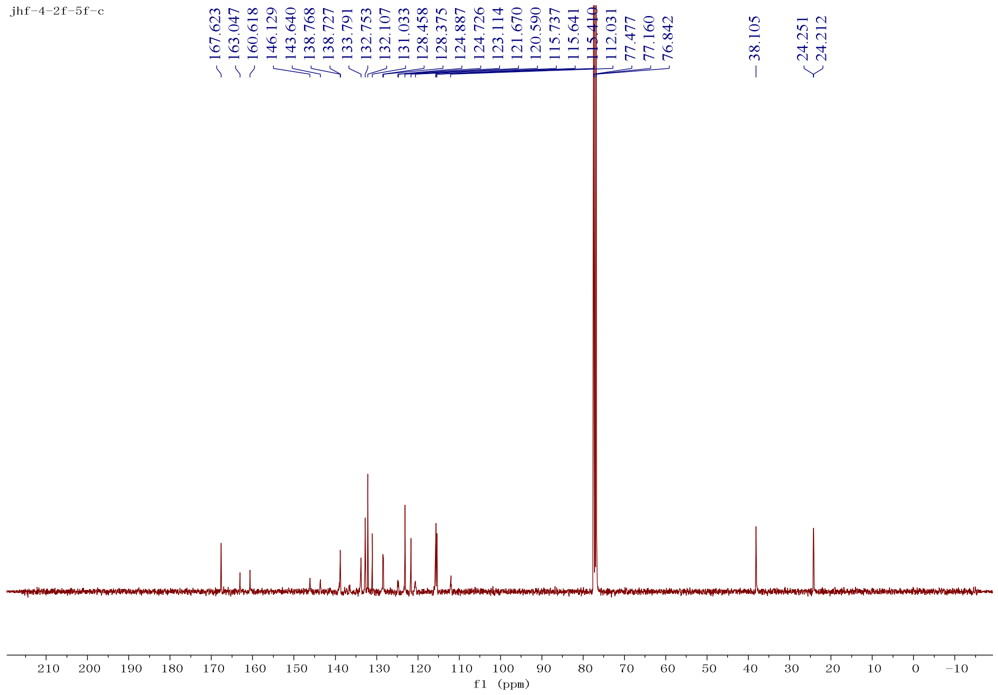
jhf-4-2f-5f-c
167.623
163.047
160.618
146.129
143.640
138.768
138.727
133.791
132.753
132.107
131.033
128.458
128.375
124.887
124.726
123.114
121.670
120.590
115.737
115.641
115.410
112.031
77.477
77.160
76.842
38.105
24.251
24.212
f1 (ppm)

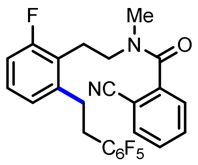
F
Me
N
O
NC
C6F5

8

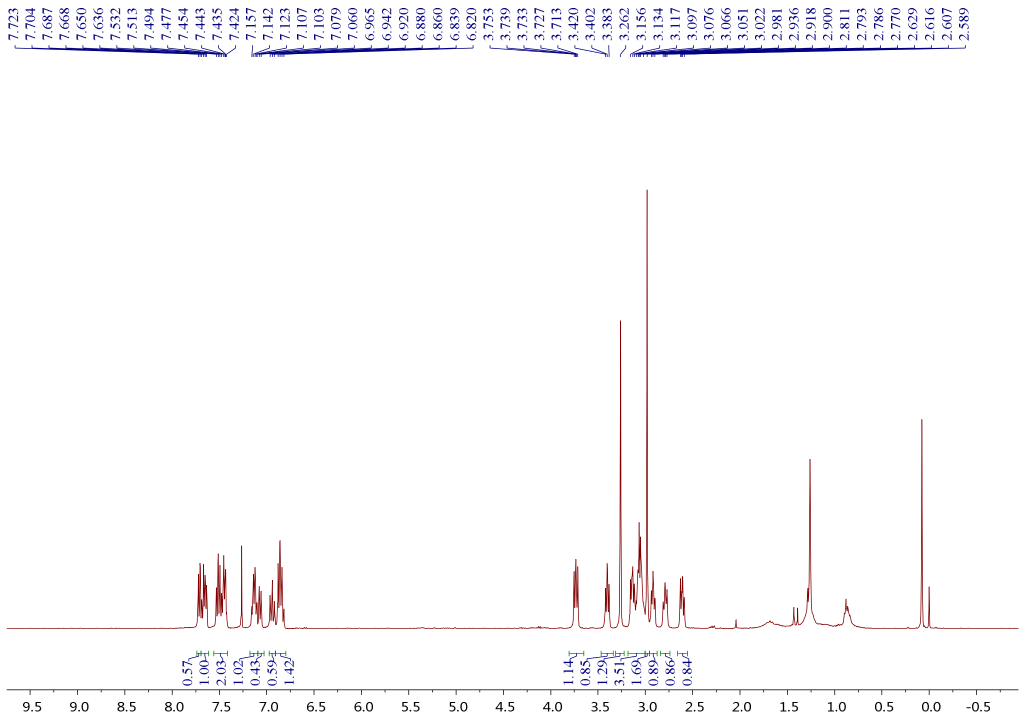
7.723
7.704
7.687
7.668
7.650
7.636
7.532
7.513
7.494
7.477
7.454
7.443
7.435
7.424
7.157
7.142
7.123
7.107
7.103
7.079
7.060
6.965
6.942
6.920
6.880
6.860
6.839
6.820
3.753
3.739
3.733
3.727
3.713
3.420
3.402
3.383
3.262
3.156
3.134
3.117
3.097
3.076
3.066
3.051
3.022
2.981
2.936
2.918
2.900
2.811
2.793
2.786
2.770
2.629
2.616
2.607
2.589
0.57
1.00
2.03
1.02
0.43
0.59
1.42
1.14
0.85
1.29
3.51
1.69
0.89
0.86
0.84
9.5
9.0
8.5
8.0
7.5
7.0
6.5
6.0
5.5
5.0
4.5
4.0
3.5
3.0
2.5
2.0
1.5
1.0
0.5
0.0
-0.5

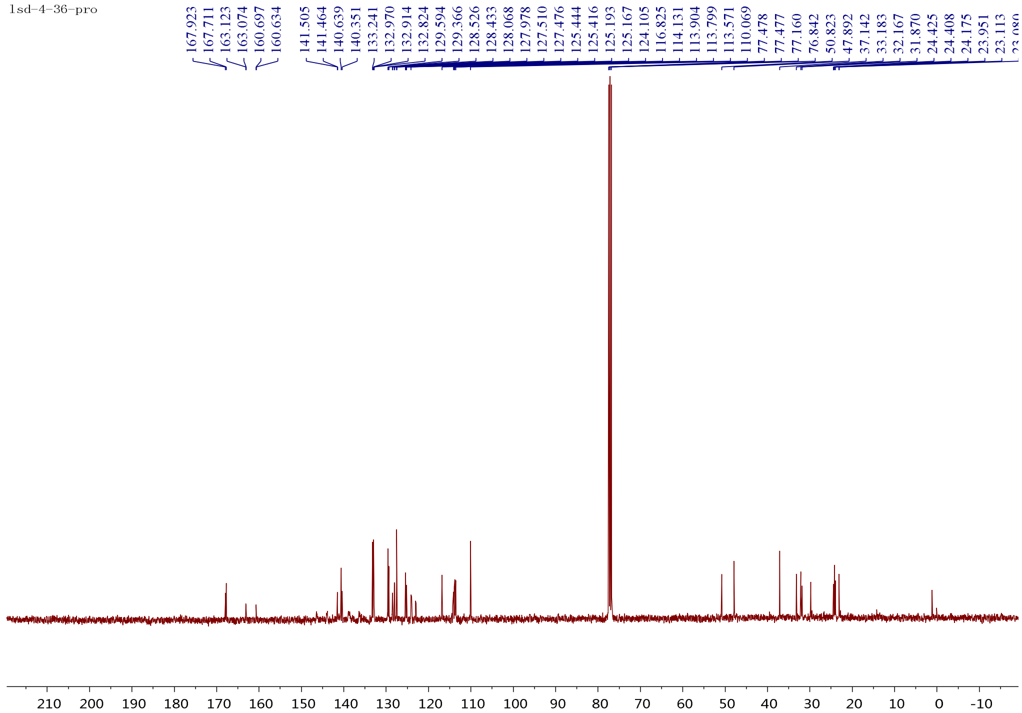
1sd-4-36-pro
167.923
167.711
163.123
163.074
160.697
160.634
141.505
141.464
140.639
140.351
133.241
132.970
132.914
132.824
129.366
129.594
128.526
128.433
128.068
127.978
127.510
127.476
125.444
125.416
125.193
125.167
124.105
116.825
114.131
113.904
113.799
113.571
110.069
77.478
77.477
77.160
76.842
50.823
47.892
37.142
33.183
32.167
31.870
24.425
24.408
24.175
23.951
23.113
210
200
190
180
170
160
150
140
130
120
110
100
90
80
70
60
50
40
30
20
10
0
-10

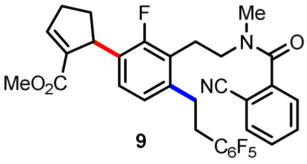
F
Me
N
O
MeO2C
NC
C6F5

9

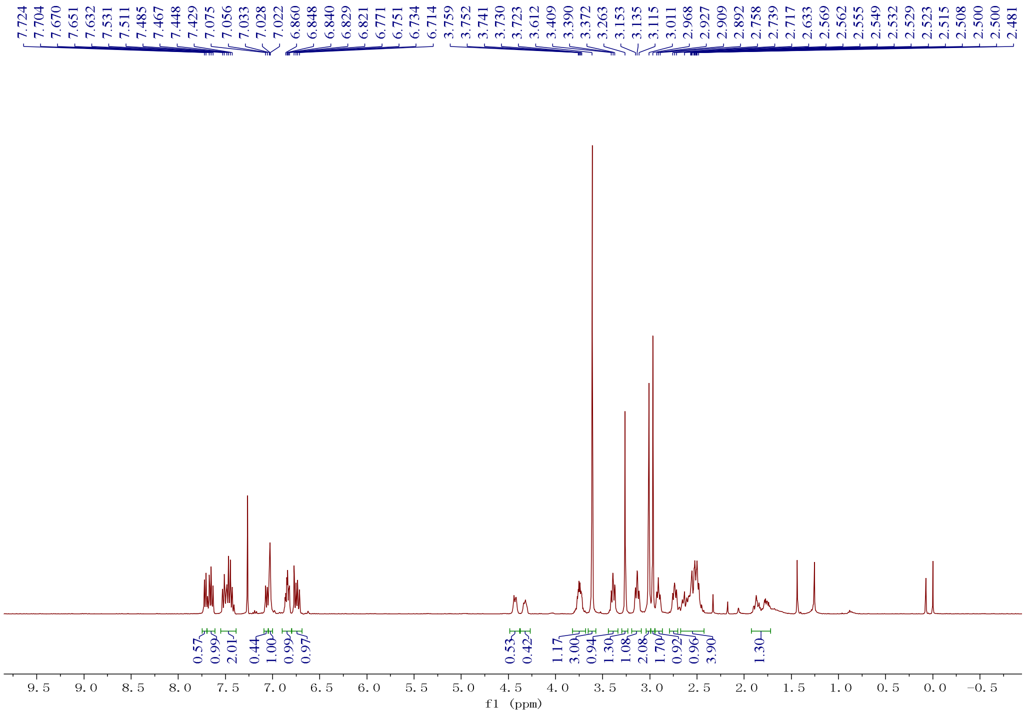
7.724 7.704 7.670 7.651 7.632 7.531 7.511 7.485 7.467 7.448 7.429 7.075 7.056 7.033 7.028 7.022 6.860 6.848 6.840 6.829 6.821 6.771 6.751 6.734 6.714 3.759 3.752 3.741 3.730 3.723 3.612 3.409 3.390 3.372 3.263 3.153 3.135 3.115 3.011 2.968 2.927 2.909 2.892 2.758 2.739 2.717 2.633 2.569 2.562 2.555 2.549 2.532 2.529 2.523 2.515 2.508 2.500 2.500 2.481
0.57 0.99 2.01 0.44 1.00 0.99 0.97 0.53 0.42 1.17 3.00 0.94 1.30 1.08 2.08 1.70 0.92 0.96 3.90 1.30
9.5 9.0 8.5 8.0 7.5 7.0 6.5 6.0 5.5 5.0 4.5 4.0 3.5 3.0 2.5 2.0 1.5 1.0 0.5 0.0 −0.5
f1 (ppm)

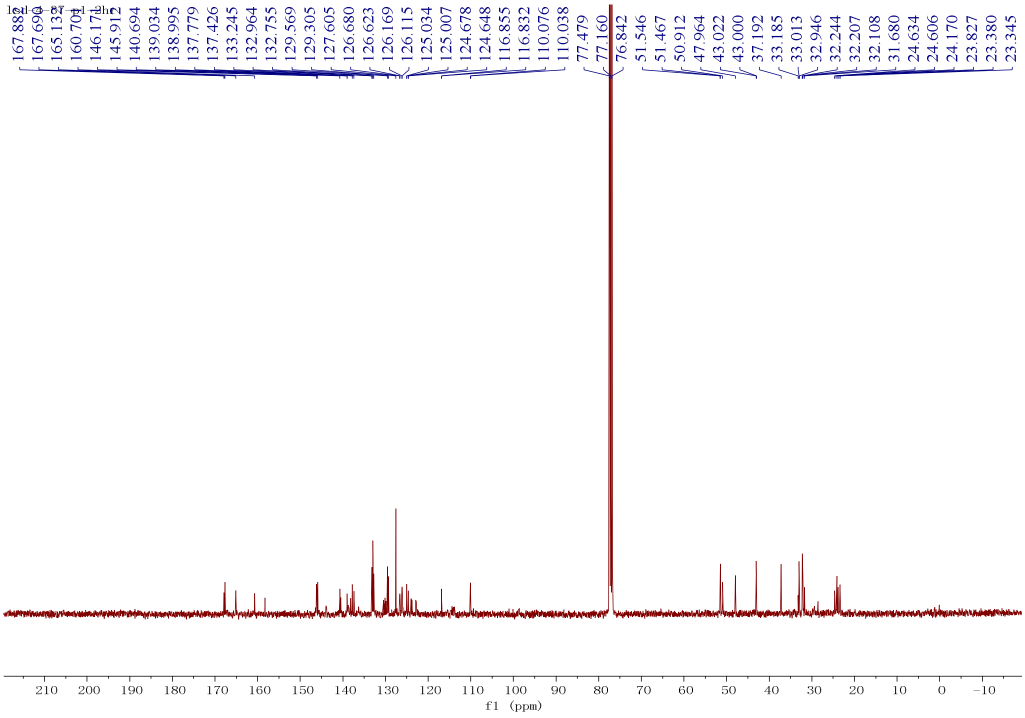
167.882 167.690 165.137 160.701 146.171 145.912 140.694 139.034 138.995 137.779 137.426 133.245 132.964 132.755 129.569 129.305 127.605 126.680 126.623 126.169 126.115 125.034 125.007 124.678 124.648 116.855 116.832 110.076 110.038 77.479 77.160 76.842 51.546 51.467 50.912 47.964 43.022 43.000 37.192 33.185 33.013 32.946 32.244 32.207 32.108 31.680 24.634 24.606 24.170 23.827 23.380 23.345
210 200 190 180 170 160 150 140 130 120 110 100 90 80 70 60 50 40 30 20 10 0 −10
f1 (ppm)

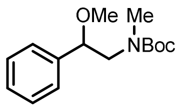

S4

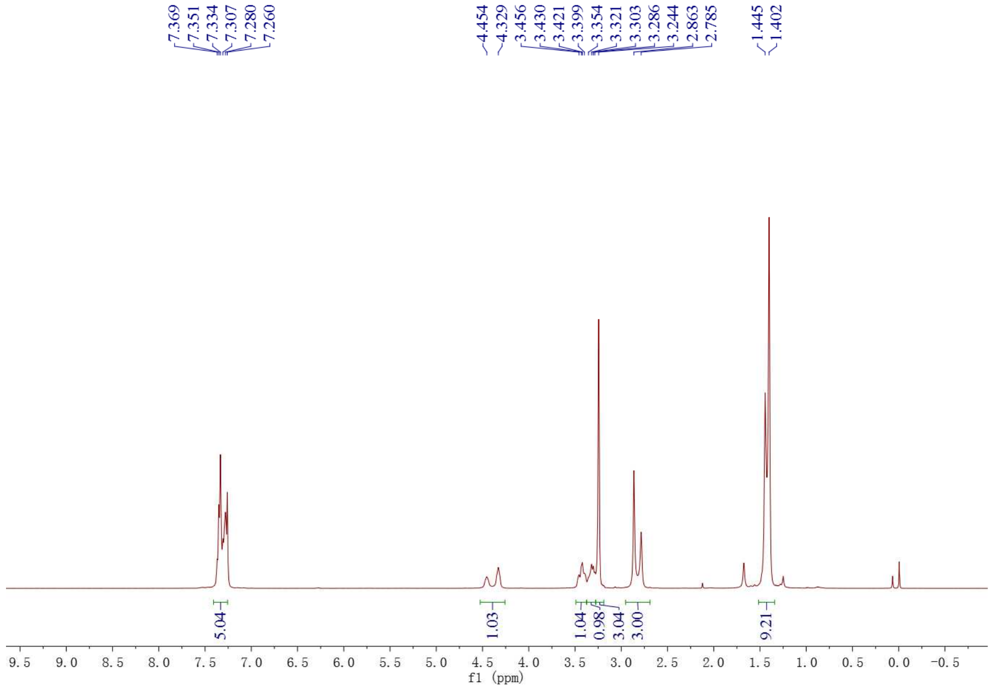

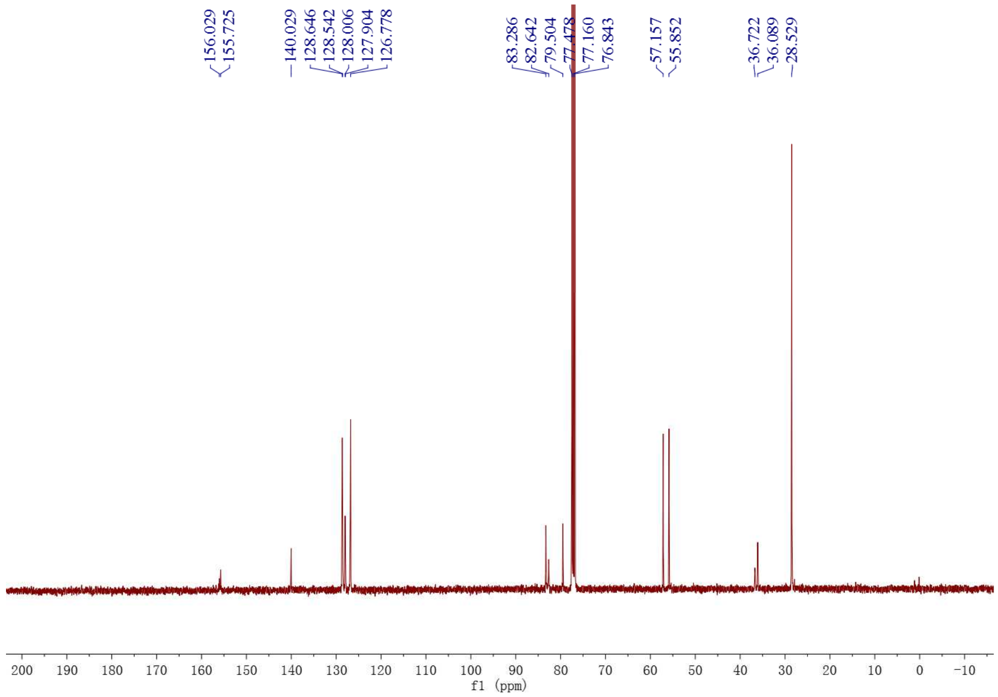

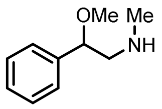

S5

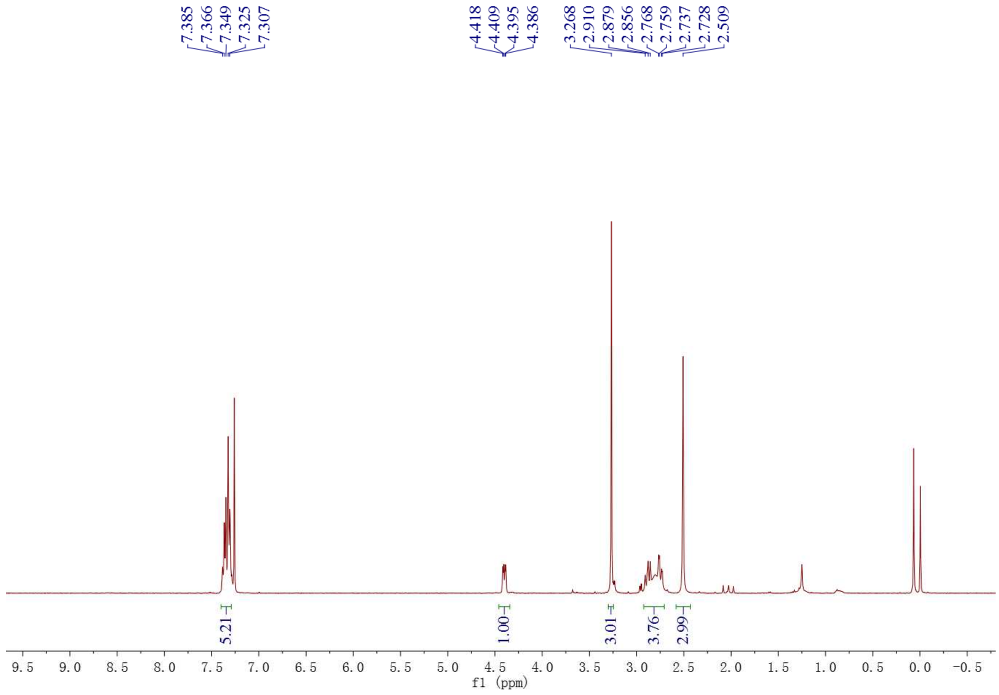
7.385
7.366
7.349
7.325
7.307
4.418
4.409
4.395
4.386
3.268
2.910
2.879
2.856
2.768
2.759
2.737
2.728
2.509
5.21
1.00
3.01
3.76
2.99
9.5 9.0 8.5 8.0 7.5 7.0 6.5 6.0 5.5 5.0 4.5 4.0 3.5 3.0 2.5 2.0 1.5 1.0 0.5 0.0 -0.5
f1 (ppm)

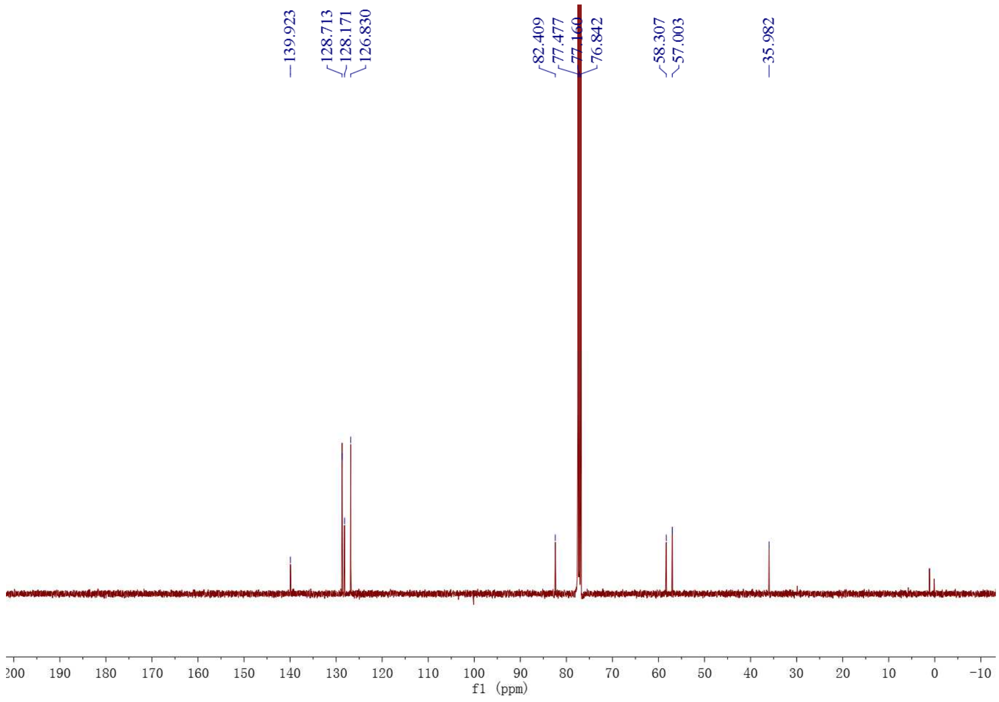
139.923
128.713
128.171
126.830
82.409
77.477
77.160
76.842
58.307
57.003
35.982
200 190 180 170 160 150 140 130 120 110 100 90 80 70 60 50 40 30 20 10 0 -10
f1 (ppm)

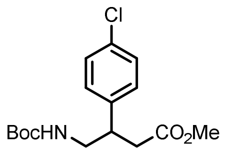

S6

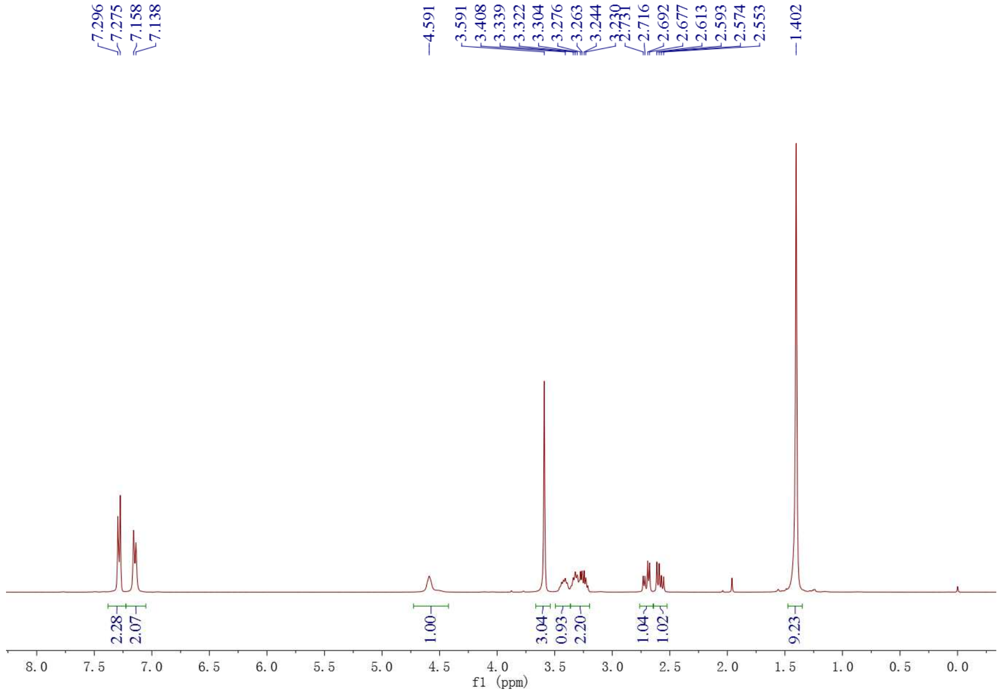

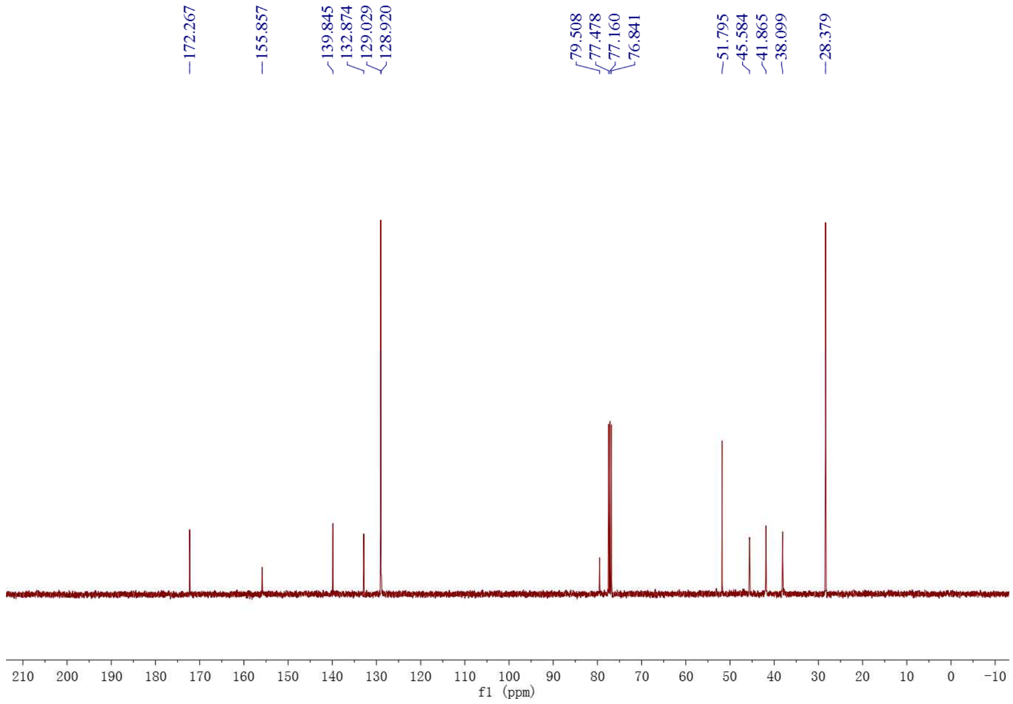

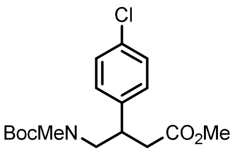
Cl
BocMeN
CO_2Me

S7

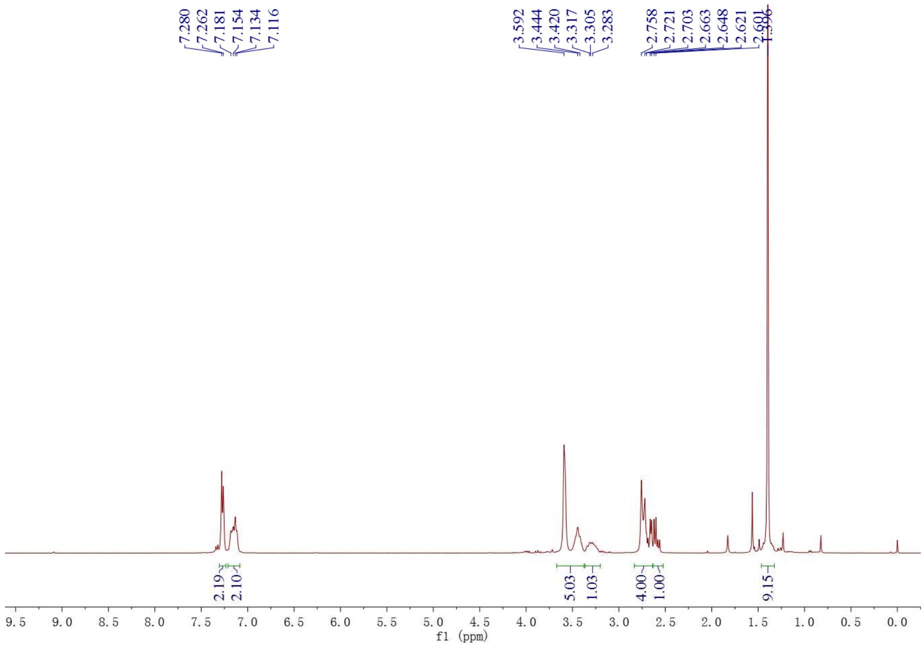
7.280
7.262
7.181
7.154
7.134
7.116
3.592
3.444
3.420
3.317
3.305
3.283
2.758
2.721
2.703
2.663
2.648
2.621
2.601
1.396
2.19
2.10
5.03
1.03
4.00
1.00
9.15
9.5 9.0 8.5 8.0 7.5 7.0 6.5 6.0 5.5 5.0 4.5 4.0 3.5 3.0 2.5 2.0 1.5 1.0 0.5 0.0
f1 (ppm)

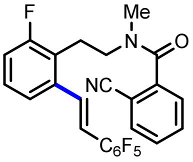
F
Me
N
O
NC
C_6F_5

S9

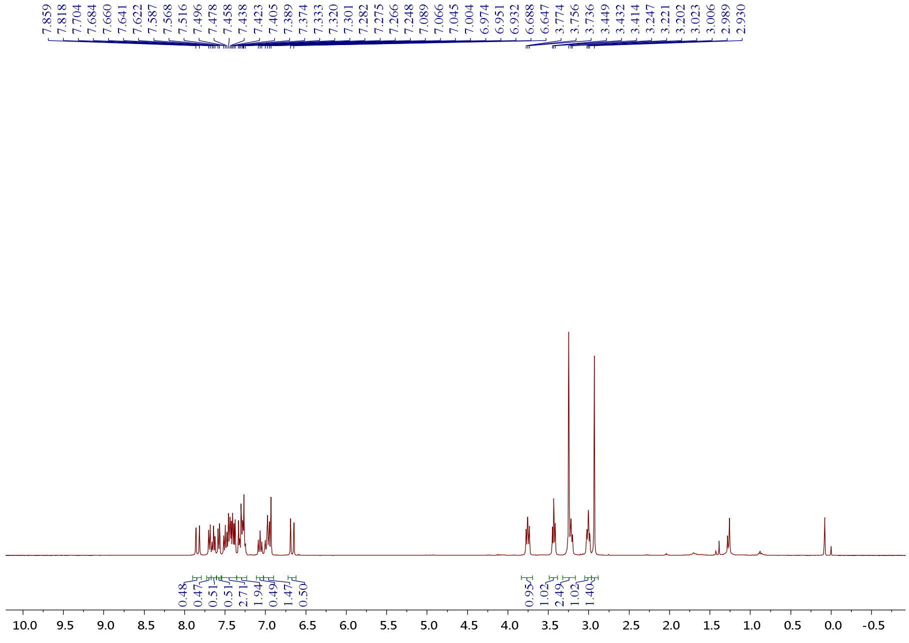
7.859
7.818
7.704
7.684
7.660
7.641
7.622
7.587
7.568
7.516
7.496
7.478
7.458
7.438
7.423
7.405
7.389
7.374
7.333
7.320
7.301
7.282
7.275
7.266
7.248
7.089
7.066
7.045
7.004
6.974
6.951
6.932
6.688
6.647
3.774
3.756
3.736
3.449
3.432
3.414
3.247
3.221
3.202
3.023
3.006
2.989
2.930
0.48
0.47
0.51
0.51
2.71
1.94
0.49
1.47
0.50
0.95
1.02
2.49
1.02
1.40
10.0 9.5 9.0 8.5 8.0 7.5 7.0 6.5 6.0 5.5 5.0 4.5 4.0 3.5 3.0 2.5 2.0 1.5 1.0 0.5 0.0 -0.5

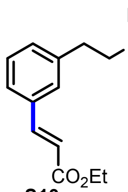
Me
N
H
CO2Et

S10

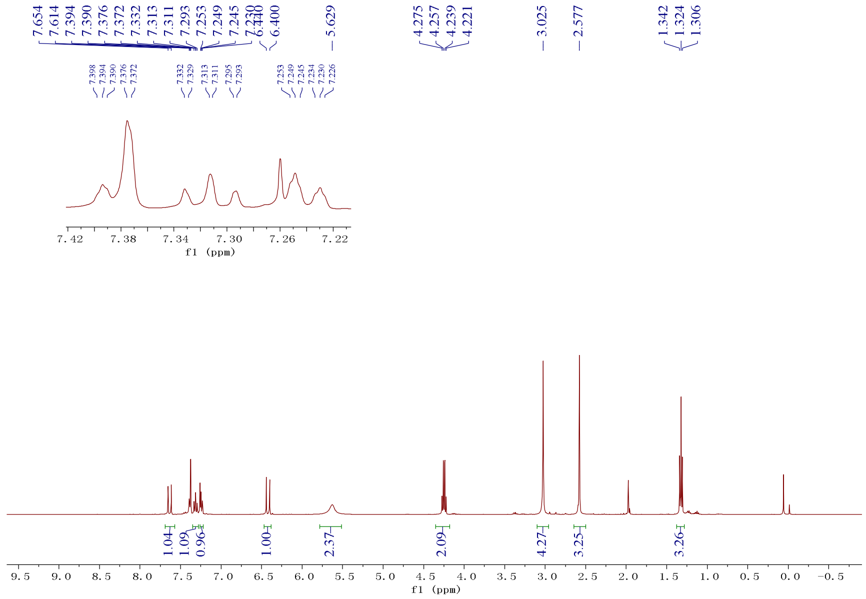
7.654
7.614
7.394
7.390
7.376
7.372
7.332
7.313
7.311
7.293
7.253
7.249
7.245
6.440
6.400
5.629
4.275
4.257
4.239
4.221
3.025
2.577
1.342
1.324
1.306
f1 (ppm)
1.04
1.09
0.96
1.00
2.37
2.09
4.27
3.25
3.26

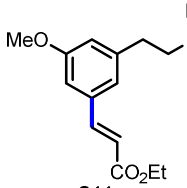
MeO
Me
N
H
CO2Et

S11

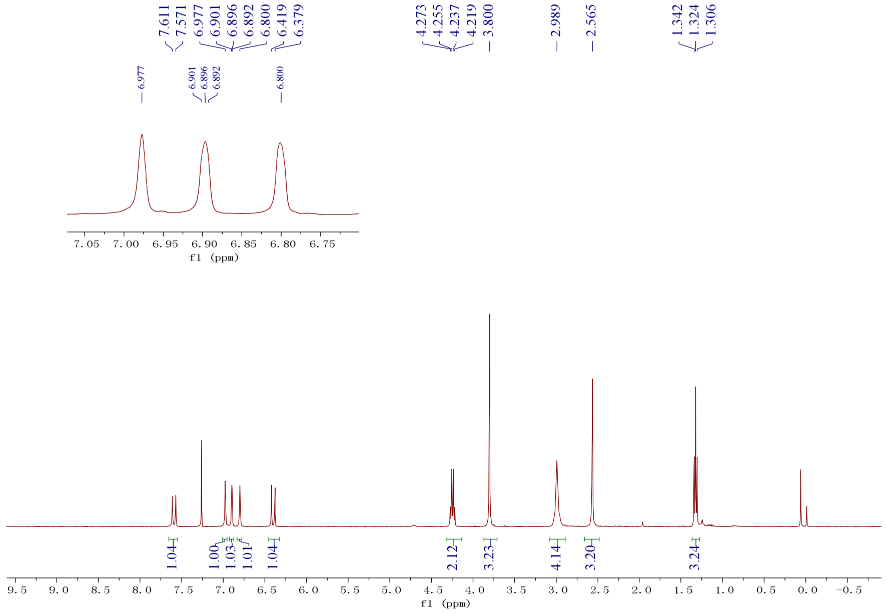
7.611
7.571
6.977
6.901
6.896
6.892
6.800
6.419
6.379
4.273
4.255
4.237
4.219
3.800
2.989
2.565
1.342
1.324
1.306
f1 (ppm)
1.04
1.00
1.03
1.01
1.04
2.12
3.23
4.14
3.20
3.24

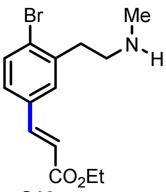

S12

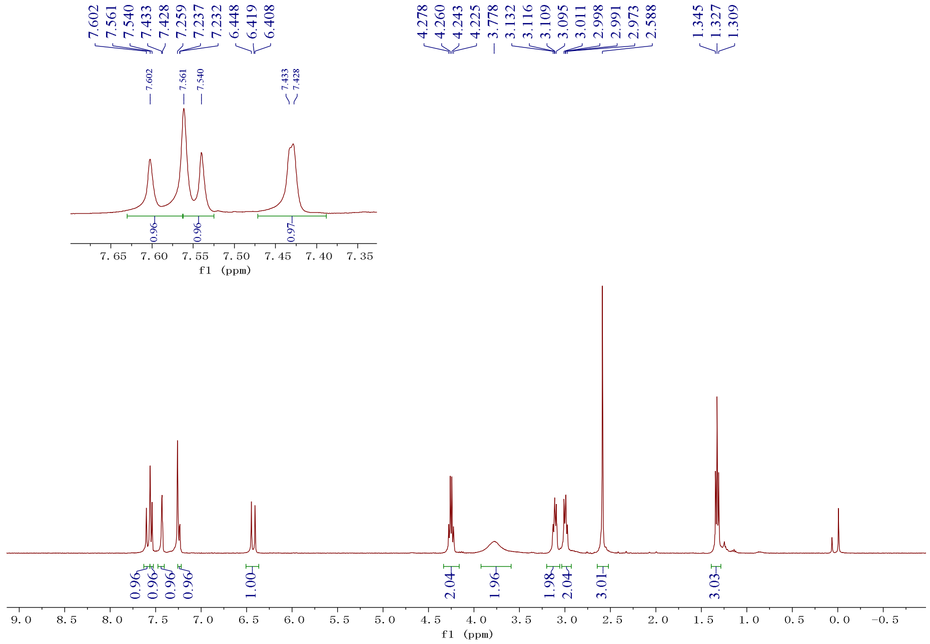

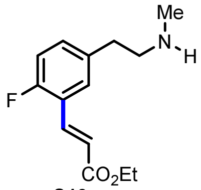

S13

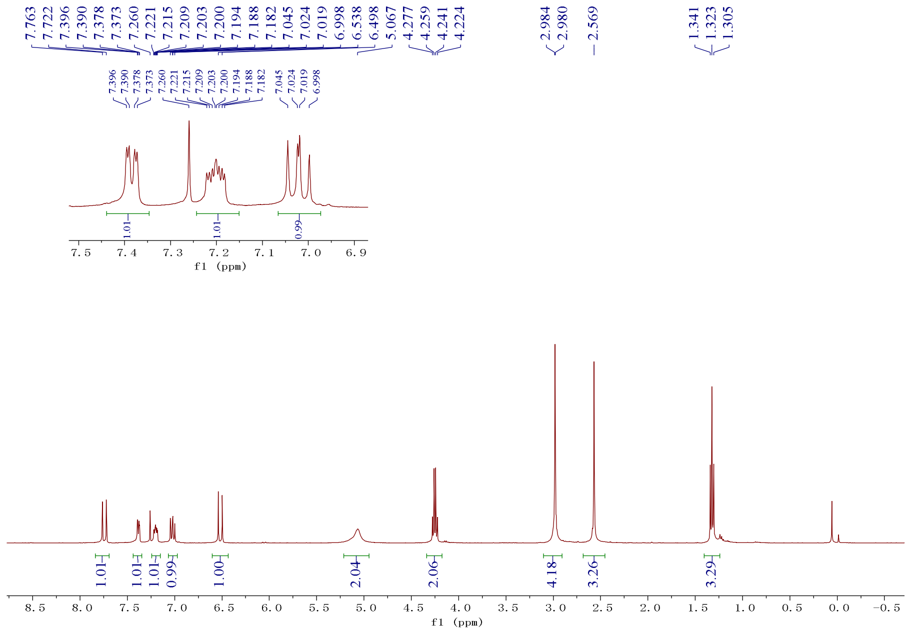

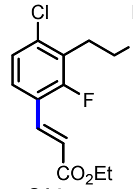

S14

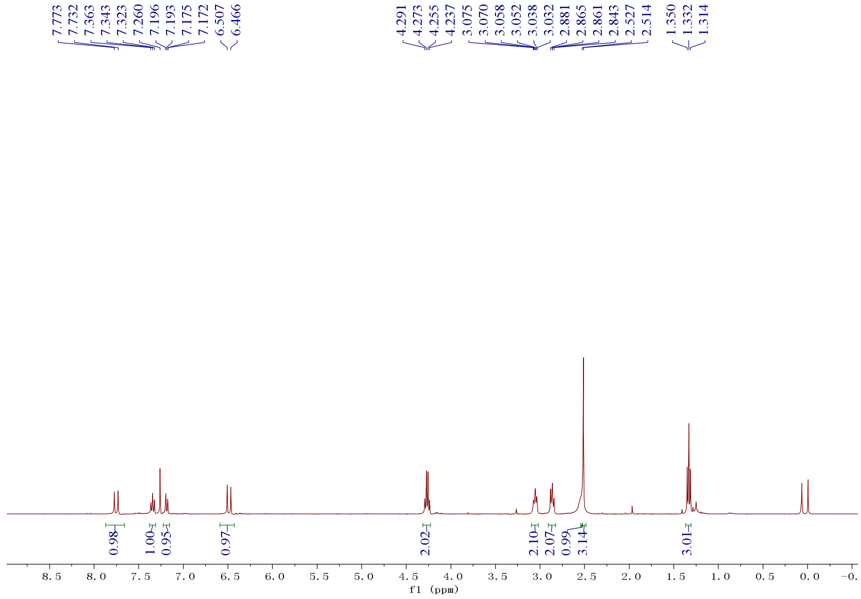

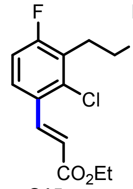

S15

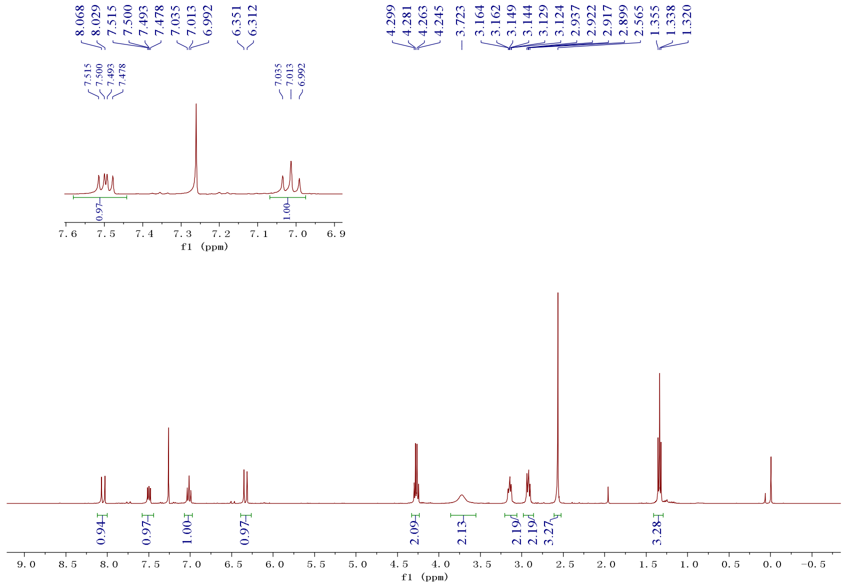

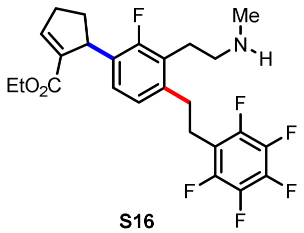

Me
N
H
F
EtO2C
F
F
F
F
F

S16

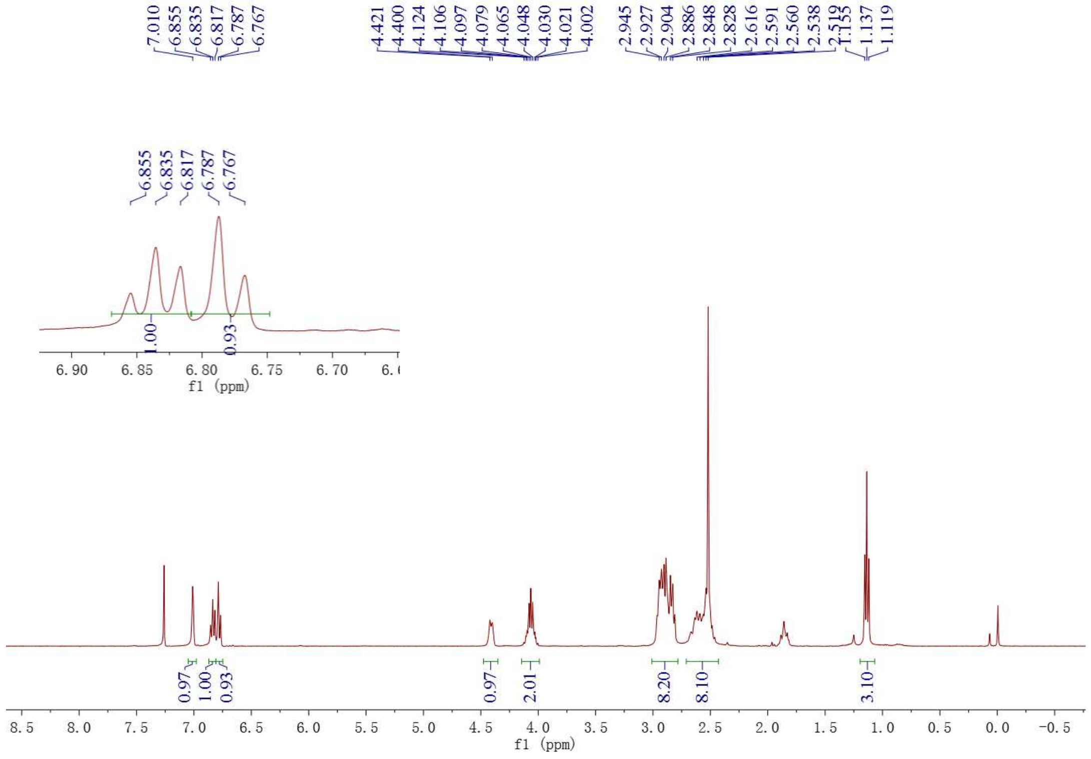

7.010
6.855
6.835
6.817
6.787
6.767
4.421
4.400
4.124
4.106
4.097
4.079
4.065
4.048
4.030
4.021
4.002
2.945
2.927
2.904
2.886
2.848
2.828
2.616
2.591
2.560
2.538
2.519
1.155
1.137
1.119
1.00
0.93
6.90
6.85
6.80
6.75
6.70
f1 (ppm)
0.97
1.00
0.93
0.97
2.01
8.20
8.10
3.10
8.5
8.0
7.5
7.0
6.5
6.0
5.5
5.0
4.5
4.0
3.5
3.0
2.5
2.0
1.5
1.0
0.5
0.0
-0.5
f1 (ppm)

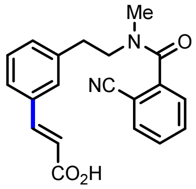
Me
N
O
NC
CO2H

S17

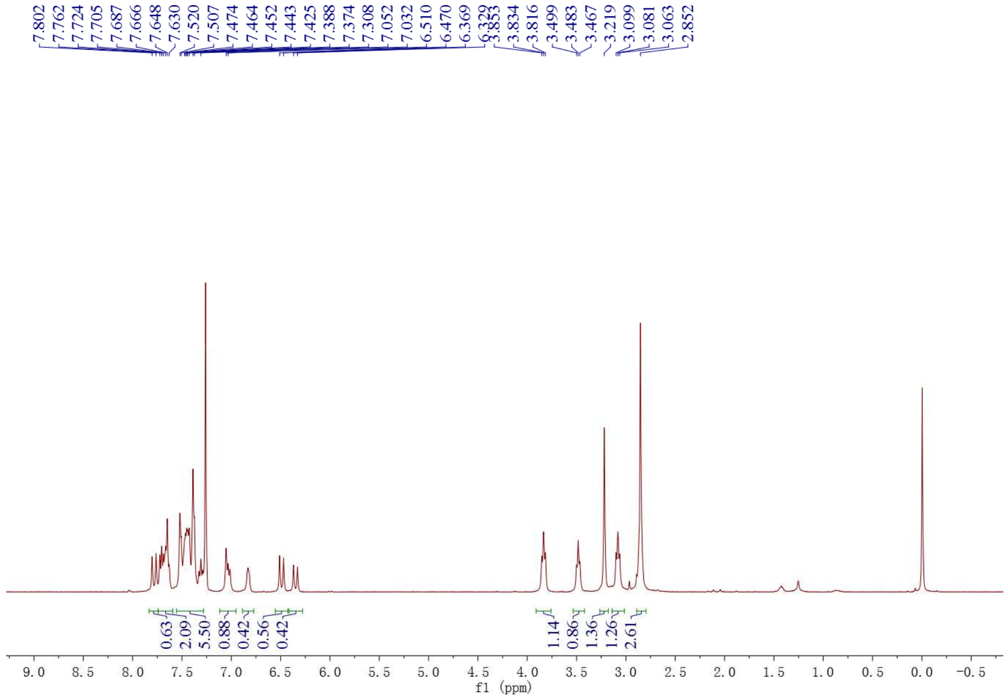
7.802
7.762
7.724
7.705
7.687
7.666
7.648
7.630
7.520
7.507
7.474
7.464
7.452
7.443
7.425
7.388
7.374
7.308
7.052
7.032
6.510
6.470
6.369
6.329
3.853
3.834
3.816
3.499
3.483
3.467
3.219
3.099
3.081
3.063
2.852
0.63
2.09
5.50
0.88
0.42
0.56
0.42
1.14
0.86
1.36
1.26
2.61
9.0 8.5 8.0 7.5 7.0 6.5 6.0 5.5 5.0 4.5 4.0 3.5 3.0 2.5 2.0 1.5 1.0 0.5 0.0 -0.5
f1 (ppm)

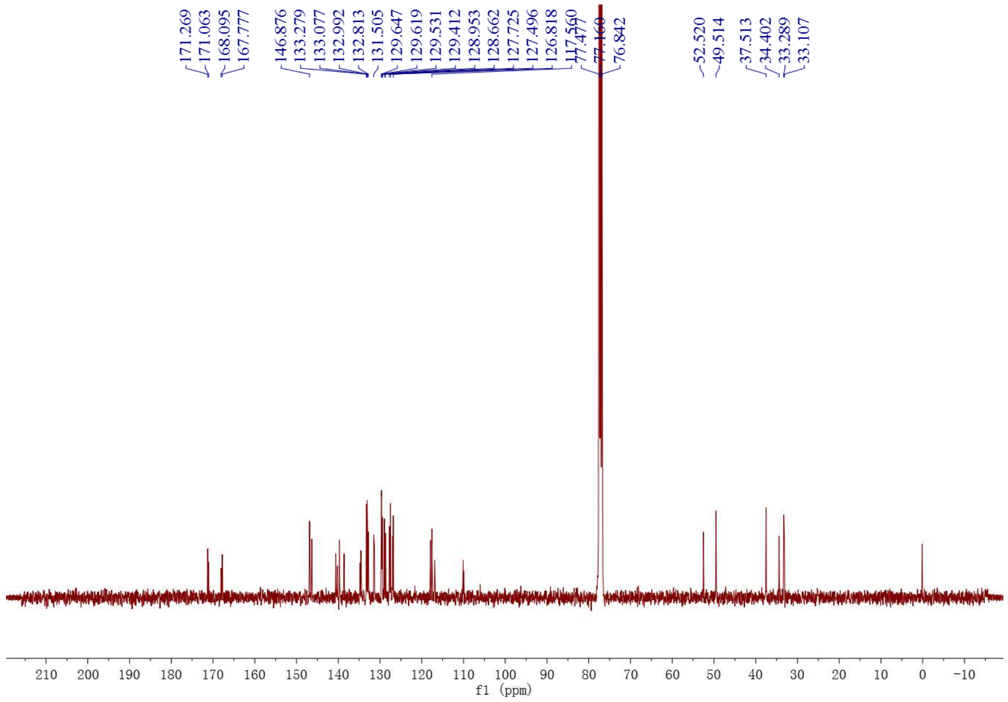
171.269
171.063
168.095
167.777
146.876
133.279
133.077
132.992
132.813
131.505
129.647
129.619
129.531
129.412
128.953
128.662
127.725
127.496
126.818
117.560
77.477
77.160
76.842
52.520
49.514
37.513
34.402
33.289
33.107
210 200 190 180 170 160 150 140 130 120 110 100 90 80 70 60 50 40 30 20 10 0 -10
f1 (ppm)

4. X-Ray Crystallography data for 1a' (CCDC 1056709) and S17 (CCDC 1406595)

1a' (CCDC 1056709):

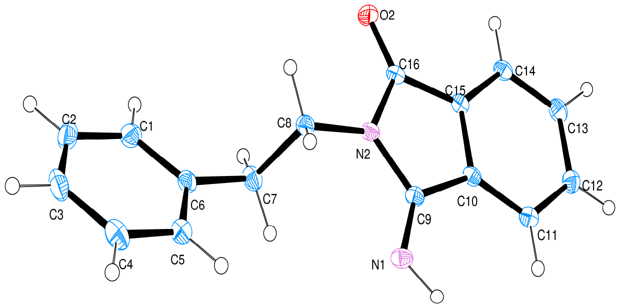

Table S1. Crystal data and structure refinement for Compound **1a'**.

Identification code	Compound-1a	
Empirical formula	C16 H14 N2 O	
Formula weight	250.29	
Temperature	100.0(2) K	
Wavelength	1.54184 Å	
Crystal system	Monoclinic	
Space group	P 21/n	
Unit cell dimensions	a = 13.4616(3) Å	α= 90°.
	b = 4.95900(10) Å	β= 93.710(2)°.
	c = 19.7696(5) Å	γ = 90°.
Volume	1316.98(5) $Å^3$	
Z	4	
Density (calculated)	1.262 Mg/m^3	
Absorption coefficient	0.637 mm^{-1}	
F(000)	528	
Crystal size	0.120 x 0.030 x 0.020 mm^3	
Theta range for data collection	3.859 to 73.251°.	
Index ranges	-15<=h<=16, -3<=k<=5, -20<=l<=24	
Reflections collected	4354	
Independent reflections	2540 [R(int) = 0.0222]	
Completeness to theta = 67.684°	99.9 %	
Absorption correction	Semi-empirical from equivalents	
Max. and min. transmission	1.00000 and 0.87424	
Refinement method	Full-matrix least-squares on F^2	

Data / restraints / parameters	2540 / 0 / 176
Goodness-of-fit on F^2	1.042
Final R indices [I>2sigma(I)]	R1 = 0.0368, wR2 = 0.0911
R indices (all data)	R1 = 0.0474, wR2 = 0.0986
Extinction coefficient	n/a
Largest diff. peak and hole	0.211 and -0.205 e.Å^{-3}

Table S2. Atomic coordinates (x 10^4) and equivalent isotropic displacement parameters ($Å^2$x 10^3) for lg-003_a. U(eq) is defined as one third of the trace of the orthogonalized U^{ij} tensor.

	x	y	z	U(eq)
O(2)	6167(1)	5269(2)	6691(1)	26(1)
N(1)	3017(1)	2771(2)	7234(1)	22(1)
N(2)	4512(1)	4401(2)	6853(1)	19(1)
C(1)	3362(1)	8744(3)	4795(1)	27(1)
C(2)	2802(1)	10768(3)	4462(1)	32(1)
C(3)	1881(1)	11453(3)	4677(1)	33(1)
C(4)	1514(1)	10119(3)	5223(1)	32(1)
C(5)	2073(1)	8106(3)	5555(1)	27(1)
C(6)	3004(1)	7395(3)	5348(1)	22(1)
C(7)	3614(1)	5276(3)	5733(1)	24(1)
C(8)	4060(1)	6445(3)	6403(1)	20(1)
C(9)	3962(1)	2696(3)	7252(1)	18(1)
C(10)	4707(1)	1037(3)	7653(1)	18(1)
C(11)	4585(1)	-984(3)	8126(1)	21(1)
C(12)	5446(1)	-2135(3)	8433(1)	23(1)
C(13)	6390(1)	-1270(3)	8277(1)	23(1)
C(14)	6506(1)	756(3)	7799(1)	21(1)
C(15)	5647(1)	1864(3)	7490(1)	18(1)
C(16)	5531(1)	4030(3)	6971(1)	19(1)

Table S3. Bond lengths [Å] and angles [°] for lg-003_a.

O(2)-C(16)	1.2162(16)
N(1)-C(9)	1.2713(16)
N(1)-H(1N)	0.937(19)
N(2)-C(16)	1.3889(15)
N(2)-C(9)	1.4007(17)
N(2)-C(8)	1.4554(16)
C(1)-C(6)	1.3940(19)
C(1)-C(2)	1.394(2)
C(1)-H(1)	0.9500
C(2)-C(3)	1.378(2)
C(2)-H(2)	0.9500
C(3)-C(4)	1.384(2)
C(3)-H(3)	0.9500
C(4)-C(5)	1.389(2)
C(4)-H(4)	0.9500
C(5)-C(6)	1.3904(19)
C(5)-H(5)	0.9500
C(6)-C(7)	1.5081(19)
C(7)-C(8)	1.5331(18)
C(7)-H(7A)	0.9900
C(7)-H(7B)	0.9900
C(8)-H(8A)	0.9900
C(8)-H(8B)	0.9900
C(9)-C(10)	1.4855(18)
C(10)-C(15)	1.3880(17)
C(10)-C(11)	1.3878(19)
C(11)-C(12)	1.3950(18)
C(11)-H(11)	0.9500
C(12)-C(13)	1.3961(19)
C(12)-H(12)	0.9500
C(13)-C(14)	1.394(2)
C(13)-H(13)	0.9500
C(14)-C(15)	1.3852(18)
C(14)-H(14)	0.9500
C(15)-C(16)	1.4867(19)

C(9)-N(1)-H(1N)	111.5(11)
C(16)-N(2)-C(9)	112.16(11)
C(16)-N(2)-C(8)	124.34(11)
C(9)-N(2)-C(8)	123.37(10)
C(6)-C(1)-C(2)	120.79(14)
C(6)-C(1)-H(1)	119.6
C(2)-C(1)-H(1)	119.6
C(3)-C(2)-C(1)	120.10(14)
C(3)-C(2)-H(2)	119.9
C(1)-C(2)-H(2)	119.9
C(2)-C(3)-C(4)	119.74(14)
C(2)-C(3)-H(3)	120.1
C(4)-C(3)-H(3)	120.1
C(3)-C(4)-C(5)	120.17(14)
C(3)-C(4)-H(4)	119.9
C(5)-C(4)-H(4)	119.9
C(4)-C(5)-C(6)	120.93(13)
C(4)-C(5)-H(5)	119.5
C(6)-C(5)-H(5)	119.5
C(5)-C(6)-C(1)	118.27(13)
C(5)-C(6)-C(7)	119.96(12)
C(1)-C(6)-C(7)	121.74(13)
C(6)-C(7)-C(8)	110.03(11)
C(6)-C(7)-H(7A)	109.7
C(8)-C(7)-H(7A)	109.7
C(6)-C(7)-H(7B)	109.7
C(8)-C(7)-H(7B)	109.7
H(7A)-C(7)-H(7B)	108.2
N(2)-C(8)-C(7)	112.95(11)
N(2)-C(8)-H(8A)	109.0
C(7)-C(8)-H(8A)	109.0
N(2)-C(8)-H(8B)	109.0
C(7)-C(8)-H(8B)	109.0
H(8A)-C(8)-H(8B)	107.8
N(1)-C(9)-N(2)	122.07(12)
N(1)-C(9)-C(10)	132.16(12)
N(2)-C(9)-C(10)	105.77(10)
C(15)-C(10)-C(11)	121.25(12)

C(15)-C(10)-C(9)	107.89(11)
C(11)-C(10)-C(9)	130.85(11)
C(10)-C(11)-C(12)	117.26(12)
C(10)-C(11)-H(11)	121.4
C(12)-C(11)-H(11)	121.4
C(11)-C(12)-C(13)	121.35(13)
C(11)-C(12)-H(12)	119.3
C(13)-C(12)-H(12)	119.3
C(14)-C(13)-C(12)	121.00(12)
C(14)-C(13)-H(13)	119.5
C(12)-C(13)-H(13)	119.5
C(15)-C(14)-C(13)	117.23(12)
C(15)-C(14)-H(14)	121.4
C(13)-C(14)-H(14)	121.4
C(14)-C(15)-C(10)	121.90(13)
C(14)-C(15)-C(16)	129.66(12)
C(10)-C(15)-C(16)	108.44(11)
O(2)-C(16)-N(2)	125.01(12)
O(2)-C(16)-C(15)	129.27(12)
N(2)-C(16)-C(15)	105.72(10)

Symmetry transformations used to generate equivalent atoms:

Table S4. Anisotropic displacement parameters ($Å^2$x 10^3)for lg-003_a. The anisotropic displacement factor exponent takes the form: $-2\pi^2[h^2a^{*2}U^{11} + ... + 2 h k a^* b^* U^{12}]$

	U^{11}	U^{22}	U^{33}	U^{23}	U^{13}	U^{12}
O(2)	18(1)	32(1)	29(1)	5(1)	2(1)	-5(1)
N(1)	14(1)	20(1)	30(1)	-1(1)	2(1)	1(1)
N(2)	14(1)	19(1)	23(1)	0(1)	-1(1)	-1(1)
C(1)	30(1)	28(1)	23(1)	-3(1)	1(1)	-6(1)
C(2)	49(1)	26(1)	21(1)	3(1)	-5(1)	-10(1)
C(3)	42(1)	24(1)	29(1)	0(1)	-16(1)	2(1)
C(4)	25(1)	37(1)	33(1)	-2(1)	-6(1)	5(1)
C(5)	24(1)	31(1)	26(1)	4(1)	-1(1)	-2(1)
C(6)	25(1)	19(1)	21(1)	-2(1)	-4(1)	-2(1)
C(7)	26(1)	21(1)	25(1)	-3(1)	-4(1)	2(1)
C(8)	19(1)	17(1)	24(1)	1(1)	-2(1)	0(1)
C(9)	17(1)	16(1)	21(1)	-3(1)	0(1)	0(1)
C(10)	16(1)	17(1)	20(1)	-4(1)	-1(1)	1(1)
C(11)	18(1)	21(1)	23(1)	-3(1)	1(1)	-1(1)
C(12)	25(1)	22(1)	22(1)	1(1)	0(1)	2(1)
C(13)	18(1)	26(1)	24(1)	-1(1)	-3(1)	5(1)
C(14)	14(1)	25(1)	25(1)	-1(1)	-1(1)	1(1)
C(15)	16(1)	20(1)	19(1)	-3(1)	0(1)	0(1)
C(16)	15(1)	21(1)	22(1)	-4(1)	-1(1)	-1(1)

S17 (CCDC 1406595):

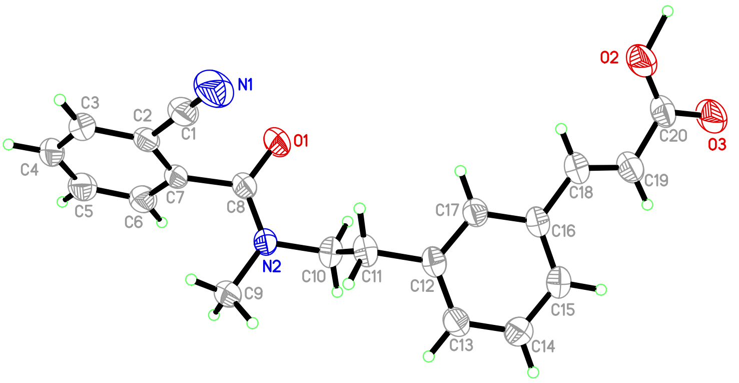

Table 1. Crystal data and structure refinement for 1_a.

Identification code	compound-1	
Empirical formula	C20 H18 N2 O3	
Formula weight	334.36	
Temperature	293(2) K	
Wavelength	0.71073 Å	
Crystal system	Monoclinic	
Space group	C 2/c	
Unit cell dimensions	a = 33.970(16) Å	α= 90°.
	b = 6.299(3) Å	β= 92.257(8)°.
	c = 16.431(8) Å	γ = 90°.
Volume	3513(3) $Å^3$	
Z	8	
Density (calculated)	1.264 Mg/m^3	
Absorption coefficient	0.086 mm^{-1}	
F(000)	1408	
Crystal size	0.200 x 0.180 x 0.150 mm^3	
Theta range for data collection	2.400 to 27.516°.	
Index ranges	-44<=h<=42, -8<=k<=8, -20<=l<=21	
Reflections collected	12961	
Independent reflections	4016 [R(int) = 0.0269]	
Completeness to theta = 25.242°	99.7 %	
Absorption correction	Semi-empirical from equivalents	
Max. and min. transmission	1.0000 and 0.9560	
Refinement method	Full-matrix least-squares on F^2	
Data / restraints / parameters	4016 / 0 / 230	

Goodness-of-fit on F^2	1.090
Final R indices [I>2sigma(I)]	R1 = 0.0586, wR2 = 0.1406
R indices (all data)	R1 = 0.0856, wR2 = 0.1579
Extinction coefficient	n/a
Largest diff. peak and hole	0.202 and -0.178 e.$Å^{-3}$

Table 2. Atomic coordinates (x 10^4) and equivalent isotropic displacement parameters ($Å^2$x 10^3) for 1_a. U(eq) is defined as one third of the trace of the orthogonalized U^{ij} tensor.

	x	y	z	U(eq)
O(1)	6374(1)	358(3)	4870(1)	100(1)
O(2)	5338(1)	548(3)	9311(1)	91(1)
O(3)	4831(1)	2477(3)	9675(1)	92(1)
N(1)	7374(1)	36(4)	5463(1)	97(1)
N(2)	6407(1)	3734(2)	4426(1)	51(1)
C(1)	7270(1)	70(3)	4796(1)	65(1)
C(2)	7135(1)	118(3)	3949(1)	51(1)
C(3)	7372(1)	-688(3)	3355(1)	62(1)
C(4)	7239(1)	-648(3)	2551(1)	68(1)
C(5)	6876(1)	156(3)	2344(1)	66(1)
C(6)	6640(1)	968(3)	2932(1)	60(1)
C(7)	6768(1)	960(3)	3743(1)	49(1)
C(8)	6499(1)	1682(3)	4398(1)	58(1)
C(9)	6603(1)	5366(3)	3965(1)	62(1)
C(10)	6134(1)	4463(3)	5033(1)	57(1)
C(11)	6344(1)	5200(3)	5814(1)	58(1)
C(12)	6052(1)	5940(3)	6419(1)	52(1)
C(13)	5885(1)	7952(3)	6357(1)	58(1)
C(14)	5618(1)	8629(3)	6919(1)	62(1)
C(15)	5510(1)	7305(3)	7541(1)	58(1)
C(16)	5659(1)	5249(3)	7599(1)	52(1)
C(17)	5934(1)	4611(3)	7038(1)	53(1)
C(18)	5535(1)	3720(3)	8211(1)	58(1)
C(19)	5234(1)	3871(4)	8687(1)	65(1)
C(20)	5133(1)	2203(4)	9259(1)	63(1)

Table 3. Bond lengths [Å] and angles [°] for 1_a.

O(1)-C(8)	1.226(2)
O(2)-C(20)	1.254(3)
O(2)-H(2O)	1.26(3)
O(3)-C(20)	1.268(2)
N(1)-C(1)	1.139(3)
N(2)-C(8)	1.331(2)
N(2)-C(9)	1.454(2)
N(2)-C(10)	1.462(2)
C(1)-C(2)	1.448(3)
C(2)-C(3)	1.386(3)
C(2)-C(7)	1.387(3)
C(3)-C(4)	1.380(3)
C(3)-H(3)	0.9500
C(4)-C(5)	1.363(3)
C(4)-H(4)	0.9500
C(5)-C(6)	1.378(3)
C(5)-H(5)	0.9500
C(6)-C(7)	1.385(3)
C(6)-H(6)	0.9500
C(7)-C(8)	1.508(2)
C(9)-H(9A)	0.9800
C(9)-H(9B)	0.9800
C(9)-H(9C)	0.9800
C(10)-C(11)	1.517(3)
C(10)-H(10A)	0.9900
C(10)-H(10B)	0.9900
C(11)-C(12)	1.506(2)
C(11)-H(11A)	0.9900
C(11)-H(11B)	0.9900
C(12)-C(17)	1.387(2)
C(12)-C(13)	1.390(3)
C(13)-C(14)	1.387(3)
C(13)-H(13)	0.9500
C(14)-C(15)	1.380(3)
C(14)-H(14)	0.9500
C(15)-C(16)	1.393(3)

C(15)-H(15)	0.9500
C(16)-C(17)	1.397(2)
C(16)-C(18)	1.467(3)
C(17)-H(17)	0.9500
C(18)-C(19)	1.314(3)
C(18)-H(18)	0.9500
C(19)-C(20)	1.459(3)
C(19)-H(19)	0.9500

C(20)-O(2)-H(2O)	122.8(11)
C(8)-N(2)-C(9)	123.76(16)
C(8)-N(2)-C(10)	119.01(15)
C(9)-N(2)-C(10)	116.66(15)
N(1)-C(1)-C(2)	179.6(3)
C(3)-C(2)-C(7)	120.70(16)
C(3)-C(2)-C(1)	119.92(18)
C(7)-C(2)-C(1)	119.38(17)
C(4)-C(3)-C(2)	119.3(2)
C(4)-C(3)-H(3)	120.3
C(2)-C(3)-H(3)	120.3
C(5)-C(4)-C(3)	120.31(18)
C(5)-C(4)-H(4)	119.8
C(3)-C(4)-H(4)	119.8
C(4)-C(5)-C(6)	120.64(18)
C(4)-C(5)-H(5)	119.7
C(6)-C(5)-H(5)	119.7
C(5)-C(6)-C(7)	120.21(19)
C(5)-C(6)-H(6)	119.9
C(7)-C(6)-H(6)	119.9
C(6)-C(7)-C(2)	118.80(16)
C(6)-C(7)-C(8)	120.65(18)
C(2)-C(7)-C(8)	120.35(16)
O(1)-C(8)-N(2)	123.41(18)
O(1)-C(8)-C(7)	118.74(18)
N(2)-C(8)-C(7)	117.85(15)
N(2)-C(9)-H(9A)	109.5
N(2)-C(9)-H(9B)	109.5
H(9A)-C(9)-H(9B)	109.5

N(2)-C(9)-H(9C)	109.5
H(9A)-C(9)-H(9C)	109.5
H(9B)-C(9)-H(9C)	109.5
N(2)-C(10)-C(11)	112.59(15)
N(2)-C(10)-H(10A)	109.1
C(11)-C(10)-H(10A)	109.1
N(2)-C(10)-H(10B)	109.1
C(11)-C(10)-H(10B)	109.1
H(10A)-C(10)-H(10B)	107.8
C(12)-C(11)-C(10)	110.68(15)
C(12)-C(11)-H(11A)	109.5
C(10)-C(11)-H(11A)	109.5
C(12)-C(11)-H(11B)	109.5
C(10)-C(11)-H(11B)	109.5
H(11A)-C(11)-H(11B)	108.1
C(17)-C(12)-C(13)	118.38(17)
C(17)-C(12)-C(11)	120.85(18)
C(13)-C(12)-C(11)	120.74(17)
C(14)-C(13)-C(12)	120.49(18)
C(14)-C(13)-H(13)	119.8
C(12)-C(13)-H(13)	119.8
C(15)-C(14)-C(13)	120.43(19)
C(15)-C(14)-H(14)	119.8
C(13)-C(14)-H(14)	119.8
C(14)-C(15)-C(16)	120.43(17)
C(14)-C(15)-H(15)	119.8
C(16)-C(15)-H(15)	119.8
C(15)-C(16)-C(17)	118.25(17)
C(15)-C(16)-C(18)	122.90(17)
C(17)-C(16)-C(18)	118.84(18)
C(12)-C(17)-C(16)	121.95(18)
C(12)-C(17)-H(17)	119.0
C(16)-C(17)-H(17)	119.0
C(19)-C(18)-C(16)	127.8(2)
C(19)-C(18)-H(18)	116.1
C(16)-C(18)-H(18)	116.1
C(18)-C(19)-C(20)	122.6(2)
C(18)-C(19)-H(19)	118.7

C(20)-C(19)-H(19)	118.7
O(2)-C(20)-O(3)	122.4(2)
O(2)-C(20)-C(19)	119.91(19)
O(3)-C(20)-C(19)	117.7(2)

Symmetry transformations used to generate equivalent atoms:

Table 4. Anisotropic displacement parameters ($Å^2 x 10^3$)for 1_a. The anisotropic displacement factor exponent takes the form: $-2\pi^2[h^2a^{*2}U^{11} + ... + 2 h k a^* b^* U^{12}]$

	U^{11}	U^{22}	U^{33}	U^{23}	U^{13}	U^{12}
O(1)	143(2)	59(1)	105(1)	17(1)	77(1)	0(1)
O(2)	92(1)	100(1)	82(1)	30(1)	40(1)	20(1)
O(3)	83(1)	106(1)	91(1)	29(1)	45(1)	17(1)
N(1)	137(2)	100(2)	54(1)	10(1)	-6(1)	4(1)
N(2)	58(1)	49(1)	48(1)	-2(1)	16(1)	-3(1)
C(1)	85(2)	59(1)	51(1)	7(1)	8(1)	2(1)
C(2)	70(1)	40(1)	44(1)	5(1)	10(1)	-3(1)
C(3)	72(1)	57(1)	58(1)	7(1)	16(1)	11(1)
C(4)	97(2)	57(1)	52(1)	0(1)	27(1)	10(1)
C(5)	96(2)	59(1)	43(1)	-2(1)	3(1)	2(1)
C(6)	69(1)	58(1)	52(1)	3(1)	3(1)	2(1)
C(7)	63(1)	38(1)	46(1)	2(1)	15(1)	-3(1)
C(8)	72(1)	48(1)	55(1)	3(1)	21(1)	-5(1)
C(9)	80(1)	49(1)	59(1)	3(1)	19(1)	-4(1)
C(10)	52(1)	67(1)	52(1)	-9(1)	11(1)	1(1)
C(11)	55(1)	75(1)	46(1)	-1(1)	10(1)	-1(1)
C(12)	52(1)	64(1)	41(1)	-3(1)	5(1)	-6(1)
C(13)	63(1)	62(1)	49(1)	5(1)	7(1)	-5(1)
C(14)	62(1)	60(1)	64(1)	0(1)	6(1)	4(1)
C(15)	55(1)	68(1)	51(1)	-7(1)	10(1)	0(1)
C(16)	52(1)	64(1)	41(1)	-2(1)	7(1)	-7(1)
C(17)	59(1)	56(1)	44(1)	-1(1)	6(1)	-2(1)
C(18)	58(1)	72(1)	44(1)	0(1)	7(1)	-6(1)
C(19)	64(1)	80(2)	52(1)	10(1)	9(1)	2(1)
C(20)	56(1)	85(2)	48(1)	7(1)	11(1)	1(1)

Table 5. Hydrogen coordinates (x 10^4) and isotropic displacement parameters ($Å^2$x 10^3) for 1_a.

	x	y	z	U(eq)
H(2O)	5263(7)	-960(40)	9787(16)	109
H(3)	7624	-1263	3500	75
H(4)	7401	-1184	2139	82
H(5)	6785	156	1790	79
H(6)	6388	1536	2780	72
H(9A)	6868	4881	3839	94
H(9B)	6623	6671	4289	94
H(9C)	6451	5646	3457	94
H(10A)	5952	3291	5159	68
H(10B)	5975	5648	4801	68
H(11A)	6502	4018	6052	70
H(11B)	6526	6378	5692	70
H(13)	5954	8869	5926	70
H(14)	5508	10014	6875	74
H(15)	5333	7798	7932	69
H(17)	6043	3225	7080	63
H(18)	5690	2471	8270	70
H(19)	5076	5115	8658	78

Table 6. Hydrogen bonds for 1_a [Å and °].

D-H...A	d(D-H)	d(H...A)	d(D...A)	<(DHA)
O(2)-H(2O)...O(2)#1	1.26(3)	2.58(3)	3.361(3)	117.7(15)
O(2)-H(2O)...O(3)#1	1.26(3)	1.35(3)	2.610(2)	176(2)

Symmetry transformations used to generate equivalent atoms:

#1 -x+1,-y,-z+2
